# Connective synthesis of 5,5-disubstituted hydantoins by tandem α-amination and α-arylation of silyl ketene acetals†
†Electronic supplementary information (ESI) available. CCDC 1867365 and 1867366. For ESI and crystallographic data in CIF or other electronic format see DOI: 10.1039/c8sc05263h


**DOI:** 10.1039/c8sc05263h

**Published:** 2019-02-06

**Authors:** Rakesh K. Saunthwal, Matthew T. Cornall, Roman Abrams, John W. Ward, Jonathan Clayden

**Affiliations:** a School of Chemistry , University of Bristol , Cantock's Close , Bristol BS8 1TS , UK . Email: john.ward@liverpool.ac.uk ; Email: j.clayden@bristol.ac.uk

## Abstract

5,5-Disubstituted hydantoins, formally the cyclisation products of quaternary amino acids, were formed connectively from simple ester-derived starting materials by a one-pot tandem method. Amination of the silyl ketene acetal derivative of a methyl ester takes place by silver-catalysed addition to the N=N bond of an azocarboxamide, generating a *N*-amino-*N*′-aryl urea derivative of a substituted aminoester. Treatment with a base forms an ester enolate which undergoes arylation by intramolecular migration of an aryl ring to the α-position of the ester. The product undergoes ring closure to a hydantoin, which may itself be deprotected and functionalised. Aryl migration is successful with rings of various electronic character and with esters bearing functionalised and unfunctionalised chains, and the products have features in common with several bioactive compounds.

## Abstract 2

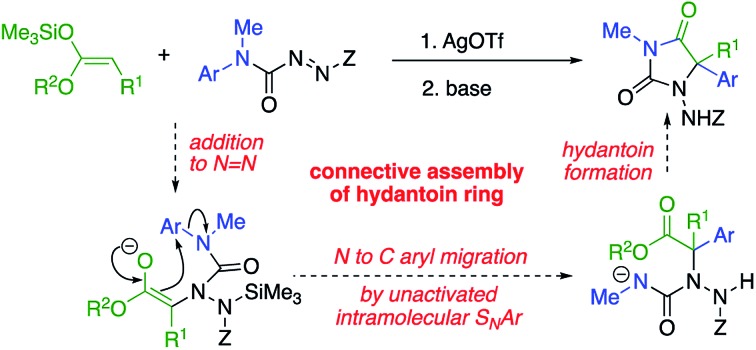
Amination of a silylated ester generates an intermediate urea that transfers an aryl ring to the aminated centre and cyclises to a hydantoin.

## Introduction

**Fig. 1:**
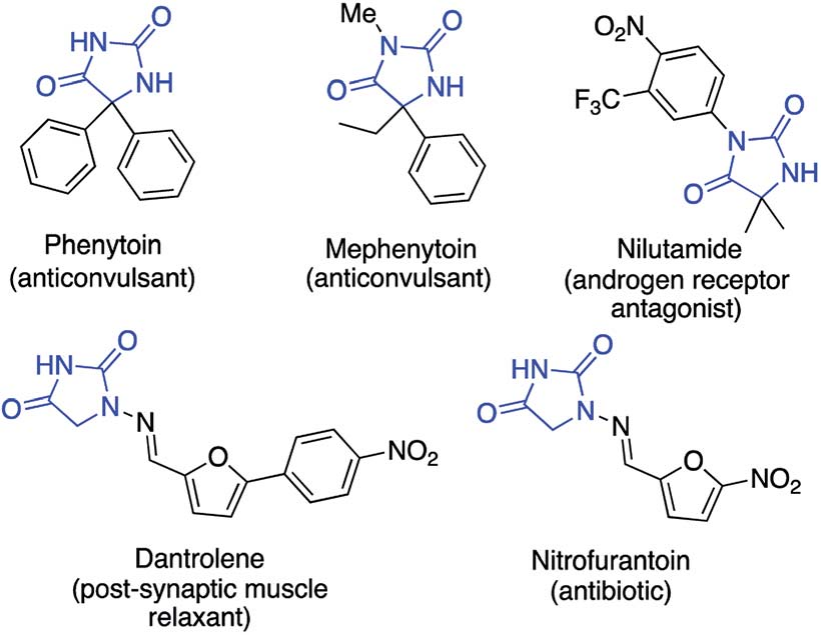
Examples of drugs containing the hydantoin motif.

Hydantoin rings, formally the cyclocarbonylation products of amino acids, are found in a number of medicinally significant molecules (Fig. 1).^1^,^2^ For example the sodium salts of phenytoin and fosphenytoin have anticonvulsant and antiarrhythmic properties;3*a* nitrofurantoin is an antibacterial drug;3b,c nilutamide is an androgen receptor antagonist for the treatment of advanced prostate cancer;^4^ dantrolene is used as a muscle relaxant and to prevent malignant hyperthermia.^5^ Substituted hydantoins are furthermore valuable intermediates in the synthesis of amino acids using hydantoinases and other related biocatalysts.^6^

Methods for the synthesis of substituted hydantoins^7^ include the classical Urech synthesis,^8^ and the Bucherer–Bergs^9^ and Biltz reactions.^10^ Milder transition metal catalysed approaches have been reported, including the Ugi condensation,^11^ an aminobarbituric acid-hydantoin rearrangement,^12^ and reactions of activated carboxylic acids.^13^ Hydantoins have also been made by α-amination of esters using copper catalysts.^14^,^15^

We have shown that intramolecular migration of an aryl ring to the α-position of an amino acid-derived urea can provide a general method for making substituted hydantoins^16^ in reactions that involve intramolecular nucleophilic aromatic substitution of enolates on even unactivated aromatic rings.^17^ However, such methods make use of available amino acid starting materials and are less applicable to the synthesis of molecules containing ‘non-proteinogenic’ side chains. We therefore sought to develop a tandem approach from simple precursors in which the α-amination^18^ of an enolate generates a suitable substrate for a tandem intramolecular arylation,^19^ leading directly to an α-arylated quaternary hydantoin.^20^

**Scheme 1:**
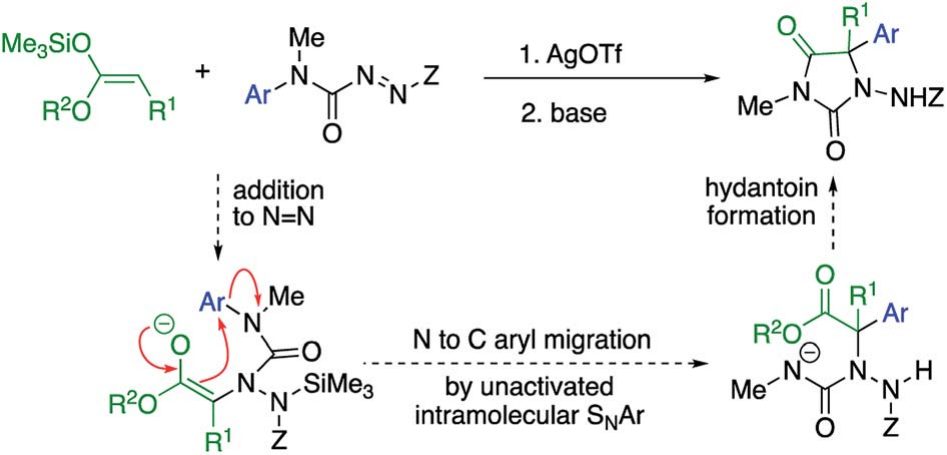
A route to α-arylated quaternary hydantoins by tandem amination-arylation of silyl ketene acetals. Z = electron-withdrawing group.

Our initial plan for a direct route to structurally diverse 5,5-disubstituted hydantoins is illustrated in Scheme 1. We aimed to initiate the hydantoin synthesis with a silver-catalysed regioselective α-amination using an azocarboxamide to generate a urea derivative from which *N*′-aryl migration to the α-position of the resulting ester followed by ring closure would give a hydantoin.

Three major challenges need to be overcome. Regioselective addition of the silyl ketene acetal to the azocarboxamide must lead to a 2-ureido ester to allow the subsequent aryl migration step. Secondly, the enolate of the product must undergo rearrangement rather than any other alternative reaction (such as substitution or elimination), and finally the product must cyclise to a hydantoin. All these steps ideally should occur in a single, tandem process.

## Results and discussion

We started by exploring the amination step with a symmetrical azodicarboxamide to allow us to study the viability of the rearrangement while avoiding issues of regioselectivity. Silver-catalysed aminations of silyl ketene acetals were known using azodicarboxylates,^21^ so azodicarboxamides **1** were made by acylation of hydrazine with *N*-methyl-*N*-arylcarbamoyl chloride followed by oxidation with NBS.^22^ Treatment of a mixture of the azodicarboxamide **1a** and the silyl ketene acetal **2a** with AgOTf (20 mol%) in dichloromethane gave the addition product **3** in 68% yield (Table 1, entry 1). Reducing the catalyst loading to 10 mol% in THF improved this yield to 80% (entry 2). **3** carries an *N*′-aryl urea function suitably located for possible aryl transfer to an enolate derivative. We therefore added 2.0 equiv. KHMDS to the reaction mixture in the hope of promoting this intramolecular arylation. The rearranged product **4a** was obtained in 60% yield, together with 40% of intermediate **3** (entry 3). Increasing the amount of KHMDS to 3.0 equiv. gave clean product **4a** in 75% yield (entry 4).

**Table 1:** Optimising the amination and rearrangement^*a*^

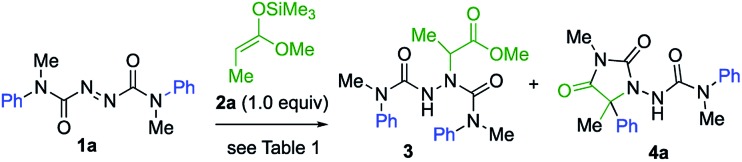			
Entry	Reaction conditions	Yields^*b*^ (%)	
		**3**	**4a**
1	20 mol% AgOTf, CH_2_Cl_2_, –78 → +20 °C, 3 h	68	—
2	10 mol% AgOTf, THF, –78 → +20 °C, 3 h	80^*c*^	—
3	(1) 10 mol% AgOTf, THF, –78 → +20 °C, 3 h	40	60
	(2) KHMDS (2.0 equiv.), –78 → –40 °C, 3 h		
4	(1) 10 mol% AgOTf, THF, –78 → +20 °C, 3 h	0	75
	(2) KHMDS (3.0 equiv.), –78 → –40 °C, 3 h		

^*a*^Reactions performed using 0.34 mmol of **1a** and 0.34 mmol of **2a** in 3.4 ml solvent.

^*b*^Isolated yield.

^*c*^Similar results obtained with 15 or 20 mol% catalyst.

**Scheme 2:**
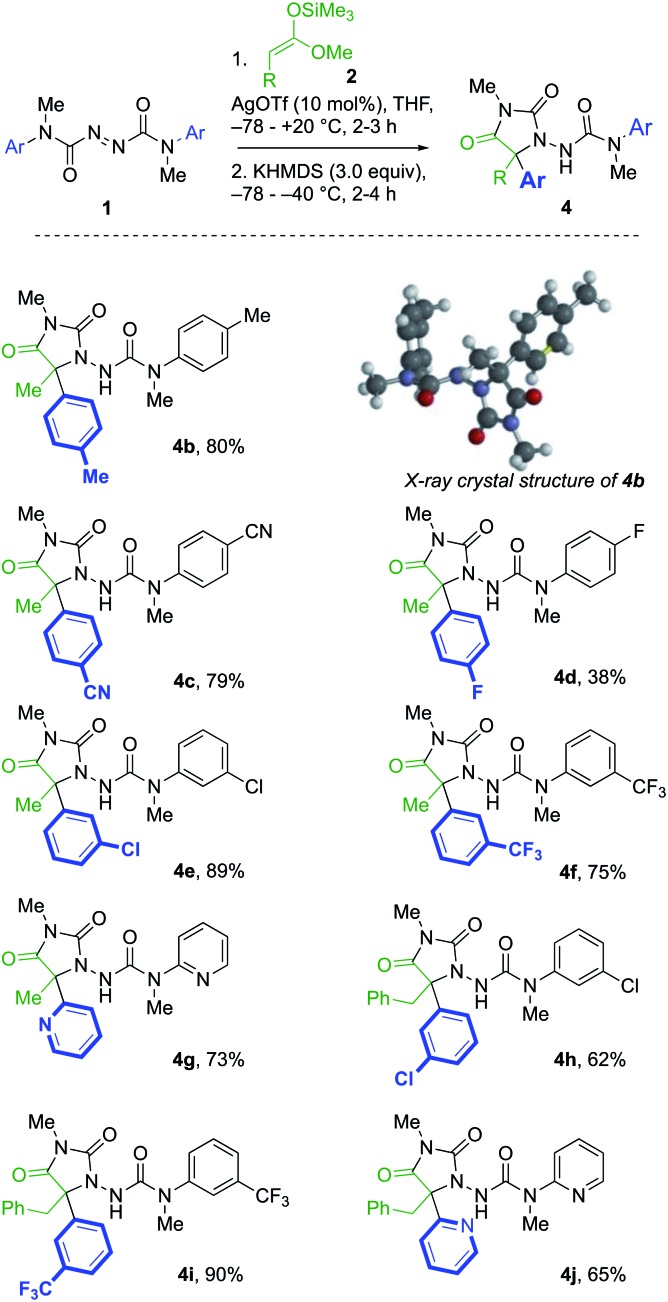
Hydantoin formation from symmetrical azodicarboxamides.

A further series of azodicarboxamides **1b–g** were made, and likewise treated with silyl ketene acetals **2a** and **2b** (Scheme 2). Hydantoin products **4b–f** were formed successfully bearing electron donating, electron withdrawing groups, and the reaction was successful even with the pyridyl substituted **1g**. Additionally, the structure of the *p*-tolyl derivative **4b** was confirmed by X-ray crystallography.^23^ The tandem reaction was also successful with the more hindered silyl ketene acetal **2b** derived from 3-phenylpropionic acids, generating in one pot hydantoins** 4h–j** in good yields.

The products **4** all contain a pendent *N*-aryl urea function derived from the second aryl substituent of the symmetrical starting material, and a greater atom economy would be achieved if an alternative unsymmetrical, mono-*N*-arylated azocarboxamide was used as the aminating agent. By treating *N*-methyl-*N*-tolylcarbamoyl chloride with *t*-butyl carbazate, and oxidising the product hydrazide with NBS (see ESI†), we were able to form the azocarboxamides **5**. Treating silyl ketene acetal **2a** with this compound in the presence of 10 mol% AgOTf in CH_2_Cl_2_ gave the product **6** in 76% yield, accompanied by less than 5% of the alternative regioisomer (Table 2, entry 1). In THF, the yield of **6** increased to 85% and the amination was fully regioselective (entry 2).

**Table 2:** Optimising the use of unsymmetrical aminating agents^*a*^

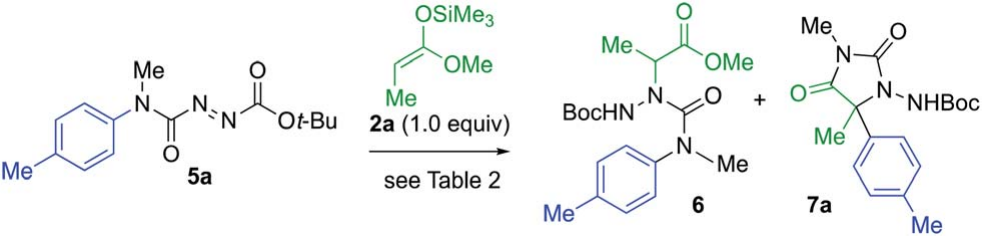			
Entry	Reaction conditions	Yield^*b*^ (%)	
		**6**	**7a**
1	10 mol% AgOTf, CH_2_Cl_2_, –78 → +20 °C, 2 h	76^*c*^	—
2	10 mol% AgOTf, THF, –78 → +20 °C, 2 h	85	—
3	(1) 10 mol% AgOTf, THF, –78 → +20 °C, 2 h	40	20
	(2) KHMDS (2.0 equiv.), –78 → –40 °C, 2 h		
4	(1) 10 mol% AgOTf, THF, –78 → +20 °C, 2 h	0	50
	(2) KHMDS (3.0 equiv.), –78 → –40 °C, 3 h		
5	(1) 10 mol% AgOTf, THF, –78 → +20 °C, 2 h	0	72
	(2) KHMDS (3.0 equiv.), –78 → –20 °C, 2 h		

^*a*^Reactions performed using 0.36 mmol of **4a**, 0.36 mmol of **2a** in 3.6 ml solvent.

^*b*^Isolated yield.

^*c*^Similar results obtained with 15 or 20 mol% catalyst.

When KHMDS was added directly to the crude reaction mixture containing the amination product, arylation and cyclisation to the *N*-Boc-protected aminohydantoin **7a** (entries 3–5) took place, in parallel with the results seen using the symmetrical aminating agent **1a**. With 2.0 equiv. of KHMDS, warming the reaction to –40 °C for 2 h, hydantoin product **7a** was formed in 20% yield (entry 3), increasing to 72% yield on warming to –20 °C (entry 5). Other unsymmetrical aminating agents were also explored, including *N*-benzoyl, *N-tert*-butyl-carboxamido and *N*-methyl-*N-tert*-butyl carboxamido substituted azo compounds. Although intermediate aminated products corresponding to **6** were obtained, treatment with the base led only to decomposition.

**Scheme 3:**
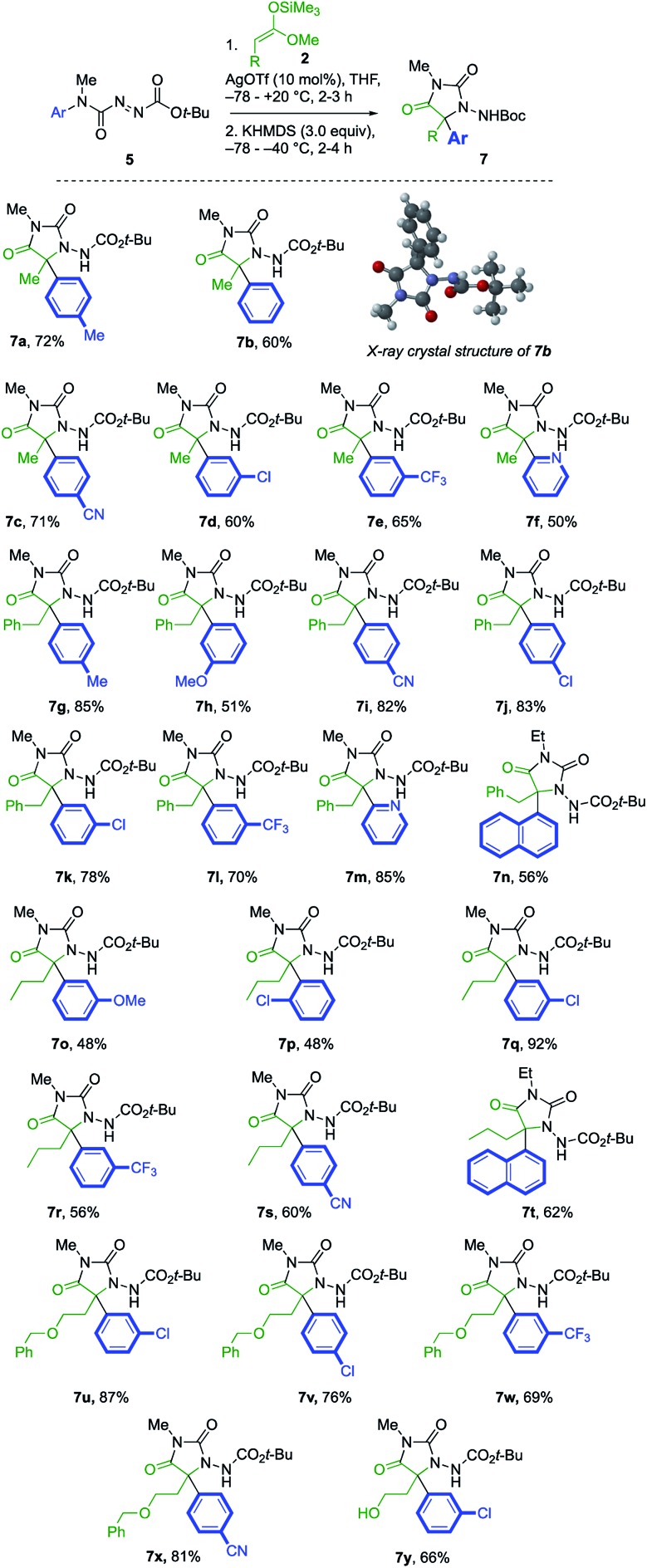
A general, connective synthesis of protected *N*-aminohydantoins.

Under these optimised reaction conditions, the generality of the reaction was explored with various azocarboxamides **5** and silyl ketene acetals **2** (Scheme 3). The α-amination of **2a** with a range of azocarboxamides **5** was fully regioselective in all cases. With a simple phenyl ring, the product **7b** was formed in 60% yield and its structure was confirmed by X-ray crystal structure analysis.^23^ Electron withdrawing groups *p*-CN, *m*-Cl, and *m*-CF_3_ were well tolerated, giving the products **7c–7e** in 65–71% yields. 2-Pyridyl azocarboxamide **5f** likewise performed well and gave hydantoin **7f** in 50% yield.

A wider range of alternative silyl ketene acetal partners were explored. 3-Phenylpropionate-derived **2b** provided the hydantoins **7g–l** by migration of either electron-donating or electron-withdrawing rings in 51–85% yields. With a heteroaryl migrating group, the 2-pyridyl azocarboxamide **5f** provided the desired product **7m** in 85% yield. The more hindered 1-naphthyl-substituted azocarboxamide **5i** formed **7n** in 56% yield, and the migration of other aromatic groups substituted at the ortho position, such as the 2-chlorophenyl group of **7p**, also gave reduced yields.

The pentanoate-derived precursor **2c** likewise yielded the hydantoins** 7q–t** in the presence of electron-donating, electron-withdrawing and bulky groups, as did the benzyloxy substituted silyl ketene acetal **2d**, giving the functionalised hydantoins **7u–x** in good to excellent yields. Deprotection of **7u** using 10% Pearlman's catalyst in MeOH^24^ gave 5-hydroxyethyl substituted **7y**.

**Scheme 4:**
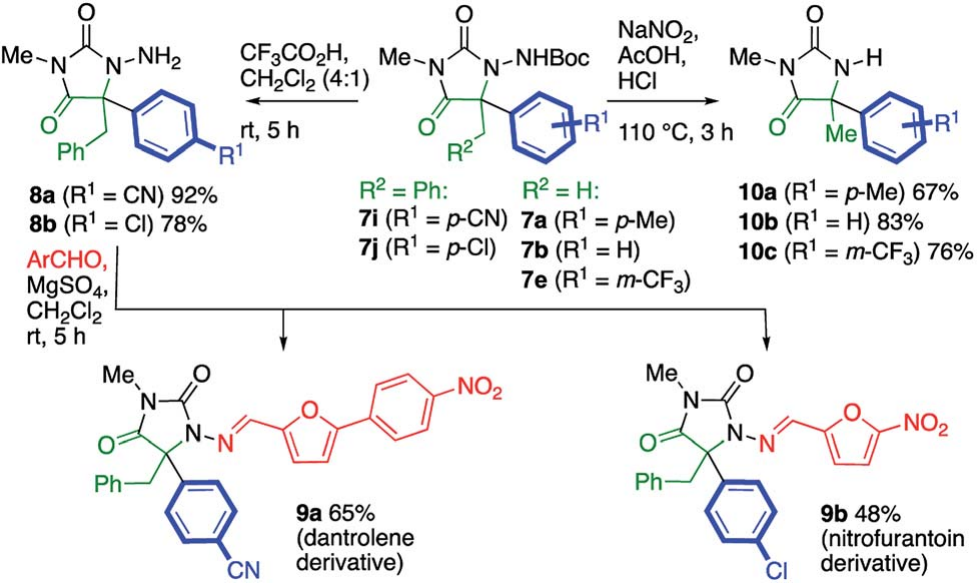
Analogues of bioactive compounds containing the hydantoin motif.

The potential utility of the products was demonstrated by several transformations of the products **7**, as illustrated in Scheme 4. Removal of the Boc protecting groups from **7i** and **7j** proceeded cleanly with 20% trifluoroacetic acid in CH_2_Cl_2_. Condensation of the product *N*-aminohydantoins **8a** and **8b** with appropriate aromatic aldehydes yielded alkylated analogues of the muscle relaxant dantrolene (**9a**) and the antibacterial drug nitrofurantoin (**9b**). Alternatively, the N–N bond of the product could be cleaved to reveal the parent hydantoins **10**. Several methods were screened for this transformation, and we found that treatment of a selection of products **7** with sodium nitrite in 3 : 1 acetic acid/1 M HCl at 110 °C (ref. 25) gave the hydantoins **10a–c** in good yield.

**Scheme 5:**
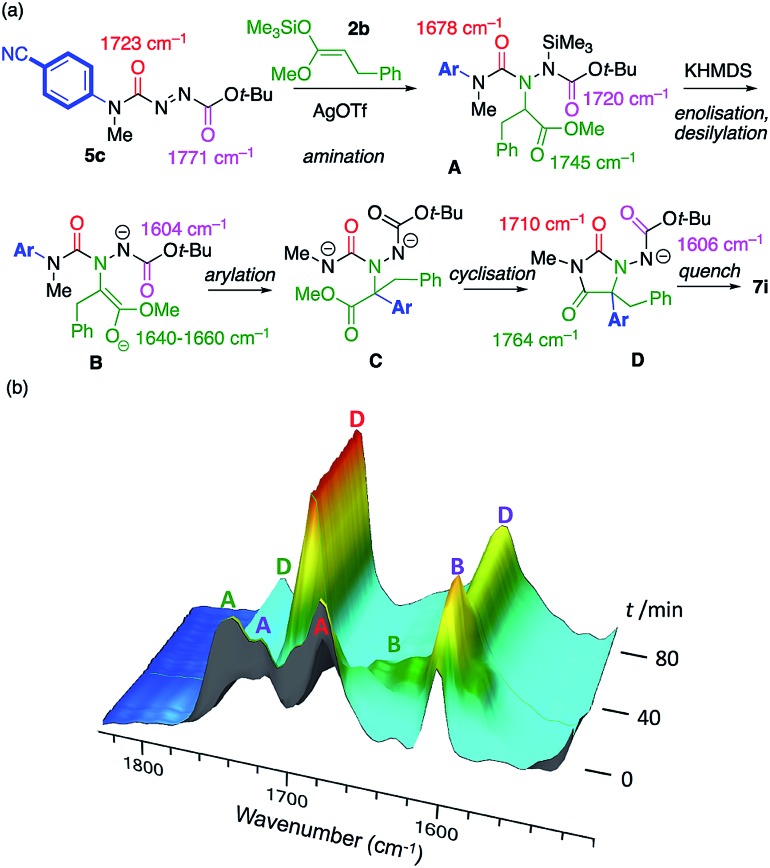
(a) Proposed mechanism of the reaction; (b) Intermediates identified by *in situ* infra-red spectroscopy.

The course of the reaction between model substrates **5c** and **2b** was studied by *in situ* infra-red spectroscopy^26^,16a (see ESI† for full details) and our proposed mechanism for the amination/arylation cascade is presented in Scheme 5a. Silver-catalysed regioselective addition of silyl ketene acetal **2b** to the azocarboxamide **5c** leads to the silylated addition product **A**, consistent with the development of C=O stretching absorptions at 1745 cm^–1^ (ester), 1720 cm^–1^ (carbamate) and 1678 cm^–1^ (urea). Scheme 5b follows the course of the reaction after addition of KHMDS. **A** transforms initially into an intermediate which we assign as the enolate **B**, consistent with the disappearance of the ester C=O stretch at 1745 cm^–1^, and the appearance of peaks we assign to the enolate function at 1640–1660 cm^–1^ plus a peak at 1604 cm^–1^ corresponding to the anionic carbamate. The enolate evolves to a species that has C=O stretching absorptions at 1764 cm^–1^ and 1710 cm^–1^, typical of a hydantoin,16*a* and retains an anionic carbamate peak at 1606 cm^–1^. We assign these peaks to species **D**, the conjugate base of the ultimate product **7i**. The rearrangement of **B** to **D** presumably passes through an undetectable transient intermediate **C** that cyclises rapidly to **D**. Evidence from related reactions suggests that the formation of the new C–C bond and breakage of the old C–N bond during the formation of the proposed intermediate **C** are to some extent concerted.16e,^27^

## Conclusions

In conclusion, 5,5-disubstituted hydantoins may be formed by a tandem amination-intramolecular arylation sequence of silyl ketene acetals. The amination entails silver-catalysed regioselective addition to the N=N bond of a new class of unsymmetrical azocarboxamides, and the arylation takes place by base-promoted intramolecular N to C migration within the *N*′-aryl urea linkage that results from the amination step. The hydantoin then forms directly from the product of enolate arylation. *In situ* infra-red spectroscopy reveals four successive species on the reaction pathway from the amination step to the hydantoin ring closure. The one-pot protocol allowed the connective synthesis of a range of 5,5-disubstituted hydantoins bearing electronically diverse aryl substituents, compounds which have potential applications in the construction of biologically active molecules.

## Conflicts of interest

There are no conflicts of interest to declare.

## Supplementary Material

Supplementary information

Crystal structure data
