# Supplementary material for: Connective synthesis of 5,5-disubstituted hydantoins by tandem α-amination and α-arylation of silyl ketene acetals
Source: Chem Sci. 2019 Feb 6;10(11):3408–12. doi: 10.1039/c8sc05263h (PMC6429467; doi:10.1039/c8sc05263h)

Electronic Supplementary Material (ESI) for Chemical Science.
This journal is © The Royal Society of Chemistry 2019

Direct Connective Synthesis of 5,5-Disubstituted Hydantoins by Tandem α-Amination and α-Arylation of Silyl Enol Ethers

Rakesh K. Saunthwal, Matthew T. Cornall, John W. Ward* and Jonathan Clayden*

School of Chemistry, University of Bristol, Cantock's Close, Bristol, BS8 1TS (UK)

E-mail: john.ward@liverpool.ac.uk; j.clayden@bristol.ac.uk

Contents

1 Experimental

1.1 General Directions	2-2
1.2 Analytical Directions	2-3
1.3 Literature Known Starting Materials	3-3
1.4 General scheme for Starting materials preparation	3-3
1.5 General Procedure 1: Carbamoyl Chloride Synthesis from the secondary amines and analytical data	4-5
1.6 General Procedure 2: Synthesis of Symmetrical arylhydrazine-1,2-dicarboxamide	5-16
1.7 General Procedure 3: Synthesis of Symmetrical α, β unsaturated azocarbonamides	6-16
1.8 General Procedure 4: Carbamoyl Chloride and tert-Butyl carbamate coupling	16-29
1.9 General Procedure 5: Synthesis of unsymmetrical α, β unsaturated azocarbonamides	16-29
1.10 General Procedure 6: Synthesis of Silyl enol ether	29-33
1.11 X-ray crystallography	33-34
1.12 ReactIR studies	35-35
1.13 Procedures and Analytical data of Urea substituted hydantoins (4)	36-46
1.14 General Procedure 7: Synthesis of Boc-hyantoins (7)	46-46
1.15 Procedure and Analytical data of hydantoins	47-69
1.16 General procedure 8: Benzyl deprotection	69-69
1.17 General Procedure 9: Boc-group deprotection:	69-71
1.18 General Procedure 9: Synthesis of bioactive compound analogues	71-73
1.19 ^{1}H and ^{13}C NMR Spectra	74-243

1 Experimental

1.1 General Directions: Reactions requiring anhydrous conditions (where specified) were executed under dry nitrogen or argon atmospheres in glassware that was dried using either a combination of vacuum and heat-gun, oven, or flame drying. Reaction mixtures were stirred magnetically. Air- and moisture-sensitive liquids and solutions were transferred via syringe into the reaction vessels through rubber septa. Reactions run in a microwave oven were completed on a Biotage Initiator+. All reagents were purchased (unless specified) at highest commercial quality and used as received. Non-anhydrous solvents were purchased (unless specified) at the highest commercial quality and used as received. Anhydrous CH_2Cl_2 and THF were obtained from the University of Bristol's dry solvent system and were purified by filtration over a column of activated alumina. All temperatures described below −10 °C were achieved using a Julabo cryostat

1.2 Analytical Directions

Rf: TLC was performed on aluminium backed silica plates (0.2 mm, 60 F254) which were developed using standard visualising agents: UV fluorescence (254 & 366 nm), phosphomolybdic acid / Δ, vanillin / Δ, potassium permanganate / Δ and Seebach / Δ. Chromatography: Flash chromatography was performed on an automated Biotage Isolera TM Spectra Four using gradient elution on pre-packed silica gel Biotage® SNAP Ultra columns.

MP: Melting points were measured on a Kofler hotstage melting point apparatus and are uncorrected.

IR: IR spectra were recorded on neat compounds using a Perkin Elmer (Spectrum One) FT-IR spectrometer (ATR sampling accessory). Only strong and selected absorbance's (νmax expressed in cm^{-1}) are reported.

^{1}H NMR: Spectra were recorded on Jeol ECS (400 MHz) or Bruker NMR (400 MHz or 500 MHz) instruments. Chemical shifts (δ H) are quoted in parts per million (ppm) was

used. Spin-spin coupling constants (*J*) are reported in Hertz (Hz). 2D NMR experiments HSQC and HMBC where necessary. Spin-spin coupling constants (J) are reported in Hertz (Hz)

^{13}C NMR: Spectra were recorded on Jeol ECS (101 MHz) or Bruker NMR (101 MHz or 125 MHz) instruments. Chemical shifts (δ C) are quoted in parts per million (ppm) and referenced to the appropriate solvent peak(s). Spin-spin coupling constants (*J*) are reported in Hertz (Hz).

HRMS: High resolution mass spectra were recorded on a Bruker Daltronics MicrOTOF 2 mass spectrometer (ESI).

1.3 Literature Known Starting Materials[1]

Starting materials carbamoyl chlorides were prepared according to the procedures reported, Spectroscopic data for the materials prepared as described above were consistent with those reported in the literature.

1.4 General scheme for Starting materials preparation

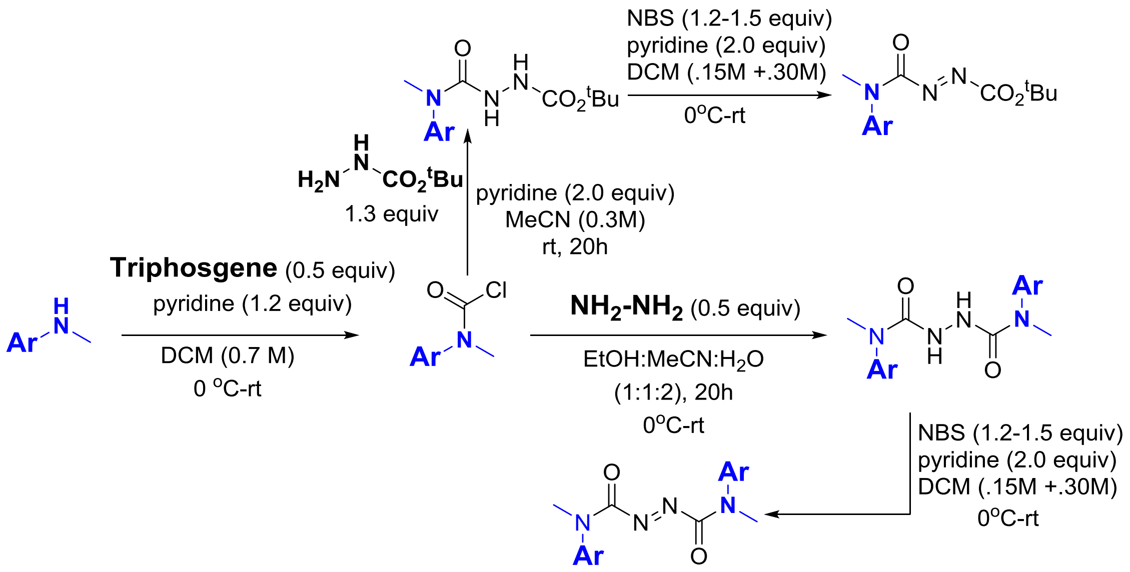

[1] (a) F. Fernández-Nieto, J. Mas Roselló, S. Lenoir, S. Hardy, J. Clayden, *Org. Lett.* **2015**, *17*, 3838-3841 (b) R. C. Atkinson, F. Fernández-Nieto, J. Mas Roselló, J. Clayden, *Angew. Chemie Int. Ed.* **2015**, *54*, 8961-8965 (c) R. C. Atkinson, D. J. Leonard, J. Maury, D. Castagnolo, N. Volz, J. Clayden, *Chem. Commun.* **2013**, *49*, 9734-9736. (d) J. Maury, J. Clayden, *J. Org. Chem.* **2015**, *80*, 10757-10768

1.5 General Procedure 1: Carbamoyl Chloride Synthesis from the secondary Amines

A flame-dried two-necked round bottom flask was allowed to cool to RT under vacuum. Triphosgene (0.46 equiv) was then added and the reaction vessel subsequently nitrogen/vacuum cycled three times. Anhydrous CH_2Cl_2 (0.7 M) was then added under an atmosphere of nitrogen and the reaction was cooled to 0 °C. Pyridine (1 equiv was then added dropwise and the reaction allowed to stir for 5 min at 0 °C. Aniline (1 equiv) was then added to the reaction mixture dropwise and allowed to stir for 5 min. The reaction mixture was then allowed to warm to RT until consumption of the aniline was observed by TLC. The reaction mixture was quenched with HCl (1 M, 3 × 20 mL) and extracted with CH_2Cl_2 (3 x 20 mL). The combined organic phases were washed with sat. $NaHCO_3$ (20 mL), dried over $MgSO_4$, filtered and the subsequent filtrate concentrated under vacuum to yield the crude carbamoyl chloride. Purification through a pad of silica eluting with 10% EtOAc/Petrol (200 mL) gave the desired carbamoyl chloride, which could be used directly in the next step or stored in the freezer at −20 °C until required. (N.B. prior to purification the carbamoyl chlorides often have vibrant colours. Some of the carbamoyl chlorides solidify on standing at −20 °C).

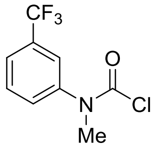

***V*max /cm^{-1}(neat):** 2987, 1732, 1322, 1120, 698

Methyl(3-(trifluoromethyl)phenyl)carbamic chloride (S1f) **^{1}H NMR** (400 MHz; $CDCl_3$) δ 7.67–7.57 (m, 3H), 7.51–7.49 (m, 1H), 3.45 (s, 3H); **^{13}C NMR** (101 MHz; $CDCl_3$) δ 148.8, 143.5, 132.3 (q, $J_{C\text{-}F}$ = 33.0 Hz), 131.0 (br), 130.3, 125.3 (br), 124.6 (br), 123.4 (q, $J_{C\text{-}F}$ = 272.6.0 Hz), 40.3; **HRMS** (ESI) calcd for [$C_9H_7ClF_3NONa$] requires [M + Na]+ 260.0066, found 260.0059

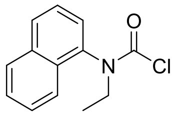

V_{max} **/cm^{-1}(neat):** 2976, 1728, 1401, 1233, 773

Methyl(naphthalen-1-yl)carbamic chloride (S5i) (colourless Semi-solid) **^{1}H NMR** (400 MHz; $CDCl_3$) δ 7.92 (dd, J = 7.9, 4.0 Hz, 2H), 7.85 (d, J = 8.7 Hz, 1H), 7.64 – 7.47 (m, 3H), 7.38 (d, J = 7.3 Hz, 1H), 4.17 (dq, J = 14.2, 7.2 Hz, 1H), 3.59 (dt, J = 13.6, 6.9 Hz, 1H), 1.26 (t, J = 7.2 Hz, 3H); **^{13}C NMR** (101 MHz; $CDCl_3$) δ 149.8, 137.8, 134.7, 130.2, 129.4, 128.7, 127.6, 126.8, 125.5, 122.3, 48.07, 13.2; **HRMS** (ESI) calcd for [$C_{13}H_{12}C1NONa$] requires [M + Na]+ 256.0505, found 256.0494

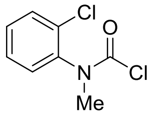

V_{max} **/cm^{-1}(neat):** 2945, 1735, 1481, 1351, 1267, 1059,

(2-Chlorophenyl)(methyl)carbamic chloride (S5j), ^{1}H NMR (400 MHz; $CDCl_3$) δ 7.51 – 7.46 (m, 1H), 7.36 – 7.29 (m, 3H), 3.30 (s, 3H). **^{13}C NMR** (101 MHz; $CDCl_3$) δ 149.3, 140.4, 133.0, 130.6, 130.3, 129.8, 128.2, 38.9. **HRMS** (ESI) calcd for [$C_8H_7C1_2NONa$] requires [M + Na]+ 225.9802, found 225.9797

1.6 General Procedure 2: Synthesis of Symmetrical arylhydrazine-1,2-dicarboxamide:

A solution of arylcarbamic chloride (1.0 equiv) in MeCN (1.5 M) was added dropwise to a solution of hydrazine monohydrate (1 equiv) in EtOH (1.5 M) at 0 °C. After 10 minutes a solution of arylcarbamic chloride (1.0 equiv) in MeCN (1.5 M) and a solution of Na_2CO_3 (1 equiv) in H_2O (0.75 M) were added to the reaction flask simultaneously. The resulting solution was stirred at room temperature for 20 h, giving a precipitate. The reaction was concentrated under reduced pressure, re-dissolved in DCM, filtered and concentrated under reduced pressure to give a crude residue. Purification by flash silica chromatography afforded the title compound.

1.7 General Procedure 3: Synthesis of Symmetrical α, β unsaturated azocarbonamides

A solution of *N*-bromosuccinimide (1.2-1.5 equiv) in DCM (0.15 M) was added dropwise to a solution of pyridine (2 equiv) and arylhydrazine-1,2-dicarboxamide **S1** in DCM (11 mL, 0.30 M) at 0 ºC. The reaction was stirred at RT for 3 h, before being quenched with sat. aq. $NaHCO_3$ (40 mL) and the aqueous layer was extracted with DCM (3 × 20 mL). The combined organics were washed with brine (1 × 20 mL), dried over $MgSO_4$ and concentrated under reduced pressure to give a crude residue. Purification by flash silica chromatography afforded the s an orange solid.

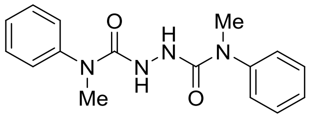

N^1,N^2-dimethyl-N^1,N^2-diphenylhydrazine-1,2-dicarboxamide (**S1a**). A solution of methyl(phenyl)carbamic chloride (750 mg, 4.4 mmol) in MeCN (2.9 mL, 1.5 M) was added dropwise to a solution of hydrazine monohydrate (142 mg, 4.4 mmol, 1 equiv) in EtOH (2.9 mL, 1.5 M) at 0 ºC. After 10 minutes a solution of methyl(phenyl)carbamic chloride (750 mg, 4.4 mmol) in MeCN (2.9 mL, 1.5 M) and a solution of Na_2CO_3 (465 mg 4.4 mmol) in H_2O (5.8 mL, 0.75 M) were added to the reaction flask simultaneously. The resulting solution was stirred at room temperature for 20 h, giving a precipitate. The reaction was concentrated under reduced pressure, re-dissolved in DCM, filtered and concentrated under reduced pressure to give a crude residue. Purification by flash silica chromatography (5% MeOH/DCM) afforded the title compound (1013 mg, 75%) as a white solid.

MP: 145-147 ºC

***V*$_{max}$ /cm^{-1}(neat):** 3287, 2922, 1662, 1512, 1476, 1335, 1138, 824

^{1}H NMR (400 MHz; $CDCl_3$) δ 7.42 (d, *J* = 8.3 Hz, 2H), 7.40 – 7.35 (m, 6H), 7.29 (tt, *J* = 6.1, 1.6 Hz, 2H), 6.06 (s, 2H), 3.27 (s, 6H); **^{13}C NMR** (101 MHz; $CDCl_3$) δ ^{13}C NMR (101 MHz,

CDCl3) δ 159.2, 142.3, 130.0, 127.6, 127.2, 37.4; **HRMS m/z** (ESI$^+$) [$C_{16}H_{18}N_4O_2Na$] requires [M + Na]+: 321.1327; found: 321.1320

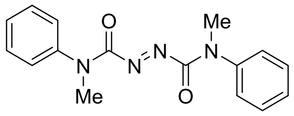

(*E*)-N^1,N^2-dimethyl-N^1,N^2-diphenyldiazene-1,2-dicarboxamide (1a). A solution of *N*-bromosuccinimide (356 mg, 2.23 mmol, 1.2 eq.) in DCM (11 mL, 0.15 M) was added dropwise to a solution of pyridine (0.30 mL, 3.3 mmol, 2 eq.) and N^1,N^2-dimethyl-N^1,N^2-diphenylhydrazine-1,2-dicarboxamide **S1a** (500 mg, 1.67 mmol) in DCM (5.5 mL, 0.30 M) at 0 ^{o}C. The reaction was stirred at RT for 3 h, before being quenched with sat. aq. $NaHCO_3$ (40 mL) and the aqueous layer was extracted with DCM (3 × 20 mL). The combined organics were washed with brine (1 × 20 mL), dried over $MgSO_4$ and concentrated under reduced pressure to give a crude residue. Purification by flash silica chromatography (10% EtOAc/Hexane) afforded *the title compound* (372 mg, 75%) as an orange solid.

MP: 171-173 ^{o}C

***V*$_{max}$ /cm^{-1}(neat):** 2921, 1710, 1705, 1574, 1362,

^{1}H NMR (400 MHz, CDCl3) δ 7.39 – 7.24 (m, 10H), 3.39 (s, 6H); **^{13}C NMR** (100 MHz, CDCl3) δ ^{13}C NMR (101 MHz, CHLOROFORM-*D*) δ 160.9, 140.6, 129.4, 128.0, 127.1, 38.5; HRMS (ESI) calcd for [$C_{16}H_{16}N_4O_2Na$] requires [M + Na]+ 319.1171, found 319.1164

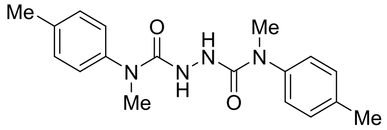

N^1, N^2-dimethyl-N^1, N^2-di-*p*-tolylhydrazine-1,2-dicarboxamide (**S1b**). A solution of *N*-methyl(*p*-tolyl)carbamic chloride (750 mg, 4.09 mmol) in MeCN (2.73 mL, 1.5 M) was added dropwise to a solution of hydrazine monohydrate (131 mg, 4.09 mmol, 1 equiv) in EtOH (2.73 mL, 1.5 M) at 0 ^{o}C. After 10 minutes a solution of *N*-methyl(*p*-tolyl)carbamic chloride (750 mg, 4.09 mmol) in MeCN (2.73 mL, 1.5 M) and a solution of Na_2CO_3 (430 mg 4.09 mmol) in H_2O (5.46 mL, 0.75 M) were added to the reaction flask simultaneously. The resulting solution

was stirred at room temperature for 20 h, giving a precipitate. The reaction was concentrated under reduced pressure, re-dissolved in DCM, filtered and concentrated under reduced pressure to give a crude residue. Purification by flash silica chromatography (5% MeOH/DCM) afforded the title compound (1023 mg, 76%) as a white solid.

MP: 150-152 °C

***V*max /cm^{-1}(neat):** 3287, 2922, 1662, 1512, 1476, 1335, 1138, 824

^{1}H NMR (400 MHz; $CDCl_3$) δ 7.25 – 7.22 (m, 4H), 7.20 – 7.17 (m, 4H), 6.04 (br, s, 2H), 3.23 (s, 6H), 2.23 (s, 6H); **^{13}C NMR** (101 MHz; $CDCl_3$) δ 158.0, 139.6, 137.9, 130.8, 127.1, 37.7, 21.2; **HRMS m/z** (ESI$^+$) [$C_{18}H_{22}N_4O_2Na$] requires [M + Na]+: 349.1640; found: 349.1644

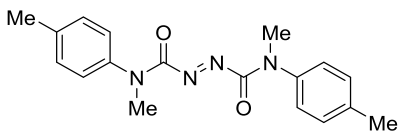

(*E*)-N^1,N^2-dimethyl-N^1,N^2-di-*p*-tolyldiazene-1,2-dicarboxamide (1b); A solution of *N*-bromosuccinimide (651 mg, 3.68 mmol, 1.2 equiv) in DCM (20 mL, 0.15 M) was added dropwise to a solution of pyridine (0.557 mL, 6.12 mmol, 2 equiv) and N^1,N^2-dimethyl-N^1,N^2-di-*p*-tolylhydrazine-1,2-dicarboxamide **S1b** (1g, 3.06 mmol) in DCM (10 mL, 0.30 M) at 0 °C. The reaction was stirred at RT for 3 h, before being quenched with sat. aq. $NaHCO_3$ (40 mL) and the aqueous layer was extracted with DCM (3 × 20 mL). The combined organics were washed with brine (1 × 20 mL), dried over $MgSO_4$ and concentrated under reduced pressure to give a crude residue. Purification by flash silica chromatography (30% EtOAc/Hexane) afforded the title compound (876 mg, 88%) as orange needles.

MP: 181-183 °C

***V*max /cm^{-1}(neat):** 2922, 1709, 1572, 1365, 1125, 820, 555

^{1}H NMR (400 MHz, $CDCl_3$) δ 7.12 (d, *J* = 8.0 Hz, 4H), 6.86 (d, *J* = 8.0 Hz, 4H), 3.37 (s, 6H) 2.34 (s, 6H); **^{13}C NMR** (100 MHz, $CDCl_3$) δ 161.1, 138.1, 137.9, 130.0, 126.9, 38.6, 21.2; HRMS (ESI) calcd for [$C_{18}H_{20}N_4O_2$ Na] requires [M + Na]+ 347.1484, found 347.1477

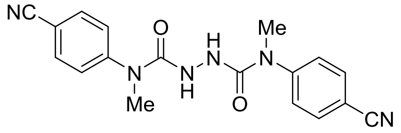

N[1],N[2]-bis(4-cyanophenyl)-N[1],N[2]-dimethylhydrazine-1,2-dicarboxamide (**S1c**). A solution of (4-cyanophenyl)(methyl)carbamic chloride (400 mg, 2.06 mmol) in MeCN (1.37 mL, 1.5 M) was added dropwise to a solution of hydrazine monohydrate (65 mg, 2.03 mmol, 1 equiv) in EtOH (1.37 mL, 1.5 M) at 0 °C. After 10 minutes a solution of (4-cyanophenyl)(methyl)carbamic chloride (400 mg, 2.06 mmol) in MeCN (1.37 mL, 1.5 M) and a solution of Na_2CO_3 (259 mg 2.47 mmol) in H_2O (2.74 mL, 0.75 M) were added to the reaction flask simultaneously. The resulting solution was stirred at room temperature for 20 h, giving a precipitate. The reaction was concentrated under reduced pressure, re-dissolved in DCM, filtered and concentrated under reduced pressure to give a crude residue. Purification by flash silica chromatography (5% MeOH/DCM) afforded the title compound (509 mg, 71%) as a white solid.

MP: 200-202 °C

$\boldsymbol{V_{max}}$ **/cm^{-1}(neat):** 3320, 2970, 2227, 1665, 1603, 1505, 1333, 1110, 754

^{1}H NMR (400 MHz; $CDCl_3$) δ 7.76 – 7.73 (m, 4H), 7.59 – 7.56 (m, 4H), 6.33 (br, s, 2H), 3.36 (s, 6H); **^{13}C NMR** (101 MHz; $CDCl_3$) δ 156.9, 146.5, 133.9, 127.0, 118.0, 110.8, 37.4; **HRMS** m/z (ESI$^+$) [$C_{18}H_{16}N_6O_2Na$] requires [M + Na]+: 371.1232; found: 371.1243

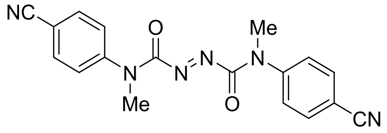

(*E*)-N[1],N[2]-bis(4-cyanophenyl)-N[1],N[2]-dimethyldiazene-1,2-dicarboxamide (1c); A solution of *N*-bromosuccinimide (305 mg, 1.7 mmol, 1.2 equiv) in DCM (9.4 mL, 0.15 M) was added dropwise to a solution of pyridine (0.260 mL, 2.8 mmol, 2 equiv) and N[1],N[2]-bis(4-cyanophenyl)-N[1],N[2]-dimethylhydrazine-1,2-dicarboxamide **S1c** (500, 1.4 mmol) in DCM (4.7 mL, 0.30 M) at 0 °C. The reaction was stirred at RT for 3 h, before being quenched with sat.

aq. $NaHCO_3$ (40 mL) and the aqueous layer was extracted with DCM (3 × 20 mL). The combined organics were washed with brine (1 × 20 mL), dried over $MgSO_4$ and concentrated under reduced pressure to give a crude residue. Purification by flash silica chromatography (30% EtOAc/Hexane) afforded the *title compound* (420 mg, 84%) as orange needles.

MP: 204-206 °C

***V*max /cm⁻¹(neat):** 3398, 2312, 1720, 1658, 1023, 995

^{1}H NMR (500 MHz, $CDCl_3$) δ 7.66 (d, *J* = 8.6 Hz, 4H), 7.25 – 7.15 (m, 4H), 3.46 (s, 6H); **^{13}C NMR** (126 MHz, $CDCl_3$)160.0, 144.3, 133.3, 127.4, 117.7, 111.8, 29.7; **HRMS** (ESI) calcd for [$C_{18}H_{14}N_6O_2Na$] requires [M + Na]+ 369.1076, found 369.1070

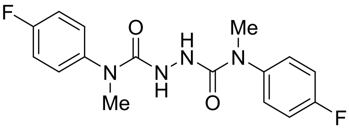

N^1,N^2-bis(4-fluorophenyl)-N^1,N^2-dimethylhydrazine-1,2-dicarboxamide (**S1d**); A solution of (4-fluorophenyl)(methyl)carbamic chloride (500 mg, 2.67 mmol) in MeCN (1.78 mL, 1.5 M) was added dropwise to a solution of hydrazine monohydrate (85 mg, 2.65 mmol, 1 equiv) in EtOH (1.78 mL, 1.5 M) at 0 °C. After 10 minutes a solution of (4-fluorophenyl) (methyl)carbamic chloride (500 mg, 2.67 mmol) in MeCN (1.5 mL, 1.5 M) and a solution of Na_2CO_3 (245 mg 2.3 mmol) in H_2O (3.5 mL, 0.75 M) were added to the reaction flask simultaneously. The resulting solution was stirred at room temperature for 20 h, giving a precipitate. The reaction was concentrated under reduced pressure, re-dissolved in DCM, filtered and concentrated under reduced pressure to give a crude residue. Purification by flash silica chromatography (05% MeOH/DCM) afforded the title compound (630 mg, 70%) as a white solid.

MP: 100-102 °C

***V*max /cm⁻¹(neat):** 3286, 2967, 1786, 1660, 1505, 1338, 1220, 1140, 842, 752

^{1}H NMR (400 MHz; $CDCl_3$) δ 7.36 – 7.33 (m, 4H), 7.11 – 7.07 (m, 4H), 6.00 (br, s, 2H), 3.23 (s, 6H); **^{13}C NMR** (101 MHz; $CDCl_3$) δ 161.0 (d, $J_{C\text{-}F}$ = 249.0 Hz), 157.9, 138.1, 129.2 (d, $J_{C\text{-}F}$ = 8.0 Hz), 117.2 (d, $J_{C\text{-}F}$ = 23.2 Hz), 37.9; HRMS m/z (ESI^+) [$C_{16}H_{16}F_2N_4O_2Na$] requires $[M+Na]^+$ 357.1139; found: 357.1136

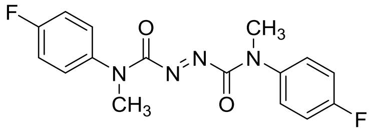

(*E*)-N^1,N^2-bis(4-fluorophenyl)-N^1,N^2-dimethyldiazene-1,2-dicarboxamide (1d); A solution of *N*-bromosuccinimide (381 mg, 2.15 mmol, 1.2 equiv) in DCM (11.9 mL, 0.15 M) was added dropwise to a solution of pyridine (0.32 mL, 3.5 mmol, 2 equiv) and N^1,N^2-bis(4-fluorophenyl)-N^1,N^2-dimethylhydrazine-1,2-dicarboxamide **S1d** (250 mg, 0.89 mmol) in DCM (5.56 mL, 0.30 M) at 0 ^{o}C. The reaction was stirred at RT for 5 h, before being quenched with sat. aq. $NaHCO_3$ (20 mL) and the aqueous layer was extracted with DCM (3 × 20 mL). The combined organics were washed with brine (1 × 20 mL), dried over $MgSO_4$ and concentrated under reduced pressure to give a crude residue. Purification by flash silica chromatography (30% EtOAc/Hexane) afforded the title compound (550 mg, 92%) as orange needles.

MP: 161-163 ^{o}C

***V_{max}* /cm^{-1}(neat):** 2986, 1713, 1507, 1370, 1222, 842

^{1}H NMR (400 MHz, $CDCl_3$) δ 7.07-6.99 (m, 8H), 3.41 (s, 6H); **^{13}C NMR** (100 MHz, CDCl3) δ 161.2 (d, $J_{C\text{-}F}$ = 219.0 Hz), 160.5, 136.5, 128.9 (d, $J_{C\text{-}F}$ = 9.0 Hz), 116.4 (d, $J_{C\text{-}F}$ = 23.2.0 Hz), 38.9; HRMS (ESI) calcd for [$C_{16}H_{14}F_2N_4O_2Na$] requires [M + Na]+ 355.0983, found 355.0985

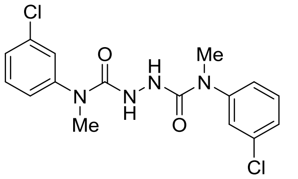

N^1,N^2-bis(3-chlorophenyl)-N^1,N^2-dimethylhydrazine-1,2-dicarboxamide (**S1e**); A solution of (3-chlorophenyl)(methyl)carbamic chloride (400 mg, 1.98 mmol) in MeCN (1.3 mL, 1.5 M) was added dropwise to a solution of hydrazine monohydrate (63 mg, 1.98 mmol, 1 equiv) in EtOH (1.3 mL, 1.5 M) at 0 °C. After 10 minutes a solution of (4-fluorophenyl)(methyl)carbamic chloride (500 mg, 2.67 mmol) in MeCN (1.3 mL, 1.5 M) and a solution of Na_2CO_3 (207 mg 1.98 mmol) in H_2O (3.0 mL, 0.75 M) were added to the reaction flask simultaneously. The resulting solution was stirred at room temperature for 20 h, giving a precipitate. The reaction was concentrated under reduced pressure, re-dissolved in DCM, filtered and concentrated under reduced pressure to give a crude residue. Purification by flash silica chromatography (05% MeOH/DCM) afforded the title compound (600 mg, 82%) as white needles.

MP: 164-166 °C

***V*max /cm^{-1}(neat):** 3280, 2969, 1660 1591, 1479, 1351

^{1}H NMR (400 MHz; $CDCl_3$) δ 7.35 (dd, *J* = 4.9, 2.9 Hz, 3H), 7.32 – 7.30 (m, 2H), 7.28 (dt, *J* = 3.6, 1.8 Hz, 2H), 7.27 – 7.24 (m, 1H), 6.13 (s, 2H), 3.25 (s, 6H); **^{13}C NMR** (101 MHz; $CDCl_3$) δ 157.41, 143.47, 135.53, 131.15, 128.11, 127.47, 125.41, 37.74; **HRMS m/z** (ESI^+) [$C_{16}H_{16}Cl_2N_4O_2Na$] requires $[M+Na]^+$ 389.0548; found: 389.0548

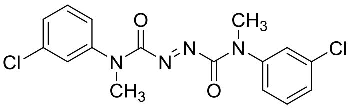

(*E*)-N^1,N^2-bis(3-chlorophenyl)-N^1,N^2-dimethyldiazene-1,2-dicarboxamide(1e); A solution of *N*-bromosuccinimide (402 mg, 2.2 mmol, 1.2 equiv) in DCM (12.6 mL, 0.15 M) was added dropwise to a solution of pyridine (0.34 mL, 3.7 mmol, 2 equiv) and N^1,N^2-bis(3-chlorophenyl)-N^1,N^2-dimethylhydrazine-1,2-dicarboxamide **S1e** (690 mg, 1.89 mmol) in DCM (6.3 mL, 0.30 M) at 0 °C. The reaction was stirred at RT for 5 h, before being quenched with sat. aq. $NaHCO_3$ (20 mL) and the aqueous layer was extracted with DCM (3 × 20 mL). The combined organics were washed with brine (1 × 20 mL), dried over $MgSO_4$ and

concentrated under reduced pressure to give a crude residue. Purification by flash silica chromatography (10% EtOAc/Hexane) afforded the title compound (620 mg, 90%) as orange needles.

MP: 116-118 °C

***V*max /cm-1(neat):** 2987, 1713, 1364, 786, 693

^{1}H NMR (400 MHz, $CDCl_3$) δ 7.28 (d, *J* = 4.0 Hz, 5H), 7.08 (s, 2H), 6.87 (s, 1H), 3.40 (s, 6H); **^{13}C NMR** (100 MHz, CDCl3) δ 160.4, 141.7, 135.0, 130.5, 128.4, 127.1, 125.6, 125.2, 38.6; HRMS (ESI) calcd for [$C_{16}H_{14}Cl_2N_4O_2Na$] requires [M + Na]+ 387.0392, found 387.0389

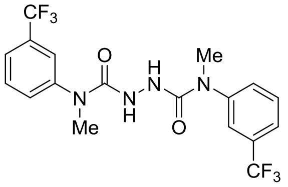

N^1,N^2-dimethyl-N^1,N^2-bis(3-(trifluoromethyl)phenyl)hydrazine-1,2-dicarboxamide(S1f).

A solution of methyl(3-(trifluoromethyl)phenyl)carbamic chloride (400 mg, 1.68 mmol) in MeCN (1.1 mL, 1.5 M) was added dropwise to a solution of hydrazine monohydrate (85 mg, 2.65 mmol, 1 equiv) in EtOH (1.1 mL, 1.5 M) at 0 °C. After 10 minutes a solution of methyl(3-(trifluoromethyl)phenyl)carbamic chloride (400 mg, 1.68 mmol) in MeCN (1.1 mL, 1.5 M) and a solution of Na_2CO_3 (212 mg 2.0 mmol) in H_2O (2.2 mL, 0.75 M) were added to the reaction flask simultaneously. The resulting solution was stirred at room temperature for 20 h, giving a precipitate. The reaction was concentrated under reduced pressure, re-dissolved in DCM, filtered and concentrated under reduced pressure to give a crude residue. Purification by flash silica chromatography (5% MeOH/DCM) afforded the title compound (663 mg, 90%) as white needles.

MP: 112-114 °C

***V*max /cm-1(neat):** 3274, 2968, 1658, 1613, 1593, 1492, 1327, 1120, 701

^{1}H NMR (400 MHz; $CDCl_3$) δ 7.65 – 7.63 (m, 2H), 7.60 – 7.55 (m, 6H), 6.09 (br, s, 1H), 3.31 (s, 6H); **^{13}C NMR** (101 MHz; $CDCl_3$) δ 157.3, 142.9, 132.8, 132.4, 130.8, 130.6, 126.0 (q, J_{C-F} = 272 Hz), 124.5 (q, J_{C-F} = 4.0 Hz, CF_3), 123.9 (d, J_{C-F} = 4.0 Hz, CF_3), 37.7; **HRMS m/z** (ESI^+) [$C_{18}H_{16}F_6N_4O_2Na$] requires[M + Na]+: 457.1075; found: 457.1057

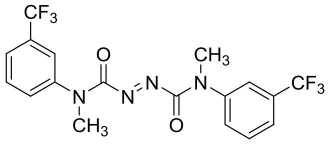

(*E*)-N^1,N^2-dimethyl-N^1,N^2-bis(3-(trifluoromethyl)phenyl)diazene-1,2-dicarboxamide(1f); A solution of *N*-bromosuccinimide (244.7 mg, 1.38 mmol, 1.2 equiv) in DCM (7.6 mL, 0.15 M) was added dropwise to a solution of pyridine (0.20 mL, 2.3 mmol, 2 equiv) and N^1,N^2-dimethyl-N^1,N^2-bis(3-(trifluoromethyl)phenyl)hydrazine-1,2-dicarboxamide **S1f** (500 mg, 1.15 mmol) in DCM 3.8 mL, 0.30 M) at 0 °C. The reaction was stirred at RT for 5 h, before being quenched with sat. aq. $NaHCO_3$ (20 mL) and the aqueous layer was extracted with DCM (3 × 20 mL). The combined organics were washed with brine (1 × 20 mL), dried over $MgSO_4$ and concentrated under reduced pressure to give a crude residue. Purification by flash silica chromatography (30% EtOAc/Hexane) afforded the title compound (420 mg, 84%) as orange needles.

MP: 143-145 °C

***V*$_{max}$ /cm^{-1}(neat):** 2936, 1718, 1709 1452, 1328, 1121, 1071, 699

^{1}H NMR (400 MHz, $CDCl_3$) δ 7.57-7.33 (m, 7H), 7.19-7.13 (m, 1H), 3.44 (s, 6H); **^{13}C NMR** (100 MHz, CDCl3) δ 160.1, 141.4, 132.2 (d, J_{C-F} = 33.4 Hz), 130.6, 130.10, 124.9, (d, J_{C-F} = 23.0 Hz), 123.3 (q, J_{C-F} = 272.7 Hz), 121.7 (d, J_{C-F} = 22.0 Hz), 38.6; HRMS (ESI) calcd for [$C_{18}H_{14}F_6N_4O_2Na$] requires [M + Na]+ 455.0919, found 455.0903

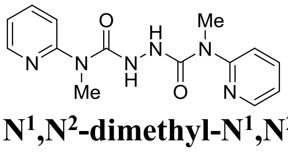

N^1,N^2-dimethyl-N^1,N^2-di(pyridin-2-yl)hydrazine-1,2-dicarboxamide (**S1g**). A solution of N-methyl(pyridin-2-yl)carbamic chloride (400 mg, 2.35 mmol) in MeCN (1.56 mL, 1.5 M)

was added dropwise to a solution of hydrazine monohydrate (75mg, 2.3 mmol, 1 equiv) in EtOH (1.56 mL, 1.5 M) at 0 ºC. After 10 minutes a solution of N-methyl(pyridin-2-yl)carbamic chloride (400 mg, 2.35 mmol) in MeCN (1.56 mL, 1.5 M) and a solution of Na_2CO_3 (247 mg 2.3 mmol) in H_2O (3.1 mL, 0.75 M) were added to the reaction flask simultaneously. The resulting solution was stirred at room temperature for 20 h, giving a precipitate. The reaction was concentrated under reduced pressure, re-dissolved in DCM, filtered and concentrated under reduced pressure to give a crude residue. Purification by flash silica chromatography (2% MeOH/DCM) afforded the title compound (560 mg, 79%) as a pale white solid.

MP: 160-162 ºC

***V*max /cm^{-1}(neat):** 3112, 2983, 1666, 1592, 1573, 1469, 1431, 1316, 1133, 774

^{1}H NMR (400 MHz; $CDCl_3$) δ 12.0 (br s, 2H), 8.31 – 8.29 (m, 2H), 7.71 – 7.67 (m, 2H), 700–6.94 (m, 4H), 3.42 (s, 6H); **^{13}C NMR** (101 MHz; $CDCl_3$) δ 156.8, 155.4, 146.3, 138.8, 117.5, 111.6, 33.1; HRMS m/z (ESI$^+$) [$C_{14}H_{16}N_6O_2Na$] requires: 323.1232; found: 323.1228.

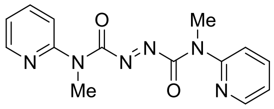

(*E*)-N^1,N^2-dimethyl-N^1,N^2-di(pyridin-2-yl)diazene-1,2-dicarboxamide (1g); A solution of *N*-bromosuccinimide (396 mg, 2.23 mmol, 1.2 equiv) in DCM (12 mL, 0.15 M) was added dropwise to a solution of pyridine (0.34 mL, 4.2 mmol, 2 equiv) and N^1,N^2-dimethyl-N^1,N^2-di(pyridin-2-yl)hydrazine-1,2-dicarboxamide **S1g** (560 mg, 1.8 mmol) in DCM (6 mL, 0.30 M) at 0 ºC. The reaction was stirred at RT for 3 h, before being quenched with sat. aq. $NaHCO_3$ (40 mL) and the aqueous layer was extracted with DCM (3 × 20 mL). The combined organics were washed with brine (1 × 20 mL), dried over $MgSO_4$ and concentrated under reduced pressure to give a crude residue. Purification by flash silica chromatography (40% EtOAc/Hexane) afforded the title compound (438 mg, 78%) as orange needles.

MP: 134-136 ºC

***V*max /cm^{-1}(neat):** 2918, 1768, 1717, 1437, 1362, 1113, 785

^{1}H NMR (400 MHz, $CDCl_3$) δ 8.42 (d, *J* = 4.0 Hz, 2H), 7.73–7.69 (m, 2H), 7.26–7.25 (m, 2H), 7.17–7.14 (m, 2H), 3.35 (br s, 6H); **^{13}C NMR** (101 MHz, CDCl3) δ 161.0, 152.9, 148.3, 137.9, 121.8, 119.6, 29.5; HRMS (ESI) calcd for [$C_{14}H_{14}N_6O_2Na$] requires [M + Na]+ 321.1076, found 321.1071

1.8 General Procedure 4: Carbamoyl Chloride and *tert*-Butyl carbamate coupling

Pyridine (2.0 equiv) was added in one portion to a solution of carbamoyl chloride and tert-butyl carbazate (1.3 equiv) in MeCN (0.5 M) and the resulting solution stirred at RT for 18-40 h. The reaction was quenched with H_2O (20 mL) and the aqueous layer was extracted with DCM (3 × 15 mL). The combined organics were washed with brine (1 × 15 mL), dried over MgSO4 and concentrated under reduced pressure to give a crude residue. Purification by flash silica chromatography.

1.9 General Procedure 5: Synthesis of unsymmetrical α, β unsaturated azocarbonamides

A solution of *N*-bromosuccinimide (1.2-1.5 equiv) in DCM (0.15 M) was added dropwise to a solution of pyridine (2 equiv) and arylcarbamoyl)hydrazine-1-carboxylate **S5** in DCM (11 mL, 0.30 M) at 0 ºC. The reaction was stirred at RT for 3 h, before being quenched with sat. aq. $NaHCO_3$ (40 mL) and the aqueous layer was extracted with DCM (3 × 20 mL). The combined organics were washed with brine (1 × 20 mL), dried over $MgSO_4$ and concentrated under reduced pressure to give a crude residue. Purification by flash silica chromatography afforded the s an orange solid.

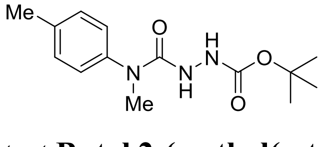

***tert*-Butyl 2-(methyl(*p*-tolyl)carbamoyl)hydrazine-1-carboxylate** (**S5a**). Pyridine (0.69 mL, 2 equiv) was added in one portion to a solution of methyl(*p*-tolyl)carbamic chloride (700 mg, 3.8 mmol, 1.0 equiv) and *tert*-butyl carbazate (656 mg, 4.9 mmol, 1.3 equiv) in MeCN (7.6 mL, 0.5 M) and the resulting solution stirred at RT for 18 h. The reaction was quenched with

H_2O (20 mL) and the aqueous layer was extracted with DCM (3 × 15 mL). The combined organics were washed with brine (1 × 15 mL), dried over $MgSO_4$ and concentrated under reduced pressure to give a crude residue. Purification by flash silica chromatography (40% EtOAc/Petrol) afforded the title compound (1100 mg, 96%) as white needles.

M.P: 106-108 °C

***V*$_{max}$ /cm^{-1}(neat):** 3279, 2977, 1725, 1672, 1513, 1366, 1161, 1018, 754

^{1}H NMR (400 MHz; $CDCl_3$) δ 7.19 (d, *J* = 6.5 Hz, 4H), 6.32 (s, 1H), 6.03 (s, 1H), 3.23 (s, 3H), 2.34 (s, 3H), 1.41 (s, 9H); **^{13}C NMR** (101 MHz; $CDCl_3$) δ 157.2, 156.5, 139.5, 137.9, 130.8, 127.1, 81.2, 37.8, 28.3, 21.1; **HRMS m/z** (ESI^+) $[C_{14}H_{21}N_3O_3Na]^+$ requires[M + Na]$^+$: 302.1481; found: 302.1493

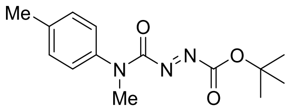

tert-Butyl (*E*)-2-(methyl(p-tolyl)carbamoyl)diazene-1-carboxylate (5a). A solution of *N*-bromosuccinimide (952 mg, 4.5 mmol, 1.5 equiv) in DCM (22 mL, 0.15 M) was added dropwise to a solution of pyridine (0.651 mL, 7.1 mmol, 2 equiv) and tert-butyl 2-(methyl(*p*-tolyl)carbamoyl)hydrazine-1-carboxylate **S4a** (1000 mg, 3.5 mmol) in DCM (11 mL, 0.30 M) at 0 °C. The reaction was stirred at RT for 3 h, before being quenched with sat. aq. $NaHCO_3$ (40 mL) and the aqueous layer was extracted with DCM (3 × 20 mL). The combined organics were washed with brine (1 × 20 mL), dried over $MgSO_4$ and concentrated under reduced pressure to give a crude residue. Purification by flash silica chromatography (25% EtOAc/Hexane) afforded the title compound (860 mg, 87%) as an orange solid.

M.P: 83-85 °C

***V*$_{max}$ /cm^{-1}(neat):** 2982, 1762, 1710, 1513, 1371, 1254, 1150, 835

^{1}H NMR (400 MHz, $CDCl_3$) δ 7.13 (d, *J* = 8.1 Hz, 2H), 6.99 (d, *J* = 8.1 Hz, 2H), 3.47 (s, 3H), 2.32 (s, 3H), 1.48 (s, 9H); **^{13}C NMR** (101 MHz, $CDCl_3$) δ 161.0, 159.8, 138.0, 137.9, 130.0,

127.1, 86.2, 38.7, 27.8, 21.1; HRMS (ESI) calcd for $[C_{14}H_{19}N_3O_3Na]^+$ requires $[M + Na]^+$ 300.1324, found 300.1309.

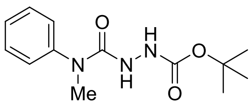

tert-Butyl 2-(methyl(phenyl)carbamoyl)hydrazine-1-carboxylate (**S5b**). Pyridine (1.07 mL, 11.8 mmol, 2.0 equiv) was added in one portion to a solution of *N*-methyl-*N*-phenylcarbamoyl chloride (1 g, 5.9 mmol, 1.1 equiv) and *tert*-butyl carbazate (0.7 mg, 5.36 mmol, 1 equiv) in MeCN (25 mL, 0.21 M) and the resulting solution stirred at RT for 18 h. The reaction was quenched with H_2O (20 mL) and the aqueous layer was extracted with DCM (3 × 15 mL). The combined organics were washed with brine (1 × 15 mL), dried over $MgSO_4$ and concentrated under reduced pressure to give a crude residue. Purification by flash silica chromatography (40% EtOAc/Petrol) afforded the *title compound* (1.1 g, 71%) as colourless oil.

***V*max /cm^{-1}(neat):** 3287, 2977, 1690, 1494, 1157, 699

^{1}H NMR (400 MHz; $CDCl_3$) δ 7.47 – 7.40 (m, 2H), 7.38 – 7.29 (m, 3H), 6.42 (br s 1H), 6.11 (br s, 1H), 3.30 (s, 3H), 1.47 (s, 9H); **^{13}C NMR** (101 MHz; $CDCl_3$) δ 156.9, 156.4, 142.8, 130.2, 127.8, 127.2, 81.2, 37.7, 28.2; HRMS (ESI$^+$) $[C_{13}H_{19}N_3O_3Na]^+$ requires [M+Na]+: 288.1324; found: 288.1317

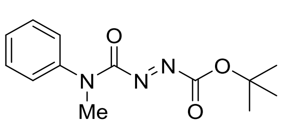

***tert*-Butyl (*E*)-2-(methyl(phenyl)carbamoyl)diazene-1-carboxylate (5b).** A solution of *N*-bromosuccinimide (540 mg, 3.05 mmol, 1.2 equiv) in DCM (16 mL, 0.15 M) was added dropwise to a solution of pyridine (0.46 mL, 6.12 mmol, 2 equiv) and tert-butyl (E)-2-(methyl(phenyl)carbamoyl)diazene-1-carboxylate **S5b** (674 mg, 2.5 mmol) in DCM (8 mL, 0.30 M) at 0 ^oC. The reaction was stirred at RT for 5 h, before being quenched with sat. aq. $NaHCO_3$ (40 mL) and the aqueous layer was extracted with DCM (3 × 20 mL). The combined

organics were washed with brine (1 × 20 mL), dried over $MgSO_4$ and concentrated under reduced pressure to give a crude residue. Purification by flash silica chromatography (30% EtOAc/Hexane) afforded the *title compound* (357 mg, 53%) as an orange solid.

MP: 65-67 °C

***V*max /cm^{-1}(neat):** 2982, 1762, 1711, 1598, 1371, 1255, 1149, 697

^{1}H NMR (400 MHz, $CDCl_3$) δ 7.40-7.36 (m, 2H), 7.32 (d, *J* = 8.0 Hz, 1H), 7.16 (d, *J* = 8.0 Hz, 2H), 3.55 (s, 3H) 1.51 (s, 9H); **^{13}C NMR** (100 MHz, $CDCl_3$) δ 160.8, 159.7, 140.5, 129.3, 127.9, 127.3, 86.2, 38.6, 27.7; HRMS (ESI) calcd for [$C_{13}H_{17}N_3O_3Na$] requires [M+ Na]+ 286.1168, found 286.1028

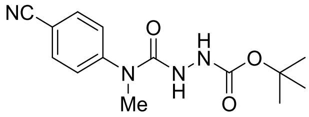

***tert*-Butyl 2-((4-cyanophenyl)(methyl)carbamoyl)hydrazine-1-carboxylate** (**S5c**). Pyridine (0.65mL, 2 equiv) was added in one portion to a solution of (4-cyanophenyl)(methyl)carbamic chloride (700 g, 3.6 mmol, 1.0 equiv) and *tert*-butyl carbazate (714 mg, 5.4 mmol, 1.5 equiv) in MeCN (7.2 mL, 0.5 M) and the resulting solution stirred at RT for 18 h. The reaction was quenched with H_2O (20 mL) and the aqueous layer was extracted with DCM (3 × 15 mL). The combined organics were washed with brine (1 × 15 mL), dried over $MgSO_4$ and concentrated under reduced pressure to give a crude residue. Purification by flash silica chromatography (40% EtOAc/Petrol) afforded the title compound (1g, 95.6%) as a white solid.

MP: 161-163 °C

***V*max /cm^{-1}(neat):** 3300, 2980, 2227, 1722, 1673, 1603, 1479, 1367, 1159, 1111, 847

^{1}H NMR (400 MHz; $CDCl_3$) δ 7.72 – 7.67 (m, 2H), 7.50 (d, *J* = 7.8 Hz, 2H), 6.37 (s, 1H), 6.25 (s, 1H), 3.32 (s, 3H), 1.45 (s, 9H); **^{13}C NMR** (101 MHz; $CDCl_3$) δ 156.4, 146.8, 133.9, 126.9, 118.1, 110.5, 81.8, 37.4, 28.2; HRMS m/z (ESI$^+$) [$C_{14}H_{18}N_4O_3Na$]$^+$ requires[M + Na]$^+$: 313.1277; found: 313.1283

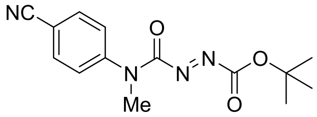

***tert*-Butyl (*E*)-2-((4-cyanophenyl)(methyl)carbamoyl)diazene-1-carboxylate (5c).** A solution of *N*-bromosuccinimide (952 mg, 4.5 mmol, 1.5 equiv) in DCM (22 mL, 0.15 M) was added dropwise to a solution of pyridine (0.651 mL, 7.1 mmol, 2 equiv) and tert-butyl 2-((4-cyanophenyl)(methyl)carbamoyl)hydrazine-1-carboxylate **S5c** (1040 mg, 3.5 mmol) in DCM (11 mL, 0.30 M) at 0 °C. The reaction was stirred at RT for 3 h, before being quenched with sat. aq. $NaHCO_3$ (40 mL) and the aqueous layer was extracted with DCM (3 × 20 mL). The combined organics were washed with brine (1 × 20 mL), dried over $MgSO_4$ and concentrated under reduced pressure to give a crude residue. Purification by flash silica chromatography (25% EtOAc/Hexane) afforded the title compound (930 mg, 90%) as an orange solid.

MP: 92-94 °C

***V*max /cm^{-1}(neat):** 2984, 2230, 1760, 1709, 1603, 1253, 1147, 833

^{1}H NMR (400 MHz, $CDCl_3$) δ 7.67 (d, *J* = 6.6 Hz, 2H), 7.30 – 7.20 (m, 2H), 3.52 (s, 3H), 1.52 (s, 9H); **^{13}C NMR** (126 MHz, $CDCl_3$) δ 159.6, 144.6, 133.2, 127.8, 117.9, 111.6, 86.9, 38.3, 27.7; HRMS (ESI) calcd for $[C_{14}H_{16}N_4O_3Na]^+$ requires $[M + Na]^+$ 311.1120, found 311.1118

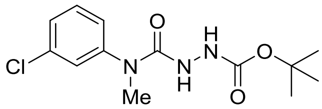

***tert*-Butyl 2-((3-chlorophenyl)(methyl)carbamoyl)hydrazine-1-carboxylate (S5d).** Pyridine (0.65mL, 2 equiv) was added in one portion to a solution of (3-chlorophenyl)(methyl)carbamic chloride (730 g, 3.6 mmol, 1.0 equiv) and *tert*-butyl carbazate (715 mg, 5.4 mmol, 1.5 equiv) in MeCN (7.2 mL, 0.5 M) and the resulting solution stirred at RT for 18 h. The reaction was quenched with H_2O (20 mL) and the aqueous layer was extracted with DCM (3 × 15 mL). The combined organics were washed with brine (1 × 15 mL), dried over $MgSO_4$ and concentrated under reduced pressure to give a crude residue. Purification by flash silica chromatography (40% EtOAc/Petrol) afforded the title compound (1000 mg, 90%) as white needles.

MP: 87-91 °C

V_{max} **/cm^{-1}(neat):** 3285, 2979, 1680, 1667, 1476, 1366, 1237, 1157, 696

^{1}H NMR (400 MHz; $CDCl_3$) δ 7.36 – 7.30 (m, 2H), 7.25 (s, 2H), 6.47 (s, 1H), 6.15 (s, 1H), 3.24 (s, 3H), 1.43 (s, 9H); **^{13}C NMR** (126 MHz; $CDCl_3$) δ 156.7, 156.4, 143.5, 135.5, 131.1, 128.0, 127.5, 125.4, 123.8, 81.5, 37.7, 28.4; HRMS m/z (ESI$^+$) $[C_{13}H_{18}ClN_3O_3Na]^+$ requires[M + Na]$^+$: 322.0934; found: 322.0919

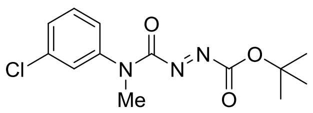

***tert*-Butyl (*E*)-2-((3-chlorophenyl)(methyl)carbamoyl)diazene-1-carboxylate (5d).** A solution of *N*-bromosuccinimide (799 mg, 4.5 mmol, 1.5 equiv) in DCM (20 mL, 0.15 M) was added dropwise to a solution of pyridine (0.546 mL, 6.0 mmol, 2 equiv) and tert-butyl 2-((3-chlorophenyl)(methyl)carbamoyl)hydrazine-1-carboxylate **S5d** (900 mg, 3.01 mmol) in DCM (10 mL, 0.30 M) at 0 °C. The reaction was stirred at RT for 3 h, before being quenched with sat. aq. $NaHCO_3$ (40 mL) and the aqueous layer was extracted with DCM (3 × 20 mL). The combined organics were washed with brine (1 × 20 mL), dried over $MgSO_4$ and concentrated under reduced pressure to give a crude residue. Purification by flash silica chromatography (15% EtOAc/Hexane) afforded the title compound (750 mg, 83%) as an orange semi-solid.

V_{max} **/cm^{-1}(neat):** 2983, 1761, 1709, 1591, 1370, 1251, 1147, 694

^{1}H NMR (400 MHz, $CDCl_3$) δ 7.28 (d, *J* = 5.5 Hz, 2H), 7.15 (s, 1H), 7.02 (d, *J* = 6.1 Hz, 1H), 3.50 (s, 3H), 1.50 (s, 9H); **^{13}C NMR** (101 MHz, CDCl3) δ 160.5, 159.7, 141.7, 134.9, 130.3, 128.3, 127.6, 125.7, 86.6, 38.5, 27.8; HRMS (ESI) calcd for $[C_{13}H_{16}ClN_3O_3Na]^+$ requires [M + Na]$^+$ 320.0778, found 320.0777

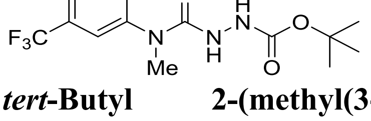

***tert*-Butyl 2-(methyl(3-(trifluoromethyl)phenyl)carbamoyl)hydrazine-1-carboxylate** (**S5e**). Pyridine (0.64 mL, 7.0 mmol, 2 equiv) was added in one portion to a solution of N-methyl(3-(trifluoromethyl)phenyl)carbamic chloride (840 g, 3.5 mmol, 1.0 equiv) and *tert*-

butyl carbazate (608 mg, 4.6 mmol, 1.3 equiv) in MeCN (7.0 mL, 0.5 M) and the resulting solution stirred at RT for 18 h. The reaction was quenched with H_2O (20 mL) and the aqueous layer was extracted with DCM (3 × 15 mL). The combined organics were washed with brine (1 × 15 mL), dried over $MgSO_4$ and concentrated under reduced pressure to give a crude residue. Purification by flash silica chromatography (20% to 40% EtOAc/Petrol) afforded the title compound (1.1 g, 93%) as a white needle.

MP: 151-153 °C

***V*max /cm^{-1}(neat):** 3286, 2980, 1716, 1671, 1493, 1331, 1163, 1126, 1070

^{1}H NMR (400 MHz; $CDCl_3$) δ 7.47 – 7.40 (m, 4H), 6.29 (br s 1H), 6.03 (br s, 1H), 3.31 (s, 3H), 1.45 (s, 9H). **^{13}C NMR** (126 MHz; $CDCl_3$) δ 156.6, 156.3, 142.9, 132.6 (q, J_{C-F} =32.9 Hz), 130.8, 130.6, 124.4 (q, J_{C-F} =3.6 Hz), 123.9 (q, J_{C-F} =3.7 Hz), 123.7 (q, J_{C-F} = 272.2 Hz), 81.6, 37.8, 28.2; HRMS m/z (ESI$^+$) $[C_{14}H_{18}F_3N_3O_3Na]^+$ requires[M + Na]$^+$: 356.1198; found: 356.1199

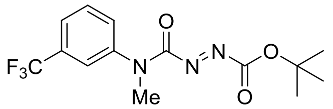

***tert*-Butyl (*E*)-2-(methyl(3-(trifluoromethyl)phenyl)carbamoyl)diazene-1-carboxylate (5e).** A solution of *N*-bromosuccinimide (877 mg, 4.95 mmol, 1.5 equiv) in DCM (22 mL, 0.15 M) was added dropwise to a solution of pyridine (0.599 mL, 6.59 mmol, 2 equiv) and *tert*-butyl 2-(methyl(3-(trifluoromethyl)phenyl)carbamoyl)hydrazine-1-carboxylate **S5e** (1.1g, 3.3 mmol) in DCM (11 mL, 0.30 M) at 0 °C. The reaction was stirred at RT for 3 h, before being quenched with sat. aq. $NaHCO_3$ (40 mL) and the aqueous layer was extracted with DCM (3 × 20 mL). The combined organics were washed with brine (1 × 20 mL), dried over $MgSO_4$ and concentrated under reduced pressure to give a crude residue. Purification by flash silica chromatography (20% EtOAc/Hexane) afforded the title compound (874 mg, 80%) as an orange Semi-solid.

***V*max /cm^{-1}(neat):** 2986, 1764, 1713, 1371, 1329, 1253, 1123, 801, 700

^{1}H NMR (400 MHz, $CDCl_3$) δ 7.55 (d, *J* = 7.5 Hz, 1H), 7.49 (t, *J* = 7.8 Hz, 1H), 7.39 (s, 1H), 7.34 (d, *J* = 7.5 Hz, 1H), 3.54 (s, 3H), 1.47 (s, 9H); **^{13}C NMR** (100 MHz, CDCl3) δ 160.2, 159.6, 141.2, 131.9 (q, $J_{C\text{-}F}$ = 33.1 Hz), 130.5, 130.0, 125.8 (q, $J_{C\text{-}F}$ = 272.6 Hz), 124.7 (q, $J_{C\text{-}F}$ = 2.5 Hz), 124.3 (q, $J_{C\text{-}F}$ = 2.8 Hz), 122.7 (q, $J_{C\text{-}F}$ = 272.3 Hz), 86.6, 38.5, 27.6; HRMS (ESI) calcd for $[C_{14}H_{16}F_3N_3O_3Na]^+$ requires $[M + Na]^+$ 354.1041, found 354.1035

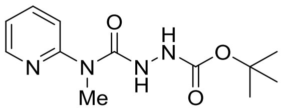

***tert*-Butyl 2-(methyl(pyridin-2-yl)carbamoyl)hydrazine-1-carboxylate** (**S5f**). Pyridine (0.60 mL, 2 equiv) was added in one portion to a solution of *N*-methyl(pyridin-2-yl)carbamic chloride (568 g, 3.3 mmol, 1.0 equiv) and *tert*-butyl carbazate (573 mg, 4.3 mmol, 1.3 equiv) in MeCN (6.68 mL, 0.5 M) and the resulting solution stirred at RT for 18 h. The reaction was quenched with H_2O (20 mL) and the aqueous layer was extracted with DCM (3 × 15 mL). The combined organics were washed with brine (1 × 15 mL), dried over $MgSO_4$ and concentrated under reduced pressure to give a crude residue. Purification by flash silica chromatography (50% EtOAc/Petrol) afforded the title compound (805 mg, 90%) as white needles.

MP: 155-157 °C

V_{max} /cm^{-1}(neat): 3273, 2977, 1731, 1690, 1477, 1436, 1162, 776

^{1}H NMR (400 MHz; $CDCl_3$) δ 11.58 (d, *J* = 2.5 Hz, 1H), 8.28–8.26 (m, 1H), 7.70 (dddd, *J* = 8.0, 7.4, 2.0, 0.6 Hz, 1H), 6.99 (m, 2H), 6.45 (d, *J* = 2.5 Hz, 1H), 3.40 (s, 3H), 1.47 (s, 9H); **^{13}C NMR** (126 MHz; $CDCl_3$) δ 157.5, 156.2, 155.2, 146.2, 138.9, 117.7, 111.7, 81.3, 33.2, 28.3; **HRMS m/z** (ESI^+) $[C_{12}H_{18}N_4O_3Na]^+$ requires$[M + Na]^+$: 289.1277; found: 289.1272.

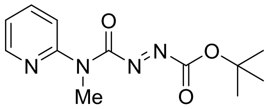

***tert*-Butyl (*E*)-2-(methyl(pyridin-2-yl)carbamoyl)diazene-1-carboxylate (5f).** A solution of *N*-bromosuccinimide (788 mg, 4.46 mmol, 1.5 equiv) in DCM (19 mL, 0.15 M) was added dropwise to a solution of pyridine (0.539 mL, 5.93 mmol, 2 equiv) and *tert*-butyl 2-(methyl

(pyridin-2-yl)carbamoyl)hydrazine-1-carboxylate **S5f** (790 mg, 2.96 mmol) in DCM (9.5 mL, 0.30 M) at 0 °C. The reaction was stirred at RT for 3 h, before being quenched with sat. aq. $NaHCO_3$ (40 mL) and the aqueous layer was extracted with DCM (3 × 20 mL). The combined organics were washed with brine (1 × 20 mL), dried over $MgSO_4$ and concentrated under reduced pressure to give a crude residue. Purification by flash silica chromatography (20% EtOAc/Hexane) afforded the title compound (588 mg, 75%) as an orange oil.

***V*$_{max}$ /cm^{-1}(neat):** 2982, 1760, 1708, 1587, 1470, 1437, 1369, 1252, 1146, 1110, 784

^{1}H NMR (400 MHz, $CDCl_3$) δ 8.47 – 8.43 (m, 1H), 7.71 (t, *J* = 7.6 Hz, 1H), 7.28 – 7.24 (m, 1H), 7.17 (ddd, *J* = 7.5, 4.9, 0.9 Hz, 1H), 3.57 (s, 3H), 1.58 (s, 9H); **^{13}C NMR** (126 MHz, CDCl3) δ 160.4, 159.8, 152.9, 148.5, 148.1, 137.8, 121.8, 86.6, 35.8, 27.7; HRMS (ESI) calcd for $[C_{12}H_{16}N_4O_3Na]^+$ requires $[M + Na]^+$ 287.1120, found 287.1116.

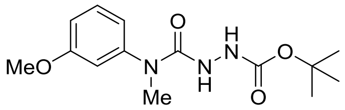

***tert*-Butyl 2-((3-methoxyphenyl)(methyl)carbamoyl)hydrazine-1-carboxylate** (**S5g**). Pyridine (0.67 mL, 2 equiv) was added in one portion to a solution of (3-methoxyphenyl)(methyl)carbamic chloride (736 g, 3.6 mmol, 1.0 equiv) and *tert*-butyl carbazate (634 mg, 4.8 mmol, 1.3 equiv) in MeCN (7.3 mL, 0.5 M) and the resulting solution stirred at RT for 18 h. The reaction was quenched with H_2O (20 mL) and the aqueous layer was extracted with DCM (3 × 15 mL). The combined organics were washed with brine (1 × 15 mL), dried over $MgSO_4$ and concentrated under reduced pressure to give a crude residue. Purification by flash silica chromatography (40% EtOAc/Petrol) afforded the title compound (1g, 91%) as a white semi-solid.

***V*$_{max}$ /cm^{-1}(neat):** 3288, 2977, 1690, 1673, 1598, 1487, 1366, 1231, 1157, 1042, 701

^{1}H NMR (400 MHz; $CDCl_3$) δ 7.29 (t, *J* = 8.2 Hz, 1H), 6.91 – 6.81 (m, 3H), 6.45 (s, 1H), 6.17 (s, 1H), 3.78 (s, 3H), 3.24 (s, 3H), 1.42 (s, 9H); **^{13}C NMR** (101 MHz; $CDCl_3$) δ 160.8, 157.0,

156.4, 143.3, 130.8, 119.2, 113.6, 112.8, 81.3, 55.5, 37.6, 28.2; **HRMS m/z** (ESI$^+$) $[C_{14}H_{21}N_3O_3Na]^+$ requires[M + Na]$^+$: 318.1430; found: 318.1424

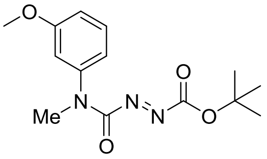

***tert*-butyl (*E*)-2-((3-methoxyphenyl)(methyl)carbamoyl)diazene-1-carboxylate (5g).** A solution of *N*-bromosuccinimide (621 mg, 3.5 mmol, 1.5 equiv) in DCM (15.5 mL, 0.15 M) was added dropwise to a solution of pyridine (0.424 mL, 4.6 mmol, 2 equiv) and tert-butyl 2-((4-methoxyphenyl)(methyl)carbamoyl)hydrazine-1-carboxylate **S5g** (690 mg, 2.3 mmol) in DCM (7.75 mL, 0.30 M) at 0 °C. The reaction was stirred at RT for 3 h, before being quenched with sat. aq. $NaHCO_3$ (20 mL) and the aqueous layer was extracted with DCM (3 × 15 mL). The combined organics were washed with brine (1 × 20 mL), dried over $MgSO_4$ and concentrated under reduced pressure to give a crude residue. Purification by flash silica chromatography (15% EtOAc/Hexane) afforded the title compound (500 mg, 72%) as orange needles.

MP: 97-99 °C

V_{max} **/cm^{-1}(neat):** 2981, 1759, 1720, 1602, 1371, 1254, 1148, 698

^{1}H NMR (400 MHz, $CDCl_3$) δ 7.22 (d, *J* = 8.1 Hz, 1H), 6.81 (dd, *J* = 8.3, 2.2 Hz, 1H), 6.71 (d, *J* = 7.8 Hz, 1H), 6.63 (t, *J* = 2.0 Hz, 1H), 3.77 (s, 3H), 3.50 (s, 3H), 1.49 (s, 9H); **^{13}C NMR** (101 MHz, CDCl3) δ 160.9, 160.2, 159.8, 141.5, 130.0, 119.4, 114.0, 112.9, 86.3, 55.4, 38.5, 27.7; HRMS (ESI) calcd for $[C_{14}H_{19}N_3O_4Na]^+$ requires [M + Na]$^+$ 316.1273, found 316.1279.

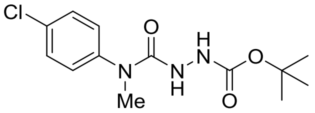

***tert-B*utyl 2-((4-chlorophenyl)(methyl)carbamoyl)hydrazine-1-carboxylate (S5h)**. Pyridine (0.62 mL, 2 equiv) was added in one portion to a solution of (4-chlorophenyl)(methyl)carbamic chloride (700 g, 3.4 mmol, 1.0 equiv) and *tert*-butyl carbazate (594 mg, 4.5 mmol, 1.5 equiv) in MeCN (6.9 mL, 0.5 M) and the resulting solution stirred at

RT for 18 h. The reaction was quenched with H_2O (20 mL) and the aqueous layer was extracted with DCM (3 × 15 mL). The combined organics were washed with brine (1 × 15 mL), dried over $MgSO_4$ and concentrated under reduced pressure to give a crude residue. Purification by flash silica chromatography (40% EtOAc/Petrol) afforded the title compound (995 mg, 96%) as white needles.

MP: 84-86 ^oC

***V*max /cm^{-1}(neat):** 3287, 2978, 1716, 1668, 1490, 1366, 1158, 1013

^{1}H NMR (400 MHz; $CDCl_3$) δ 7.39 (d, *J* = 2.2 Hz, 1H), 7.37 (d, *J* = 2.1 Hz, 1H), 7.28 (d, *J* = 8.3 Hz, 2H), 6.37 (s, 1H), 6.05 (s, 1H), 3.24 (s, 3H), 1.43 (s, 9H); **^{13}C NMR** (101 MHz; $CDCl_3$) δ 156.8, 156.4, 140.7, 133.6, 130.4, 128.6, 81.5, 37.7, 28.2; HRMS m/z (ESI$^+$) $[C_{13}H_{18}ClN_3O_3Na]^+$ requires$[M + Na]^+$: 322.0934; found: 322.0922

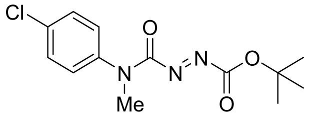

***tert*-Butyl (*E*)-2-((4-chlorophenyl)(methyl)carbamoyl)diazene-1-carboxylate (5h).** A solution of *N*-bromosuccinimide (976 mg, 5.51 mmol, 1.5 equiv) in DCM (24 mL, 0.15 M) was added dropwise to a solution of pyridine (0.668 mL, 7.3 mmol, 2 equiv) and tert-butyl 2-((4-chlorophenyl)(methyl)carbamoyl)hydrazine-1-carboxylate **S5h** (1100 mg, 3.6 mmol) in DCM (12 mL, 0.30 M) at 0 ^oC. The reaction was stirred at RT for 3 h, before being quenched with sat. aq. $NaHCO_3$ (40 mL) and the aqueous layer was extracted with DCM (3 × 20 mL). The combined organics were washed with brine (1 × 20 mL), dried over $MgSO_4$ and concentrated under reduced pressure to give a crude residue. Purification by flash silica chromatography (20% EtOAc/Hexane) afforded the title compound (896 mg, 82%) as an orange Needles.

MP: 80-82 ^oC

***V*max /cm^{-1}(neat):** 2983, 1761, 1708, 1492, 1252, 1148, 834

^{1}H NMR (400 MHz, $CDCl_3$) δ 7.32 (d, *J* = 8.6 Hz, 2H), 7.07 (d, *J* = 8.6 Hz, 2H), 3.49 (s, 3H), 1.50 (s, 9H); **^{13}C NMR** (101 MHz, $CDCl_3$) δ 160.5, 159.7, 139.1, 133.9, 129.6, 128.7, 86.5, 38.6, 27.7; HRMS (ESI) calcd for $[C_{13}H_{16}ClN_3O_3Na]^+$ requires $[M + Na]^+$ 320.0778, found 320.0772

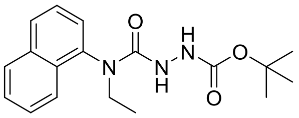

tert-Butyl 2-(ethyl(naphthalen-1-yl)carbamoyl)hydrazine-1-carboxylate (**S5i**); Pyridine (0.36 mL, 2 equiv) was added in one portion to a solution of ethyl(naphthalen-1-yl)carbamic chloride (470 g, 2.1 mmol, 1.0 equiv) and *tert*-butyl carbazate (346 mg, 2.6 mmol, 1.3 equiv) in MeCN (4.0 mL, 0.5 M) and the resulting solution stirred at RT for 42 h. The reaction was quenched with H_2O (20 mL) and the aqueous layer was extracted with DCM (3 × 15 mL). The combined organics were washed with brine (1 × 15 mL), dried over $MgSO_4$ and concentrated under reduced pressure to give a crude residue. Purification by flash silica chromatography (30% EtOAc/Petrol) afforded the title compound (500, 75%) as a white semi-solid.

***V_{max}* /cm^{-1}(neat):** 3289, 2976, 1720, 1671, 1480, 1366, 1158, 776

^{1}H NMR (400 MHz; $CDCl_3$) δ 7.90 (dt, *J* = 17.2, 9.0 Hz, 3H), 7.59 – 7.46 (m, 4H), 6.22 (s, 1H), 5.89 (s, 1H), 4.14 (dq, *J* = 14.2, 7.1 Hz, 1H), 3.49 (dq, *J* = 14.1, 7.1 Hz, 1H), 1.42 (s, 9H), 1.14 (t, *J* = 7.1 Hz, 3H); **^{13}C NMR (101 MHz; $CDCl_3$)** δ 156.3, 136.1, 135.0, 130.8, 129.2, 128.5, 127.5, 127.3, 126.9, 126.0, 122.9, 81.2, 44.7, 28.2, 13.8; HRMS (ESI^+) $[C_{18}H_{23}N_3O_3Na]^+$ requires$[M + Na]^+$: 352.1637; found: 352.1628.

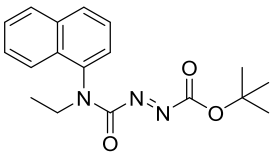

tert-Butyl (*E*)-2-(ethyl(naphthalen-1-yl)carbamoyl)diazene-1-carboxylate (5i); A solution of *N*-bromosuccinimide (322 mg, 1.8 mmol, 1.5 equiv) in DCM (8.1 mL, 0.15 M) was added dropwise to a solution of pyridine (0.220 mL, 2.4 mmol, 2 equiv) and tert-butyl 2-

(ethyl(naphthalen-1-yl)carbamoyl)hydrazine-1-carboxylate **S5i** (400 mg, 1.2 mmol) in DCM (4 mL, 0.30 M) at 0 °C. The reaction was stirred at RT for 3 h, before being quenched with sat. aq. $NaHCO_3$ (20 mL) and the aqueous layer was extracted with DCM (3 × 15 mL). The combined organics were washed with brine (1 × 20 mL), dried over $MgSO_4$ and concentrated under reduced pressure to give a crude residue. Purification by flash silica chromatography (10% EtOAc/Hexane) afforded the title compound (264 mg, 66%) as an orange semi-solid.

***V*max /cm^{-1}(neat):** 2936, 1761, 1709, 1372, 1268, 1254, 1150, 776

^{1}H NMR (400 MHz, $CDCl_3$) δ 7.85 (t, *J* = 9.2 Hz, 3H), 7.60 – 7.50 (m, 2H), 7.46 – 7.41 (m, 1H), 7.31 (d, *J* = 7.3 Hz, 1H), 4.32 (dq, *J* = 14.4, 7.2 Hz, 1H), 3.70 (dq, *J* = 14.3, 7.2 Hz, 1H), 1.30–1.26 (m, 12H); **^{13}C NMR** (101 MHz, CDCl3) δ 161.4, 159.5, 134.6, 134.5, 130.4, 129.4, 128.5, 127.7, 127.5, 126.7, 125.2, 122.7, 85.8, 45.8, 27.5, 13.1; HRMS (ESI) calcd for $[C_{18}H_{21}N_3O_3Na]^+$ requires $[M + Na]^+$ 350.1481, found 350.1491.

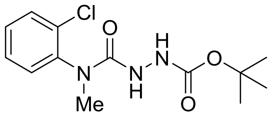

***tert-B*utyl 2-((2-chlorophenyl)(methyl)carbamoyl)hydrazine-1-carboxylate** (**S5j**); Pyridine (0.31 mL, 2 equiv) was added in one portion to a solution of (2-chlorophenyl)(methyl)carbamic chloride (352 g, 1.7 mmol, 1.0 equiv) and *tert*-butyl carbazate (299 mg, 2.2 mmol, 1.3 equiv) in MeCN (3.4 mL, 0.5 M) and the resulting solution stirred at RT for 18 h. The reaction was quenched with H_2O (20 mL) and the aqueous layer was extracted with DCM (3 × 15 mL). The combined organics were washed with brine (1 × 15 mL), dried over $MgSO_4$ and concentrated under reduced pressure to give a crude residue. Purification by flash silica chromatography (40% EtOAc/Petrol) afforded the title compound (500 mg, 95%) as white needles.

MP: 107-109 °C

***V*max /cm^{-1}(neat):** 3267, 2977, 1719, 1650, 1359, 1150, 770

^{1}H NMR (400 MHz; $CDCl_3$) δ 7.48 (dd, *J* = 7.5, 2.1 Hz, 1H), 7.43 (d, *J* = 6.4 Hz, 1H), 7.35 – 7.27 (m, 2H), 6.32 (s, 1H), 5.87 (s, 1H), 3.20 (s, 3H), 1.43 (s, 9H); **^{13}C NMR (**101 MHz; $CDCl_3$) δ 156.5, 156.3, 138.9, 133.6, 131.1, 130.4, 129.9, 128.7, 81.3, 36.4, 28.2; HRMS m/z (ESI^+) $[C_{13}H_{18}ClN_3O_3Na]^+$ requires$[M + Na]^+$: 322.0934; found: 322.0944

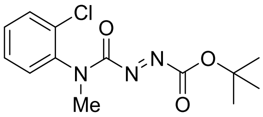

tert-Butyl (*E*)-2-((2-chlorophenyl)(methyl)carbamoyl)diazene-1-carboxylate (5j); A solution of *N*-bromosuccinimide (532 mg, 3.0 mmol, 1.5 equiv) in DCM (13 mL, 0.15 M) was added dropwise to a solution of pyridine (0.364 mL, 4.0 mmol, 2 equiv) and tert-butyl 2-((2-chlorophenyl)(methyl)carbamoyl)hydrazine-1-carboxylate **S5j** (600 mg, 2.0 mmol) in DCM (6.5 mL, 0.30 M) at 0 ºC. The reaction was stirred at RT for 3 h, before being quenched with sat. aq. $NaHCO_3$ (20 mL) and the aqueous layer was extracted with DCM (3 × 15 mL). The combined organics were washed with brine (1 × 15 mL), dried over $MgSO_4$ and concentrated under reduced pressure to give a crude residue. Purification by flash silica chromatography (10% EtOAc/Hexane) afforded the title compound (410 mg, 68%) as an orange oil.

***V_{max}* /cm^{-1}(neat):** 2983, 1761, 1715, 1481, 1370, 1252, 1480

^{1}H NMR (400 MHz, $CDCl_3$) δ 7.42 (ddt, *J* = 5.2, 3.0, 1.1 Hz, 1H), 7.30 – 7.25 (m, 3H), 3.42 (s, 3H), 1.45 (s, 9H); **^{13}C NMR** (101 MHz, CDCl3) δ 160.4, 159.8, 137.9, 132.9, 130.4, 130.1, 130.0, 127.9, 86.3, 37.5, 27.7; **HRMS** (ESI) calcd for $[C_{13}H_{16}ClN_3O_3Na]^+$ requires $[M + Na]^+$ 320.0778, found 320.0779

1.10 General Procedure 6: Synthesis of Silyl enol ether[2]

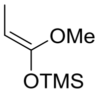

(1-methoxyprop-1-en-1-yl)oxy)trimethylsilane. (2a) The compound was prepared according to a reported procedure.[2] Under nitrogen, a 2.0 M n-BuLi solution in cyclohexane (25 mL, 50

[2] Z. Huang, Z. Liu, J. (S.) Zhou*,* *J. Am. Chem. Soc.*, **2011**, 133, 15882–15885

mmol) in an addition funnel was slowly added to a stirred solution of i-Pr_2NH (7.6 mL, 54.5 mmol) in dry THF (80 mL) at 0 ºC over 20 min. The mixture was stirred at 0 º C for 30 minutes and then it was cooled to -78 ºC in a cooling bath. A solution of methyl propionate (4g, 45 mmol) and TMSCl (6.9 mL, 54 mmol) in dry THF (40 mL) was added slowly over 1.5 hours from the addition funnel. After stirring at -78 ºC for additional 30 minutes, the mixture was slowly warmed up to 25 o C and kept stirred for 18 hours. At the end of the reaction, most of THF was removed by distillation under one atmosphere of argon. The residue was diluted with 80 mL of pentane and the resulting suspension was filtered through a fritted funnel (medium porosity) with pentane washings, to remove LiCl. The filtrate was concentrated, and the crude product was purified by distillation under vacuum which afforded the desired silyl ketene acetal as colorless oil (3.5 g *E:Z* :: 8:1).

^{1}H NMR (400 MHz, $CDCl_3$) δ 3.66 (q, J = 8.0 Hz, 1H), 3.51 (s, 3H), 1.48 (d, *J* = 8.0 Hz, 3H), 0.21 (s, 9H) **^{13}C NMR** (100 MHz, CDCl3) δ 154.0, 78.8, 54.8, 9.5, 0.18; HRMS (ESI) calcd for [$C_7H_{16}O_2SiNa$] requires [M + Na]+ 183.0817, found 183.0821

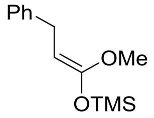

(1-Methoxy-3-phenylprop-1-en-1-yl)oxy)trimethylsilane (**2b**); Under nitrogen, a 2.0 M n-BuLi solution in cyclohexane (25 mL, 50 mmol) in an addition funnel was slowly added to a stirred solution of i-Pr_2NH (7.6 mL, 54.5 mmol) in dry THF (80 mL) at 0 ºC over 20 min. The mixture was stirred at 0 º C for 30 minutes and then it was cooled to -78 ºC in a cooling bath. A solution of methyl propionate (7.3 g, 45 mmol) and TMSCl (6.9 mL, 54 mmol) in dry THF (40 mL) was added slowly over 1.5 hours from the addition funnel. After stirring at -78 ºC for additional 30 minutes, the mixture was slowly warmed up to 25 ºC and kept stirred for 18 hours. At the end of the reaction, most of THF was removed by distillation under one atmosphere of argon. The residue was diluted with 80 mL of pentane and the resulting suspension was filtered through a fritted funnel (medium porosity) with pentane washings, to

remove LiCl. The filtrate was concentrated, under reduced pressure to give silyl ketene acetal as colorless oil (10 g *E:Z* :: 18:1), which was taken on without further purification.

V

V_{max} /cm^{-1}(neat): 2952, 1735, 1435, 1159, 750, 698

^{1}H NMR (400 MHz, $CDCl_3$) δ 7.28–7.22 (m, 4H), 7.19–7.15 (m, 1H), 3.90 (t, *J* = 8.0 Hz, 1H), 3.58 (s, 3H), 3.36 (d, *J* = 8.0 Hz, 2H), 0.27 (s, 9H); **^{13}C NMR** (100 MHz, CDCl3) δ 154.3, 143.0, 128.3, 128.2, 125.6, 83.6, 54.7, 30.9, 0.09; HRMS (ESI) calcd for [$C_{13}H_{20}O_2SiNa$] requires [M + Na]+ 259.1130, found 259.1133.

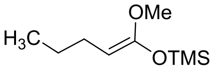

(*E*)-((1-methoxypent-1-en-1-yl)oxy)trimethylsilane (2c); Under nitrogen, a 2.0 M n-BuLi solution in cyclohexane (25 mL, 50 mmol) in an addition funnel was slowly added to a stirred solution of i-Pr_2NH (7.6 mL, 54.5 mmol) in dry THF (80 mL) at 0 ^oC over 20 min. The mixture was stirred at 0 ^{o}C for 30 minutes and then it was cooled to -78 ^oC in a cooling bath. A solution of methyl pentanoate (5.2 g, 45 mmol) and TMSCl (7.3 mL, 54 mmol) in dry THF (40 mL) was added slowly over 1.5 hours from the addition funnel. After stirring at -78 ^oC for additional 30 minutes, the mixture was slowly warmed up to 25 ^oC and kept stirred for 18 hours. At the end of the reaction, most of THF was removed by distillation under one atmosphere of argon. The residue was diluted with 80 mL of pentane and the resulting suspension was filtered through a fritted funnel (medium porosity) with pentane washings, to remove LiCl. The filtrate was concentrated, under reduced pressure to give silyl ketene acetal as colorless oil (7.4 g, *E:Z* :: 19:1), which was taken on without further purification.

V_{max} /cm^{-1}(neat): 2958, 1709, 1549, 1395, 1259, 1057, 772

^{1}H NMR (400 MHz, $CDCl_3$) δ 3.64 (t, *J* = 7.3 Hz, 1H), 3.47 (s, 3H), 1.89 (q, *J* = 7.3 Hz, 2H), 1.31 – 1.24 (m, 2H), 0.85 (t, *J* = 7.4 Hz, 3H), 0.19 (s, 9H); **^{13}C NMR** (100 MHz, CDCl3) δ 153.7, 85.1, 54.8, 26.6, 23.8, 13.6, -0.3; HRMS (ESI) calcd for [$C_9H_{20}O_2SiNa$] requires [M + Na]+ 211.1130, found 211.1135

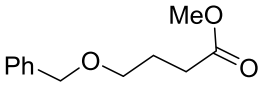

methyl 4-(benzyloxy)butanoate (S2d); To a solution of 4-(benzyloxy)butanoic acid (5 g, 25.7 mmol) in methanol (0.1M) was added conc. HCl (cat.) and the solution heated at 60 °C for 8h. The solution was allowed to cool and concentrated under reduced pressure. The colourless oil was dissolved in dichloromethane (20 ml) and washed with water and brine solution and the combined organic extracts were dried over magnesium sulphate and concentrated under reduced pressure to provide the methyl 4-(benzyloxy)butanoate (5.89 g) as a colourless oil.

V_{max} /cm^{-1}(neat): 2950, 2857, 1733, 1436, 1169, 1103, 1058, 735.

^{1}H NMR (400 MHz, $CDCl_3$) δ 7.27 – 7.20 (m, 4H), 7.20 – 7.15 (m, 1H), 4.39 (s, 2H), 3.55 (s, 3H), 3.41 (t, J = 6.2 Hz, 2H), 2.34 (t, J = 7.4 Hz, 2H), 1.84 (ddd, J = 13.6, 7.4, 6.2 Hz, 2H); **^{13}C NMR** (101 MHz, CDCl3) δ 173.8, 138.4, 128.3, 127.5, 127.5, 72.8, 69.1, 51.4, 30.8, 25.1; HRMS (ESI) calcd for $[C_{12}H_{16}O_3Na]^+$ requires $[M + Na]^+$ 231.0997, found 231.0993

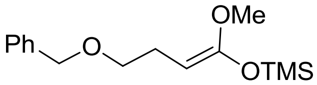

(4-(benzyloxy)-1-methoxybut-1-en-1-yl)oxy)trimethylsilane (2d) Under nitrogen, 2.0 M n-BuLi solution in cyclohexane (19 mL, 38 mmol) in an addition funnel was slowly added to a stirred solution of i-Pr_2NH (5.7 mL, 40.85 mmol) in dry THF (60 mL) at 0 °C over 20 min. The mixture was stirred at 0 ° C for 30 minutes and then it was cooled to -78 o C in a cooling bath. A solution of methyl pentanoate (5.89 g, 28 mmol) and TMSCl (5.12 mL, 37.8 mmol) in dry THF (30 mL) was added slowly over 1.5 hours from the addition funnel. After stirring at -78 °C for additional 30 minutes, the mixture was slowly warmed up to 25 °C and kept stirred for 24 hours. At the end of the reaction, most of THF was removed by distillation under one atmosphere of argon. The residue was diluted with 50 mL of pentane and the resulting suspension was filtered through a fritted funnel (medium porosity) with pentane washings, to remove LiCl. The filtrate was concentrated, under reduced pressure to give silyl ketene acetal as colorless oil (8.1 g, *E:Z* 10:1), which was taken on without further purification.

V_{max} **/cm^{-1}(neat):** 2955, 2853, 1697, 1252, 1081

^{1}H NMR (400 MHz, $CDCl_3$) δ 7.37 – 7.32 (m, 4H), 7.29 – 7.24 (m, 1H), 4.52 (s, 2H), 3.72 (t, *J* = 7.3 Hz, 1H), 3.52 (s, 3H), 3.44 (t, *J* = 7.0 Hz, 2H), 2.31 (q, *J* = 7.1 Hz, 2H), 0.25 (s, 9H).

^{13}C NMR (100 MHz, CDCl3) δ154.5, 138.9, 128.3, 127.6, 127.4, 80.4, 72.7, 71.1, 54.6, 25.3, -0.18.; HRMS (ESI) calcd for [$C_{15}H_{24}O_2SiNa$] requires [M + Na]+ 303.1392, found 303.1382

1.11 X-ray crystallography:

X-ray studies of 4b:

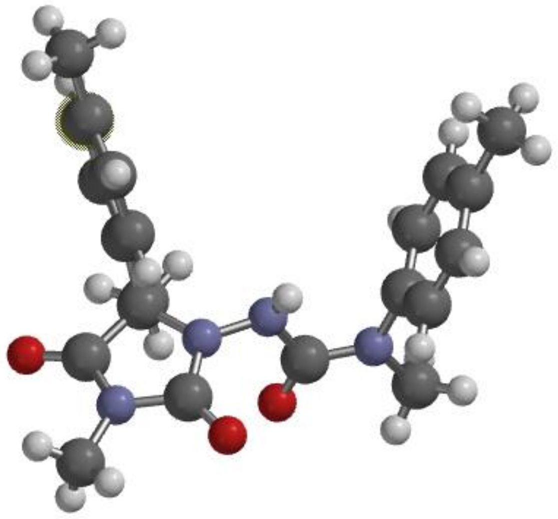

Bond precision:	C-C = 0.0020 A	
Wavelength	0.71073	
Cell:	a=22.4783(5)	α=90
	b=11.4618(2)	β=92.056(2)
	c=15.6035(4)	γ=90
Volume	4017.53(15)	
Space group	C 2/c	
Hall group	-C 2yc	
Moiety formula	C21 H24 N4 O3	
Sum formula	C21 H24 N4 O3	
Dx,g cm-3	1.258	
h,k,lmax	29,15,20	
Tmin,Tmax	0.970,0.977	
Tmin’	0.953	

Data completeness	0.998
Theta(max)	27.949
R(reflections)	0.0430(3669)
wR2(reflections)	0.1129(4827)

X-ray studies of 7b:

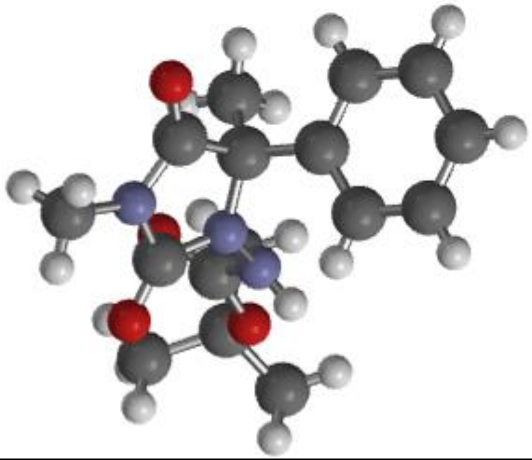

Bond precision:	C-C = 0.0018 A	
Wavelength	0.71073	
Cell:	a = 21.1353(4)	α=90
	b = 9.1587(2)	β=92.271(1)
	c = 16.9027 (3)	γ=90
Volume	3269.32(11)	
Space group	P 21/c	
Hall group	-P 2ybc	
Moiety formula	C16 H21 N3 O4	
Sum formula	C16 H21 N3 O4	
Dx,g cm-3	1.298	
h,k,lmax	27,12,22	
Tmin,Tmax	0.964,0.976	
Tmin’	0.951	
Data completeness	0.998	
Theta(max)	27.952	
R(reflections)	0.0376(6284)	
wR2(reflections)	0.0932(7838)	

1.12 ReactIR studies

(a)

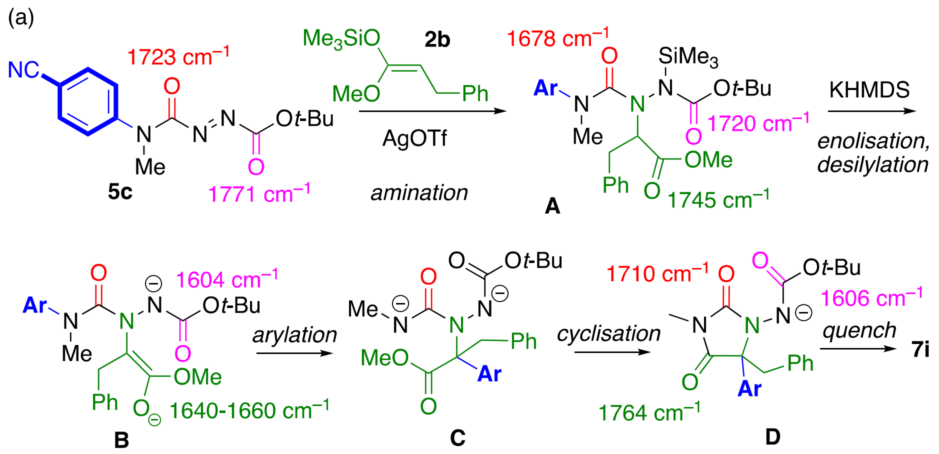

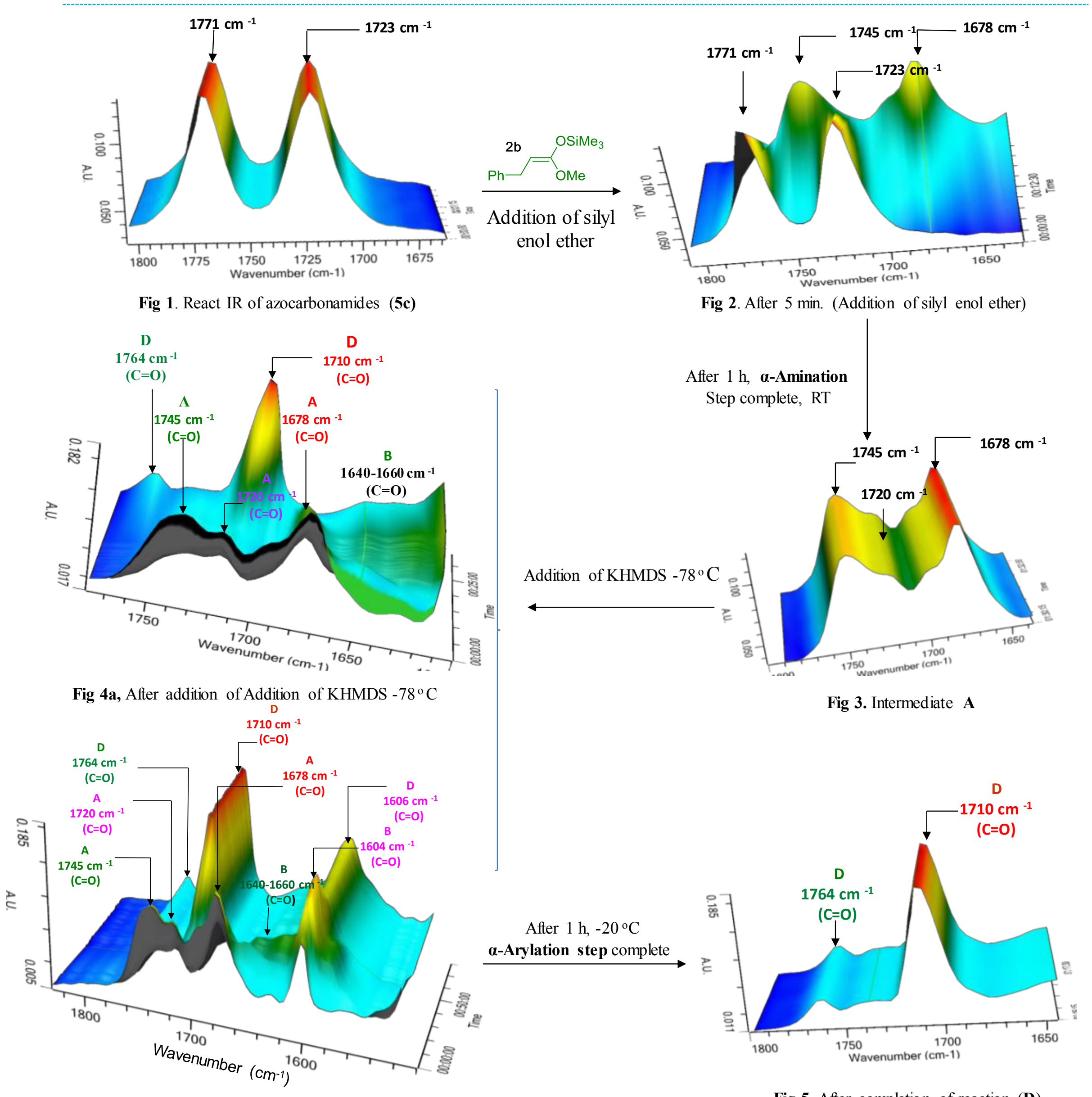

Fig 1. React IR of azocarbonamides (**5c**)

Fig 2. After 5 min. (Addition of silyl enol ether)

Fig 3. Intermediate **A**

Fig 4a, After addition of Addition of KHMDS -78 °C

Fig 4b, After addition of Addition of KHMDS -78 °C

Fig 5, After completion of reaction (**D**)

1.13 Procedures and Analytical data of hydantoin formation from symmetrical azodicarboxamides

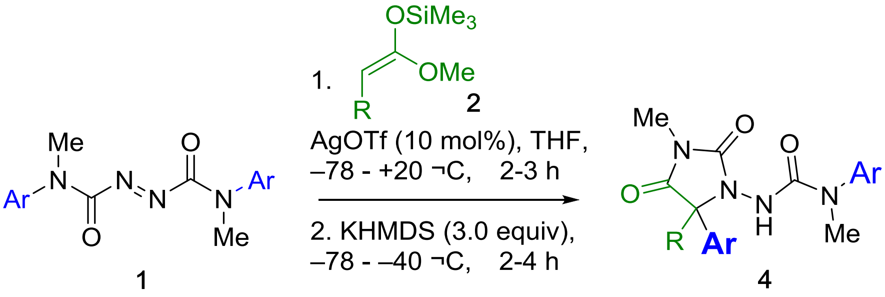

Silyl enol ether **2** (1 equiv.,) was added dropwise to a mixture of symmetric azocarboxamide compound **1** (1 equiv) and AgOTf (10 mol %.) in THF (0.1 M) at -78 °C. The reaction was warmed to RT after 30 minutes and stirred at RT for 3 h. After TLC showed consumption of symmetric azocarboxamide compound **1**, the reaction was cooled to -78 °C and KHMDS (1 M in THF, 3 equiv) was added dropwise. After 20 minutes the reaction was warmed to -40 °C and stirred at -40 °C for 4 h. The reaction was quenched with sat. aq. NH_4Cl (15 mL), and the aqueous layer was extracted with EtOAc (3 × 10 mL). The combined organics were washed with brine (1 × 10 mL), dried over $MgSO_4$ and concentrated under reduced pressure to give a crude residue. Purification by flash silica chromatography desired compound.

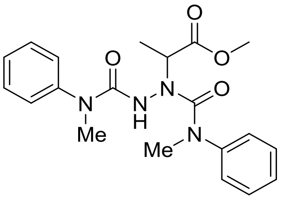

***V*max /cm^{-1}(neat):** 2950, 1739, 1687, 1659

Methyl *N*-(methyl(phenyl)carbamoyl)-*N*-(3-methyl-3-phenylureido)alaninate (3) (Semi-solid), **^{1}H NMR** (400 MHz, $CDCl_3$) δ 7.36 – 7.20 (m, 7H), 7.14 (t, *J* = 7.3 Hz, 1H), 6.91 (d, *J* = 7.6 Hz, 2H), 5.82 (s, 1H), 4.75 (s, 1H), 3.56 (s, 3H), 3.26 (s, 3H), 3.13 (s, 3H), 1.23 (d, *J* = 7.3 Hz, 3H); **^{13}C NMR** (100 MHz, CDCl3) δ 173.1, 160.0, 155.6, 145.4, 142.0, 129.8, 129.6, 127.6, 126.9,

125.3, 124.2, 57.5, 52.1, 39.8, 37.5, 14.3; HRMS (ESI) calcd for $[C_{20}H_{24}N_4O_4Na]^+$ requires $[M + Na]^+$ 407.1690, found 407.1690.

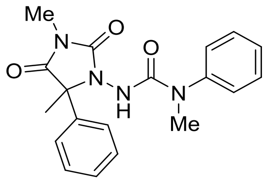

3-(3,5-dimethyl-2,4-dioxo-5-phenylimidazolidin-1-yl)-1-methyl-1-phenylurea (4a), Methoxy-1-trimethylsilyloxypropene (60 mg, 0.37 mmol,) was added dropwise to a mixture of (*E*)-2-benzoyl-N-methyl-N-phenyldiazene-1-carboxamide **1a** (101 mg, 0.37 mmol) and AgOTf (8.5 mg, 10 mol %.) in THF (3.4 mL, 0.1 M) at -78 ^oC. The reaction was warmed to RT after 30 minutes and stirred at RT for 3 h. After TLC showed consumption of (*E*)-2-benzoyl-N-methyl-N-phenyldiazene-1-carboxamide **1a**, the reaction was cooled to -78 ^oC and KHMDS (1 M in THF, 3 equiv) was added dropwise. After 20 minutes the reaction was warmed to -40 ^oC and stirred at -40 ^oC for 4 h. The reaction was quenched with sat. aq. NH_4Cl (15 mL), and the aqueous layer was extracted with EtOAc (3 × 10 mL). The combined organics were washed with brine (1 × 10 mL), dried over $MgSO_4$ and concentrated under reduced pressure to give a crude residue. Purification by flash silica chromatography (60% EtOAC/Hexane) afforded the title compound (91 mg, 75%) as colourless semi-solid.

$\boldsymbol{V_{max}}$ **/cm^{-1}(neat):** 3295, 1786, 1721, 1698

^{1}H NMR (400 MHz, $CDCl_3$) δ 7.42 – 7.35 (m, 2H), 7.35 – 7.27 (m, 6H), 7.27 – 7.23 (m, 2H), 5.99 (s, 1H), 3.30 (s, 3H), 3.10 (s, 3H), 1.91 (s, 3H);**^{13}C NMR** (100 MHz, CDCl3) δ 173.6, 155.7, 141.8, 137.2, 130.3, 129.0, 128.7, 128.2, 127.1, 125.9, 68.7, 38.2, 25.3, 20.1; HRMS (ESI) calcd for $[C_{19}H_{20}N_4O_3Na]^+$ requires $[M + Na]^+$ 375.1428, found 375.1430.

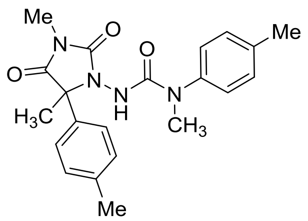

3-(3,5-Dimethyl-2,4-dioxo-5-(p-tolyl)imidazolidin-1-yl)-1-methyl-1-(p-tolyl)urea (4b); 1-Methoxy-1-trimethylsilyloxypropene (74 mg, 0.46 mmol,) was added dropwise to a mixture of (*E*)-N[1],N[2]-dimethyl-N[1]-phenyl-N[2]-(*p*-tolyl)diazene-1,2-dicarboxamide **1b** (150 mg, 0.46 mmol) and AgOTf (11.6 mg, 10 mol %.) in THF (4.6 mL, 0.1 M) at -78 °C. The reaction was warmed to RT after 30 minutes and stirred at RT for 3 h. After TLC showed consumption of (E)-N[1],N[2]-dimethyl-N[1]-phenyl-N[2]-(*p*-tolyl)diazene-1,2-dicarboxamide **1b**, the reaction was cooled to -78 °C and KHMDS (1 M in THF, 3 equiv) was added dropwise. After 20 minutes the reaction was warmed to -40 °C and stirred at -40 °C for 4 h. The reaction was quenched with sat. aq. NH_4Cl (15 mL), and the aqueous layer was extracted with EtOAc (3 × 10 mL). The combined organics were washed with brine (1 × 10 mL), dried over $MgSO_4$ and concentrated under reduced pressure to give a crude residue. Purification by flash silica chromatography (60% EtOAC/Hexane) afforded the title compound (140 mg, 80%) as colourless needles.

MP: 166-168°C

***V*max /cm^{-1}(neat):** 3298, 2942, 1783, 1717, 1690, 1513, 1457, 824, 753

^{1}H NMR (400 MHz, $CDCl_3$) δ 7.15 (s, 4H), 7.11 (s, 4H), 5.98 (br s, 1H) 3.25 (s, 3H), 3.07 (s, 3H), 2.33 (s, 3H), 2.29 (s, 3H), 1.87 (s, 3H); **^{13}C NMR** (100 MHz, CDCl3) δ 173.9, 155.9, 139.2, 138.6, 138.3, 134.3, 130.9, 129.7, 126.9, 125.8, 68.5, 38.2, 25.2, 21.1, 21.0, 20.0; HRMS (ESI) calcd for [$C_{21}H_{24}N_4O_3Na$] requires [M + Na]+ 403.1746, found 403.1743.

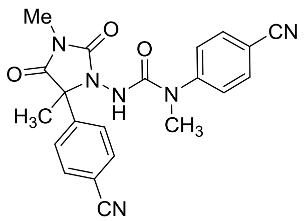

1-(4-Cyanophenyl)-3-(5-(4-cyanophenyl)-3,5-dimethyl-2,4-dioxoimidazolidin-1-yl)-1-methylurea (4c); 1-Methoxy-1-trimethylsilyloxypropene (48 mg, 0.30 mmol,) was added dropwise to a mixture of (*E*)-N[1],N[2]-bis(4-cyanophenyl)-N[1],N[2]-dimethyldiazene-1,2-dicarboxamide **1c** (104 mg, 0.30 mmol) and AgOTf (7.5 mg, 10 mol %.) in THF (3 mL, 0.1 M) at -78 ^{o}C. The reaction was warmed to RT after 30 minutes and stirred at RT for 3 h. After TLC showed consumption of (*E*)-N[1],N[2]-bis(4-cyanophenyl)-N[1],N[2]-dimethyldiazene-1,2-dicarboxamide **1c**, the reaction was cooled to -78 ^{o}C and KHMDS (1 M in THF, 3 equiv) was added dropwise. After 20 minutes the reaction was warmed to -40 ^{o}C and stirred at -40 ^{o}C for 4 h. The reaction was quenched with sat. aq. NH_4Cl (10 mL), and the aqueous layer was extracted with EtOAc (3 × 10 mL). The combined organics were washed with brine (1 × 10 mL), dried over $MgSO_4$ and concentrated under reduced pressure to give a crude residue. Purification by flash silica chromatography (1% to 10% (10% MeOH in DCM)/DCM) afforded the title compound (95 mg, 79%) as a white semi-solid.

***V*max /cm^{-1}(neat):** 3299, 3018, 2218, 1786, 1719, 1688, 1505, 1338, 844, 748

^{1}H NMR (400 MHz, $CDCl_3$) δ 7.65–7.62 (m, 1H), 7.60–7.57 (m, 3H), 7.56–7.53 (m, 1H), 7.45–7.41 (m, 3H), 6.27 (br s, 1H), 2.94 (s, 3H), 3.04 (s, 3H), 1.83 (s, 3H); **^{13}C NMR** (100 MHz, CDCl3) δ 172.1, 155.6, 155.2, 148.8, 145.2, 134.0, 133.6, 132.6, 127.2, 127.0, 126.5, 118.0, 117.7, 112.8, 111.3, 68.4, 38.0, 25.5, 20.3; HRMS (ESI) calcd for [$C_{21}H_{18}N_6O_3Na$] requires [M + Na]+ 425.1338, found 425.1334.

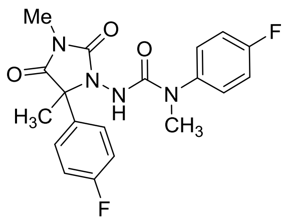

1-(4-Fluorophenyl)-3-(5-(4-fluorophenyl)-3,5-dimethyl-2,4-dioxoimidazolidin-1-yl)-1-methylurea (4d); 1-Methoxy-1-trimethylsilyloxypropene (48 mg, 0.30 mmol,) was added dropwise to a mixture of (*E*)-N^1,N^2-bis(4-fluorophenyl)-N^1,N^2-dimethyldiazene-1,2-dicarboxamide **1d** (100 mg, 0.30 mmol) and AgOTf (7.5 mg, 10 mol %.) in THF (3.0 mL, 0.1 M) at -78 ^oC. The reaction was warmed to RT after 15 minutes, and stirred at RT for 3 h. After TLC showed consumption of (*E*)-N^1,N^2-bis(4-fluorophenyl)-N^1,N^2-dimethyldiazene-1,2-dicarboxamide **1d**, the reaction was cooled to -78 ^oC and KHMDS (1 M in THF, 3 equiv) was added dropwise. After 20 minutes the reaction was warmed to -40 ^oC and stirred at -40 ^oC for 4 h. The reaction was quenched with sat. aq. NH_4Cl (10 mL), and the aqueous layer was extracted with EtOAc (3 × 10 mL). The combined organics were washed with brine (1 × 10 mL), dried over $MgSO_4$ and concentrated under reduced pressure to give a crude residue. Purification by flash silica chromatography (50% to 70% EtOAC + Hexane) afforded the *title compound* (44 mg, 38%) as colourless needles.

MP: 130-132 ^oC

***V*$_{max}$ /cm^{-1}(neat):** 3299, 2988, 1784, 1716, 1683, 1508, 1452, 1222, 840, 751

^{1}H NMR (400 MHz, $CDCl_3$) δ 7.29–7.22 (m, 4H), 7.09–7.05 (m, 2H), 7.01–6.97 (m, 2H), 5.94 (br s, 1H), 3.24 (s, 3H), 3.07 (s, 3H), 1.84 (s, 3H); **^{13}C NMR** (100 MHz, CDCl3) *δ* 173.5, 163.5 (d, $J_{C\text{-}F}$ = 249.2 Hz), 162.3 (d, $J_{C\text{-}F}$ = 249.2 Hz), 155.7, 155.6, 137.7 (d, $J_{C\text{-}F}$ = 3.0 Hz), 133.0(d, $J_{C\text{-}F}$ = 3.0 Hz), 129.2 (d, $J_{C\text{-}F}$ = 9.0 Hz) 128.0 (d, $J_{C\text{-}F}$ = 9.0 Hz), 117.4 (d, $J_{C\text{-}F}$ = 23.2 Hz), 115.9 (d, $J_{C\text{-}F}$ = 23.2 Hz), 68.2, 38.4, 25.3, 20.4; HRMS (ESI) calcd for [$C_{19}H_{18}F_2N_4O_3Na$] requires [M + Na]+ 411.1245, found 411.1256.

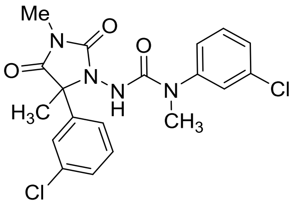

1-(3-Chlorophenyl)-3-(5-(3-chlorophenyl)-3,5-dimethyl-2,4-dioxoimidazolidin-1-yl)-1-methylurea (4e); 1-Methoxy-1-trimethylsilyloxypropene (45 mg, 0.28 mmol,) was added dropwise to a mixture of (*E*)-N^1,N^2-bis(3-chlorophenyl)-N^1,N^2-dimethyldiazene-1,2-dicarboxamide **1e** (95 mg, 0.26 mmol) and AgOTf (6.5 mg, 10 mol %.) in THF (2.6 mL, 0.1 M) at -78 °C. The reaction was warmed to RT after 30 minutes and stirred at RT for 4 h. After TLC showed consumption of (*E*)-N^1,N^2-bis(3-chlorophenyl)-N^1,N^2-dimethyldiazene-1,2-dicarboxamide **1e**, the reaction was cooled to -78 °C and KHMDS (1 M in THF, 3 equiv) was added dropwise. After 20 minutes the reaction was warmed to -40 °C and stirred at -40 °C for 5 h. The reaction was quenched with sat. aq. NH_4Cl (10 mL), and the aqueous layer was extracted with EtOAc (3 × 10 mL). The combined organics were washed with brine (1 × 10 mL), dried over $MgSO_4$ and concentrated under reduced pressure to give a crude residue. Purification by flash silica chromatography (60% EtOAC/Hexane) afforded the title compound (97 mg, 89%) as a pale-yellow oil.

V $\boldsymbol{V_{max}}$ **/cm^{-1}(neat):** 3287, 2942, 1785, 1718, 1689, 1591, 1470, 748, 695

^{1}H NMR (400 MHz, $CDCl_3$) δ 7.30–7.29 (m, 2H), 7.27–7.25 (m, 2H), 7.21–7.19 (m, 2H), 7.16–7.13 (,. 2H), 6.10 (br s, 1H), 3.26 (s, 3H), 3.07 (s, 3H), 1.85 (s, 3H); **^{13}C NMR** (100 MHz, CDCl3) δ 173.02, 155.5, 155.4, 145.4, 143.0, 139.2, 135.8, 131.3, 130.3, 129.0, 128.6, 127.5, 126.4, 125.3, 124.3, 68.3, 38.2, 25.4, 20.1; **HRMS** (ESI) calcd for [$C_{19}H_{18}Cl_2N_4O_3Na$] requires [M + Na]+ 443.0654, found 443.0644.

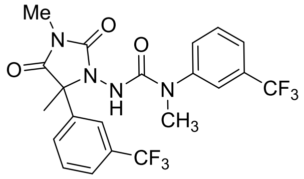

3-(3,5-Dimethyl-2,4-dioxo-5-(3-(trifluoromethyl)phenyl)imidazolidin-1-yl)-1-methyl-1-(3-(trifluoromethyl)phenyl)urea (4f); (1-methoxyprop-1-en-1-yl)oxy) trimethylsilane (33 mg, 0.20 mmol,) was added dropwise to a mixture of (E)-N^1,N^2-dimethyl-N^1,N^2-bis(3-(trifluoromethyl)phenyl)diazene-1,2-dicarboxamide **1f** (100 mg, 0.23 mmol) and AgOTf (5.8 mg, 10 mol %.) in THF (2.3 mL, 0.1 M) at -78 ^oC. The reaction was warmed to RT after 15 minutes, and stirred at RT for 3 h. After TLC showed consumption of (*E*)-N^1,N^2-dimethyl-N^1,N^2-bis(3-(trifluoromethyl)phenyl)diazene-1,2-dicarboxamide **1f** the reaction was cooled to -78 ^oC and KHMDS (1 M in THF, 3 equiv) was added dropwise. After 20 minutes the reaction was warmed to -40 ^oC and stirred at -40 ^oC for 3 h. The reaction was quenched with sat. aq. NH_4Cl (10 mL), and the aqueous layer was extracted with EtOAc (3 × 10 mL). The combined organics were washed with brine (1 × 10 mL), dried over $MgSO_4$ and concentrated under reduced pressure to give a crude residue. Purification by flash silica chromatography (50% to 70% EtOAC + Hexane) afforded the *title compound* (85 mg, 75%) as a colourless oil.

V_{max} **/cm^{-1}(neat):** 3284, 2987, 1786, 1718, 1449, 1120, 735, 698

^{1}H NMR (400 MHz, $CDCl_3$) δ 7.57–7.50 (m, 7H), 7.48–7.44 (m, 1H), 6.17 (br s, 1H), 3.30 (s 3H), 3.08 (s, 3H), 1.89 (s, 3H); **^{13}C NMR** (100 MHz, CDCl3) 172.9, 155.6, 155.4, 142.5, 138.4, 132.9, 132.6, 131.5, 131.2, 130.9, 130.4 (q, $J_{C\text{-}F}$ = 2.2 Hz), 129.6 (q, $J_{C\text{-}F}$ = 2.0 Hz), 129.60, 125.7 (q, $J_{C\text{-}F}$ = 3.8 Hz), 124.8 (q, $J_{C\text{-}F}$ = 3.7 Hz), 123.9 (q, $J_{C\text{-}F}$ = 3.8 Hz), 122.8 (q, $J_{C\text{-}F}$ = 3.9 Hz), 68.3, 25.4, 20.6; HRMS (ESI) calcd for [$C_{21}H_{18}F_6N_4O_3Na$] requires [M + Na]+ 511.1181, found 511.1150.

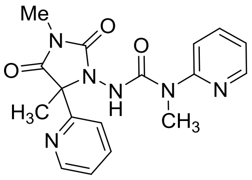

3-(3,5-Dimethyl-2,4-dioxo-5-(pyridin-2-yl)imidazolidin-1-yl)-1-methyl-1-(pyridin-2-yl) urea (4g); 1-Methoxy-1-trimethylsilyloxypropene (59 mg, 0.36 mmol,) was added dropwise to a mixture of (*E*)-N[1],N[2]-dimethyl-N[1],N[2]-di(pyridin-2-yl)diazene-1,2-dicarboxamide **1g** (100 mg, 0.33 mmol) and AgOTf (8.4 mg, 10 mol %.) in THF (3.3 mL, 0.1 M) at -78 ^oC. The reaction was warmed to RT after 30 minutes, and stirred at RT for 4 h. After TLC showed consumption of (*E*)-N[1],N[2]-dimethyl-N[1],N[2]-di(pyridin-2-yl)diazene-1,2-dicarboxamide **1g**, the reaction was cooled to -78 ^oC and KHMDS (1 M in THF, 3 equiv) was added dropwise. After 20 minutes the reaction was warmed to -40 ^oC and stirred at -40 ^oC for 4 h. The reaction was quenched with sat. aq. NH_4Cl (10 mL), and the aqueous layer was extracted with EtOAc (3 × 10 mL). The combined organics were washed with brine (1 × 10 mL), dried over $MgSO_4$ and concentrated under reduced pressure to give a crude residue. Purification by flash silica chromatography (10% MeOH/DCM)) afforded the *title compound* (87 mg, 73%) as white needles.

MP: 179-181 ^oC

***V*max /cm^{-1}(neat):** 3264, 1786, 1716, 1687, 1434, 742

^{1}H NMR (400 MHz, $CDCl_3$) δ 12.09 (br s, 1H), 8.62–8.60 (m, 1H), 8.08–8.06 (m, 1H), 7.74–7.65 (m, 2H), 7.56–7.53 (m, 1H), 7.28–7.34 (m, 1H), 6.97–6.90 (m, 2H), 3.38 (s, 3H), 3.07 (s, 3H), 1.93 (s, 3H); **^{13}C NMR** (100 MHz, CDCl3) δ 172.8, 156.8, 156.4, 155.8, 155.0, 149.2, 145.8, 139.0, 137.4, 123.5, 121.8, 117.8, 111.8, 70.4, 33.2, 25.3, 19.9; HRMS (ESI) calcd for $[C_{17}H_{18}N_6O_3Na]$ requires [M + Na]+ 377.1338, found 377.1320.

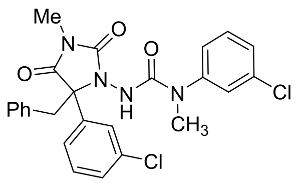

3-(5-Benzyl-5-(3-chlorophenyl)-3-methyl-2,4-dioxoimidazolidin-1-yl)-1-(3-chlorophenyl) -1-methylure (4h); (1-methoxy-3-phenylprop-1-en-1-yl)oxy)trimethylsilane (58 mg, 0.24 mmol,) was added dropwise to a mixture of (*E*)-N^1,N^2-bis(3-chlorophenyl)-N^1,N^2-dimethyldiazene-1,2-dicarboxamide **1e** (100 mg, 0.27 mmol) and AgOTf (6.9 mg, 10 mol %.) in THF (2.7 mL, 0.1 M) at -78 ºC. The reaction was warmed to RT after 15 minutes, and stirred at RT for 3 h. After TLC showed consumption of (*E*)-N^1,N^2-bis(3-chlorophenyl)-N^1,N^2-dimethyldiazene-1,2-dicarboxamide **1e**, the reaction was cooled to -78 ºC and KHMDS (1 M in THF, 3 equiv) was added dropwise. After 20 minutes the reaction was warmed to -40 ºC and stirred at -40 ºC for 3 h. The reaction was quenched with sat. aq. NH_4Cl (10 mL), and the aqueous layer was extracted with EtOAc (3 × 10 mL). The combined organics were washed with brine (1 × 10 mL), dried over $MgSO_4$ and concentrated under reduced pressure to give a crude residue. Purification by flash silica chromatography (50% to 70% EtOAC + Hexane) afforded the title compound (85 mg, 62%) as a colourless oil.

V_{max} /cm^{-1}(neat): 3333, 2983, 1788, 1711, 1590, 1452, 733, 698

^{1}H NMR (400 MHz, $CDCl_3$) δ 7.82 (d, *J* = 2.0 Hz, 1H), 7.76–7.73 (m, 1H), 7.44–7.43 (m, 2H), 7.36–7.35 (m, 1H), 7.31–7.30 (m, 1H), 7.21–7.16 (m, 3H), 7.07–7.03 (m, 2H) 6.97 (d, *J* = 8.0 Hz, 2H), 5.85 (br s, 1H), 3.27 (d, *J* = 12.0 Hz, 1H), 3.24 (s, 3H), 3.14 (d, *J* = 12.0 Hz, 1H), 3.00 (s, 3H); **^{13}C NMR** (100 MHz, CDCl3) 172.1, 155.9, 155.4, 143.3, 138.03, 135.9, 134.7, 133.8, 131.5, 130.1, 129.2, 129.1, 128.7, 128.6, 128.2, 128.1, 127.3, 125.9, 125.3, 72.7, 41.3, 38.0, 25.0; HRMS (ESI) calcd for [$C_{25}H_{22}Cl_2N_4O_3Na$] requires [M + Na]+ 519.0967, found 519.0963.

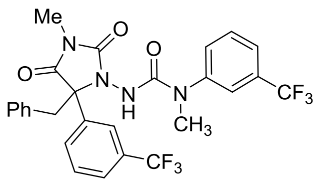

3-(5-Benzyl-3-methyl-2,4-dioxo-5-(3-(trifluoromethyl)phenyl)imidazolidin-1-yl)-1-methyl-1-(3-(trifluoromethyl)phenyl)urea (4i); (1-methoxy-3-phenylprop-1-en-1-yl)oxy)trimethylsilane (49 mg, 0.20 mmol,) was added dropwise to a mixture of (*E*)-N[1],N[2]-dimethyl-N[1],N[2]-bis(3-(trifluoromethyl)phenyl)diazene-1,2-dicarboxamide **1f** (100 mg, 0.23 mmol) and AgOTf (5.8 mg, 10 mol %) in THF (2.3 mL, 0.1 M) at -78 °C. The reaction was warmed to RT after 10 minutes, and stirred at RT for 3 h. After TLC showed consumption of (*E*)-N[1],N[2]-dimethyl-N[1],N[2]-bis(3-(trifluoromethyl)phenyl)diazene-1,2-dicarboxamide **1f**, the reaction was cooled to -78 °C and KHMDS (1 M in THF, 3 equiv) was added dropwise. After 20 minutes the reaction was warmed to -40 °C and stirred at -40 °C for 3 h. The reaction was quenched with sat. aq. NH_4Cl (10 mL), and the aqueous layer was extracted with EtOAc (3 × 10 mL). The combined organics were washed with brine (1 × 10 mL), dried over $MgSO_4$ and concentrated under reduced pressure to give a crude residue. Purification by flash silica chromatography (50% to 70% EtOAC + Hexane) afforded the *title compound* (107 mg, 82%) as a colourless oil.

***V_{max}* /cm^{-1}(neat):** 3260, 1787, 1720, 1447, 1328, 1126, 748

^{1}H NMR (400 MHz, $CDCl_3$) δ 8.07 (d, *J* = 8.0 Hz, 2H), 7.72 (d, *J* = 8.4 Hz, 1H), 7.65–7.61 (m, 2H), 7.58–7.54 (m, 2H), 7.49 (d, *J* = 8.3 Hz, 1H), 7.17–7.13 (m, 1H), 7.01–6.97 (m, 2H), 6.65 (d, *J* = 7.2 Hz, 2H), 5.82 (s, 1H), 3.76 (d, *J* = 14.0 Hz, 1H), 3.26 (s, 3H), 3.16 (d, *J* = 14.0 Hz, 1H), 2.88 (s, 3H); **^{13}C NMR** (100 MHz, CDCl3) δ 172.0, 155.8, 155.4, 142.8, 137.1, 133.7, 131.2, 131.1 (q, $J_{C\text{-}F}$ = 1.0 Hz), 130.7 (q, $J_{C\text{-}F}$ = 1.3 Hz), 129.4, 129.2, 128.5, 128.2, 125.8 (q, $J_{C\text{-}F}$ = 3.8 Hz), 125.1 (q, $J_{C\text{-}F}$ = 3.7 Hz), 124.7 (q, $J_{C\text{-}F}$ = 3.7 Hz), 123.9 (q, $J_{C\text{-}F}$ = 4.0 Hz), 72.7, 41.5, 38.1, 25.1; HRMS (ESI) calcd for [$C_{27}H_{22}F_6N_4O_3Na$] requires [M + Na]+ 587.1494, found 587.1475.

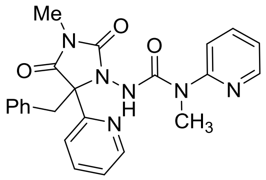

3-(5-Benzyl-3-methyl-2,4-dioxo-5-(pyridin-2-yl)imidazolidin-1-yl)-1-methyl-1-(pyridin-2-yl)urea (4j); (1-methoxy-3-phenylprop-1-en-1-yl)oxy)trimethylsilane (71 mg, 0.30 mmol,) was added dropwise to a mixture of (*E*)-N^1,N^2-dimethyl-N^1,N^2-di(pyridin-2-yl)diazene-1,2-dicarboxamide **1g** (100 mg, 0.33 mmol) and AgOTf (5.8 mg, 8.4 mol %) in THF (3.3 mL, 0.1 M) at -78 °C. The reaction was warmed to RT after 10 minutes, and stirred at RT for 3 h. After TLC showed consumption of (*E*)-N^1,N^2-dimethyl-N^1,N^2-di(pyridin-2-yl)diazene-1,2-dicarboxamide **1g**, the reaction was cooled to -78 °C and KHMDS (1 M in THF, 3 equiv) was added dropwise. After 10 minutes the reaction was warmed to -20 °C and stirred at -40 °C for 3 h. The reaction was quenched with sat. aq. NH_4Cl (10 mL), and the aqueous layer was extracted with EtOAc (3 × 10 mL). The combined organics were washed with brine (1 × 10 mL), dried over $MgSO_4$ and concentrated under reduced pressure to give a crude residue. Purification by flash silica chromatography (50% to 70% EtOAC + Hexane) afforded the *title compound* (95 mg, 65%) as a colourless semi-solid.

***V_{max}* /cm^{-1}(neat):** 3234, 2989, 1787, 1718, 1690, 1593, 1434, 731, 699

^{1}H NMR (400 MHz, $CDCl_3$) δ 12.23 (s, 1H), 8.64 – 8.60 (m, 1H), 8.22 (dd, *J* = 5.2, 1.5 Hz, 1H), 7.75 – 7.72 (m, 2H), 7.40 (d, *J* = 7.0 Hz, 2H), 7.29 – 7.24 (m, 5H), 6.99 (dd, *J* = 7.3, 5.0 Hz, 1H), 6.95 (d, *J* = 8.6 Hz, 1H), 3.82 (d, *J* = 14.2 Hz, 1H), 3.70 (d, *J* = 14.2 Hz, 1H), 3.31 (s, 3H), 2.84 (s, 3H). **^{13}C NMR** (101 MHz, $CDCl_3$) δ 171.8, 156.4, 155.5, 155.0, 154.8, 149.0, 145.5, 139.2, 138.1, 137.0, 134.5 130.3, 127.5, 128.48, 123.7, 122.8, 117.9, 111.9, 74.6, 38.4, 33.3, 24.9; HRMS (ESI) calcd for [$C_{23}H_{22}N_6O_3Na$] requires [M + Na]$^+$ 453.1651, found 453.1647.

1.13General Procedure 7: A general, connective synthesis of protected *N*-aminohydantoins **(7):**

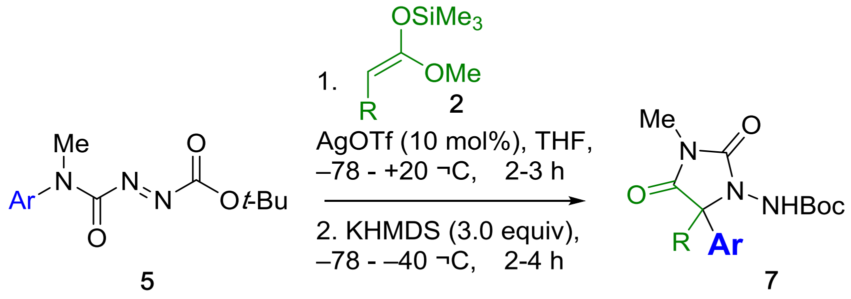

Silyl enol ether **2** (1 equiv,) was added dropwise to a mixture of azocarboxylate **5** (1 equiv) and AgOTf (10 mol %) in THF (0.1 M) at -78 °C. The reaction was warmed to RT after 10 minutes and stirred at RT for 2-3 h. After TLC showed consumption of azocarboxylate **5**, the reaction was cooled to -78 °C and KHMDS (1 M in THF, 3 equiv) was added dropwise. After 10 minutes the reaction was warmed to -20 °C and stirred at -20 °C for 1-4 h. The reaction was quenched with sat. aq. NH_4Cl (10 mL), and the aqueous layer was extracted with EtOAc (3 × 10 mL). The combined organics were washed with brine (1 × 10 mL), dried over $MgSO_4$ and concentrated under reduced pressure to give a crude residue. Purification by flash silica chromatography (EtOAC + Hexane) afforded the title compound.

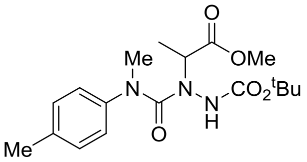

tert-Butyl 2-(1-methoxy-1-oxopropan-2-yl)-2-(methyl(*p*-tolyl)carbamoyl) hydrazinecarboxylate (6) **^{1}H NMR** (400 MHz, $CDCl_3$) δ 7.17 – 7.02 (m, 4H), 5.93 (br s, 1H), 4.86 (br s, 1H), 3.69 (s, 3H), 3.19 (s, 3H), 2.30 (s, 3H), 1.44 – 1.24 (m, 12H); **^{13}C NMR** (101 MHz, $CDCl_3$) δ 171.2, 159.9, 154.4, 142.3, 135.7, 130.1, 124.7, 80.6, 66.8, 52.2, 40.1, 28.1, 20.9, 14.1. HRMS (ESI) calcd for [$C_{18}H_{27}N_3O_5Na$] requires [M + Na]+ 388.1848, found 388.1845.

1.15 Procedure and Analytical data of aza boc-hydantoins (7)

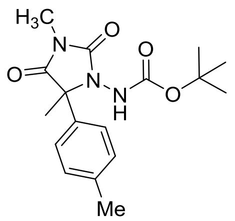

tert-Butyl (3,5-dimethyl-2,4-dioxo-5-(p-tolyl)imidazolidin-1-yl)carbamate (7a); (1-methoxyprop-1-en-1-yl)oxy)trimethylsilane (66 mg, 0.41 mmol,) was added dropwise to a mixture of tert-butyl (*E*)-2-(methyl(*p*-tolyl)carbamoyl)diazene-1-carboxylate **4a** (115 mg, 0.41 mmol) and AgOTf (10.4 mg, 10 mol %) in THF (4.1 mL, 0.1 M) at -78 ºC. The reaction was warmed to RT after 10 minutes and stirred at RT for 2 h. After TLC showed consumption of **4a**, the reaction was cooled to -78 ºC and KHMDS (1 M in THF, 3 equiv) was added dropwise. After 10 minutes the reaction was warmed to -20 ºC and stirred at -20 ºC for 2 h. The reaction was quenched with sat. aq. NH_4Cl (10 mL), and the aqueous layer was extracted with EtOAc (3 × 10 mL). The combined organics were washed with brine (1 × 10 mL), dried over $MgSO_4$ and concentrated under reduced pressure to give a crude residue. Purification by flash silica chromatography (20% EtOAC + Hexane) afforded the title compound (100 mg, 72%) as a colourless needle

MP: 180-182 ºC

***V*$_{max}$ /cm^{-1}(neat):** 3289, 2979, 1786, 1710, 1454, 1245, 1157, 1046, 756

^{1}H NMR (400 MHz, $CDCl_3$) δ 7.23 – 7.14 (m, 4H), 6.56 (s, 1H), 3.09 (s, 3H), 2.33 (s, 3H), 1.83 (s, 3H), 1.44 (s, 9H); **^{13}C NMR** (126 MHz, CDCl3) δ 173.6, 155.3, 154.7, 138.8, 133.6, 129.8, 125.9, 82.5, 68.3, 28.0, 25.2, 21.0, 19.9; HRMS (ESI) calcd for $[C_{17}H_{23}N_3O_4Na]^+$ requires $[M + Na]^+$ 356.1586, found 356.1587

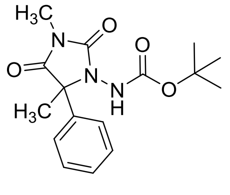

tert-Butyl (3,5-dimethyl-2,4-dioxo-5-phenylimidazolidin-1-yl)carbamate (7b); 1-Methoxy-1-trimethylsilyloxypropene (71 mg, 0.44 mmol,) was added dropwise to a mixture of tert-butyl (*E*)-2-(methyl(phenyl)carbamoyl)diazene-1-carboxylate **5b** (100 mg, 0.38 mmol) and AgOTf (9.5 mg, 10 mol %.) in THF (3.8 mL, 0.1 M) at -78 ºC. The reaction was warmed to RT after 30 minutes and stirred at RT for 2 h. After TLC showed consumption of **5b**, the reaction was cooled to -78 ºC and KHMDS (1 M in THF, 3 equiv) was added dropwise. After 10 minutes the reaction was warmed to -20 ºC and stirred at -20 ºC for 2 h. The reaction was quenched with sat. aq. NH_4Cl (10 mL), and the aqueous layer was extracted with EtOAc (3 × 10 mL). The combined organics were washed with brine (1 × 10 mL), dried over $MgSO_4$ and concentrated under reduced pressure to give a crude residue. Purification by flash silica chromatography (20% EtOAC/Hexane) afforded the title compound (78 mg, 60%) as white needles.

MP: 176-178 ºC

V_{max} /cm^{-1}(neat): 3289, 2980, 1787, 1707, 1448, 1155, 751

^{1}H NMR (400 MHz, $CDCl_3$) δ 7.40–7.31 (m, 5H), 6.33 (br s, 1H), 3.09 (s, 3H), 1.85 (s, 3H), 1.43 (s, 9H); **^{13}C NMR** (100 MHz, CDCl3) δ 173.4, 154.6, 129.1, 128.9, 125.9, 82.6, 68.5, 28.0, 25.2, 19.9; HRMS (ESI) calcd for [$C_{16}H_{21}N_3O_4Na$] requires [M + Na]+ 342.1430, found 342.1419.

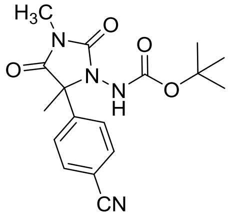

tert-Butyl (5-(4-cyanophenyl)-3,5-dimethyl-2,4-dioxoimidazolidin-1-yl)carbamate (7c); (1-methoxyprop-1-en-1-yl)oxy)trimethylsilane (77 mg, 0.48 mmol,) was added dropwise to a mixture of tert-butyl (*E*)-2-((4-cyanophenyl)(methyl)carbamoyl)diazene-1-carboxylate **5c** (140 mg, 0.48 mmol) and AgOTf (12.2 mg, 10 mol %) in THF (4.8 mL, 0.1 M) at -78 ºC. The

reaction was warmed to RT after 10 minutes and stirred at RT for 3 h. After TLC showed consumption of tert-butyl (*E*)-2-((4-cyanophenyl)(methyl)carbamoyl)diazene-1-carboxylate **5c**, the reaction was cooled to -78 ºC and KHMDS (1 M in THF, 3 equiv) was added dropwise. After 10 minutes the reaction was warmed to -20 ºC and stirred at -20 ºC for 2 h. The reaction was quenched with sat. aq. NH_4Cl (10 mL), and the aqueous layer was extracted with EtOAc (3 × 10 mL). The combined organics were washed with brine (1 × 10 mL), dried over $MgSO_4$ and concentrated under reduced pressure to give a crude residue. Purification by flash silica chromatography (30% EtOAC + Hexane) afforded the title compound (120 mg, 71.8%) as a colourless needle

MP: 180-182 ºC

***V*max /cm^{-1}(neat):** 3298, 2981, 2230, 1789, 1712, 1454, 1246, 1156, 1047, 845, 753

^{1}H NMR (400 MHz, $CDCl_3$) δ 7.68 (d, *J* = 8.7 Hz, 2H), 7.52 (d, *J* = 8.4 Hz, 2H), 6.35 (s, 1H), 3.10 (s, 3H), 1.87 (s, 3H), 1.44 (s, 9H); **^{13}C NMR** (126 MHz, CDCl3) δ 172.3, 155.1, 154.6, 142.0, 132.6, 127.0, 118.1, 112.8, 83.0, 68.2, 28.0, 25.4, 20.3; HRMS (ESI) calcd for $[C_{17}H_{20}N_4O_4Na]^+$ requires $[M + Na]^+$ 367.1382, found 367.1388.

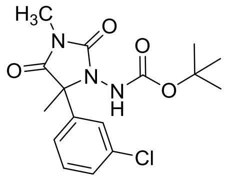

***tert*-Butyl (5-(3-chlorophenyl)-3,5-dimethyl-2,4-dioxoimidazolidin-1-yl)carbamate (7d)**; (1-methoxyprop-1-en-1-yl)oxy)trimethylsilane (67 mg, 0.41 mmol,) was added dropwise to a mixture of tert-butyl (*E*)-2-((3-chlorophenyl)(methyl)carbamoyl)diazene-1-carboxylate **5d** (125 mg, 0.42 mmol) and AgOTf (10.6 mg, 10 mol %) in THF (4.2 mL, 0.1 M) at -78 ºC. The reaction was warmed to RT after 10 minutes and stirred at RT for 2 h. After TLC showed consumption of tert-butyl (*E*)-2-((3-chlorophenyl)(methyl)carbamoyl)diazene-1-carboxylate **5d**, the reaction was cooled to -78 ºC and KHMDS (1 M in THF, 3 equiv) was added dropwise. After 10 minutes the reaction was warmed to -20 ºC and stirred at -20 ºC for 2 h. The reaction

was quenched with sat. aq. NH_4Cl (10 mL), and the aqueous layer was extracted with EtOAc (3 × 10 mL). The combined organics were washed with brine (1 × 10 mL), dried over $MgSO_4$ and concentrated under reduced pressure to give a crude residue. Purification by flash silica chromatography (25% EtOAC+Hexane) afforded the title compound (90 mg, 60%) as a colourless needle

MP: 169-171 °C

***V*max /cm^{-1}(neat):** 3293, 2979, 1788, 1708, 1453, 1244, 1155, 1046, 753

^{1}H NMR (400 MHz, $CDCl_3$) δ 7.32 (d, *J* = 3.0 Hz, 3H), 7.23 (dd, *J* = 4.2, 2.9 Hz, 1H), 6.37 (s, 1H), 3.10 (s, 3H), 1.84 (s, 3H), 1.44 (s, 9H); **^{13}C NMR** (126 MHz, CDCl3) δ 172.8, 155.0, 154.6, 135.1, 130.3, 129.1, 126.4, 124.3, 82.8, 68.1, 28.0, 25.4, 20.1.HRMS (ESI) calcd for $[C_{16}H_{20}ClN_3O_4Na]^+$ requires $[M + Na]^+$ 376.1040, found 376.1041

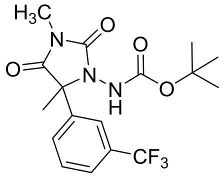

***tert*-Butyl (3,5-dimethyl-2,4-dioxo-5-(3-(trifluoromethyl)phenyl)imidazolidin-1-yl)carbamate (7e)**; (1-methoxyprop-1-en-1-yl)oxy)trimethylsilane (48 mg, 0.30 mmol,) was added dropwise to a mixture of tert-butyl (*E*)-2-(methyl(3-(trifluoromethyl)phenyl)carbamoyl)diazene-1-carboxylate **5e** (100 mg, 0.30 mmol) and AgOTf (7.6 mg, 10 mol %) in THF (3.0 mL, 0.1 M) at -78 °C. The reaction was warmed to RT after 10 minutes, and stirred at RT for 3 h. After TLC showed consumption of tert-butyl (*E*)-2-(methyl(3-(trifluoromethyl)phenyl)carbamoyl)diazene-1-carboxylate **5e**, the reaction was cooled to -78 °C and KHMDS (1 M in THF, 3 equiv) was added dropwise. After 10 minutes the reaction was warmed to -20 °C and stirred at -20 °C for 2 h. The reaction was quenched with sat. aq. NH_4Cl (10 mL), and the aqueous layer was extracted with EtOAc (3 × 10 mL). The combined organics were washed with brine (1 × 10 mL), dried over $MgSO_4$ and concentrated under reduced pressure to give a crude residue. Purification by flash silica

chromatography (30% EtOAC + Hexane) afforded the *title compound* (91 mg, 65%) as colourless needles.

MP: 177-179 ºC

V_{max} /cm^{-1}(neat): 3292, 2982, 1789, 1709, 1328, 1123, 754, 699

^{1}H NMR (400 MHz, $CDCl_3$) δ 7.66 – 7.54 (m, 3H), 7.54 – 7.46 (m, 1H), 6.64 (s, 1H), 3.09 (s, 3H), 1.87 (s, 3H), 1.41 (s, 9H); **^{13}C NMR** (100 MHz, CDCl3) δ 172.8, 155.4, 154.8, 138.1, 131.6, 131.3, 130.9, 127.7 (q, J_{C-F} = 2.6 Hz), 129.6, 126.2 (q, J_{C-F} = 272.2 Hz), 125.7 (q, J_{C-F} = 3.6 Hz), 122.9 (q, J_{C-F} = 3.5 Hz), 82.9, 68.3, 28.0, 25.4, 20.5; HRMS (ESI) calcd for $[C_{17}H_{20}F_3N_3O_4Na]^+$ requires $[M + Na]^+$ 410.1304, found 410.1283

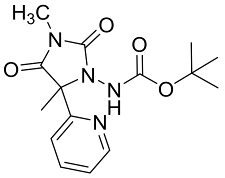

***tert*-Butyl (3,5-dimethyl-2,4-dioxo-5-(pyridin-2-yl)imidazolidin-1-yl)carbamate (7f)**; (*E*)-((1-methoxyprop-1-en-1-yl)oxy)trimethylsilane (60 mg, 0.37 mmol,) was added dropwise to a mixture of *tert*-butyl (E)-2-(methyl(pyridin-2-yl)carbamoyl)diazene-1-carboxylate **5f** (100 mg, 0.37 mmol) and AgOTf (9.5 mg, 8.4 mol %) in THF (3.7 mL, 0.1 M) at -78 ºC. The reaction was warmed to RT after 10 minutes, and stirred at RT for 3 h. After TLC showed consumption of *tert*-butyl (E)-2-(methyl(pyridin-2-yl)carbamoyl)diazene-1-carboxylate **5f**, the reaction was cooled to -78 ºC and KHMDS (1 M in THF, 3 equiv) was added dropwise. After 10 minutes the reaction was warmed to -20 ºC and stirred at -40 ºC for 2 h. The reaction was quenched with sat. aq. NH_4Cl (10 mL), and the aqueous layer was extracted with EtOAc (3 × 10 mL). The combined organics were washed with brine (1 × 10 mL), dried over $MgSO_4$ and concentrated under reduced pressure to give a crude residue. Purification by flash silica chromatography (30% EtOAC + Hexane) afforded the *title compound* (60 mg, 50%) as a colourless semi-solid.

***V*$_{max}$ /cm^{-1}(neat):** 3288, 2980, 1789, 1711, 1452, 1154, 748

^{1}H NMR (400 MHz, $CDCl_3$) δ 8.46 (ddd, *J* = 4.8, 1.7, 0.8 Hz, 1H), 7.69 (td, *J* = 7.8, 1.8 Hz, 1H), 7.49 (d, *J* = 7.9 Hz, 1H), 7.23 – 7.18 (m, 1H), 6.83 (s, 1H), 2.99 (s, 3H), 1.83 (s, 3H), 1.40 (s, 9H); **^{13}C NMR** (100 MHz, CDCl3) δ 172.3, 154.7, 149.2, 137.5, 123.5, 121.6, 81.9, 69.9, 28.1, 25.2, 19.8; HRMS (ESI) calcd for $[C_{15}H_{20}N_4O_4Na]^+$ requires $[M + Na]^+$ 343.1382, found 343.1370

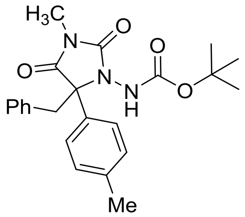

***tert*-Butyl (5-benzyl-3-methyl-2,4-dioxo-5-(*p*-tolyl)imidazolidin-1-yl)carbamate (7g)**; (1-methoxy-3-phenylprop-1-en-1-yl)oxy)trimethylsilane (85 mg, 0.36 mmol,) was added dropwise to a mixture of tert-butyl (*E*)-2-(methyl(p-tolyl)carbamoyl)diazene-1-carboxylate **5a** (100 mg, 0.36 mmol) and AgOTf (9.0 mg, 10 mol %) in THF (3.6 mL, 0.1 M) at -78 °C. The reaction was warmed to RT after 10 minutes and stirred at RT for 2 h. After TLC showed consumption of tert-butyl (*E*)-2-(methyl(p-tolyl)carbamoyl)diazene-1-carboxylate **5a**, the reaction was cooled to -78 °C and KHMDS (1 M in THF, 3 equiv) was added dropwise. After 10 minutes the reaction was warmed to -20 °C and stirred at -20 °C for 2 h. The reaction was quenched with sat. aq. NH_4Cl (10 mL), and the aqueous layer was extracted with EtOAc (3 × 10 mL). The combined organics were washed with brine (1 × 10 mL), dried over $MgSO_4$ and concentrated under reduced pressure to give a crude residue. Purification by flash silica chromatography (20% EtOAC + Hexane) afforded the title compound (125 mg, 85%) as a semi-solid.

***V*$_{max}$ /cm^{-1}(neat):** 2978, 1730, 1665, 1513, 1366, 1238, 1153, 749

^{1}H NMR (400 MHz, $CDCl_3$) δ 7.26 – 7.17 (m, 4H), 7.09 (dt, *J* = 12.0, 6.0 Hz, 5H), 3.20 (s, 3H), 3.08 (dd, *J* = 13.8, 6.0 Hz, 1H), 3.02 – 2.89 (m, 1H), 2.32 (s, 3H), 1.39 (s, 9H); **^{13}C NMR** (101MHz, CDCl3) δ 171.2, 160.0, 154.4, 142.1, 137.5, 135.6, 130.1, 129.2, 128.2, 126.4, 124.4, 81.0, 61.0, 39.9, 35.3, 28.1, 20.9; HRMS (ESI) calcd for $[C_{23}H_{27}N_3O_4Na]^+$ requires $[M + Na]^+$ 432.1899, found 432.1902

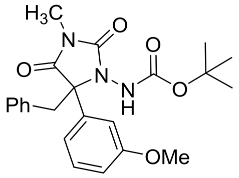

***tert*-Butyl (5-benzyl-5-(3-methoxyphenyl)-3-methyl-2,4-dioxoimidazolidin-1-yl) carbamate (7h)**; (1-methoxy-3-phenylprop-1-en-1-yl)oxy)trimethylsilane (80 mg, 0.34 mmol,) was added dropwise to a mixture of tert-butyl (*E*)-2-((3-methoxyphenyl)(methyl)carbamoyl)diazene-1-carboxylate **5g** (100 mg, 0.34 mmol) and AgOTf (8.6 mg, 10 mol %) in THF (3.4 mL, 0.1 M) at -78 °C. The reaction was warmed to RT after 10 minutes and stirred at RT for 3.5 h. After TLC showed consumption of tert-butyl (*E*)-2-((3-methoxyphenyl)(methyl)carbamoyl)diazene-1-carboxylate **5g**, the reaction was cooled to -78 °C and KHMDS (1 M in THF, 3 equiv) was added dropwise. After 10 minutes the reaction was warmed to -20 °C and stirred at -20 °C for 2 h. The reaction was quenched with sat. aq. NH_4Cl (10 mL), and the aqueous layer was extracted with EtOAc (3 × 10 mL). The combined organics were washed with brine (1 × 10 mL), dried over $MgSO_4$ and concentrated under reduced pressure to give a crude residue. Purification by flash silica chromatography (25% EtOAC + Hexane) afforded the title compound (75 mg, 51%) as colorless semi-solid

***V_{max}* /cm^{-1}(neat):** 3282, 2978, 1788, 1709, 1453, 1155, 734, 700

^{1}H NMR (400 MHz, $CDCl_3$) δ 7.35 – 7.26 (m, 4H), 7.18 (dd, *J* = 7.6, 1.7 Hz, 3H), 7.13 (d, *J* = 7.7 Hz, 1H), 6.91 (dd, *J* = 8.2, 2.4 Hz, 1H), 6.35 (s, 1H), 3.80 (s, 3H), 3.63 – 3.48 (m, 2H), 2.81 (s, 3H), 1.32 (s, 9H); **^{13}C NMR** (101MHz, $CDCl_3$) δ 172.2, 159.8, 154.9, 154.5, 136.7,

134.0, 129.8, 129.6, 128.9, 127.9, 119.5, 82.5, 72.9, 55.4, 40.3, 27.8, 24.8; HRMS (ESI) calcd for $[C_{23}H_{27}N_3O_5Na]^+$ requires $[M + Na]^+$ 448.1848, found 448.1844

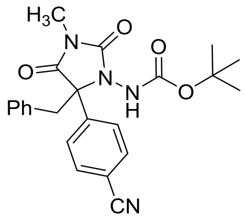

***tert*-Butyl (5-benzyl-5-(4-cyanophenyl)-3-methyl-2,4-dioxoimidazolidin-1-yl)carbamate (7i)**; (*E*)-((1-methoxy-3-phenylprop-1-en-1-yl)oxy)trimethylsilane (73 mg, 0.30 mmol,) was added dropwise to a mixture of tert-butyl (E)-2-((4-cyanophenyl)(methyl)carbamoyl)diazene-1-carboxylate **5c** (100 mg, 034 mmol) and AgOTf (8.7 mg, 10 mol %) in THF (3.4 mL, 0.1 M) at -78 °C. The reaction was warmed to RT after 10 minutes and stirred at RT for 1 h. After TLC showed consumption of tert-butyl (*E*)-2-((4-cyanophenyl) (methyl)carbamoyl)diazene-1-carboxylate **5c**, the reaction was cooled to -78 °C and KHMDS (1 M in THF, 3 equiv) was added dropwise. After 10 minutes the reaction was warmed to -20 °C and stirred at -20 °C for 1 h. The reaction was quenched with sat. aq. NH_4Cl (10 mL), and the aqueous layer was extracted with EtOAc (3 × 10 mL). The combined organics were washed with brine (1 × 10 mL), dried over $MgSO_4$ and concentrated under reduced pressure to give a crude residue. Purification by flash silica chromatography (30% EtOAC + Hexane) afforded the title compound (120 mg, 82%) as colourless needles

MP: 184-185 °C

***V*max /cm^{-1}(neat):** 3295, 2980, 2231, 1790, 1712, 1454, 1156, 754

^{1}H NMR (500 MHz, $CDCl_3$) δ 7.82 (d, *J* = 7.8 Hz, 2H), 7.73 (d, *J* = 8.7 Hz, 2H), 7.39 – 7.31 (m, 3H), 7.17 (dd, *J* = 7.6, 1.7 Hz, 2H), 6.24 (s, 1H), 3.67 (d, *J* = 12.9 Hz, 1H), 3.49 (d, *J* = 13.9 Hz, 1H), 2.88 (s, 3H), 1.39 (s, 9H); **^{13}C NMR** (126 MHz, CDCl3) δ 171.4, 154.7, 154.3, 140.4, 133.2, 132.3, 129.3, 129.1, 128.3, 128.2, 118.3, 112.9, 83.1, 72.5, 40.3, 27.8, 24.9; HRMS (ESI) calcd for $[C_{23}H_{24}N_4O_4Na]^+$ requires $[M + Na]^+$ 443.1695, found 443.1672

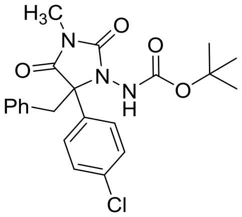

***tert*-Butyl (5-benzyl-5-(4-chlorophenyl)-3-methyl-2,4-dioxoimidazolidin-1-yl)carbamate (7j)**; (1-methoxy-3-phenylprop-1-en-1-yl)oxy)trimethylsilane (79 mg, 0.33 mmol,) was added dropwise to a mixture of tert-butyl (*E*)-2-((4-chlorophenyl)(methyl)carbamoyl) diazene-1-carboxylate **5h** (100 mg, 0.33 mmol) and AgOTf (8.4 mg, 10 mol %) in THF (3.3 mL, 0.1 M) at -78 ^{o}C. The reaction was warmed to RT after 10 minutes and stirred at RT for 2 h. After TLC showed consumption of tert-butyl (*E*)-2-((4-chlorophenyl)(methyl)carbamoyl) diazene-1-carboxylate **5h**, the reaction was cooled to -78 ^{o}C and KHMDS (1 M in THF, 3 equiv) was added dropwise. After 10 minutes the reaction was warmed to -20 ^{o}C and stirred at -20 ^{o}C for 2 h. The reaction was quenched with sat. aq. NH_4Cl (10 mL), and the aqueous layer was extracted with EtOAc (3 × 10 mL). The combined organics were washed with brine (1 × 10 mL), dried over $MgSO_4$ and concentrated under reduced pressure to give a crude residue. Purification by flash silica chromatography (20% EtOAC + Hexane) afforded the title compound (120 mg, 83%) as a white needls.

MP: 169-171°C

$\boldsymbol{V_{max}}$ **/cm^{-1}(neat):** 3273, 2979, 1789, 1708, 1453, 1156, 753

^{1}H NMR (400 MHz, $CDCl_3$) δ 7.53 (d, *J* = 8.2 Hz, 2H), 7.37 (d, *J* = 8.7 Hz, 2H), 7.29 (q, *J* = 5.5 Hz, 3H), 7.14 (dd, *J* = 7.5, 1.8 Hz, 2H), 6.25 (s, 1H), 3.61 – 3.42 (m, 2H), 2.82 (s, 3H), 1.33 (s, 9H); **^{13}C NMR** (101MHz, CDCl3) δ 171.9, 154.6, 154.4, 135.0, 133.7, 133.6, 129.5, 128.9, 128.8, 128.7, 127.9, 82.7, 72.3, 40.2, 27.8, 24.7; HRMS (ESI) calcd for $[C_{22}H_{24}ClN_3O_4Na]^+$ requires $[M + Na]^+$ 452.1353, found 452.1338

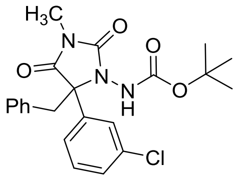

***tert*-Butyl (5-benzyl-5-(3-chlorophenyl)-3-methyl-2,4-dioxoimidazolidin-1-yl)carbamate (7k)**; (1-methoxy-3-phenylprop-1-en-1-yl)oxy)trimethylsilane (76 mg, 0.32 mmol,) was added dropwise to a mixture of tert-butyl (E)-2-((3-chlorophenyl)(methyl)carbamoyl)diazene-1-carboxylate **5d** (107 mg, 0.36 mmol) and AgOTf (9.07 mg, 10 mol %) in THF (3.6 mL, 0.1 M) at -78 °C. The reaction was warmed to RT after 10 minutes and stirred at RT for 2 h. After TLC showed consumption of tert-butyl (*E*)-2-((3-chlorophenyl)(methyl)carbamoyl)diazene-1-carboxylate **5d**, the reaction was cooled to -78 °C and KHMDS (1 M in THF, 3 equiv) was added dropwise. After 10 minutes the reaction was warmed to -20 °C and stirred at -20 °C for 2 h. The reaction was quenched with sat. aq. NH_4Cl (10 mL), and the aqueous layer was extracted with EtOAc (3 × 10 mL). The combined organics were washed with brine (1 × 10 mL), dried over $MgSO_4$ and concentrated under reduced pressure to give a crude residue. Purification by flash silica chromatography (20% EtOAC + Hexane) afforded the title compound (113 mg, 78%) as a colourless needle **MP:** 152-154 °C.

***V_{max}* /cm^{-1}(neat):** 3268, 2980, 1789, 1709, 1454, 1155, 753, 701

^{1}H NMR (400 MHz, $CDCl_3$) δ 7.78 (d, *J* = 8.3 Hz, 2H), 7.70 (d, *J* = 8.4 Hz, 2H), 7.32 (qd, *J* = 5.9, 2.2 Hz, 3H), 7.13 (dd, *J* = 7.5, 1.9 Hz, 2H), 6.19 (s, 1H), 3.63 (d, *J* = 14.3 Hz, 1H), 3.45 (d, *J* = 13.9 Hz, 1H), 2.84 (s, 3H), 1.35 (s, 9H); **^{13}C NMR** (101MHz, CDCl3) δ 171.7, 155.0, 154.5, 137.2, 134.6, 133.5, 130.0, 129.7, 129.1, 128.8, 128.0, 127.7, 125.7, 82.7, 72.4, 40.2, 27.8, 24.8; HRMS (ESI) calcd for $[C_{22}H_{24}ClN_3O_4Na]^+$ requires $[M + Na]^+$ 452.1353, found 452.1331

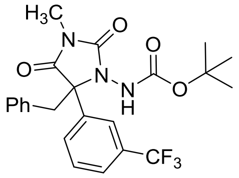

***tert*-Butyl (5-benzyl-3-methyl-2,4-dioxo-5-(3-(trifluoromethyl)phenyl)imidazolidin-1-yl)carbamate (7l)**; (1-methoxy-3-phenylprop-1-en-1-yl)oxy)trimethylsilane (121.9 mg, 0.51 mmol,) was added dropwise to a mixture of tert-butyl (*E*)-2-(methyl(3-(trifluoromethyl)phenyl)carbamoyl)diazene-1-carboxylate **5e** (190 mg, 0.57 mmol) and AgOTf (15.2 mg, 10 mol %) in THF (6.0 mL, 0.1 M) at -78 ºC. The reaction was warmed to RT after 10 minutes, and stirred at RT for 3 h. After TLC showed consumption of tert-butyl (*E*)-2-(methyl(3-(trifluoromethyl)phenyl)carbamoyl)diazene-1-carboxylate **5e**, the reaction was cooled to -78 ºC and KHMDS (1 M in THF, 3 equiv) was added dropwise. After 10 minutes the reaction was warmed to -20 ºC and stirred at -40 ºC for 2 h. The reaction was quenched with sat. aq. NH_4Cl (10 mL), and the aqueous layer was extracted with EtOAc (3 × 10 mL). The combined organics were washed with brine (1 × 10 mL), dried over $MgSO_4$ and concentrated under reduced pressure to give a crude residue. Purification by flash silica chromatography (30% EtOAC + Hexane) afforded the title compound (155 mg, 70%) as colourless needles.

MP: 141-143 ºC

***V*max /cm⁻¹(neat):** 3268, 2988, 1790, 1713, 1329, 1129, 748, 699

^{1}H NMR (400 MHz, $CDCl_3$) δ 7.78 (s, 1H), 7.65 (d, *J* = 7.9 Hz, 1H), 7.50 (d, *J* = 7.7 Hz, 1H), 7.40 (t, *J* = 7.9 Hz, 1H), 7.16 (d, *J* = 7.1 Hz, 3H), 7.02 (dd, *J* = 7.5, 1.9 Hz, 2H), 6.26 (s, 1H), 3.41 (dd, *J* = 16.9, 13.6 Hz, 2H), 2.69 (s, 3H), 1.16 (s, 9H); **^{13}C NMR** (100 MHz, CDCl3) δ 171.6, 154.6, 154.2, 136.4, 133.4, 130.9 (q, J_{C-F} = 12.4 Hz), 129.4, 129.2, 128.9, 128.1, 125.7 (q, J_{C-F} = 3.8 Hz), 125.2 (q, J_{C-F} = 272.5 Hz), 124.0 (q, J_{C-F} = 3.8 Hz), 82.8, 72.4, 40.6, 27.7, 24.8; HRMS (ESI) calcd for $[C_{23}H_{24}F_3N_3O_4Na]^+$ requires $[M + Na]^+$ 486.1617, found 486.1615

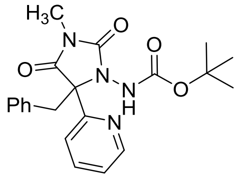

***tert*-Butyl (5-benzyl-3-methyl-2,4-dioxo-5-(3-(trifluoromethyl)phenyl)imidazolidin-1-yl) carbamate (7m)**; (1-methoxy-3-phenylprop-1-en-1-yl)oxy)trimethylsilane (126 mg, 0.53 mmol,) was added dropwise to a mixture of tert-butyl (*E*)-2-(methyl(pyridin-2-yl)carbamoyl)diazene-1-carboxylate **5f** (157 mg, 0.59 mmol) and AgOTf (14.9 mg, 10 mol %) in THF (5.94 mL, 0.1 M) at -78 °C. The reaction was warmed to RT after 10 minutes, and stirred at RT for 2 h. After TLC showed consumption of tert-butyl (*E*)-2-(methyl(pyridin-2-yl)carbamoyl)diazene-1-carboxylate **5f**, the reaction was cooled to -78 °C and KHMDS (1 M in THF, 3 equiv) was added dropwise. After 10 minutes the reaction was warmed to -20 °C and stirred at -20 °C for 2 h. The reaction was quenched with sat. aq. NH_4Cl (10 mL), and the aqueous layer was extracted with EtOAc (3 × 10 mL). The combined organics were washed with brine (1 × 10 mL), dried over $MgSO_4$ and concentrated under reduced pressure to give a crude residue. Purification by flash silica chromatography (30% EtOAC + Hexane) afforded the title compound (163 mg, 85%) as a pale-yellow semi-solid.

***V_{max}* /cm^{-1}(neat):** 2977, 1792, 1706, 1594, 1435, 1136, 748

^{1}H NMR (400 MHz, $CDCl_3$) δ 11.77 (s, 1H), 8.26 (d, *J* = 3.6 Hz, 1H), 7.77 – 7.65 (m, 1H), 7.31 (d, *J* = 7.1 Hz, 2H), 7.28 – 7.23 (m, 2H), 7.18 (t, *J* = 7.0 Hz, 1H), 6.99 (d, *J* = 8.5 Hz, 2H), 3.40 (s, 3H), 3.25 (dd, *J* = 13.4, 7.9 Hz, 1H), 3.14 (dd, *J* = 14.0, 7.5 Hz, 1H), 1.37 (s, 9H); **^{13}C NMR** (101 MHz, CDCl3) 171.2, 157.4, 155.3, 146.0, 138.9, 138.0, 137.2, 129.4, 128.5, 128.3, 126.4, 117.6, 111.8, 82.07 74.14, 33.39, 28.08, 14.15; HRMS (ESI) calcd for $[C_{21}H_{24}N_4O_4Na]^+$ requires $[M + Na]^+$ 419.1695, found 419.1675

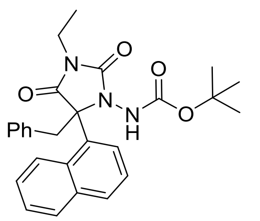

***tert*-Butyl (5-benzyl-3-methyl-5-(naphthalen-1-yl)-2,4-dioxoimidazolidin-1-yl)carbamate (7n)**; (1-methoxy-3-phenylprop-1-en-1-yl)oxy)trimethylsilane (81 mg, 0.34 mmol,) was added

dropwise to a mixture of tert-butyl (E)-2-(ethyl(naphthalen-1-yl)carbamoyl)diazene-1-carboxylate **5i** (100 mg, 0.34 mmol) and AgOTf (8.7 mg, 10 mol %) in THF (3.4 mL, 0.1 M) at -78 °C. The reaction was warmed to RT after 10 minutes and stirred at RT for 2.5 h. After TLC showed consumption of tert-butyl (E)-2-(ethyl(naphthalen-1-yl)carbamoyl)diazene-1-carboxylate **5i**, the reaction was cooled to -78 °C and KHMDS (1 M in THF, 3 equiv) was added dropwise. After 10 minutes the reaction was warmed to -20 °C and stirred at -20 °C for 4 h. The reaction was quenched with sat. aq. NH_4Cl (10 mL), and the aqueous layer was extracted with EtOAc (3 × 10 mL). The combined organics were washed with brine (1 × 10 mL), dried over $MgSO_4$ and concentrated under reduced pressure to give a crude residue. Purification by flash silica chromatography (20% EtOAC + Hexane) afforded the title compound (90 mg, 56%) as colorless oil.

***V*max /cm^{-1}(neat):** 3264, 2978, 1785, 1712, 1495, 1392, 1158, 775

^{1}H NMR (400 MHz, $CDCl_3$) δ 7.88 – 7.78 (m, 3H), 7.53 – 7.42 (m, 3H), 7.39 – 7.30 (m, 3H), 7.28 – 7.21 (m, 3H), 6.21 (s, 1H), 4.00 (d, *J* = 13.0 Hz, 1H), 3.54 (d, *J* = 12.9 Hz, 1H), 3.25 (q, *J* = 7.2 Hz, 2H), 0.94 (s, 9H), 0.78 (t, *J* = 7.2 Hz, 3H); **^{13}C NMR** (101MHz, CDCl3) δ 172.3, 155.4, 154.8, 134.5, 133.5, 131.6, 131.0, 130.8, 129.8, 128.7, 127.9, 127.9, 127.1, 125.8, 124.9, 123.3, 82.3, 71.9, 40.7, 33.8, 27.6, 12.3; HRMS (ESI) calcd for $[C_{27}H_{29}N_3O_4Na]^+$ requires $[M + Li]^+$ 482.2056, found 482.2058.

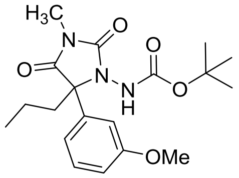

***tert*-Butyl (5-(3-methoxyphenyl)-3-methyl-2,4-dioxo-5-propylimidazolidin-1-yl)carbamate (7o)**; (1-methoxypent-1-en-1-yl)oxy)trimethylsilane (64 mg, 0.34 mmol,) was added dropwise to a mixture of tert-butyl (*E*)-2-((3-methoxyphenyl)(methyl)carbamoyl)diazene-1-carboxylate **5g** (100 mg, 0.34 mmol) and

AgOTf (8.6 mg, 10 mol %) in THF (3.4 mL, 0.1 M) at -78 °C. The reaction was warmed to RT after 10 minutes and stirred at RT for 3 h. After TLC showed consumption of tert-butyl (*E*)-2-((3-methoxyphenyl)(methyl)carbamoyl)diazene-1-carboxylate **5g**, the reaction was cooled to -78 °C and KHMDS (1 M in THF, 3 equiv) was added dropwise. After 10 minutes the reaction was warmed to -20 °C and stirred at -20 °C for 4 h. The reaction was quenched with sat. aq. NH_4Cl (10 mL), and the aqueous layer was extracted with EtOAc (3 × 10 mL). The combined organics were washed with brine (1 × 10 mL), dried over $MgSO_4$ and concentrated under reduced pressure to give a crude residue. Purification by flash silica chromatography (20% EtOAC + Hexane) afforded the title compound (53 mg, 42%) as a colorless Semi-solid.

***V_{max}* /cm^{-1}(neat):** 3284, 2966, 1786, 1710, 1454, 1245, 1154

^{1}H NMR (400 MHz, $CDCl_3$) δ 7.28 (t, *J* = 8.1 Hz, 1H), 6.92 – 6.83 (m, 3H), 6.17 (s, 1H), 3.78 (s, 3H), 3.09 (s, 3H), 2.28 (td, *J* = 13.6, 4.6 Hz, 1H), 2.06 (td, *J* = 13.7, 3.6 Hz, 1H), 1.40 (s, 9H), 1.26 – 1.17 (m, 2H), 0.94 (t, *J* = 7.3 Hz, 3H); **^{13}C NMR** (101MHz, CDCl3) δ 172.6, 160.1, 155.5, 154.4, 141.8, 137.2, 130.1, 118.6, 113.7, 82.4, 71.7, 55.4, 28.0, 25.0, 16.8, 14.1; HRMS (ESI) calcd for $[C_{19}H_{27}N_3O_5Na]^+$ requires $[M + Na]^+$ 400.1848, found 400.1854

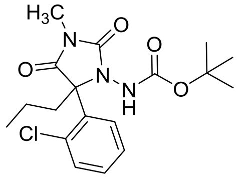

***tert*-Butyl (5-(2-chlorophenyl)-3-methyl-2,4-dioxo-5-propylimidazolidin-1-yl)carbamate (7p)**; (1-methoxypent-1-en-1-yl)oxy)trimethylsilane (70 mg, 0.37 mmol,) was added dropwise to a mixture of tert-butyl (E)-2-((2-chlorophenyl)(methyl)carbamoyl)diazene-1-carboxylate **5j** (112 mg, 0.37 mmol) and AgOTf (9.5 mg, 10 mol %) in THF (3.7 mL, 0.1 M) at -78 °C. The reaction was warmed to RT after 10 minutes and stirred at RT for 3 h. After TLC showed consumption of tert-butyl (*E*)-2-((2-chlorophenyl)(methyl)carbamoyl)diazene-1-carboxylate **5j**, the reaction was cooled to -78 °C and KHMDS (1 M in THF, 3 equiv) was added dropwise.

After 10 minutes the reaction was warmed to -20 °C and stirred at -20 °C for 4 h. The reaction was quenched with sat. aq. NH_4Cl (10 mL), and the aqueous layer was extracted with EtOAc (3 × 10 mL). The combined organics were washed with brine (1 × 10 mL), dried over $MgSO_4$ and concentrated under reduced pressure to give a crude residue. Purification by flash silica chromatography (15% EtOAC + Hexane) afforded the title compound (70 mg, 48%) as a colorless solid.

MP : 208-210 °C

V_{max} **/cm^{-1}(neat):** 3277, 2960, 1781, 1690, 1453, 1150

^{1}H NMR (400 MHz, $CDCl_3$) δ 7.58 – 7.54 (m, 1H), 7.38 – 7.35 (m, 1H), 7.33 – 7.28 (m, 2H), 6.01 (s, 1H), 3.10 (s, 3H), 2.24 (pd, *J* = 12.9, 3.8 Hz, 2H), 1.36 (s, 9H), 1.25 – 1.14 (m, 2H), 1.00 (t, *J* = 7.2 Hz, 3H); **^{13}C NMR** (101MHz, CDCl3) δ 172.5, 156.1, 154.8, 132.2, 131.5, 130.7, 130.5, 127.2, 82.3, 70.0, 36.2, 27.9, 25.0, 16.2, 14.1; **HRMS** (ESI) calcd for $[C_{18}H_{24}ClN_3O_4Na]^+$ requires $[M + Na]^+$ 404.1353, found 404.1355

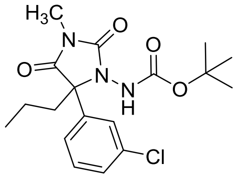

***tert*-Butyl (5-(3-chlorophenyl)-3-methyl-2,4-dioxo-5-propylimidazolidin-1-yl)carbamate (7q)**; (1-methoxypent-1-en-1-yl)oxy)trimethylsilane (65 mg, 0.35 mmol,) was added dropwise to a mixture of tert-butyl (*E*)-2-((3-chlorophenyl)(methyl)carbamoyl)diazene-1-carboxylate **5d** (104 mg, 0.34 mmol) and AgOTf (8.7 mg, 10 mol %) in THF (3.5 mL, 0.1 M) at -78 °C. The reaction was warmed to RT after 10 minutes and stirred at RT for 3 h. After TLC showed consumption of tert-butyl (*E*)-2-((3-chlorophenyl)(methyl)carbamoyl)diazene-1-carboxylate **5d**, the reaction was cooled to -78 °C and KHMDS (1 M in THF, 3 equiv) was added dropwise. After 10 minutes the reaction was warmed to -20 °C and stirred at -20 °C for 2 h. The reaction was quenched with sat. aq. NH_4Cl (10 mL), and the aqueous layer was extracted with EtOAc

(3 × 10 mL). The combined organics were washed with brine (1 × 10 mL), dried over $MgSO_4$ and concentrated under reduced pressure to give a crude residue. Purification by flash silica chromatography (20% EtOAC + Hexane) afforded the title compound (123 mg, 92%) as a colorless semi-solid.

***V*max /cm^{-1}(neat):** 3283, 2969, 1781, 1709, 1454, 1245, 1155, 750

^{1}H NMR (400 MHz, $CDCl_3$) δ 7.35 (s, 1H), 7.32 – 7.28 (m, 2H), 7.26 (dd, *J* = 7.0, 1.3 Hz, 1H), 6.41 (s, 1H), 3.10 (s, 3H), 2.24 (td, *J* = 13.7, 4.5 Hz, 1H), 2.09 (td, *J* = 14.0, 13.4, 3.9 Hz, 1H), 1.38 (s, 9H), 1.25 – 1.18 (m, 2H), 0.94 (t, *J* = 7.3 Hz, 3H); **^{13}C NMR** (101MHz, CDCl3) δ 172.2, 155.3, 154.4, 137.8, 134.9, 130.2, 129.0, 126.9, 124.9, 82.7, 71.3, 35.5, 27.9, 25.1, 16.8, 14.0.; HRMS (ESI) calcd for $[C_{18}H_{24}ClN_3O_4Na]^+$ requires $[M + Na]^+$ 404.1353, found 404.1348

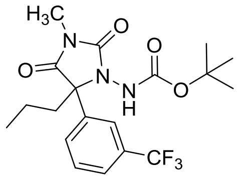

***tert*-Butyl (3-methyl-2,4-dioxo-5-propyl-5-(3-(trifluoromethyl)phenyl)imidazolidin-1-yl)carbamate (7r)**; (1-methoxypent-1-en-1-yl)oxy)trimethylsilane (64 mg, 0.34 mmol,) was added dropwise to a mixture of tert-butyl (*E*)-2-(methyl(3-(trifluoromethyl)phenyl)carbamoyl)diazene-1-carboxylate **5e** (113 mg, 0.34 mmol) and AgOTf (8.6 mg, 10 mol %) in THF (3.4 mL, 0.1 M) at -78 ºC. The reaction was warmed to RT after 10 minutes and stirred at RT for 3 h. After TLC showed consumption of tert-butyl (*E*)-2-(methyl(3-(trifluoromethyl)phenyl)carbamoyl)diazene-1-carboxylate **5e**, the reaction was cooled to -78 ºC and KHMDS (1 M in THF, 3 equiv) was added dropwise. After 10 minutes the reaction was warmed to -20 ºC and stirred at -20 ºC for 2 h. The reaction was quenched with sat. aq. NH_4Cl (10 mL), and the aqueous layer was extracted with EtOAc (3 × 10 mL). The combined organics were washed with brine (1 × 10 mL), dried over $MgSO_4$ and

concentrated under reduced pressure to give a crude residue. Purification by flash silica chromatography (20% EtOAC + Hexane) afforded the title compound (80 mg, 56%) as a colorless semi-solid.

***V_{max}* /cm^{-1}(neat):** 3282, 2968, 1784, 1712, 1454, 1329, 1163, 1127, 752

^{1}H NMR (400 MHz, $CDCl_3$) δ 7.68 (s, 1H), 7.63 – 7.56 (m, 2H), 7.53 – 7.47 (m, 1H), 6.33 (s, 1H), 3.11 (s, 3H), 2.34 – 2.09 (m, 2H), 1.37 (s, 9H), 1.21 – 1.17 (m, 2H), 0.96 (t, J = 7.3 Hz, 3H).; **^{13}C NMR** (101MHz, CDCl3) δ 172.2, 155.3, 154.4, 137.0, 131.4 (q, $J_{C\text{-}F}$ = 32.3 Hz), 130.3, 126.6 (q, $J_{C\text{-}F}$ = 272.4 Hz), 125.73 (q, $J_{C\text{-}F}$ = 3.2 Hz), 123.54 (q, $J_{C\text{-}F}$ = 3.5 Hz), 82.8, 71.3, 35.9, 27.9, 25.2, 16.9, 14.0; HRMS (ESI) calcd for $[C_{19}H_{24}F_3N_3O_4Na]^+$ requires [M + Na]$^+$ 438.1617, found 438.1611

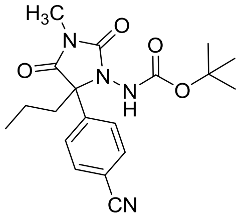

***tert*-Butyl (5-(4-cyanophenyl)-3-methyl-2,4-dioxo-5-propylimidazolidin-1-yl)carbamate (7s)**; (1-methoxypent-1-en-1-yl)oxy)trimethylsilane (67 mg, 0.35 mmol,) was added dropwise to a mixture of tert-butyl (*E*)-2-((4-cyanophenyl)(methyl)carbamoyl)diazene-1-carboxylate **5c** (103 mg, 0.35 mmol) and AgOTf (9.0 mg, 10 mol %) in THF (3.5 mL, 0.1 M) at -78 °C. The reaction was warmed to RT after 10 minutes and stirred at RT for 2.5 h. After TLC showed consumption of tert-butyl (*E*)-2-((4-cyanophenyl)(methyl)carbamoyl)diazene-1-carboxylate **5c**, the reaction was cooled to -78 °C and KHMDS (1 M in THF, 3 equiv) was added dropwise. After 10 minutes the reaction was warmed to -20 °C and stirred at -20 °C for 2 h. The reaction was quenched with sat. aq. NH_4Cl (10 mL), and the aqueous layer was extracted with EtOAc (3 × 10 mL). The combined organics were washed with brine (1 × 10 mL), dried over $MgSO_4$ and concentrated under reduced pressure to give a crude residue. Purification by flash silica

chromatography (20% EtOAC + Hexane) afforded the title compound (80 mg, 60%) as a colorless semi-solid.

***V*max /cm^{-1}(neat):** 3293, 2923, 2230, 1788, 1711, 1454, 1246, 1156, 843, 752

^{1}H NMR (400 MHz, $CDCl_3$) δ 7.65 (d, *J* = 8.3 Hz, 2H), 7.56 (d, *J* = 8.3 Hz, 2H), 6.44 (s, 1H), 3.09 (s, 3H), 2.19 (dtd, *J* = 55.3, 14.1, 4.2 Hz, 2H), 1.38 (s, 9H), 1.25-121 (m, 2H), 0.94 (t, *J* = 7.3 Hz, 3H); **^{13}C NMR** (101MHz, CDCl3) δ 171.8, 155.4, 154.5, 141.0, 132.5, 127.7, 118.2, 112.8, 82.9, 71.3, 35.9, 27.9, 25.2, 16.9, 14.0; HRMS (ESI) calcd for $[C_{19}H_{24}N_4O_4Na]^+$ requires $[M + Na]^+$ 395.1695, found 395.1699

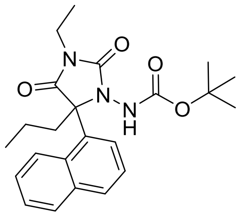

***tert*-Butyl (3-ethyl-5-(naphthalen-1-yl)-2,4-dioxo-5-propylimidazolidin-1-yl)carbamate (7t)**; (1-methoxypent-1-en-1-yl)oxy)trimethylsilane (78 mg, 0.41 mmol,) was added dropwise to a mixture of tert-butyl (E)-2-(ethyl(naphthalen-1-yl)carbamoyl)diazene-1-carboxylate **5i** (136 mg, 0.41 mmol) and AgOTf (10.4 mg, 10 mol %) in THF (4.1 mL, 0.1 M) at -78 ºC. The reaction was warmed to RT after 10 minutes and stirred at RT for 3 h. After TLC showed consumption of tert-butyl (*E*)-2-(ethyl(naphthalen-1-yl)carbamoyl) diazene-1-carboxylate **5i**, the reaction was cooled to -78 ºC and KHMDS (1 M in THF, 3 equiv) was added dropwise. After 10 minutes the reaction was warmed to -20 ºC and stirred at -20 ºC for 3 h. The reaction was quenched with sat. aq. NH_4Cl (10 mL), and the aqueous layer was extracted with EtOAc (3 × 10 mL). The combined organics were washed with brine (1 × 10 mL), dried over $MgSO_4$ and concentrated under reduced pressure to give a crude residue. Purification by flash silica chromatography (15% EtOAC + Hexane) afforded the title compound (107 mg, 62%) as colourless needles.

MP: 170-172 ºC

***V*max /cm^{-1}(neat):** 3274, 2972, 1783, 1708, 1449, 1351, 1159, 731

^{1}H NMR (400 MHz, $CDCl_3$) δ 7.82 (d, *J* = 7.8 Hz, 2H), 7.66 (d, *J* = 7.3 Hz, 1H), 7.49 (d, *J* = 8.0 Hz, 1H), 7.44 – 7.34 (m, 3H), 5.76 (s, 1H), 3.75 (q, *J* = 7.2 Hz, 2H), 2.44 (td, *J* = 12.9, 4.2 Hz, 1H), 2.32 (dt, *J* = 12.5, 8.1 Hz, 1H), 1.49 – 1.05 (m, 14H), 0.98 (t, *J* = 7.2 Hz, 3H); **^{13}C NMR** (101MHz, CDCl3) δ ^{13}C NMR (101 MHz, $CDCl_3$) δ 172.9, 155.1, 154.4, 134.5, 131.2, 130.7, 130.0, 129.8, 128.0, 127.1, 125.7, 124.9, 122.3, 82.2, 70.7, 37.2, 34.3, 27.7, 16.4, 14.2, 13.1.; HRMS (ESI) calcd for $[C_{23}H_{29}N_3O_4Na]^+$ requires $[M + Na]^+$ 434.2056, found 434.2058

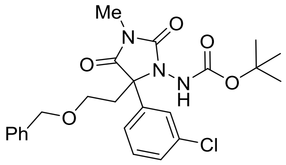

***tert*-Butyl (5-(2-(benzyloxy)ethyl)-5-(3-chlorophenyl)-3-methyl-2,4-dioxoimidazolidin-1-yl)carbamate (7u)**; (4-(benzyloxy)-1-methoxybut-1-en-1-yl)oxy)trimethylsilane (145 mg, 0.51 mmol,) was added dropwise to a mixture of (E)-tert-butyl 2-((3-chlorophenyl)(methyl)carbamoyl)diazenecarboxylate **5d** (154 mg, 0.51 mmol) and AgOTf (13.0 mg, 10 mol %) in THF (5.1 mL, 0.1 M) at -78 ºC. The reaction was warmed to RT after 10 minutes and stirred at RT for 2 h. After TLC showed consumption of (*E*)-tert-butyl 2-((3-chlorophenyl)(methyl)carbamoyl)diazenecarboxylate **5d**, the reaction was cooled to -78 ºC and KHMDS (1 M in THF, 3 equiv) was added dropwise. After 10 minutes the reaction was warmed to -20 ºC and stirred at -20 ºC for 1.5 h. The reaction was quenched with sat. aq. NH_4Cl (10 mL), and the aqueous layer was extracted with EtOAc (3 × 10 mL). The combined organics were washed with brine (1 × 10 mL), dried over $MgSO_4$ and concentrated under reduced pressure to give a crude residue. Purification by flash silica chromatography (15% EtOAC + Hexane) afforded the title compound (215 mg, 87%) as a colorless oil.

***V*max /cm^{-1}(neat):** 3310, 2978, 2933, 1791, 1714, 1453, 1157

^{1}H NMR (400 MHz, $CDCl_3$) δ 7.69–7.57 (m, 2H), 7.38 – 7.28 (m, 7H), 6.82 (s, 1H), 4.53 – 4.34 (m, 2H), 3.62–3.39 (m, 2H), 2.96 (s, 3H), 2.54 (br s, 2H), 1.41 (s, 9H);**^{13}C NMR** (101MHz, CDCl3) δ ^{13}C NMR (101 MHz, $CDCl_3$) δ 172.0, 156.1, 154.7, 138.4, 136.6, 130.0, 128.9, 128.6, 128.3, 126.9, 124.9, 81.9, 74.2, 70.4, 66.0, 34.4, 28.0, 25.1; HRMS (ESI) calcd for $[C_{24}H_{28}ClN_3O_5Na]^+$ requires $[M + Na]^+$ 496.1615, found 496.1625

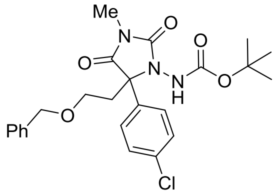

***tert*-Butyl (5-(2-(benzyloxy)ethyl)-5-(4-chlorophenyl)-3-methyl-2,4-dioxoimidazolidin-1-yl)carbamate (7v)**; (4-(benzyloxy)-1-methoxybut-1-en-1-yl)oxy)trimethylsilane (142 mg, 0.47 mmol,) was added dropwise to a mixture of tert-butyl (E)-2-((4-chlorophenyl)(methyl)carbamoyl)diazene-1-carboxylate **5h** (152 mg, 0.47 mmol) and AgOTf (12.8 mg, 10 mol %) in THF (5.0 mL, 0.1 M) at -78 ^{o}C. The reaction was warmed to RT after 10 minutes and stirred at RT for 1.5 h. After TLC showed consumption of tert-butyl (E)-2-((4-chlorophenyl)(methyl)carbamoyl)diazene-1-carboxylate **5h**, the reaction was cooled to -78 ^{o}C and KHMDS (1 M in THF, 3 equiv) was added dropwise. After 10 minutes the reaction was warmed to -20 ^{o}C and stirred at -20 ^{o}C for 1.5 h. The reaction was quenched with sat. aq. NH_4Cl (10 mL), and the aqueous layer was extracted with EtOAc (3 × 10 mL). The combined organics were washed with brine (1 × 10 mL), dried over $MgSO_4$ and concentrated under reduced pressure to give a crude residue. Purification by flash silica chromatography (15% EtOAC + Hexane) afforded the title compound (183 mg, 76%) as a colorless oil.

***V_{max}* /cm^{-1}(neat):** 3310, 2979, 2932, 1790, 1714, 1453, 1156, 1094, 731.

^{1}H NMR (400 MHz, $CDCl_3$) δ 7.63 (br s, 2H), 7.32 (q, *J* = 5.8, 5.2 Hz, 7H), 6.84 (br s, 1H), 4.52 – 4.33 (m, 2H), 3.64–3.35 (m, 2H), 2.96 (br s, 3H), 2.53 (br s, 2H), 1.41 (s, 9H). ;**^{13}C NMR** (101MHz, CDCl3) δ 172.2, 156.3, 154.8, 136.6, 134.8, 128.8, 128.7, 128.6, 128.3,

128.1, 81.9, 74.3, 70.5, 66.0, 34.4, 28.1, 25.0; HRMS (ESI) calcd for $[C_{24}H_{28}Cl_3N_3O_5Na]^+$ requires $[M + Na]^+$ 496.1615, found 496.1623

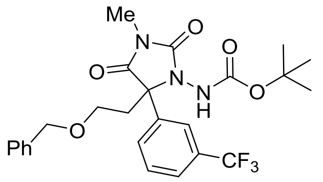

***tert*-Butyl (5-(2-(benzyloxy)ethyl)-3-methyl-2,4-dioxo-5-(3-(trifluoromethyl)phenyl) imidazolidin-1-yl)carbamate (7w)**; 4-(benzyloxy)-1-methoxybut-1-en-1-yl)oxy)trimethyl silane (126 mg, 0.45 mmol,) was added dropwise to a mixture of tert-butyl (*E*)-2-(methyl(3-(trifluoromethyl)phenyl)carbamoyl)diazene-1-carboxylate **5e** (150 mg, 0.45 mmol) and AgOTf (11.0 mg, 10 mol %) in THF (4.5 mL, 0.1 M) at -78 ^{o}C. The reaction was warmed to RT after 10 minutes and stirred at RT for 2 h. After TLC showed consumption of tert-butyl (E)-2-(methyl(3-(trifluoromethyl)phenyl)carbamoyl)diazene-1-carboxylate **5e**, the reaction was cooled to -78 ^{o}C and KHMDS (1 M in THF, 3 equiv) was added dropwise. After 10 minutes the reaction was warmed to -20 ^{o}C and stirred at -20 ^{o}C for 1.5 h. The reaction was quenched with sat. aq. NH_4Cl (10 mL), and the aqueous layer was extracted with EtOAc (3 × 10 mL). The combined organics were washed with brine (1 × 10 mL), dried over $MgSO_4$ and concentrated under reduced pressure to give a crude residue. Purification by flash silica chromatography (15% EtOAC + Hexane) afforded the title compound (160 mg, 69%) as a colorless oil.

***V*$_{max}$ /cm^{-1}(neat):** 3314, 2979, 2869, 1792, 1716, 1328, 1159, 1124.

^{1}H NMR (400 MHz, $CDCl_3$) δ 8.00 (d, *J* = 61.1 Hz, 2H), 7.58 (d, *J* = 7.6 Hz, 1H), 7.48 (t, *J* = 7.9 Hz, 1H), 7.34 (dt, *J* = 14.6, 7.2 Hz, 5H), 6.91 (s, 1H), 4.52 – 4.36 (m, 2H), 3.61–3.41 (m, 2H), 2.96 (s, 3H), 2.57 (br s, 2H), 1.41 (s, 9H);**^{13}C NMR** (101MHz, CDCl3) δ 171.9, 156.2, 154.8, 137.6, 136.5, 131.1 (q, *J* = 32.5 Hz), 130.6, 130.2, 129.2, 128.7, 128.6, 128.3, 125.5,

124.03 (d, J = 272.6 Hz) 123.6, 82.0, 74.3, 70.6, 66.0, 34.9, 28.0, 25.1; HRMS (ESI) calcd for $[C_{25}H_{28}F_3N_3O_5Na]^+$ requires $[M + Na]^+$ 530.1879, found 530.1868

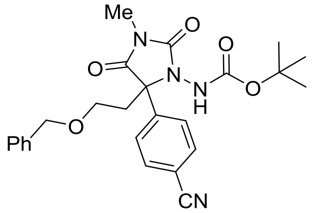

***tert*-Butyl (5-(2-(benzyloxy)ethyl)-5-(4-cyanophenyl)-3-methyl-2,4-dioxoimidazolidin-1-yl)carbamate (7x)**; (4-(benzyloxy)-1-methoxybut-1-en-1-yl)oxy)trimethylsilane (89 mg, 0.31 mmol,) was added dropwise to a mixture of (E)-tert-butyl 2-((4-cyanophenyl)(methyl)carbamoyl)diazenecarboxylate **5c** (92 mg, 0.31 mmol) and AgOTf (8.05 mg, 10 mol %) in THF (3.1 mL, 0.1 M) at -78 °C. The reaction was warmed to RT after 10 minutes and stirred at RT for 2 h. After TLC showed consumption of (E)-tert-butyl 2-((4-cyanophenyl)(methyl)carbamoyl)diazenecarboxylate **5c**, the reaction was cooled to -78 °C and KHMDS (1 M in THF, 3 equiv) was added dropwise. After 10 minutes the reaction was warmed to -20 °C and stirred at -20 °C for 1.5 h. The reaction was quenched with sat. aq. NH_4Cl (10 mL), and the aqueous layer was extracted with EtOAc (3 × 10 mL). The combined organics were washed with brine (1 × 10 mL), dried over $MgSO_4$ and concentrated under reduced pressure to give a crude residue. Purification by flash silica chromatography (15% EtOAC + Hexane) afforded the title compound (120 mg, 81%) as a colorless oil.

$\boldsymbol{V_{max}}$ **/cm^{-1}(neat):** 3309, 2978, 2932, 2230, 1791, 175, 1453, 1158, 733.

^{1}H NMR (400 MHz, $CDCl_3$) δ 7.90 (d, J = 4.4 Hz, 2H), 7.65 (d, J = 8.8 Hz, 2H), 7.39 – 7.27 (m, 5H), 6.90 (s, 1H), 4.53 – 4.31 (m, 2H), 3.69 – 3.31 (m, 2H), 2.95 (s, 3H), 2.54 (s, 2H), 1.42 (s, 9H).; **^{13}C NMR** (101MHz, CDCl3) δ ^{13}C NMR (101 MHz, $CDCl_3$) δ 171.5, 156.3, 154.9, 141.5, 136.3, 132.4, 128.8, 128.7, 128.4, 127.6, 118.4, 112.6, 82.1, 74.4, 70.8, 65.9, 34.6, 28.1, 25.2; HRMS (ESI) calcd for $[C_{25}H_{28}N_4O_5Na]^+$ requires $[M + Na]^+$ 487.1957, found 4.87.1967

1.16 General procedure 8: benzyl deprotection

***tert*-Butyl (5-(3-chlorophenyl)-5-(2-hydroxyethyl)-3-methyl-2,4-dioxoimidazolidin-1-yl)carbamate (7y)**; To a solution of **7u** 2 (130 g, 0.27 mol) in .2M MeOH was added 10% $Pd(OH)_2/C$ and the suspension was hydrogenated at atmospheric pressure and ambient temperature for 48 h. Filtration of the catalyst and concentration in vacuo afforded of the desired alcohol **7y**, A sample was purified by flash chromatography (hexanes/AcOEt 1:1) to afford pure alcohol **7y** as a clear colorless oil (70 mg 66%)

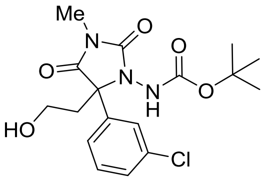

***V*max /cm^{-1}(neat):** 3475, 3298, 2979, 2934, 1787, 1706, 1156

^{1}H NMR (400 MHz, $CDCl_3$) δ 7.56 – 7.39 (m, 2H), 7.39 – 7.28 (m, 3H), 6.94 (s, 1H), 3.93 – 3.79 (m, 1H), 3.75 – 3.53 (m, 1H), 3.08 (s, 4H), 2.52–2.47 (m, 2H), 1.42 (s, 9H); **^{13}C NMR** (100 MHz, CDC13) δ172.2, 156.2, 155.3, 134.9, 130.1, 129.0, 126.8, 124.8, 82.7, 70.5, 58.4, 35.1, 28.0, 25.3 ; HRMS (ESI) calcd for [$C_{17}H_{22}ClN_3O_5Na$] requires [M + Na]+ 406.1146, found 406.1159.

1.17 General Procedure 8: Boc-group deprotection:

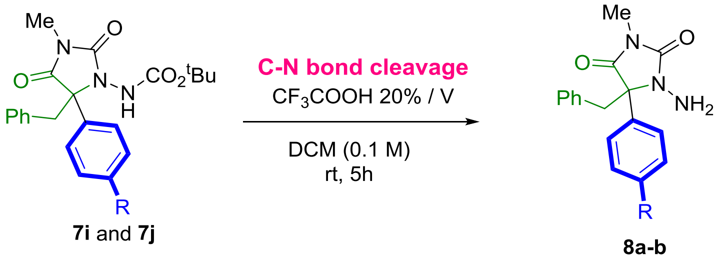

Trifluoroacetic acid (20% by volume) was added dropwise to a solution of **7** in DCM (0.1 M) at 0 °C. The reaction was the stirred at RT for 4 h and concentrated under reduced pressure to give a crude residue. Reaction mixture was diluted in 3 ml DCM and neutralized with Et_3N and again concentrated under reduced pressure. Purification by flash silica chromatography.

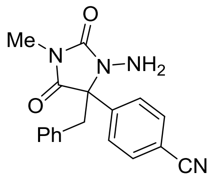

4-(3-Amino-4-benzyl-1-methyl-2,5-dioxoimidazolidin-4-yl)benzonitrile (8a); Trifluoroacetic acid (0.42 mL, 20% by volume) was added dropwise to a solution of tert-butyl (5-benzyl-5-(4-cyanophenyl)-3-methyl-2,4-dioxoimidazolidin-1-yl)carbamate **7i** (86 mg, 0.20 mmol, 1 equiv) in DCM (2.1 mL, 0.1 M) at 0 ºC. The reaction was the stirred at RT for 4 h. and concentrated under reduced pressure to give a crude residue. Reaction mixture was diluted in 3 ml DCM and neutralized with Et_3N and again concentrated under reduced pressure. Purification by flash silica chromatography (30% EtOAC + Hexane) afforded the *title compound* (60 mg, 92%) as colourless needles.

MP: 188-190 ºC

***V*max /cm^{-1}(neat):** 3339, 3270, 2925, 1774, 1710, 1608, 1490, 1454, 1058, 757

^{1}H NMR (400 MHz; $CDCl_3$) δ 7.73 (d, *J* = 8.7 Hz, 2H), 7.63 (d, *J* = 8.8 Hz, 2H), 7.31 – 7.26 (m, 3H), 7.17 (dd, *J* = 7.2, 2.3 Hz, 2H), 3.95 (s, 2H), 3.66 (d, *J* = 13.6 Hz, 1H), 3.49 (d, *J* = 13.5 Hz, 1H), 2.77 (s, 3H); **^{13}C NMR** (101 MHz, $CDCl_3$) δ 171.7, 157.0, 141.9, 132.8, 132.75, 129.4, 128.9, 128.1, 127.3, 118.2, 112.8, 72.8, 40.3, 24.9; **HRMS** (ESI) calcd for $[C_{18}H_{16}N_4O_2Na]^+$ requires $[M + Na]^+$ 343.1171, found 343.1177

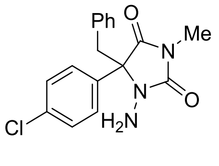

1-Amino-5-benzyl-5-(4-chlorophenyl)-3-methylimidazolidine-2,4-dione (8b); Trifluoroacetic acid (0.25mL, 20% by volume) was added dropwise to a solution of tert-butyl (5-benzyl-5-(4-chlorophenyl)-3-methyl-2,4-dioxoimidazolidin-1-yl)carbamate **7j** (54 mg, 0.12 mmol, 1 equiv) in DCM (1.25 mL, 0.1 M) at 0 ºC. The reaction was the stirred at RT for 4 h

and concentrated under reduced pressure to give a crude residue. Reaction mixture was diluted in 3 ml DCM and neutralized with Et_3N and again concentrated under reduced pressure. Purification by flash silica chromatography (30% EtOAC + Hexane) afforded the *title compound* (32 mg, 78%) as colourless needles.

MP: 219-221 °C

V_{max} **/cm^{-1}(neat):** 3276, 3240, 2987, 1771, 1710, 1494, 1454, 1057, 769

^{1}H NMR (400 MHz, $CDCl_3$) δ 7.40 –7.39 (m, 3H), 7.29 – 7.24 (m, 4H), 7.16 (dd, *J* = 7.3, 2.1 Hz, 2H), 3.92 (s, 2H), 3.64 (d, *J* = 13.5 Hz, 1H), 3.47 (d, *J* = 13.5 Hz, 1H), 2.75 (s, 3H); **^{13}C NMR** (100 MHz, CDCl3) δ 172.3, 157.0, 135.1, 134.9, 134.2, 129.4, 129.2, 128.8, 127.9, 127.7, 72.6, 39.9, 24.7; HRMS (ESI) calcd for [$C_{17}H_{16}ClN_3O_2Na$] requires [M + Na]+ 352.0829, found 352.0832.

1.18 General Procedure 9: Synthesis of bioactive compound analogues:

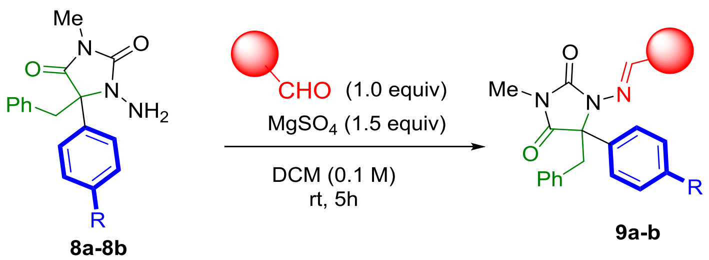

Carbaldehyde (1.0 equiv) was added to a solution of 1-amino- Hydantoins 6 (1 equiv) and MgSO4 (1.5 equiv) in DCM (0.1 M). The resulting suspension was stirred at RT for 6 h, after which the reaction was concentrated under reduced pressure to give a crude residue. Purification by flash silica chromatography.

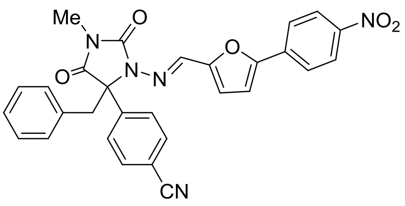

(*E*)-4-(4-Benzyl-1-methyl-3-(((5-(4-nitrophenyl)furan-2-yl)methylene)amino)-2,5-dioxoimidazolidin-4-yl)benzonitrile (**9a**); 5-(4-Nitrophenyl)furfural (38 mg, 0.17 mmol, 1 equiv) was added to a solution of 4-(3-amino-4-benzyl-1-methyl-2,5-dioxoimidazolidin-4-yl)benzonitrile **8a** (57 mg, 0.17 mmol, 1 equiv) and MgSO4 (32 mg 1.5 equiv) in DCM (1.7 mL, 0.1 M). The resulting suspension was stirred at RT for 6 h, after which the reaction was concentrated under reduced pressure to give a crude residue. Purification by flash silica chromatography (30% to 100% EtOAc/Petrol) afforded the *title compound* (60 mg, 65%) as yellow needles.

MP: 95-97 ^{o}C

***V*$_{max}$ /cm^{-1}(neat):** 2921, 1770, 1712, 1599, 1514, 1330, 851, 750

^{1}H NMR (400 MHz; $CDCl_3$) δ 9.36 (s, 1H), 8.26 (d, *J* = 9.0 Hz, 2H), 7.85 (d, *J* = 9.0 Hz, 2H), 7.73 (d, *J* = 3.0 Hz, 3H), 7.26 – 7.22 (m, 4H), 7.21 – 7.17 (m, 2H), 7.00 (d, *J* = 3.7 Hz, 1H), 6.96 (d, *J* = 3.7 Hz, 1H), 3.80 – 3.68 (m, 2H), 2.73 (s, 3H). **^{13}C NMR** (101 MHz; $CDCl_3$) ^{13}C NMR (101 MHz, CHLOROFORM-*D*) δ 170.3, 153.6, 152.9, 151.1, 147.1, 141.8, 141.3, 135.4, 133.1, 132.6, 130.1, 128.6, 128.1, 127.6, 125.8, 124.6, 124.5, 118.3, 115.8, 112.7, 111.1, 110.7, 73.2, 41.5, 24.5 **HRMS** (ESI) calcd for [$C_{29}H_{21}N_5O_5Na$] requires [M + Na]$^+$ 542.1440, found 542.1438.

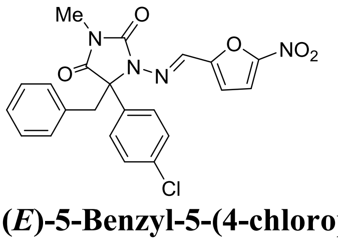

(*E*)-5-Benzyl-5-(4-chlorophenyl)-3-methyl-1-(((5-nitrofuran-2-yl)methylene)amino) imidazolidine-2,4-dione (**9b**); 5-nitrofuran-2-carbaldehyde (12 mg, 0.085 mmol, 1 equiv) was added to a solution of 1-amino-5-benzyl-5-(4-chlorophenyl)-3-methylimidazolidine-2,4-dione **8b** (30 mg, 0.09 mmol, 1 equiv) and MgSO4 (1.5 equiv) in DCM (0.9 mL, 0.1 M). The resulting suspension was stirred at RT for 6 h, after which the reaction was concentrated under reduced

pressure to give a crude residue. Purification by flash silica chromatography (30% to 100% EtOAc/Petrol) afforded the *title compound* (20 mg, 48%) as a yellow semi-solid.

***V*max /cm^{-1}(neat):** 2921, 1772, 1707, 1452, 1347, 1041, 812

^{1}H NMR (400 MHz; $CDCl_3$) δ 9.76 (s, 1H), 7.44 (d, *J* = 8.8 Hz, 1H), 7.34-7.33 (m, 4H), 7.27 (d, *J* = 3.8 Hz, 1H), 7.22 (dd, *J* = 5.1, 1.8 Hz, 3H), 7.11 (dd, *J* = 7.4, 2.0 Hz, 2H), 3.58 (d, *J* = 13.5 Hz, 1H), 3.42 (d, *J* = 13.4 Hz, 1H), 2.70 (s, 3H).**^{13}C NMR** (101 MHz; $CDCl_3$) δ 178.4, 172.3, 157.0, 151.0, 135.2, 134.9, 134.2, 129.4, 129.2, 128.8, 127.9, 127.7, 118.7, 111.7, 72.6, 39.9, 24.7; **HRMS** (ESI) calcd for [$C_{22}H_{17}ClN_4O_5Na$] requires $[M + Na]^+$ 475.0785, found 475.0765.

1.19 General Procedure 10: N-N bond cleavage:

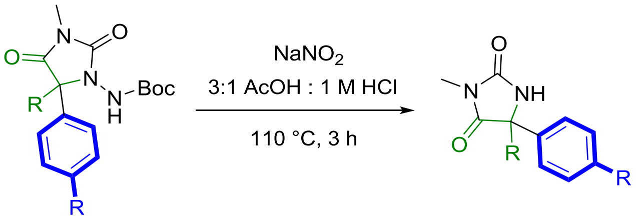

Aminohydantoin (1.0 equiv.) was dissolved in a 3:1 solution of AcOH and 1 M HCl, followed by addition of $NaNO_2$ dissolved in water. The reaction mixture was refluxed at 110 °C for 3 h, and then cooled to room temperature. The reaction mixture was concentrated under reduced pressure and the dry residue was exposed to sat. $NaHCO_3$ (4 mL). The aqueous phase was extracted with EtOAc (3 x 3 mL), the organic extracts were combined and concentrated under reduced pressure to give crude product. The crude material was purified by column chromatography (0% to 100% Et_2O in *n*-pentane) to afford desired product.

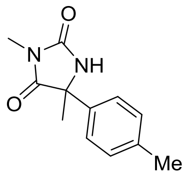

3,5-dimethyl-5-(*p*-tolyl)imidazolidine-2,4-dione (10a);

tert-butyl (3,5-dimethyl-2,4-dioxo-5-(*p*-tolyl)imidazolidin-1-yl)carbamate **7a** (13.9 mg, 0.042 mmol, 1 equiv.) was dissolved in AcOH (1.5 mL), 1 M HCl (0.5 mL) was then added followed by sodium nitrite (9 mg, 0.13 mmol, 3 equiv.) dissolved in water (0.25 mL). The reaction mixture was refluxed at 110 °C for 3 h, and then cooled to room temperature. The reaction mixture was concentrated under reduced pressure and the dry residue was exposed to sat. $NaHCO_3$ (4 mL). The aqueous phase was extracted with EtOAc (3 x 3 mL), the organic extracts were combined and concentrated under reduced pressure to give crude product. The crude material was purified by column chromatography (0% to 100% Et_2O in *n*-pentane) to afford the *title compound* (6.1 mg, 67%) as a fine white powder.

$\boldsymbol{V_{max}}$ **/cm^{-1}(film):** 3292, 2924, 1778, 1778, 1461; **^{1}H NMR** (400 MHz; $CDCl_3$) δ 7.38 (d, J = 8.2 Hz, 2H), 7.19 (d, J = 8.2 Hz, 2H), 6.00 (s, 1H), 3.02 (s, 3H), 2.34 (s, 3H), 1.81 (s, 3H); **^{13}C NMR** (101 MHz, $CDCl_3$) δ 175.4, 156.7, 138.5, 135.6, 129.6, 125.1, 63.6, 25.4, 24.9, 21.0; **HRMS** (ESI) calcd for $[C_{12}H_{15}N_2O_2]$ requires $[M + H]^+$ 219.1128, found 219.1129.

3,5-dimethyl-5-phenylimidazolidine-2,4-dione (10b);

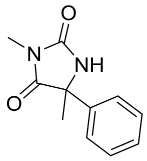

tert-butyl (3,5-dimethyl-2,4-dioxo-5-phenylimidazolidin-1-yl)carbamate **7b** (16.0 mg, 0.05 mmol, 1 equiv.) was dissolved in AcOH (1.5 mL), 1 M HCl (0.5 mL) was then added followed by sodium nitrite (10.4 mg, 0.15 mmol, 3 equiv.) dissolved in water (0.25 mL). The reaction mixture was refluxed at 110 °C for 3 h, and then cooled to room temperature. The reaction mixture was concentrated under reduced pressure and the dry residue was exposed to sat. $NaHCO_3$ (4 mL). The aqueous phase was extracted with EtOAc (3 x 3 mL), the organic extracts were combined and concentrated under reduced pressure to give crude product. The crude material was purified by column chromatography (0% to 100% Et_2O in *n*-pentane) to afford the *title compound* (8.5 mg, 83%) as a fine white powder.

VV_{max} **/cm^{-1}(film):** 3268, 1781, 1710, 1459, 1040; **^{1}H NMR** (400 MHz; $CDCl_3$) δ 7.51 (d, J = 7.5 Hz, 2H), 7.39 (t, J = 7.5 Hz, 2H), 7.36-7.31 (m, 1H), 6.20 (s, 1H), 3.03 (s, 3H), 1.84 (s, 3H); **^{13}C NMR** (101 MHz, $CDCl_3$) δ 175.3, 156.8, 138.6, 128.9, 128.5, 125.2, 63.8, 25.6, 24.9; **HRMS** (+APCI) calcd for [$C_{11}H_{12}N_2O_2$] requires $[M + H]^+$ 205.0972, found 205.0979.

3,5-dimethyl-5-(3-(trifluoromethyl)phenyl)imidazolidine-2,4-dione (10c);

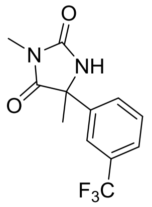

tert-butyl (3,5-dimethyl-2,4-dioxo-5-(3-(trifluoromethyl)phenyl)imidazolidin-1-yl)carbamate **7e** (23.5 mg, 0.06 mmol, 1 equiv.) was dissolved in AcOH (1.5 mL), 1 M HCl (0.5 mL) was then added followed by sodium nitrite (10.4 mg, 0.15 mmol, 2.5 equiv) dissolved in water (0.25 mL). The reaction mixture was refluxed at 110 °C for 3 h, and then cooled to room temperature. The reaction mixture was concentrated under reduced pressure and the dry residue was exposed to sat. $NaHCO_3$ (4 mL). The aqueous phase was extracted with EtOAc (3 x 3 mL), the organic extracts were combined and concentrated under reduced pressure to give crude product. The crude material was purified by column chromatography (0% to 100% Et_2O in *n*-pentane) to afford the *title compound* (12.6 mg, 76%) as a fine white powder.

V_{max} /cm^{-1}(film): 3304, 1782, 1714, 1462, 1330, 1169, 1124; **^{1}H NMR** (400 MHz; $CDCl_3$) δ 7.81-7.73 (m, 2H), 7.64 (d, J = 7.6 Hz, 1H), 7.56 (t, J = 7.6 Hz, 1H), 5.99 (s, 1H), 3.07 (s, 3H), 1.89 (s, 3H); **^{13}C NMR** (101 MHz, $CDCl_3$) δ 174.8, 157.0, 140.0, 131.4 (q, $J_{C\text{-}F}$ = 32.8 Hz), 129.6, 129.1, 125.5 (q, $J_{C\text{-}F}$ = 3.9 Hz), 124.0 (q, $J_{C\text{-}F}$ = 272.5 Hz), 122.2 (q, $J_{C\text{-}F}$ = 3.8 Hz), 63.7, 26.3, 25.1; **^{19}F NMR** (377 MHz, $CDCl_3$) δ -62.5 (s). **HRMS** (ESI) calcd for [$C_{12}H_{11}F_3N_2O_2Na$] requires $[M + Na]^+$ 295.0665, found 295.0675.

^{1}H and ^{13}C NMR Spectra

^{1}H NMR

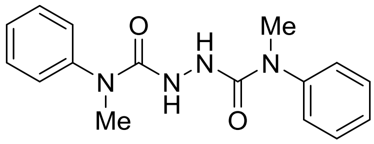

N[1],N[2]-dimethyl-N[1],N[2]-diphenylhydrazine-1,2-dicarboxamide (S1a)

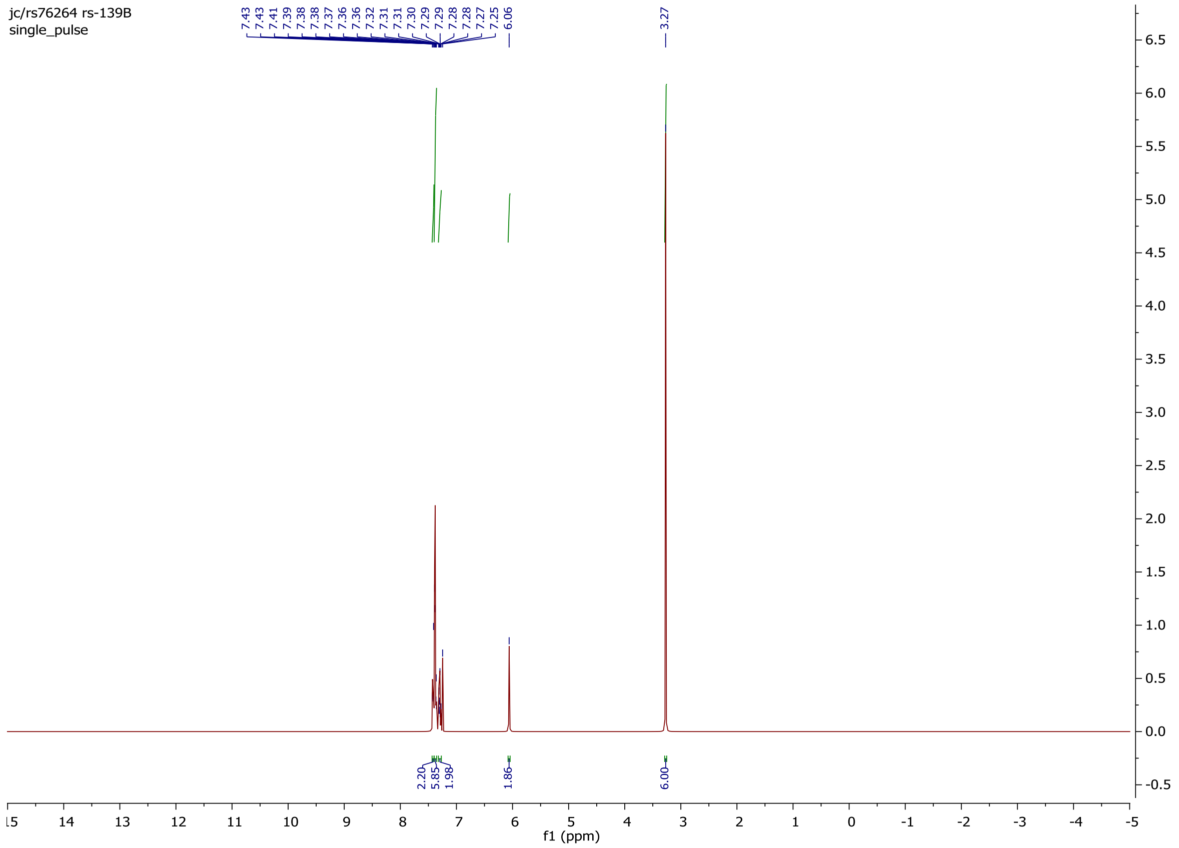

^{13}C NMR

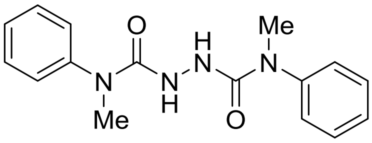

N[1],N[2]-dimethyl-N[1],N[2]-diphenylhydrazine-1,2-dicarboxamide (S1a)

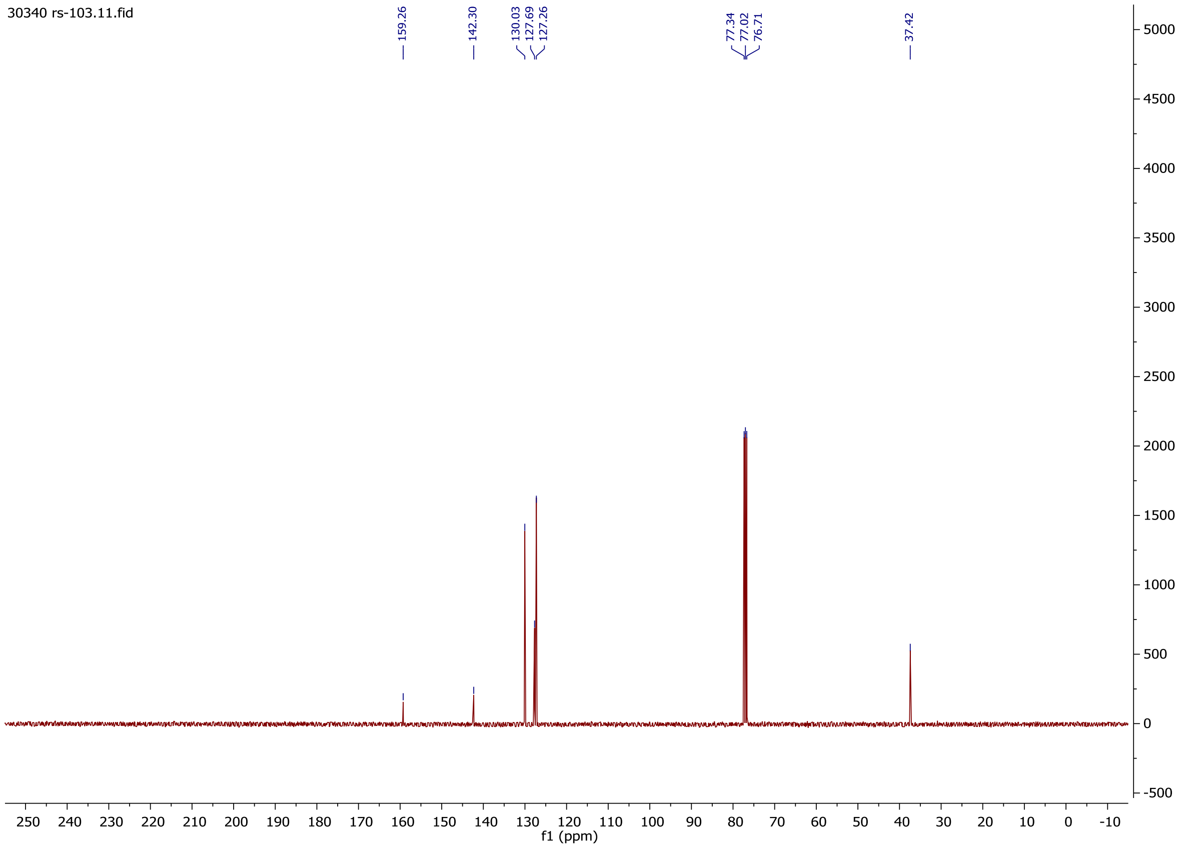

^{1}H NMR

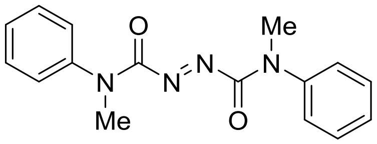

(*E*)-N[1],N[2]-dimethyl-N[1],N[2]-diphenyldiazene-1,2-dicarboxamide (1a)

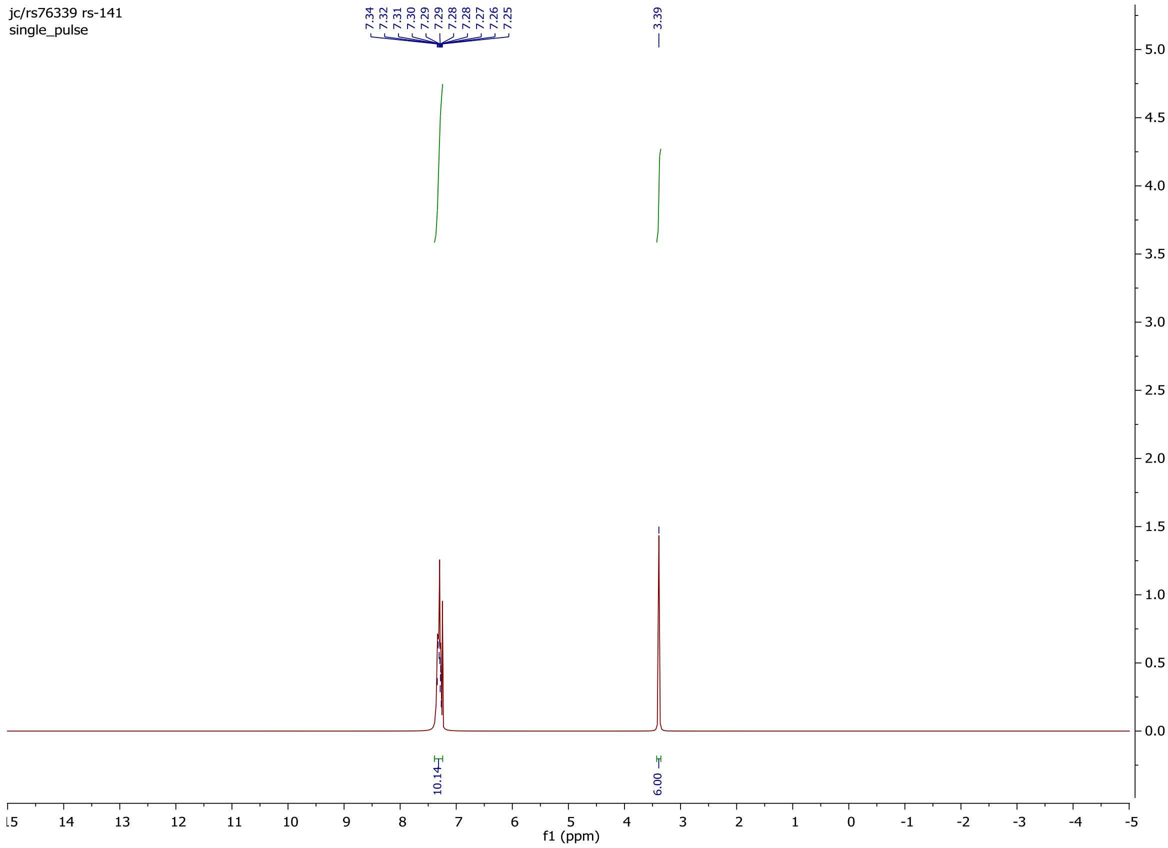

^{13}C NMR

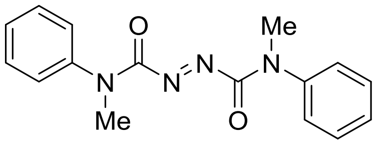

(*E*)-N^1,N^2-dimethyl-N^1,N^2-diphenyldiazene-1,2-dicarboxamide (1a)

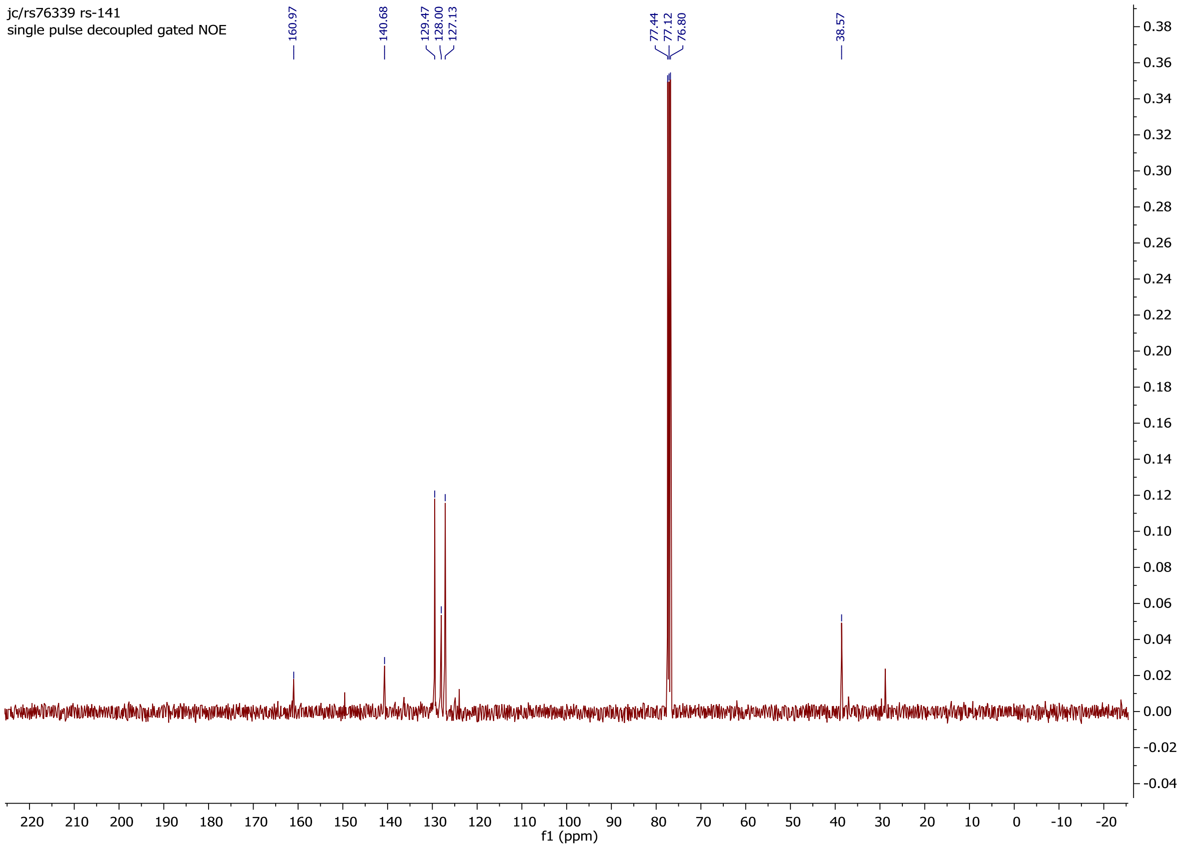

^{1}H NMR

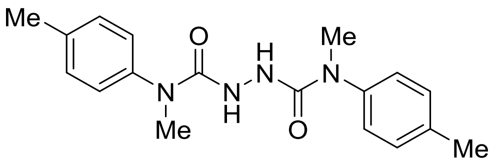

N^{1},N^{2}-dimethyl-N^{1},N^{2}-di-p-tolylhydrazine-1,2-dicarboxamide (S1b)

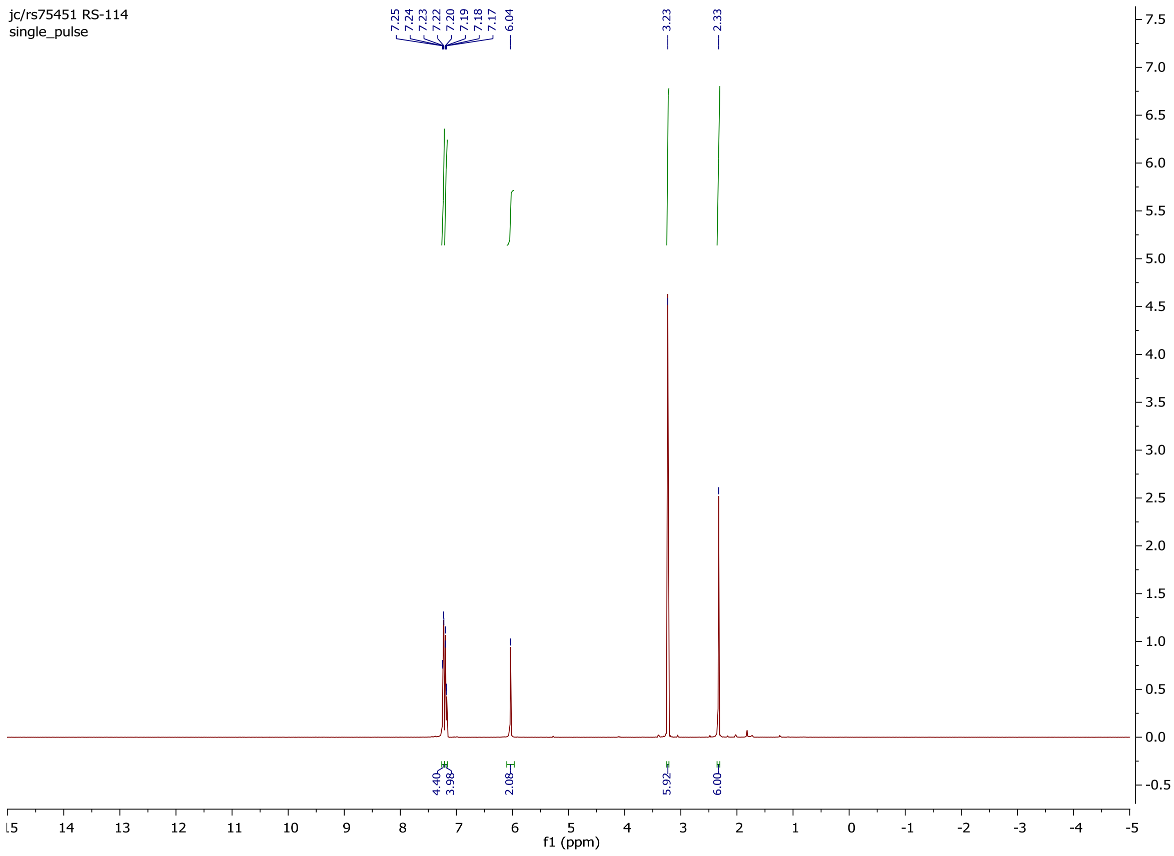

^{13}C NMR

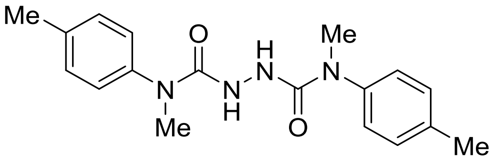

N[1],N[2]-dimethyl-N[1],N[2]-di-*p*-tolylhydrazine-1,2-dicarboxamide (S1b)

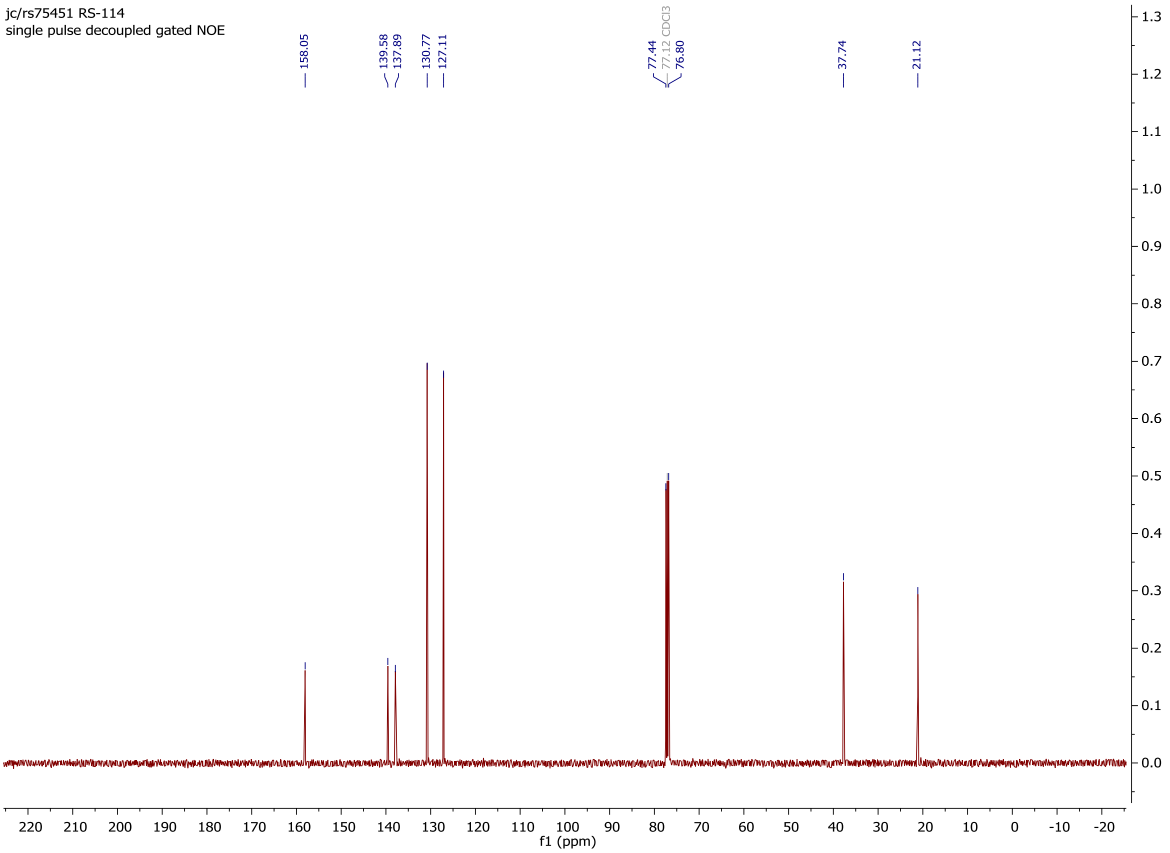

^{1}H NMR

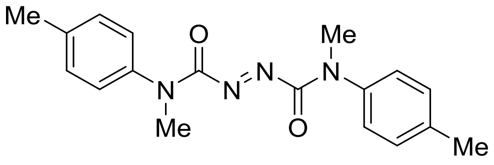

N^{1},N^{2}-dimethyl-N^{1},N^{2}-di-*p*-tolyldiazene-1,2-dicarboxamide (1b)

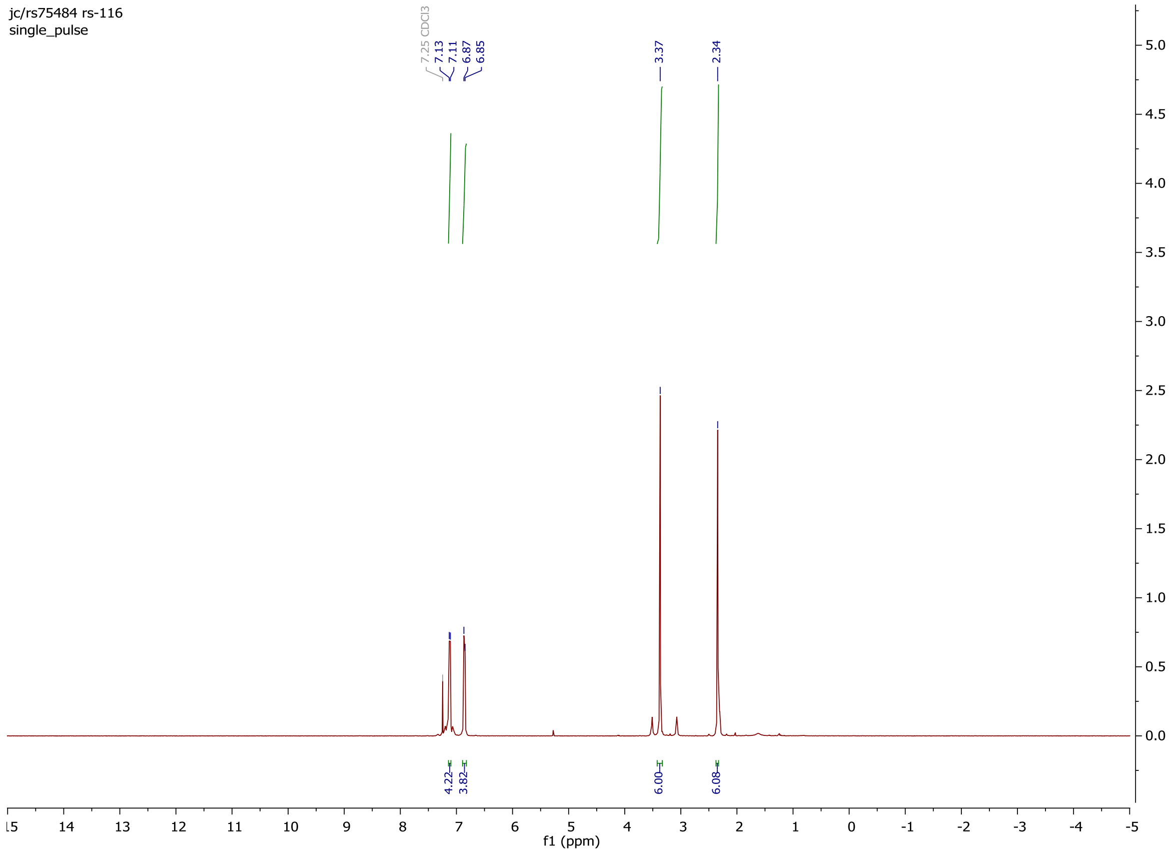

^{13}C NMR

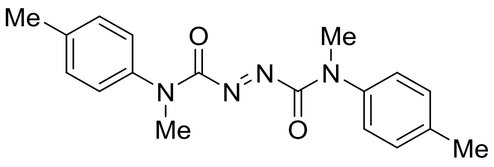

N[1],N[2]-Dimethyl-N[1],N[2]-di-*p*-tolyldiazene-1,2-dicarboxamide (1b)

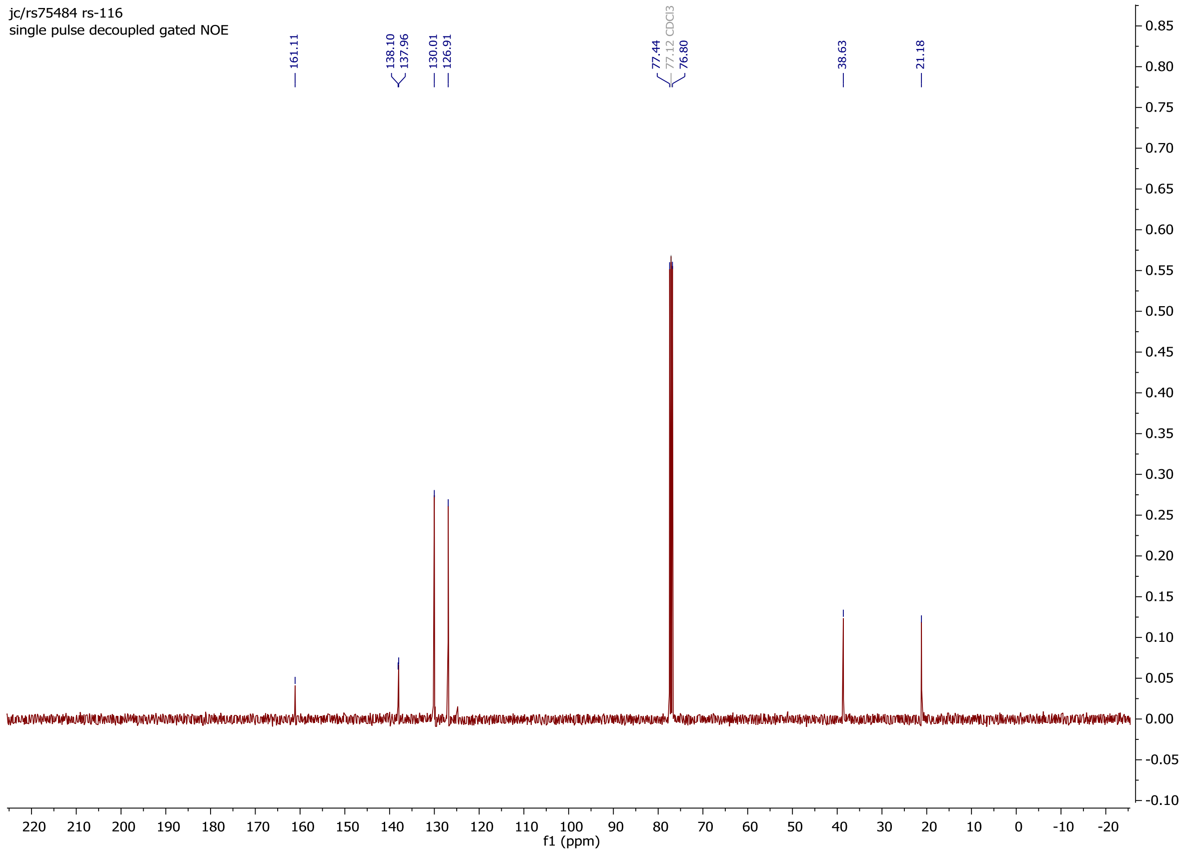

^{1}H NMR

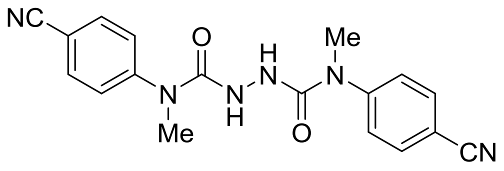

N[1],N[2]-bis(4-cyanophenyl)-N[1],N[2]-dimethylhydrazine-1,2-dicarboxamide (S1c)

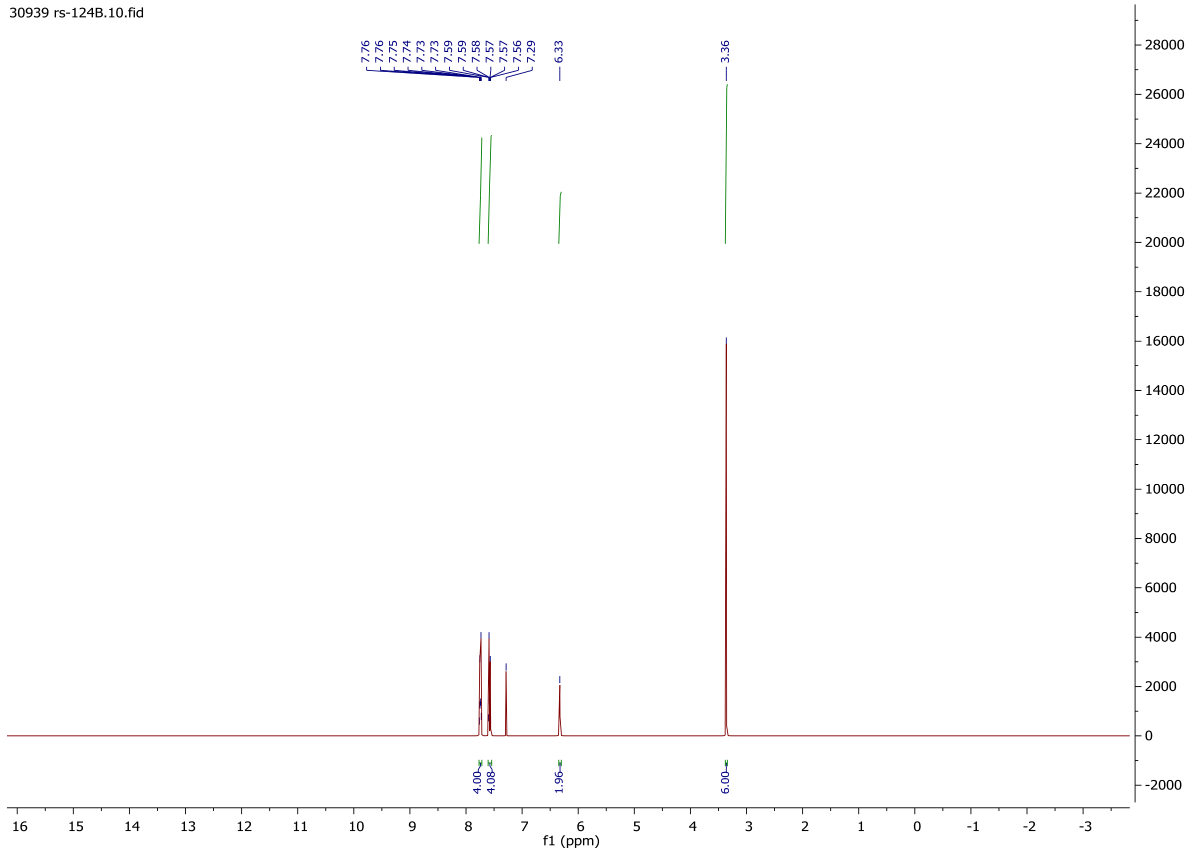

^{13}C NMR

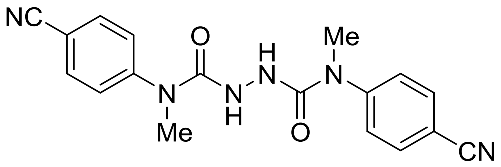

N[1],N[2]-bis(4-cyanophenyl)-N[1],N[2]-dimethylhydrazine-1,2-dicarboxamide (S1c)

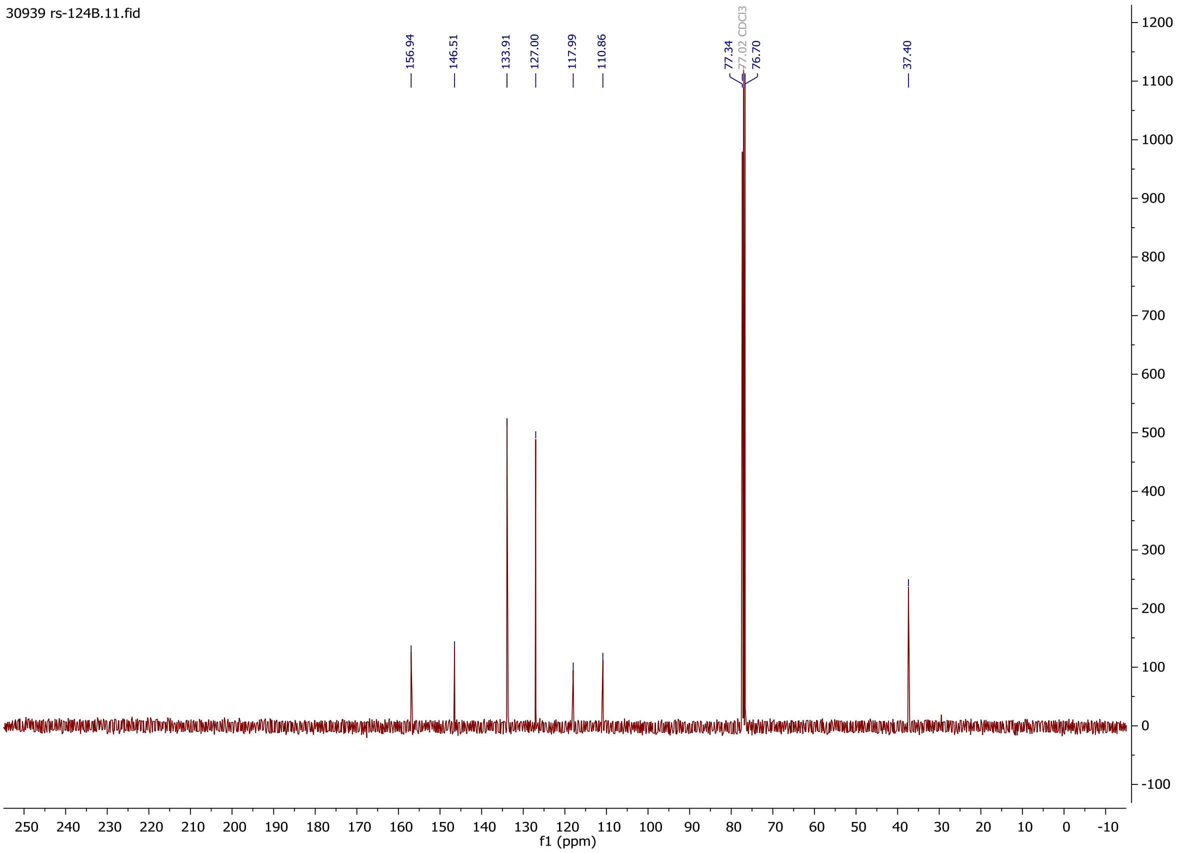

^{1}H NMR

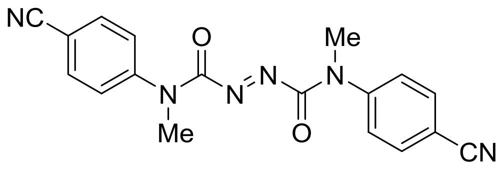

(*E*)-N^{1},N^{2}-bis(4-cyanophenyl)-N^{1},N^{2}-dimethyldiazene-1,2-dicarboxamide (1c)

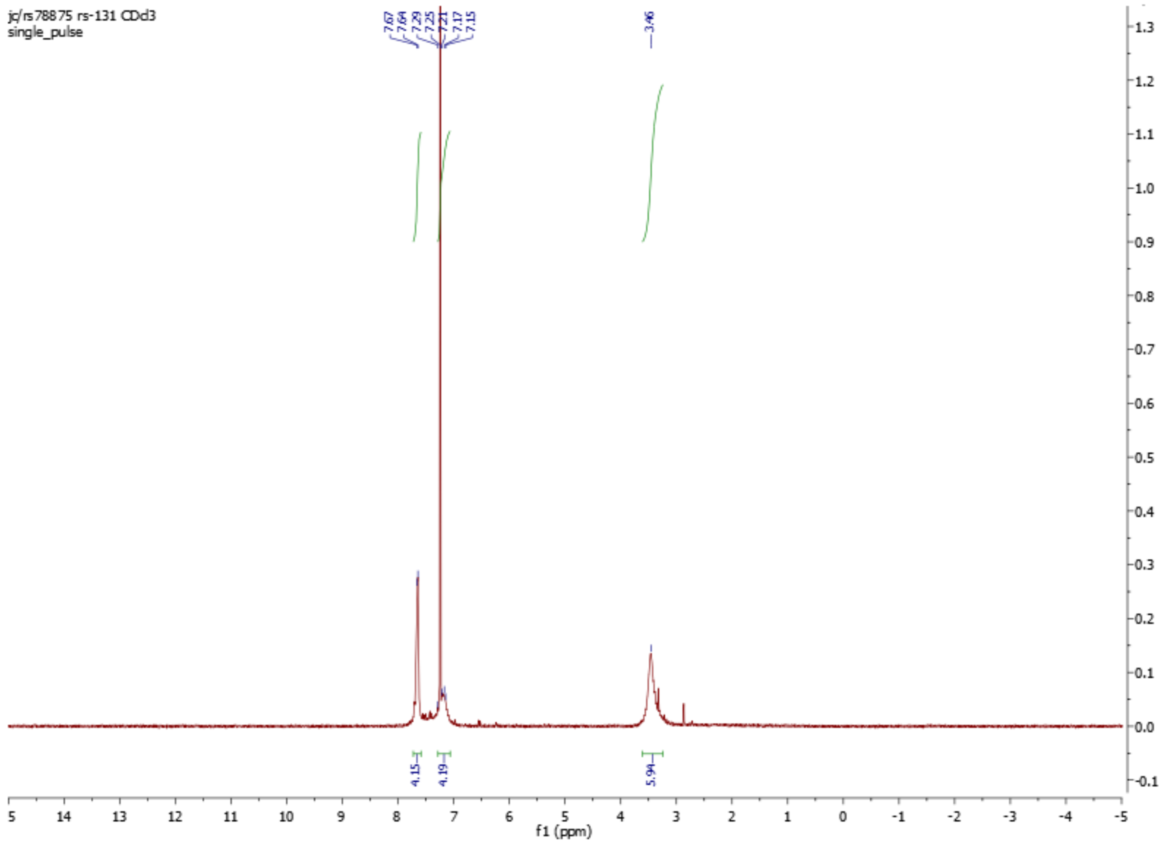

^{13}C NMR

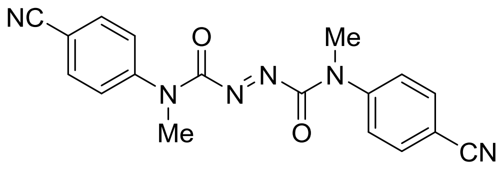

(*E*)-N^{1},N^{2}-bis(4-cyanophenyl)-N^{1},N^{2}-dimethyldiazene-1,2-dicarboxamide (1c)

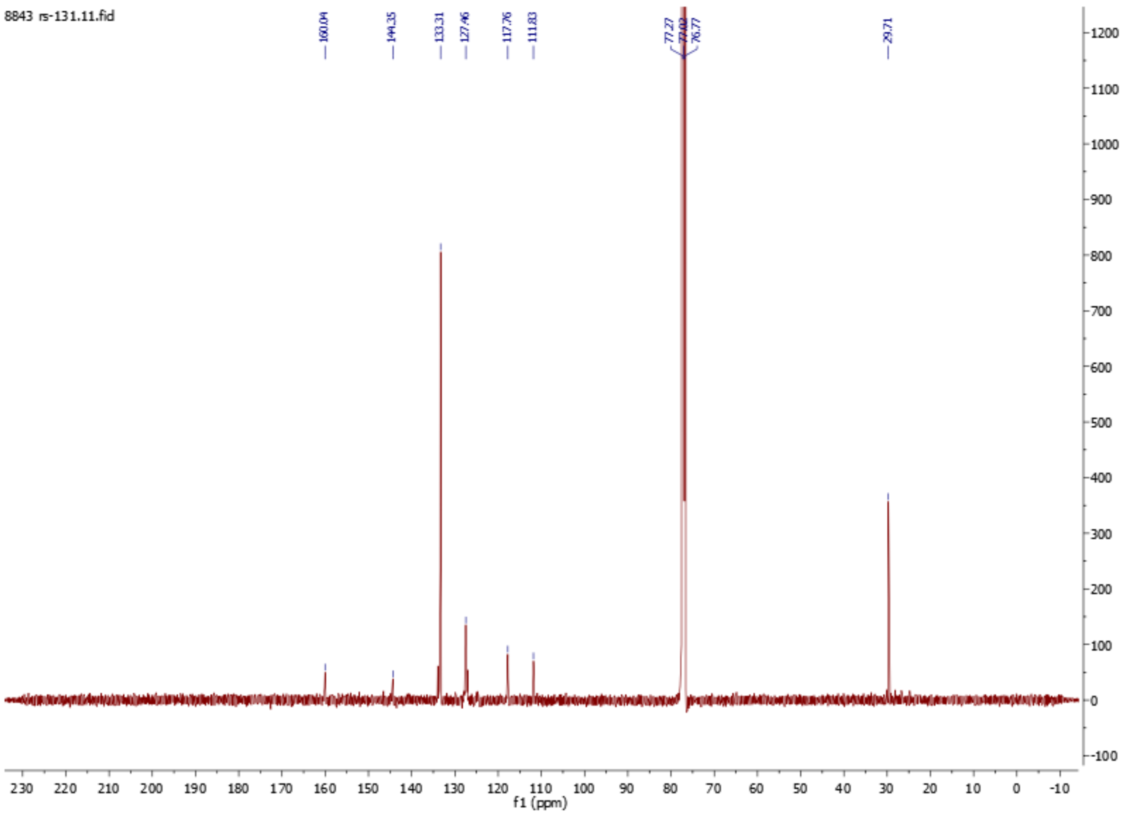

^{1}H NMR

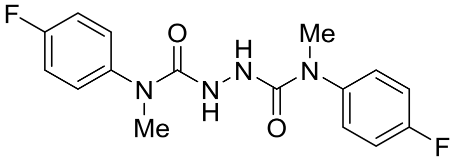

N¹,N²-bis(4-fluorophenyl)-N¹,N²-dimethylhydrazine-1,2-dicarboxamide (S1d)

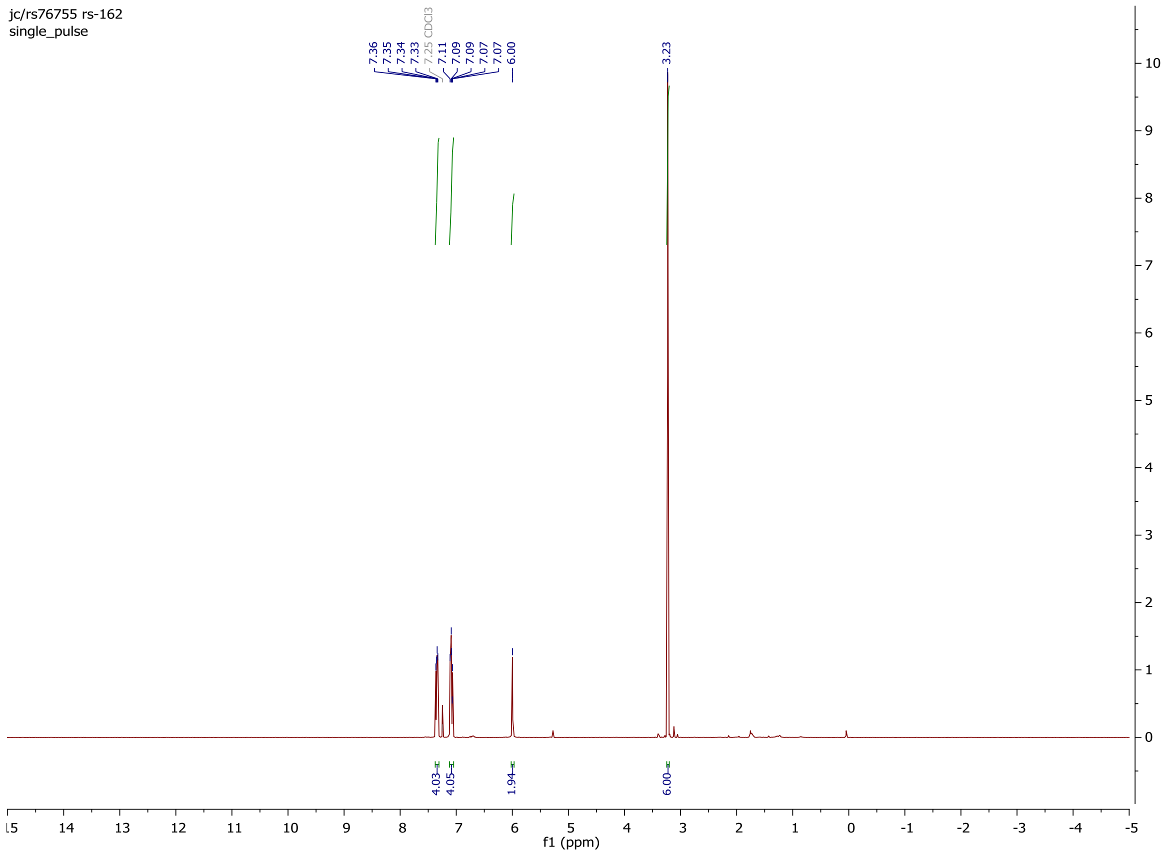

^{13}C NMR

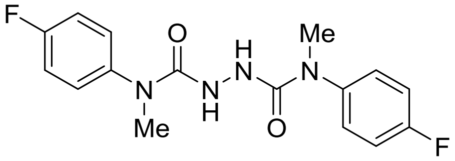

N[1],N[2]-bis(4-fluorophenyl)-N[1],N[2]-dimethylhydrazine-1,2-dicarboxamide (S1d)

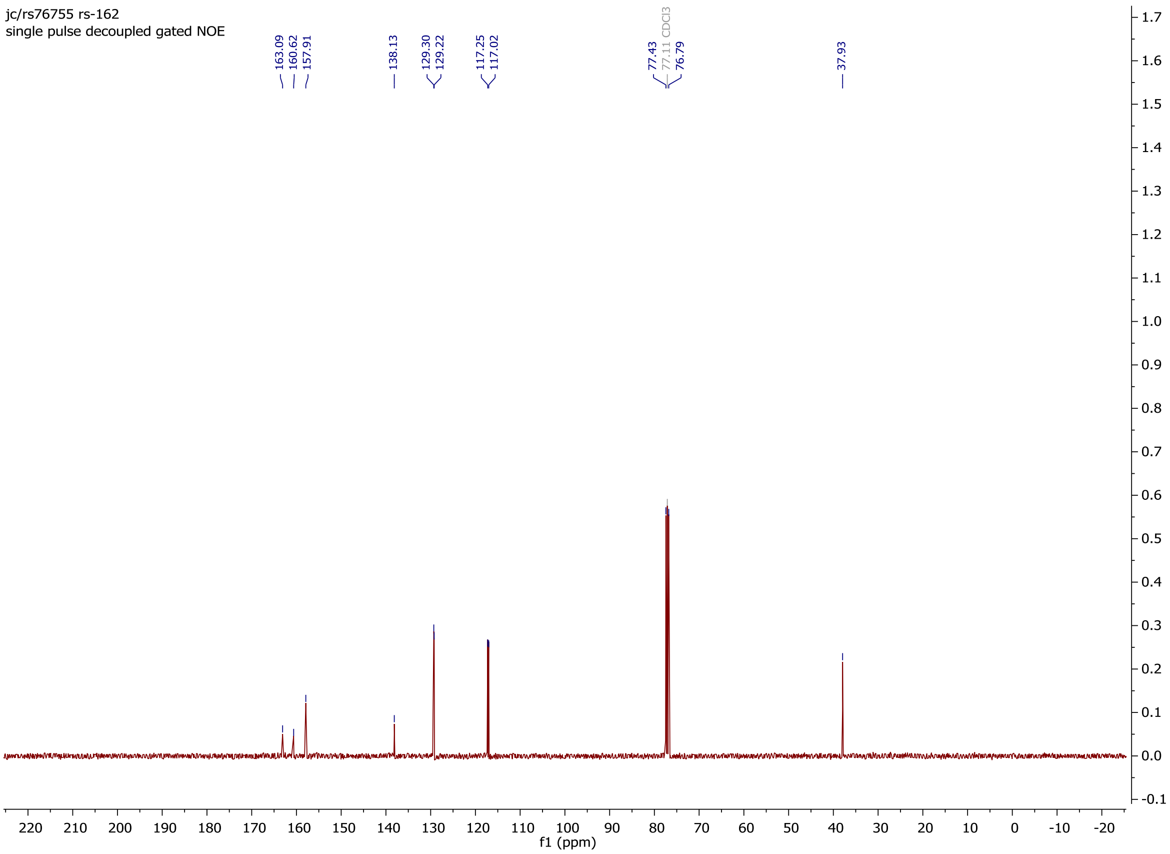

^{1}H NMR

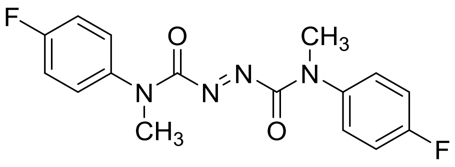

(*E*)-N[1],N[2]-bis(4-fluorophenyl)-N[1],N[2]-dimethyldiazene-1,2-dicarboxamide (1d).

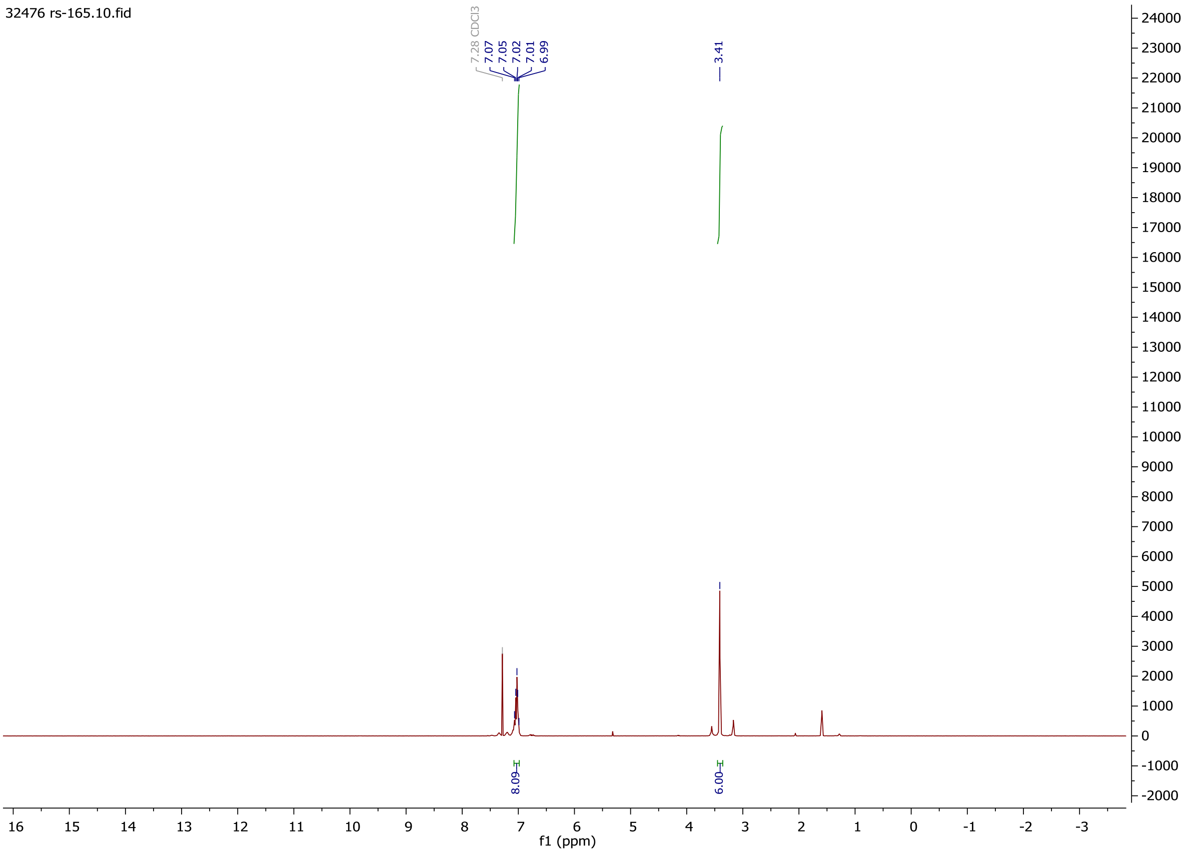

^{13}C NMR

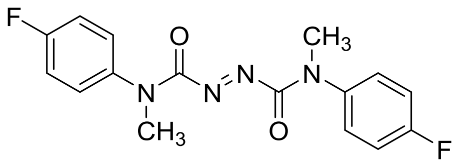

(*E*)-N[1],N[2]-bis(4-fluorophenyl)-N[1],N[2]-dimethyldiazene-1,2-dicarboxamide (1d).

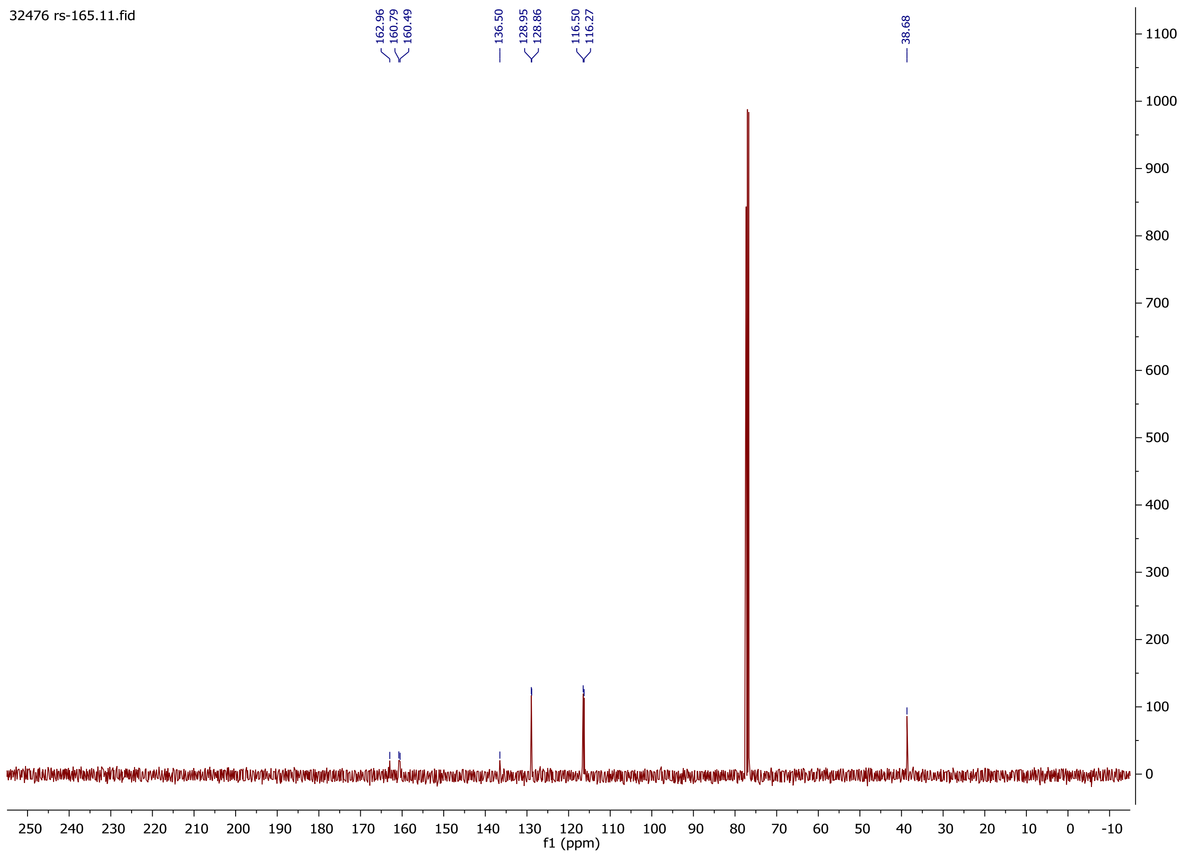

^{1}H NMR

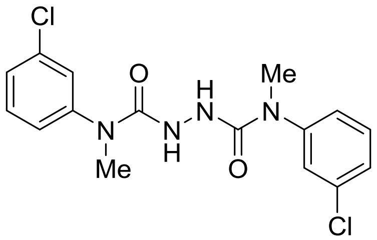

N¹,N²-bis(3-chlorophenyl)-N¹,N²-dimethylhydrazine-1,2-dicarboxamide (S1e)

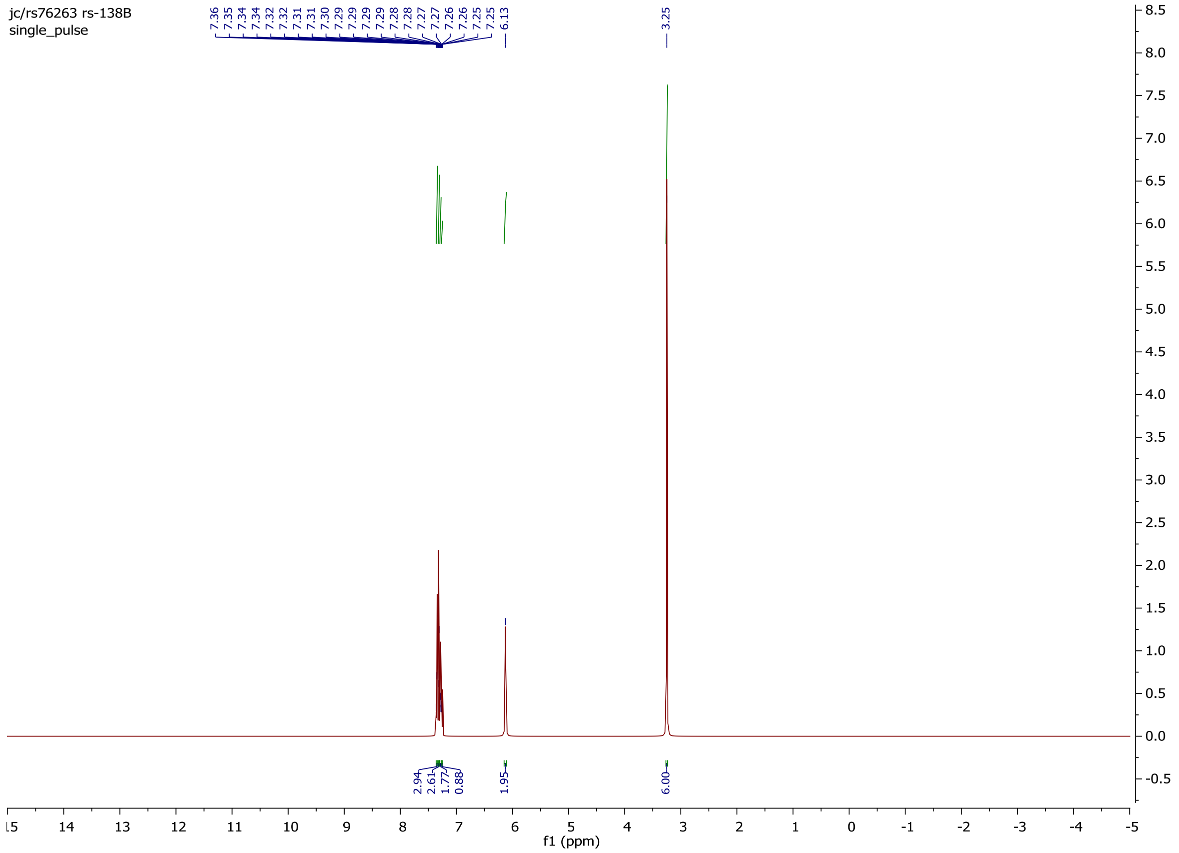

^{13}C NMR

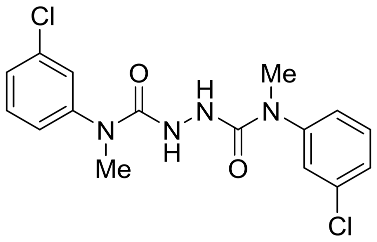

N[1],N[2]-bis(3-chlorophenyl)-N[1],N[2]-dimethylhydrazine-1,2-dicarboxamide (S1e)

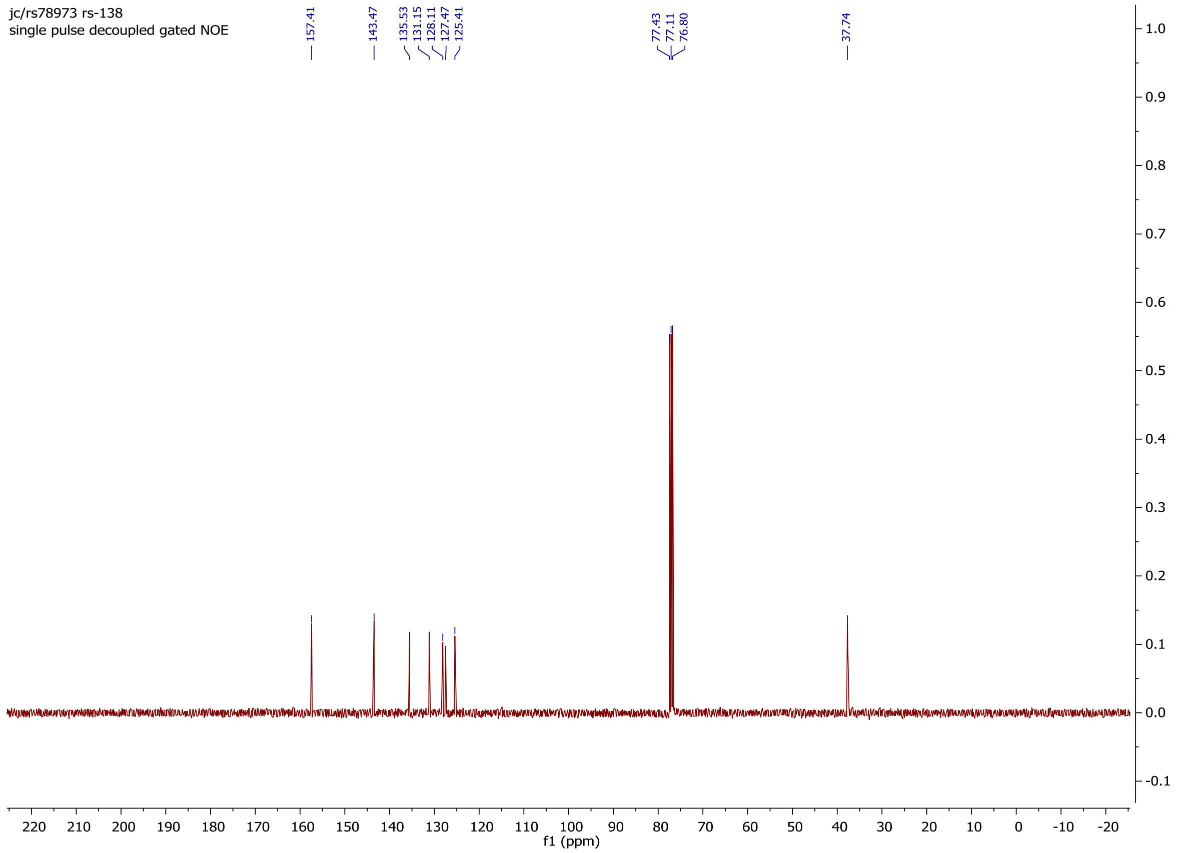

^{1}H NMR

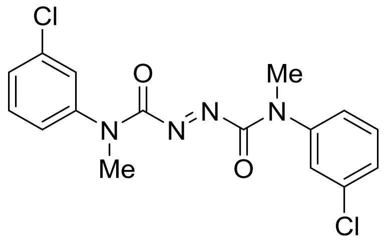

(*E*)-N^1,N^2-bis(3-chlorophenyl)-N^1,N^2-dimethyldiazene-1,2-dicarboxamide (1e)

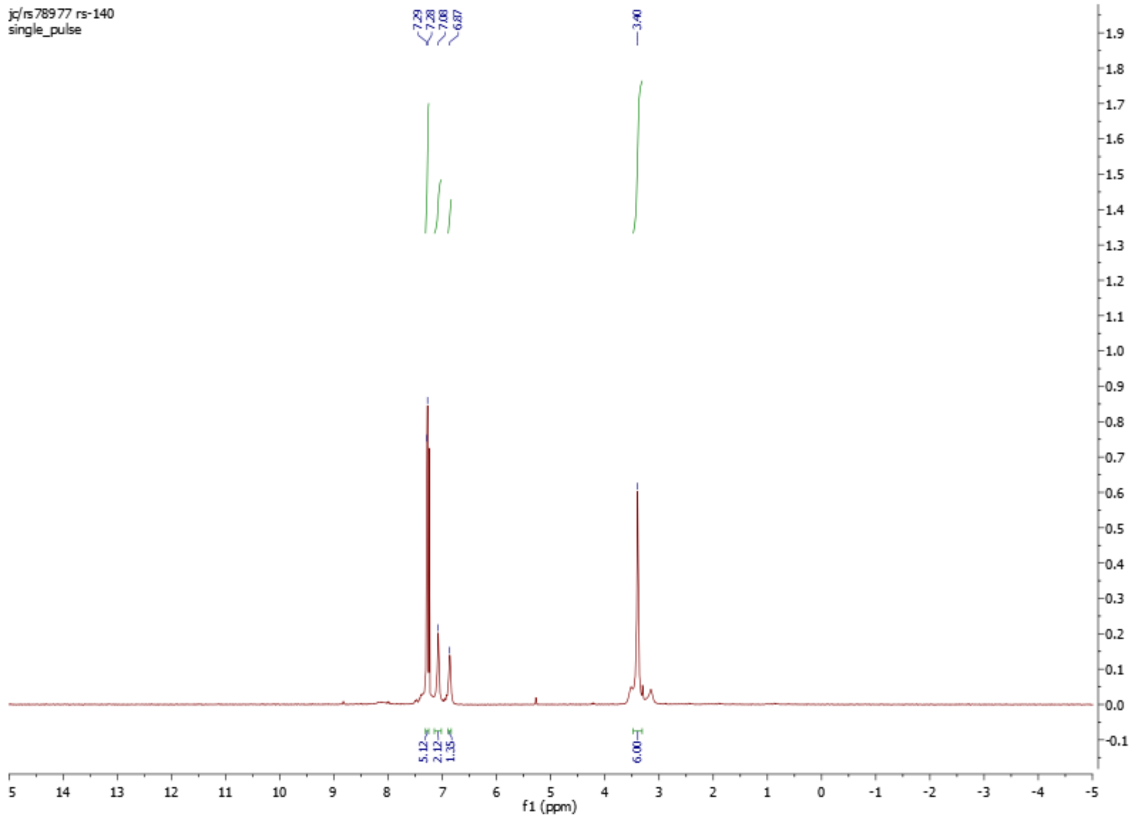

^{13}C NMR

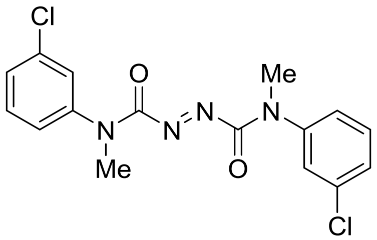

(*E*)-N[1],N[2]-bis(3-chlorophenyl)-N[1],N[2]-dimethyldiazene-1,2-dicarboxamide (1e)

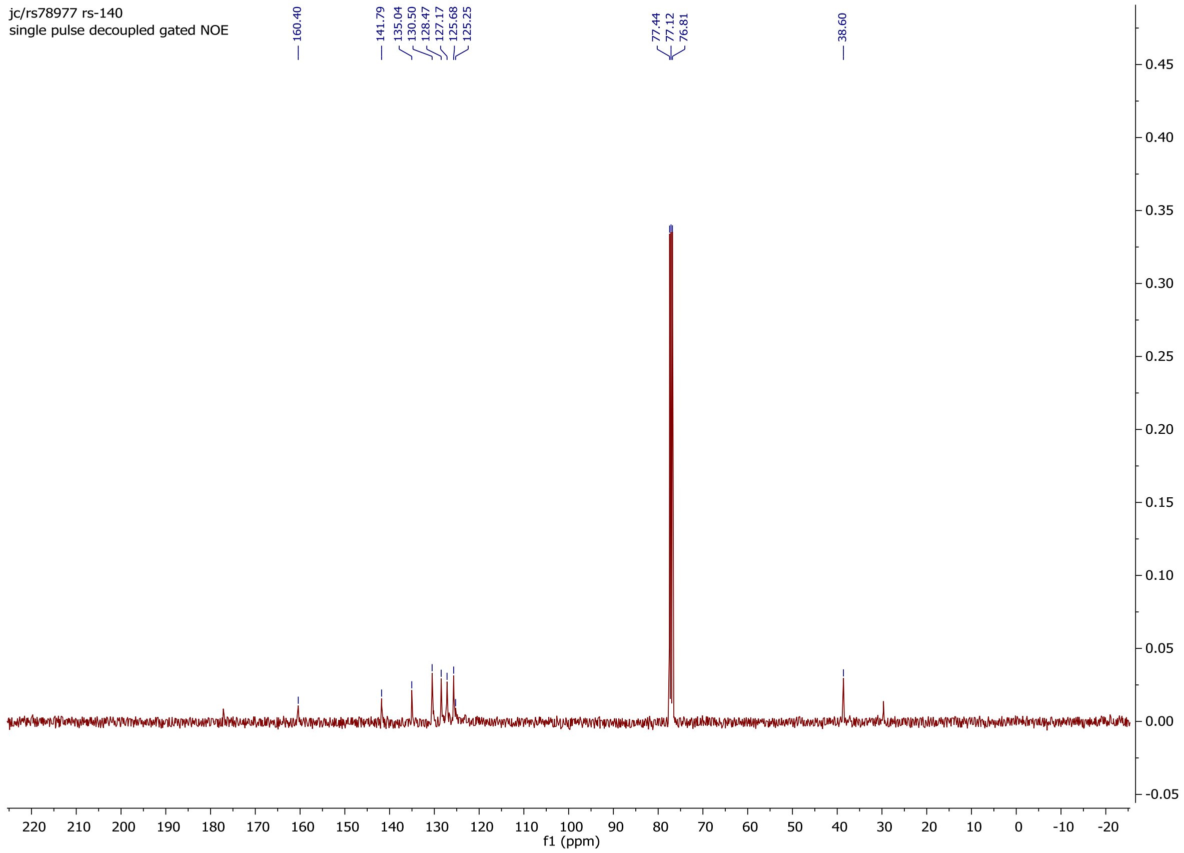

^{1}H NMR

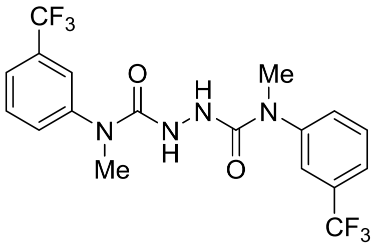

N[1],N[2]-dimethyl-N[1],N[2]-bis(3-(trifluoromethyl)phenyl)hydrazine-1,2-dicarboxamide (S1f)

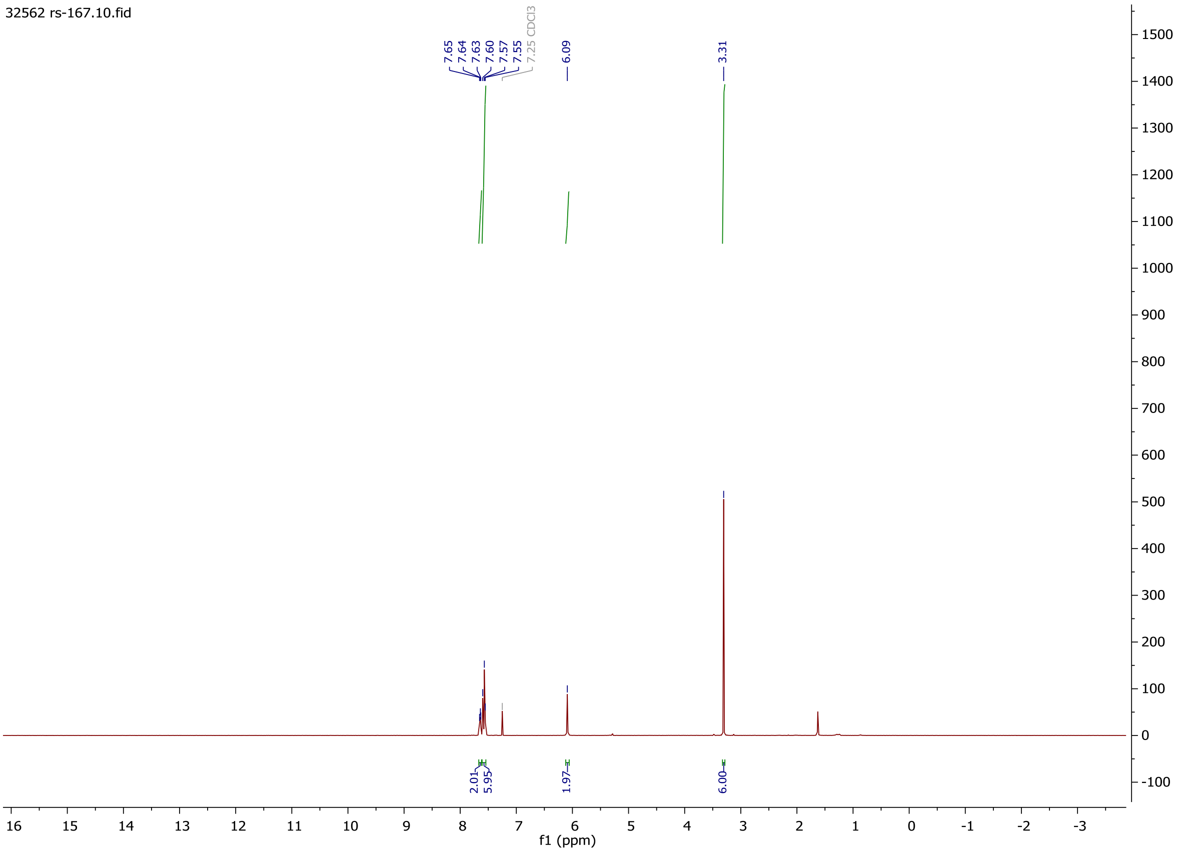

^{13}C NMR

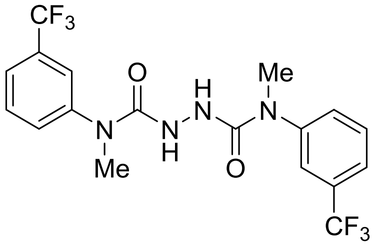

N[1],N[2]-dimethyl-N[1],N[2]-bis(3-(trifluoromethyl)phenyl)hydrazine-1,2-dicarboxamide (S1f)

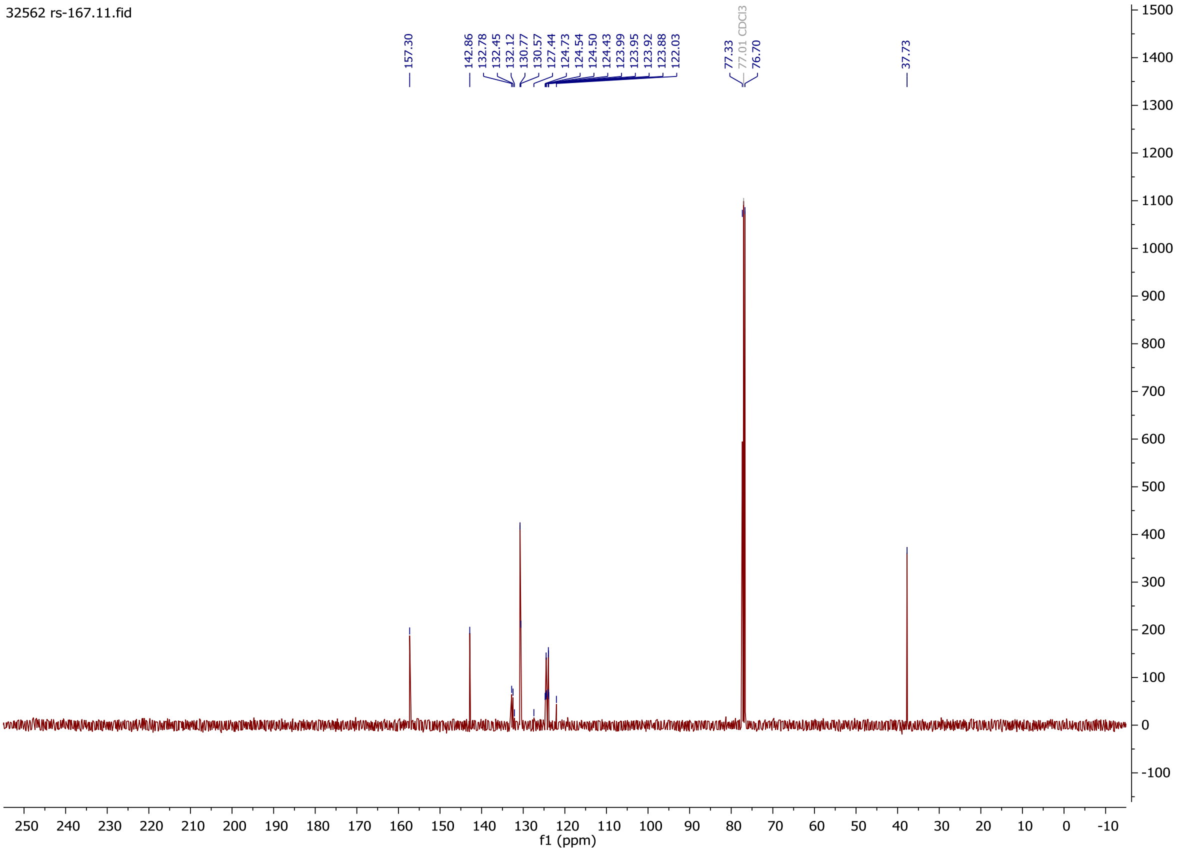

^{1}H NMR

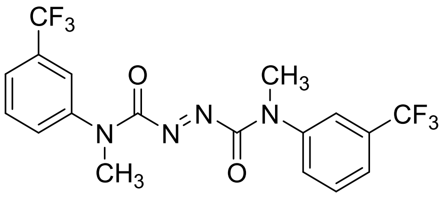

(*E*)-N^1,N^2-dimethyl-N^1,N^2-bis(3-(trifluoromethyl)phenyl)diazene-1,2-dicarboxamide (1f).

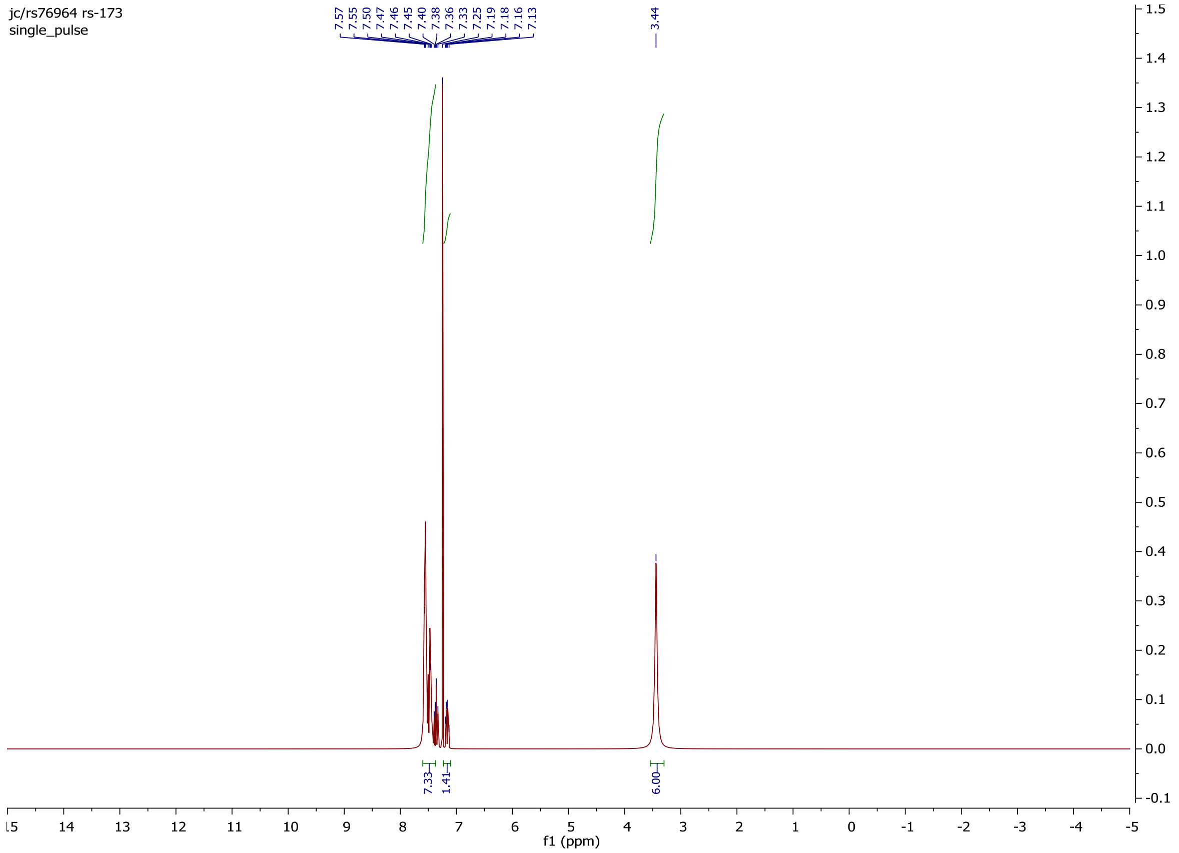

^{13}C NMR

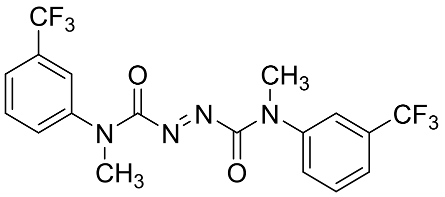

(*E*)-N[1],N[2]-dimethyl-N[1],N[2]-bis(3-(trifluoromethyl)phenyl)diazene-1,2-dicarboxamide (1f).

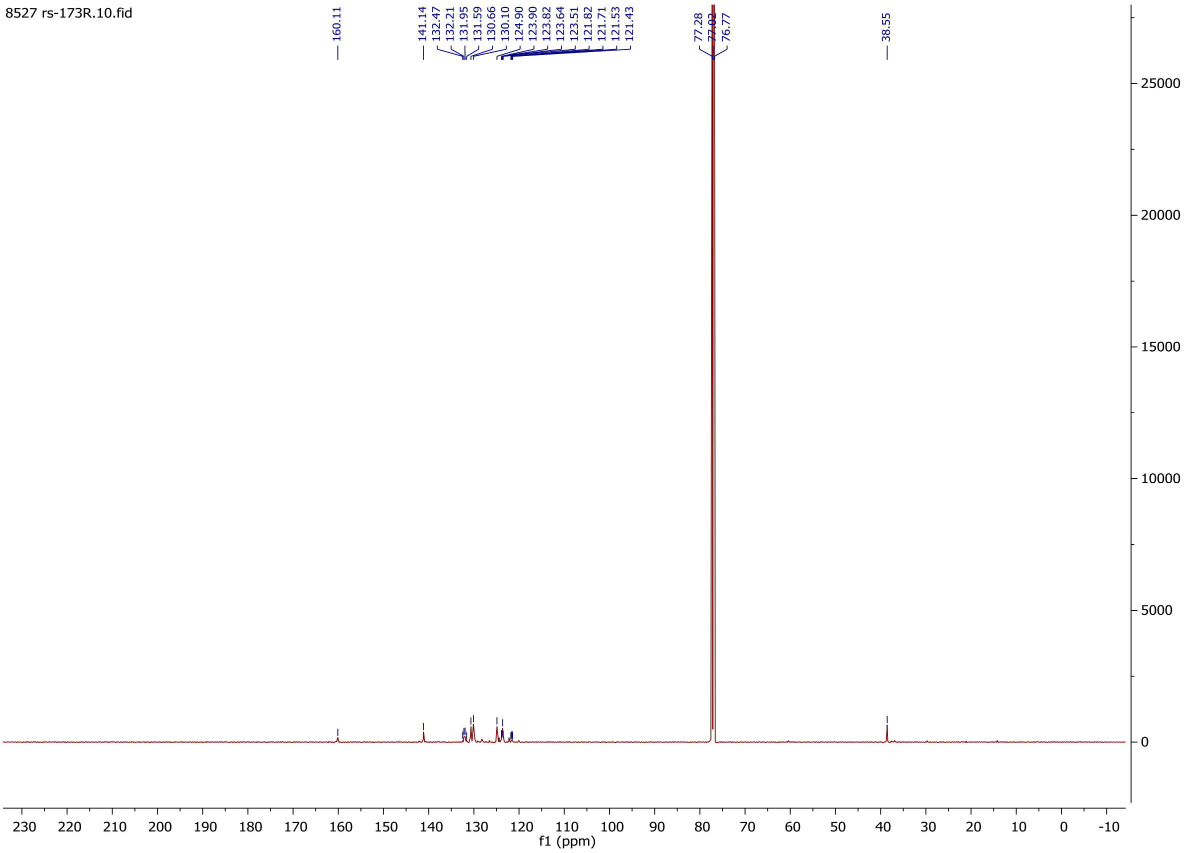

^{1}H NMR

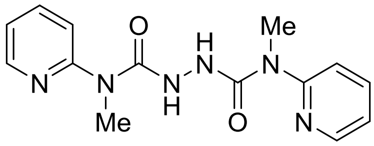

N[1],N[2]-dimethyl-N[1],N[2]-di(pyridin-2-yl)hydrazine-1,2-dicarboxamide (S1g)

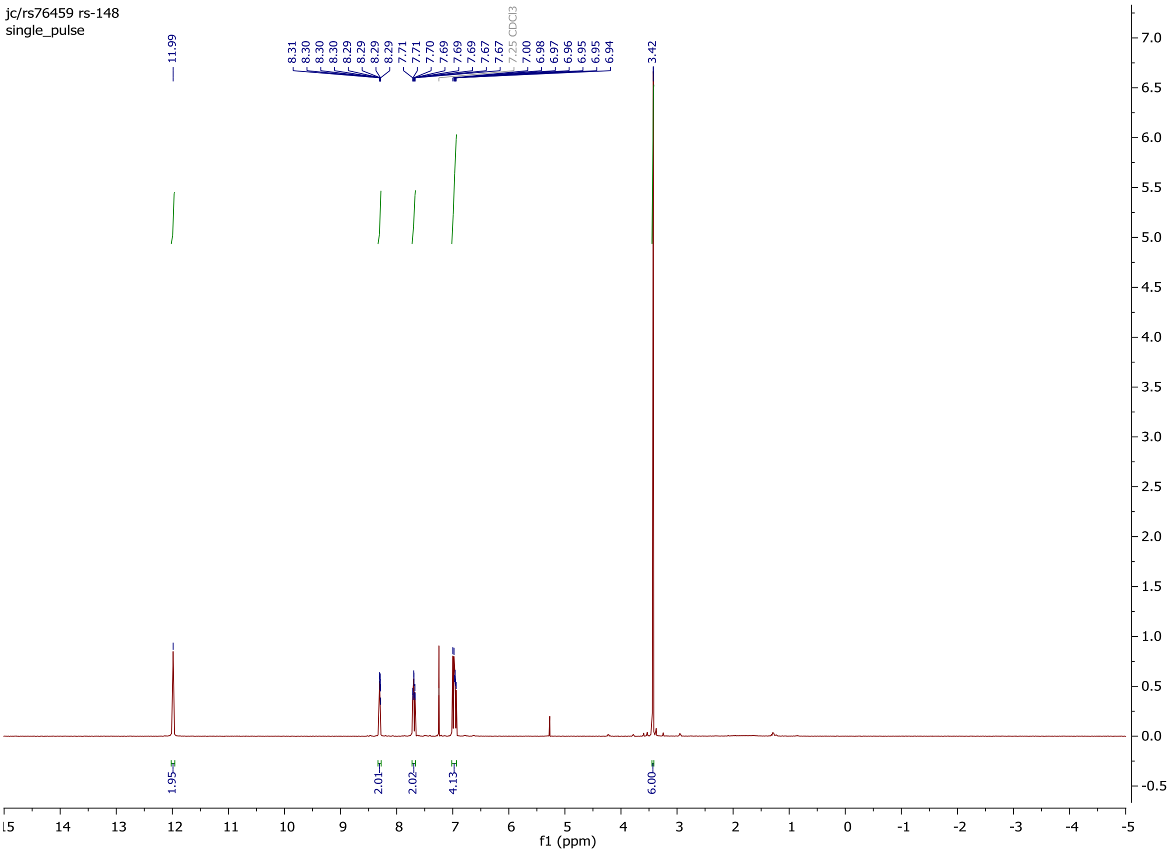

^{13}C NMR

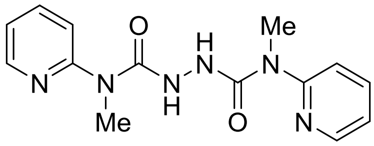

N[1],N[2]-dimethyl-N[1],N[2]-di(pyridin-2-yl)hydrazine-1,2-dicarboxamide (S1g)

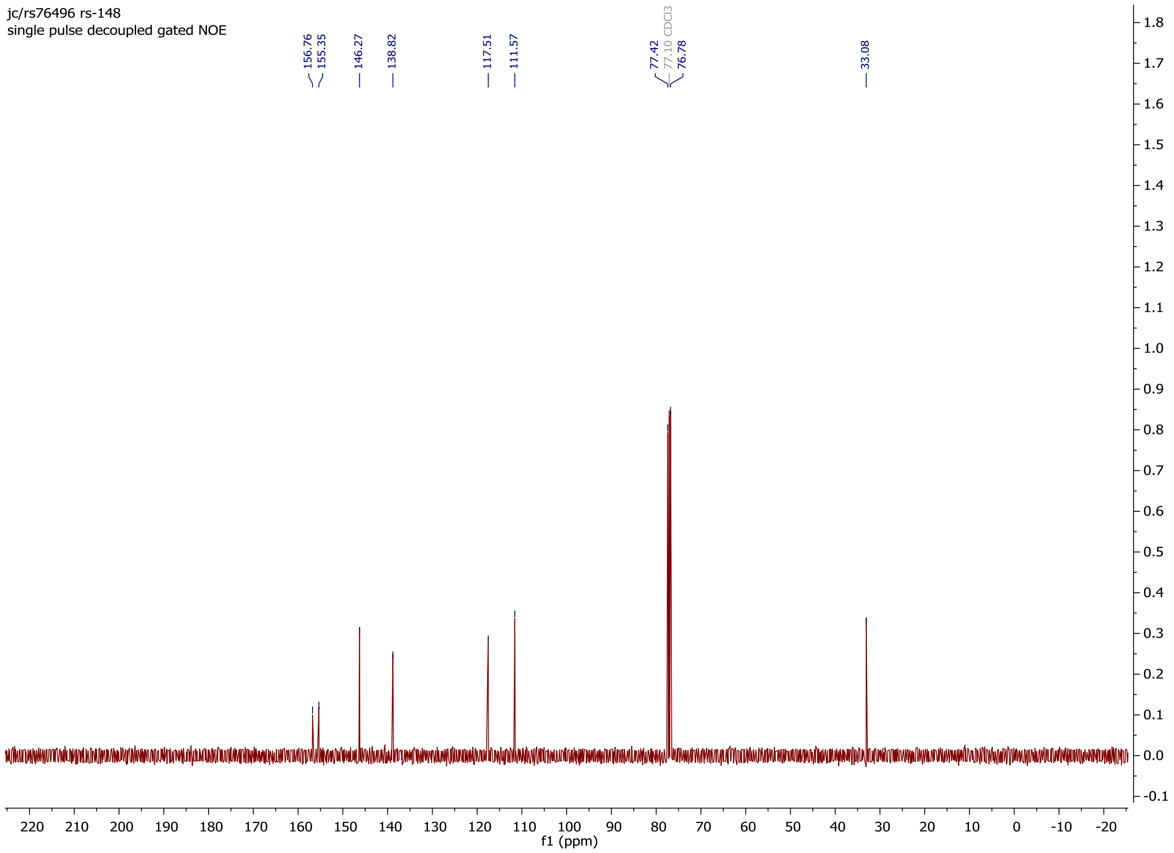

^{1}H NMR

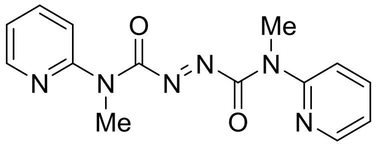

(*E*)-N^{1},N^{2}-dimethyl-N^{1},N^{2}-di(pyridin-2-yl)diazene-1,2-dicarboxamide (1g).

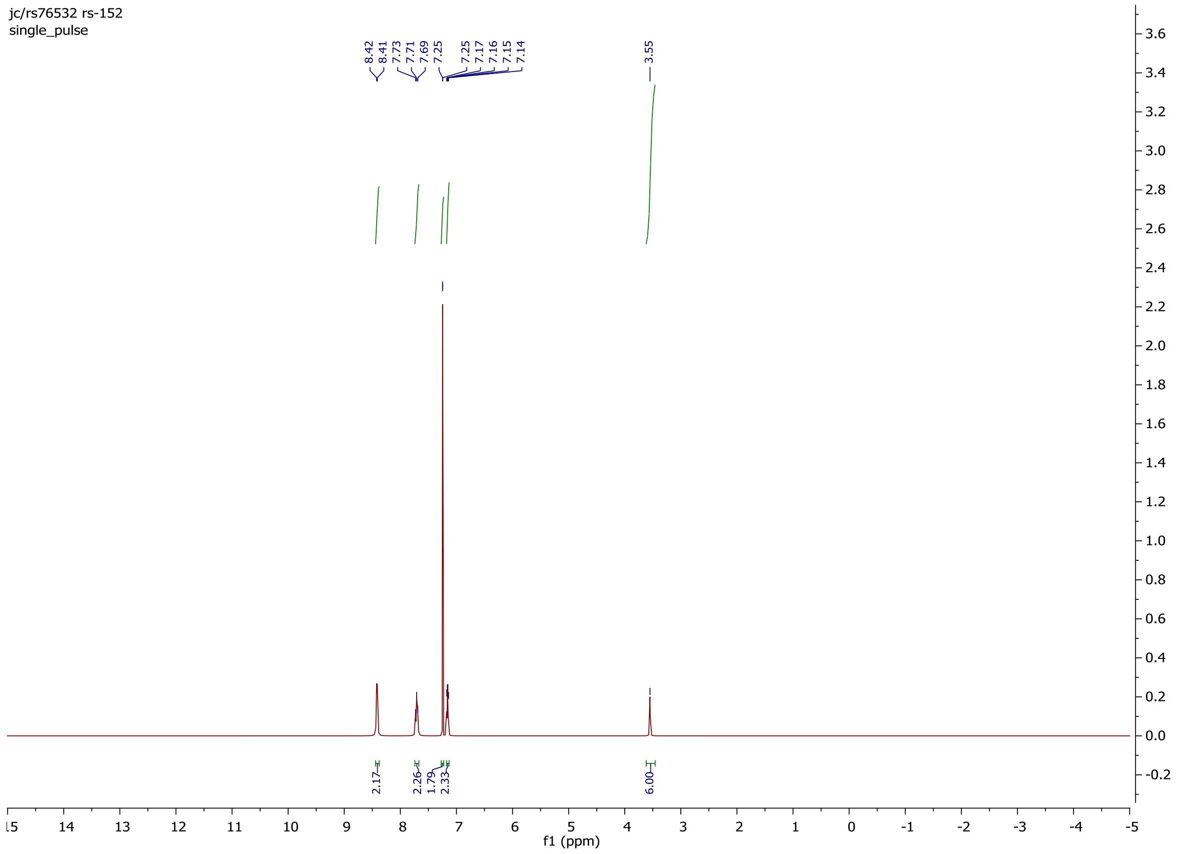

^{13}C NMR

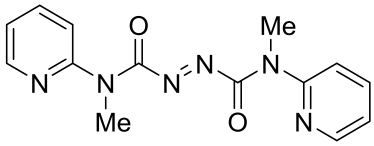

(*E*)-N[1],N[2]-dimethyl-N[1],N[2]-di(pyridin-2-yl)diazene-1,2-dicarboxamide (1g).

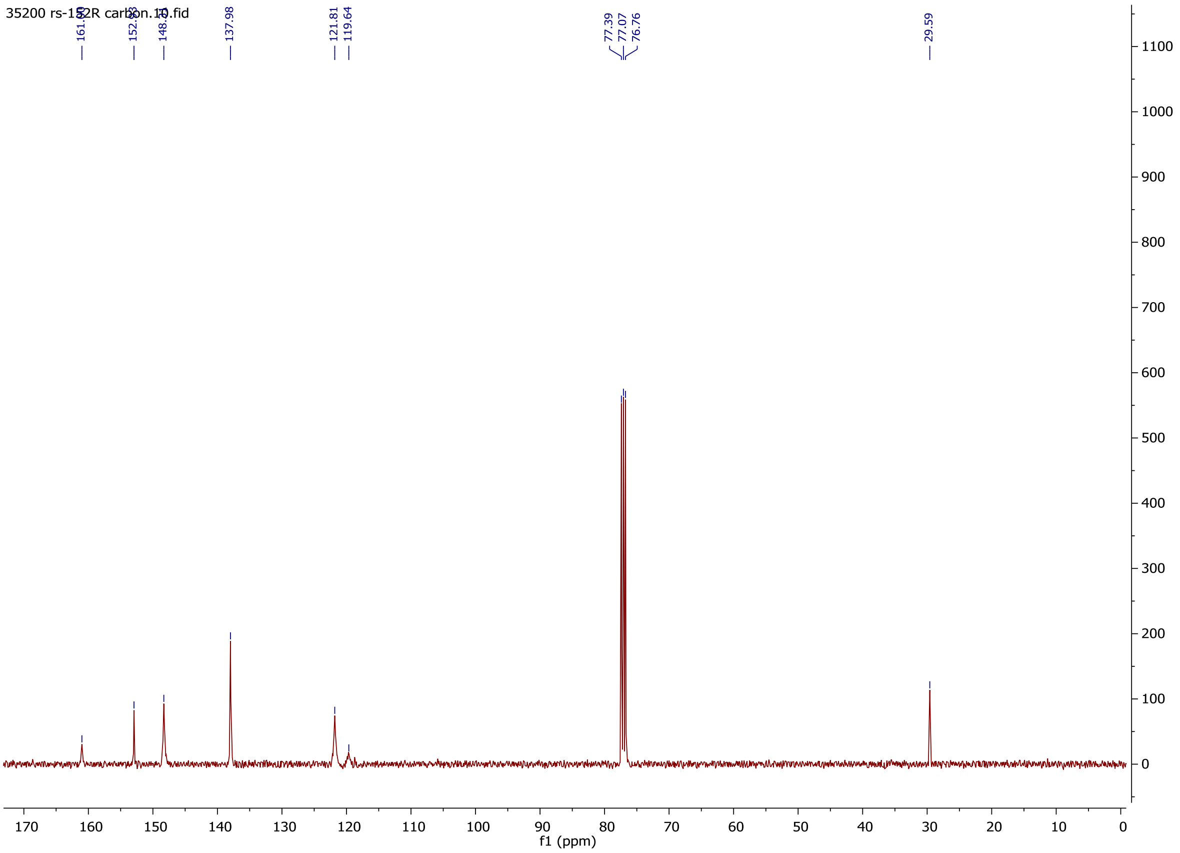

^{1}H NMR

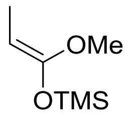

(1-Methoxyprop-1-en-1-yl)oxy)trimethylsilane (2a)

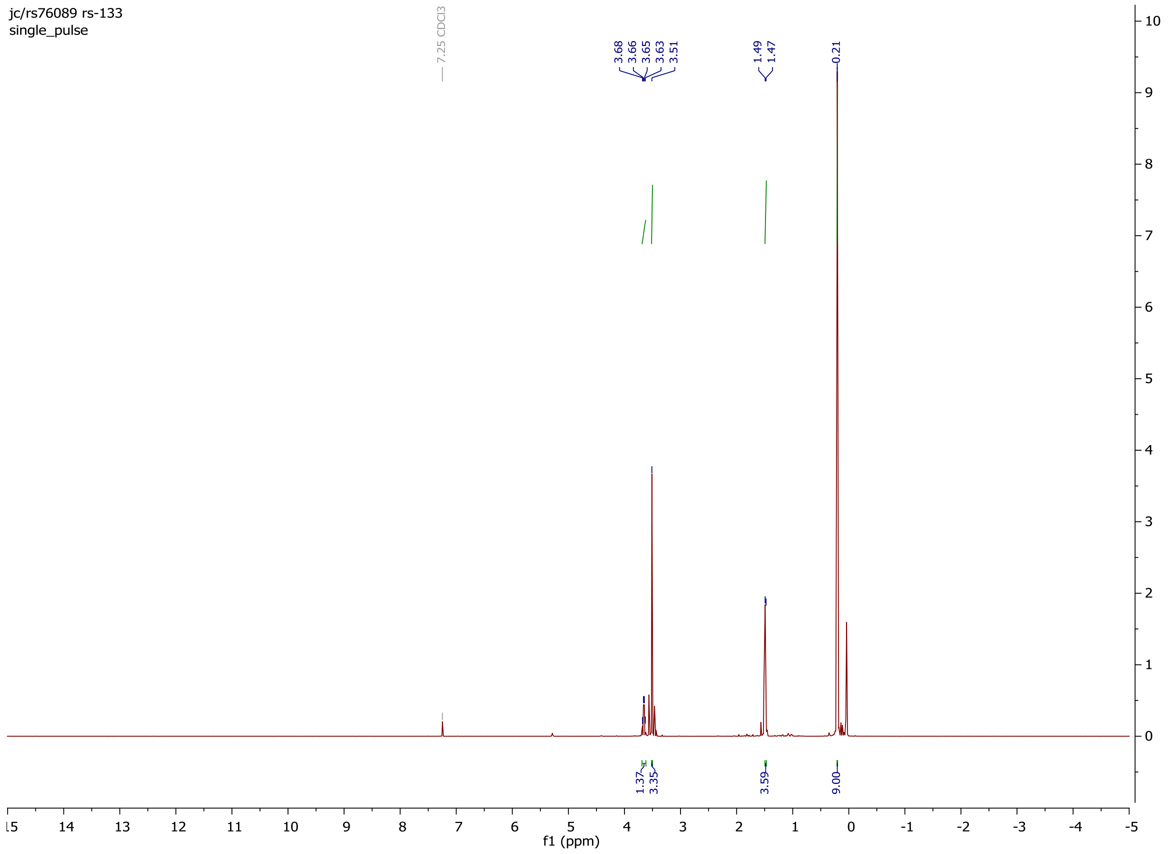

^{13}C NMR

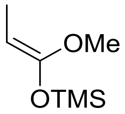

(1-Methoxyprop-1-en-1-yl)oxy)trimethylsilane (2a)

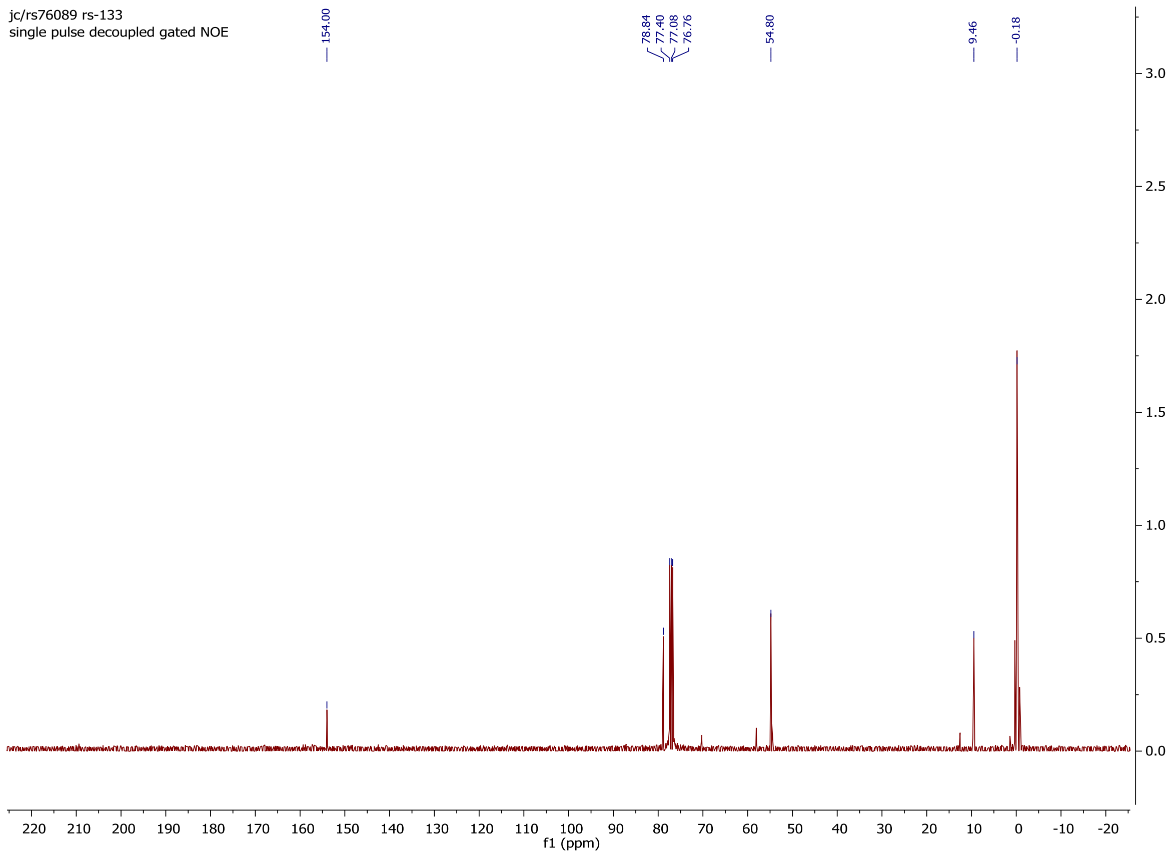

1H NMR

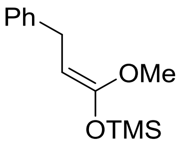

(1-Methoxy-3-phenylprop-1-en-1-yl)oxy)trimethylsilane (2b)

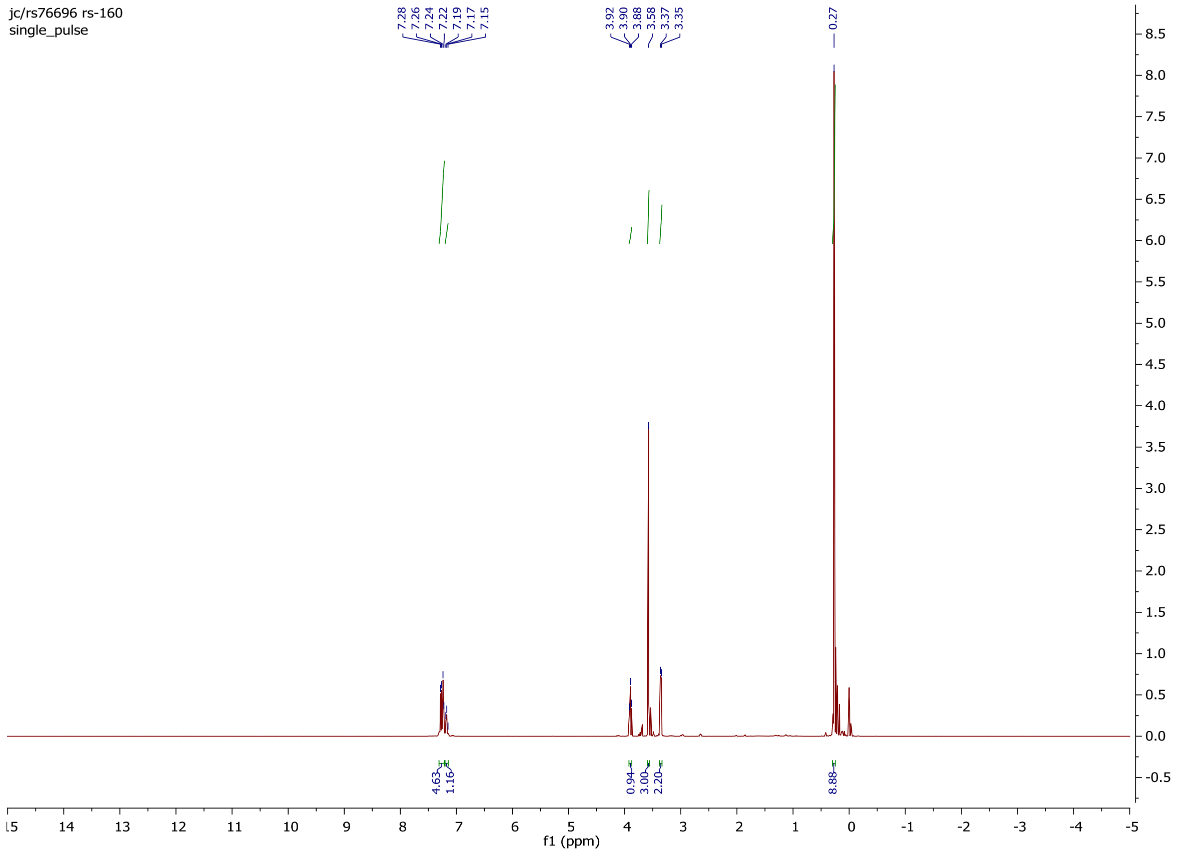

^{13}C NMR

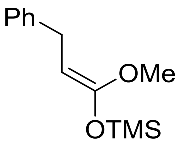

(1-Methoxy-3-phenylprop-1-en-1-yl)oxy)trimethylsilane (2b)

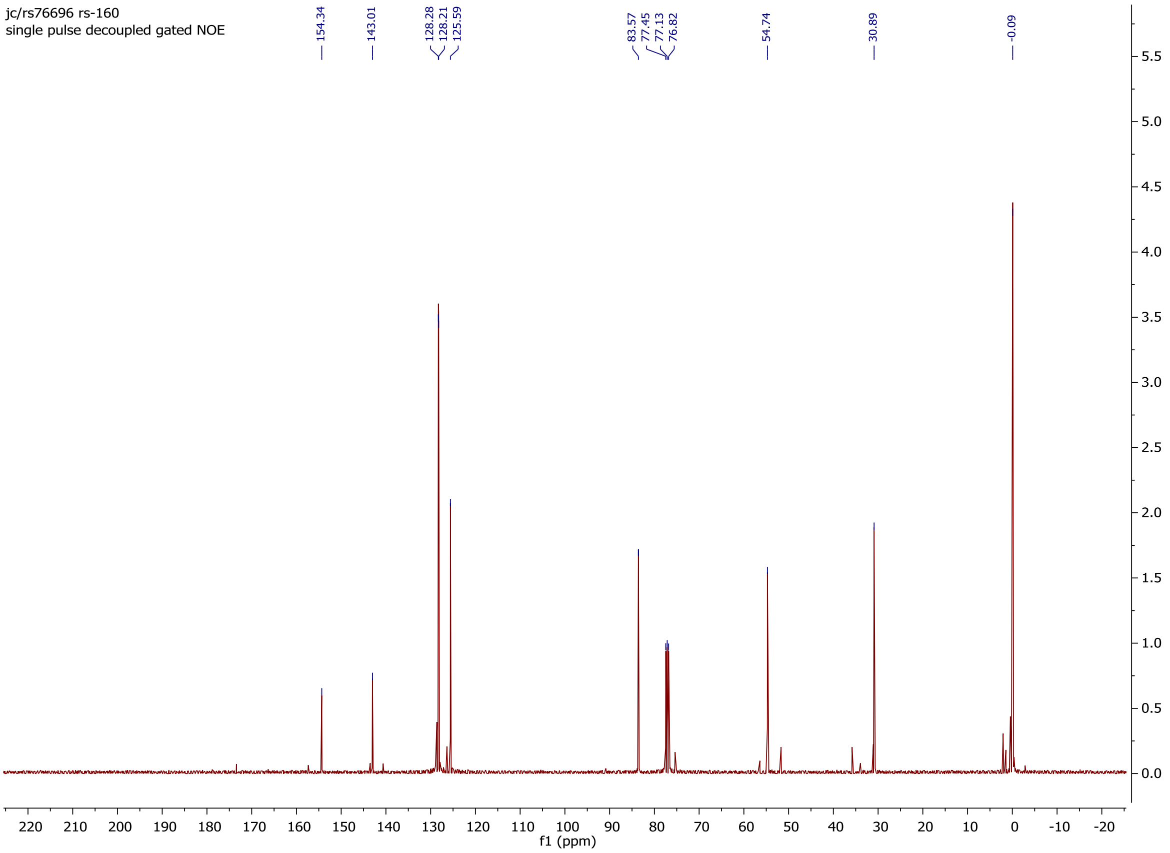

1H NMR

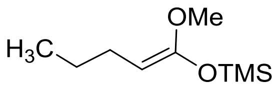

(1-Methoxypent-1-en-1-yl)oxy)trimethylsilane (2c)

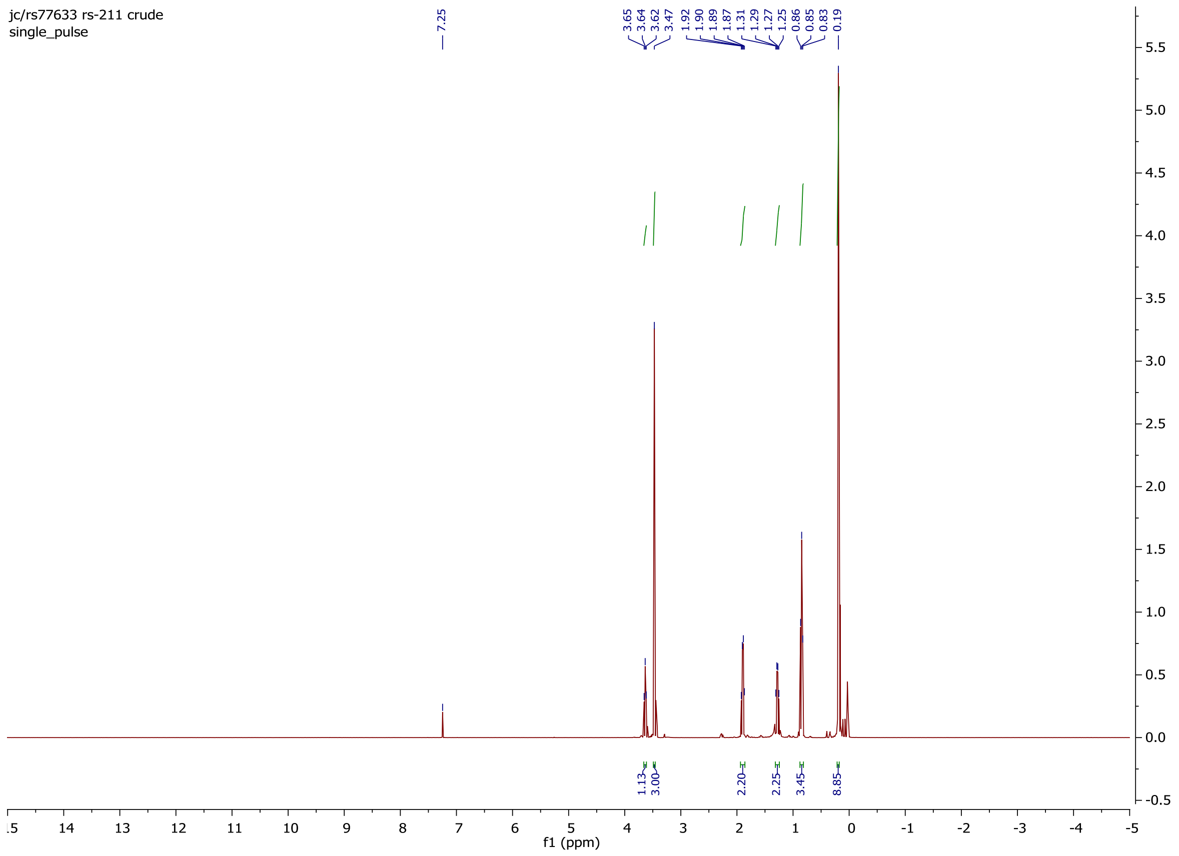

13C NMR

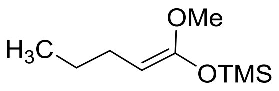

(*E*)-((1-methoxypent-1-en-1-yl)oxy)trimethylsilane (2c)

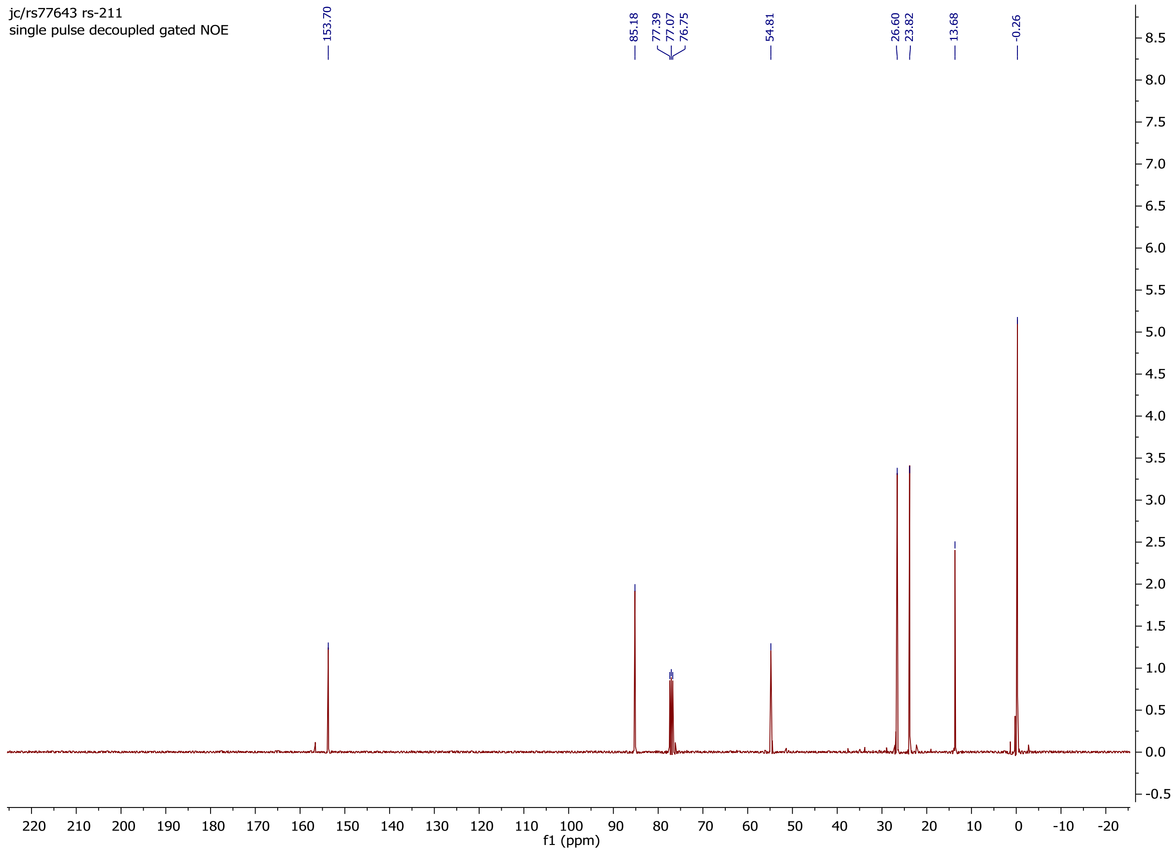

^{1}H NMR

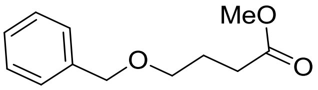

Methyl 4-(benzyloxy)butanoate S2d

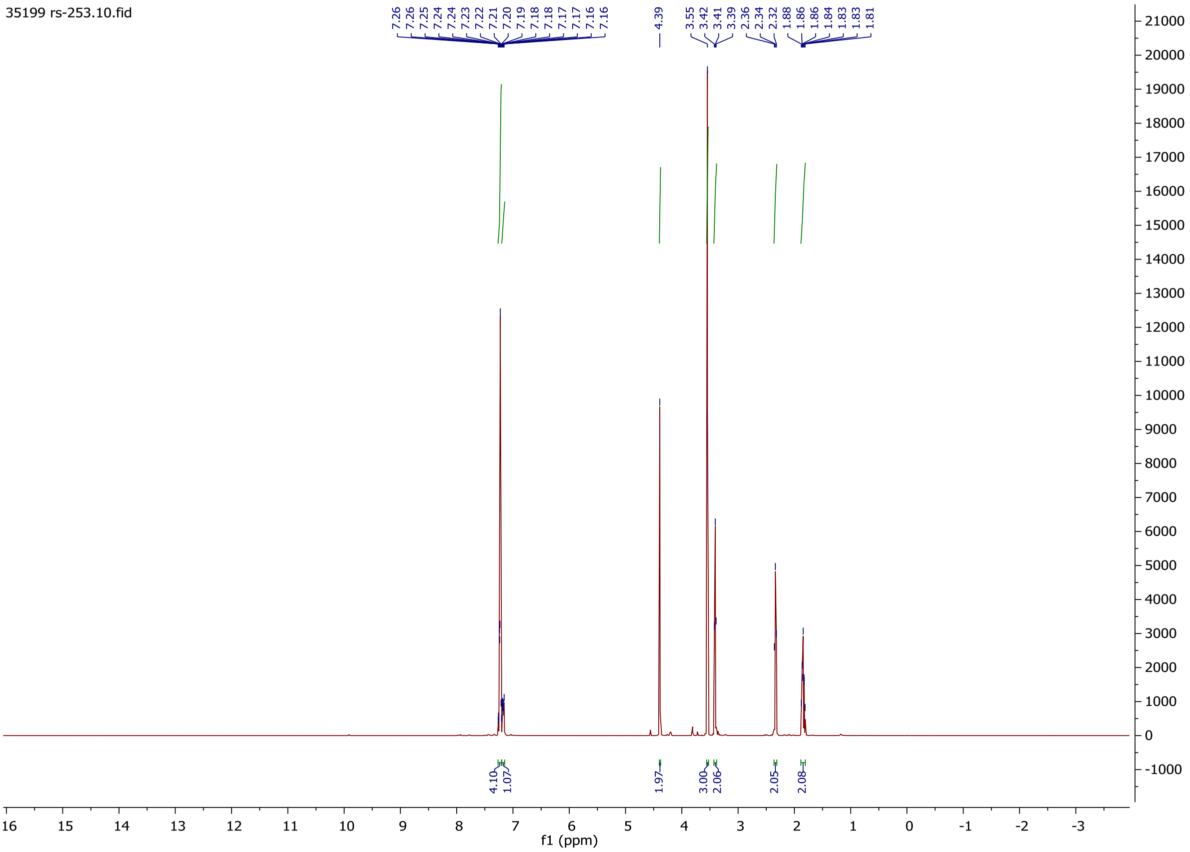

^{13}C NMR

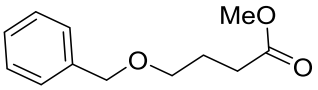

Methyl 4-(benzyloxy)butanoate (S2d)

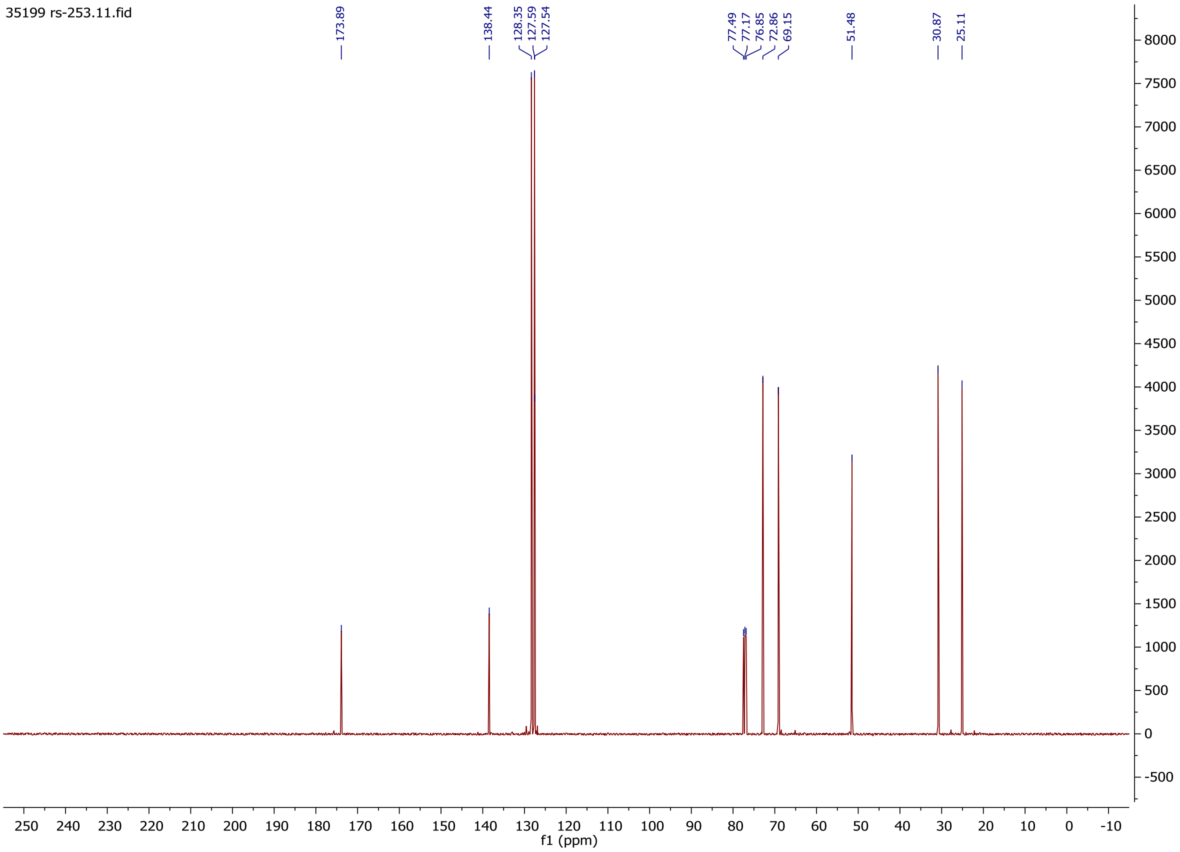

^{1}H NMR

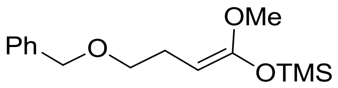

((4-(Benzyloxy)-1-methoxybut-1-en-1-yl)oxy)trimethylsilane (2d)

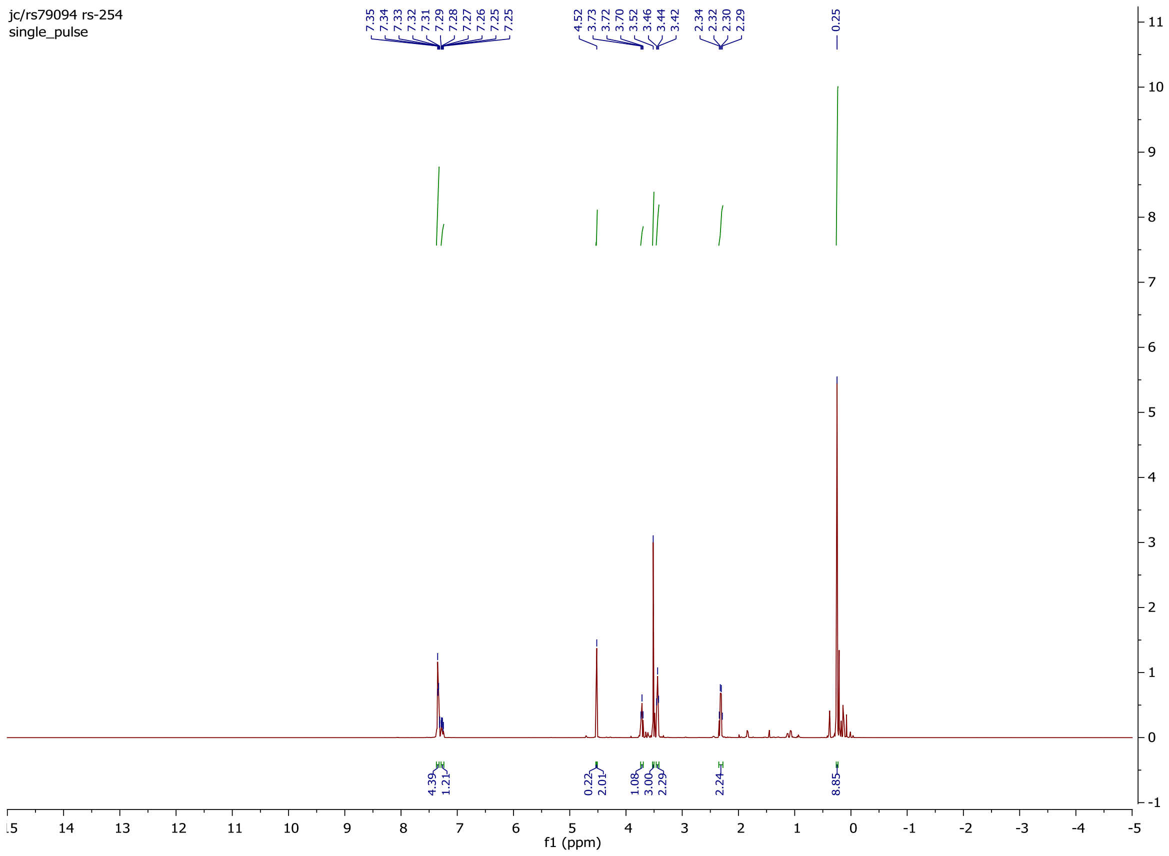

^{13}C NMR

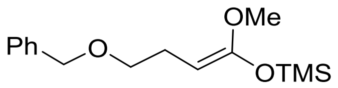

((4-(Benzyloxy)-1-methoxybut-1-en-1-yl)oxy)trimethylsilane (2d)

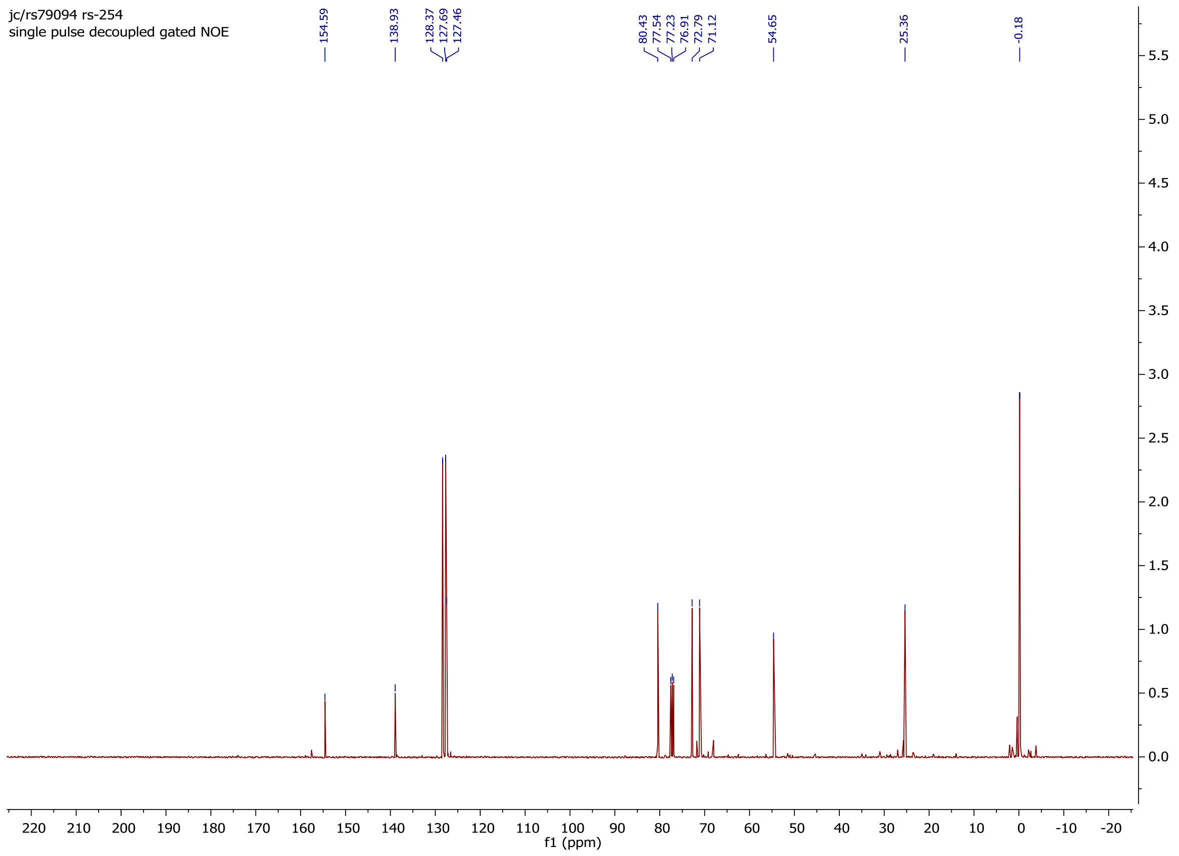

^{1}H NMR

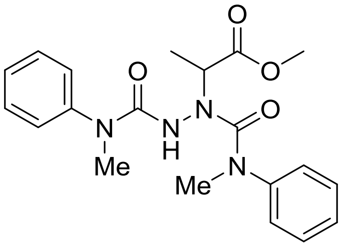

methyl N-(methyl(phenyl)carbamoyl)-N-(3-methyl-3-phenylureido)alaninate (3)

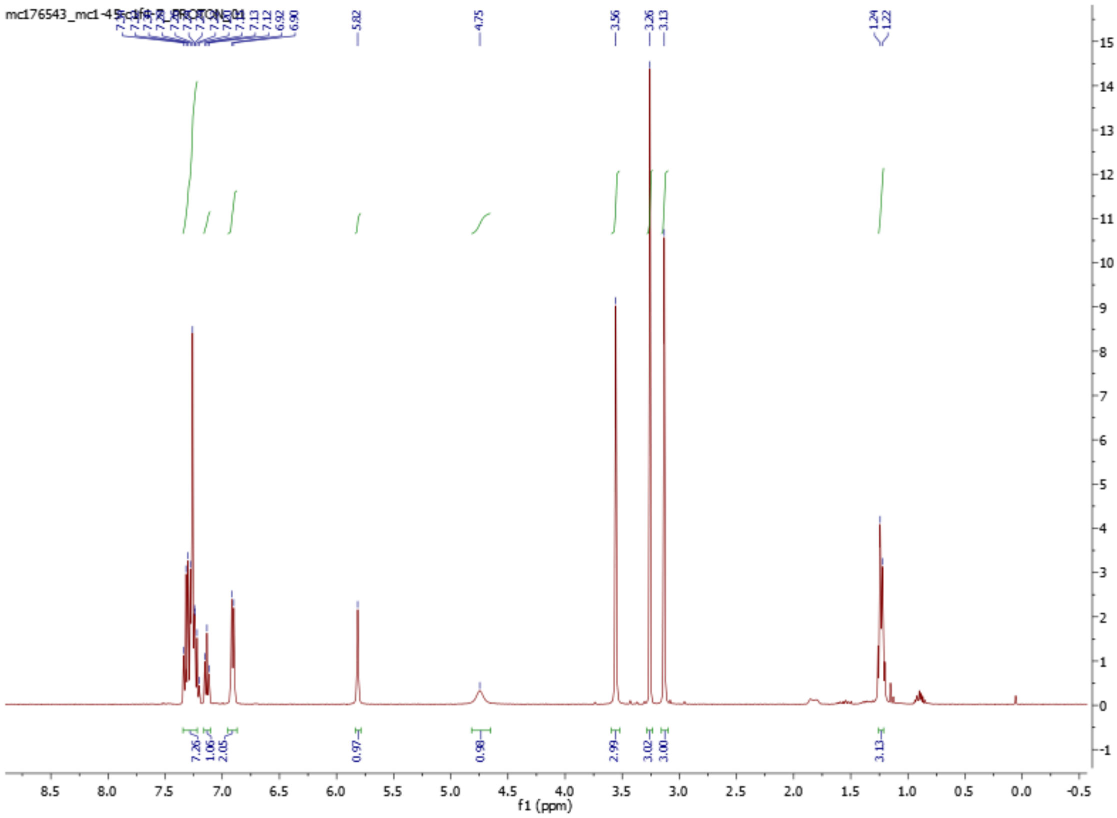

^{13}C NMR

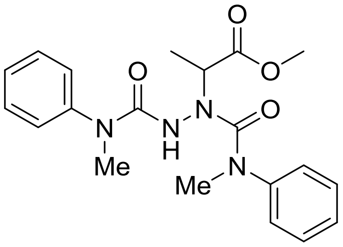

methyl N-(methyl(phenyl)carbamoyl)-N-(3-methyl-3-phenylureido)alaninate (3)

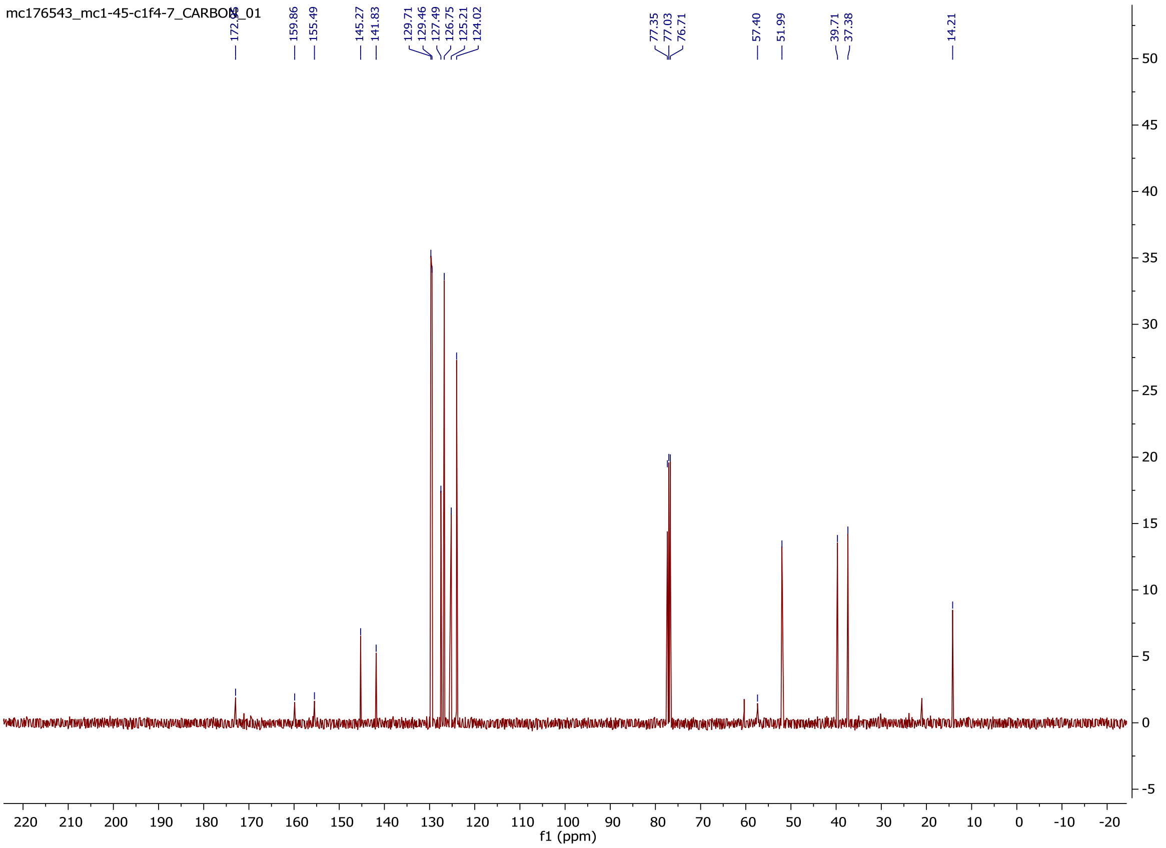

^{1}H NMR

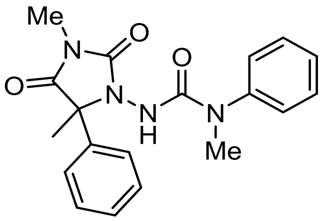

3-(3,5-dimethyl-2,4-dioxo-5-phenylimidazolidin-1-yl)-1-methyl-1-phenylurea (4a)

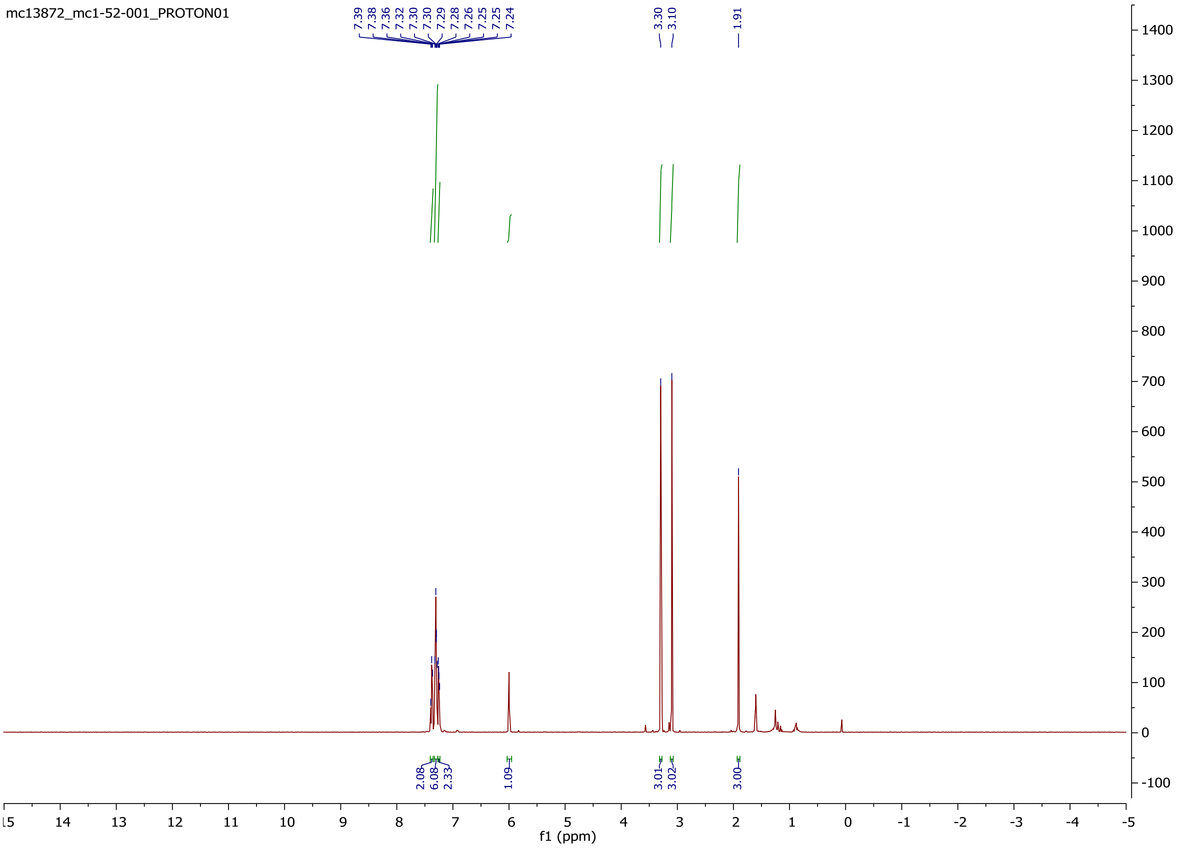

^{13}C NMR

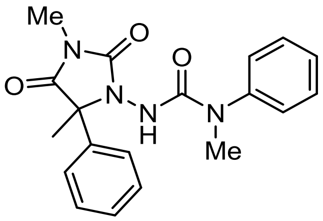

3-(3,5-dimethyl-2,4-dioxo-5-phenylimidazolidin-1-yl)-1-methyl-1-phenylurea (4a)

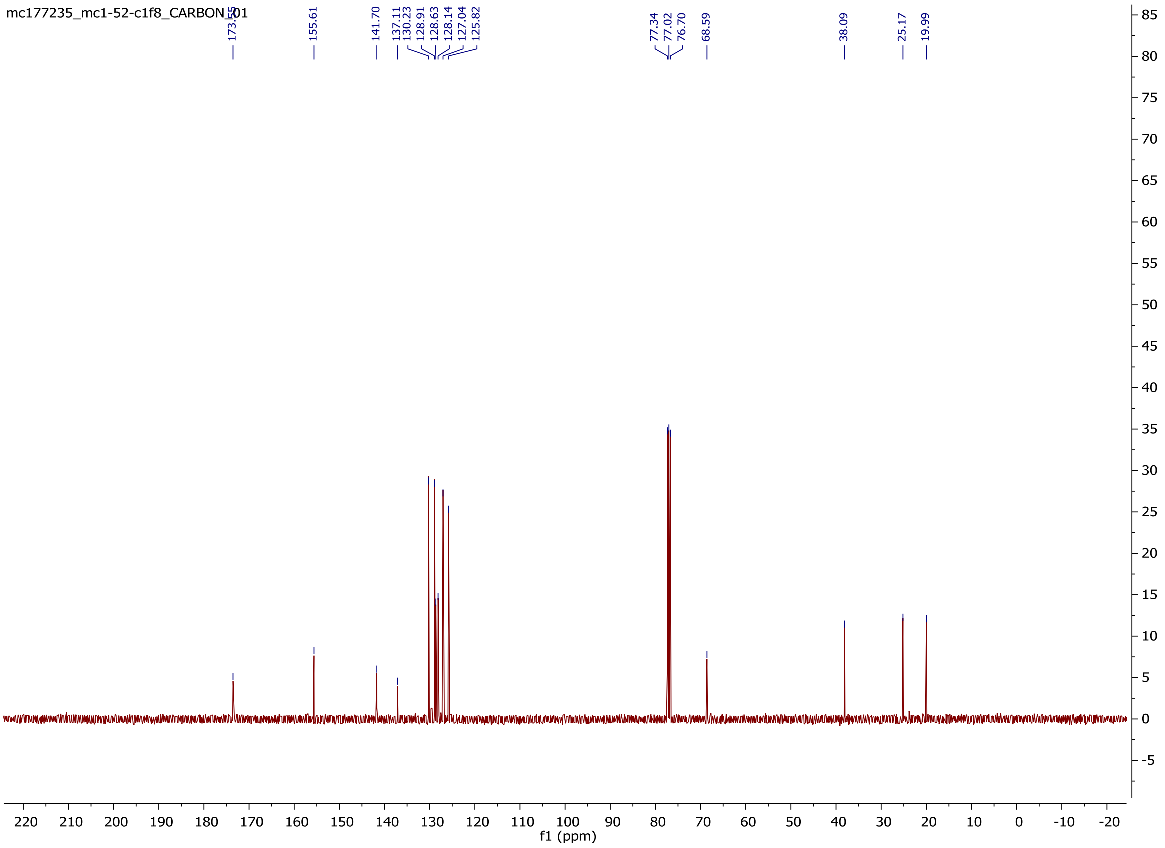

COSY

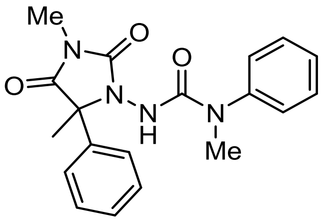

3-(3,5-dimethyl-2,4-dioxo-5-phenylimidazolidin-1-yl)-1-methyl-1-phenylurea (4a)

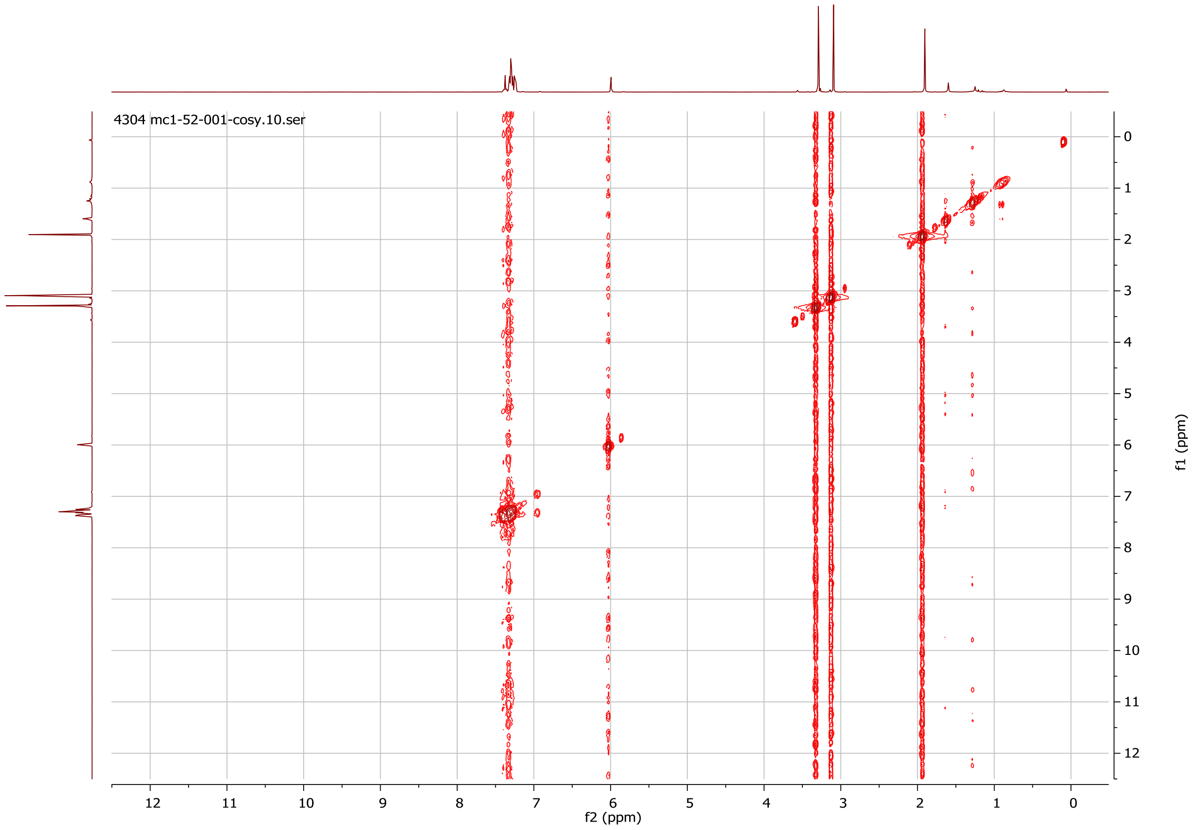

HSQC

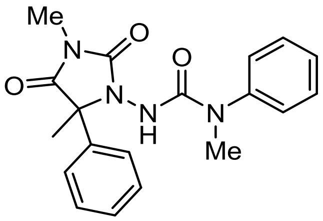

3-(3,5-dimethyl-2,4-dioxo-5-phenylimidazolidin-1-yl)-1-methyl-1-phenylurea (4a)

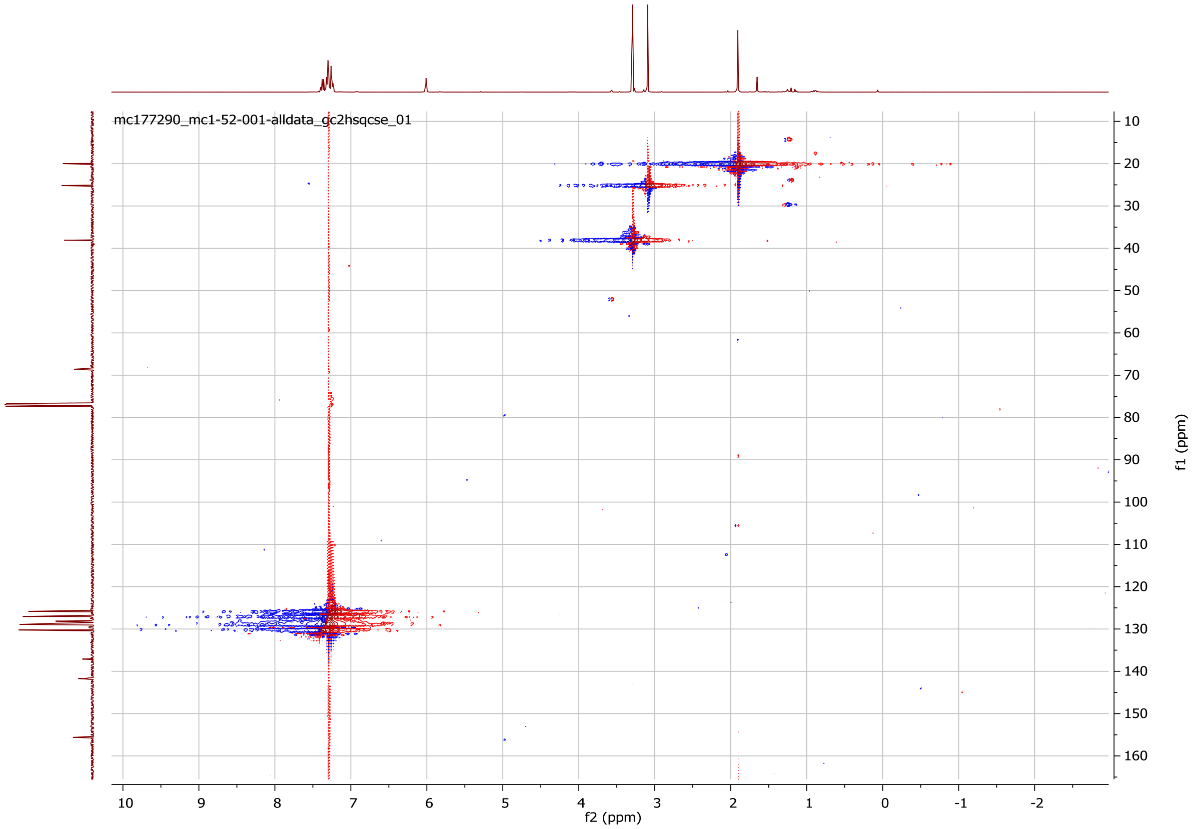

HMBC

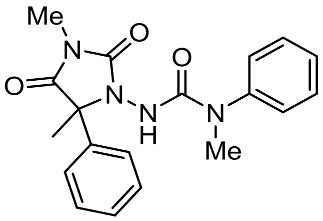

3-(3,5-dimethyl-2,4-dioxo-5-phenylimidazolidin-1-yl)-1-methyl-1-phenylurea (4a)

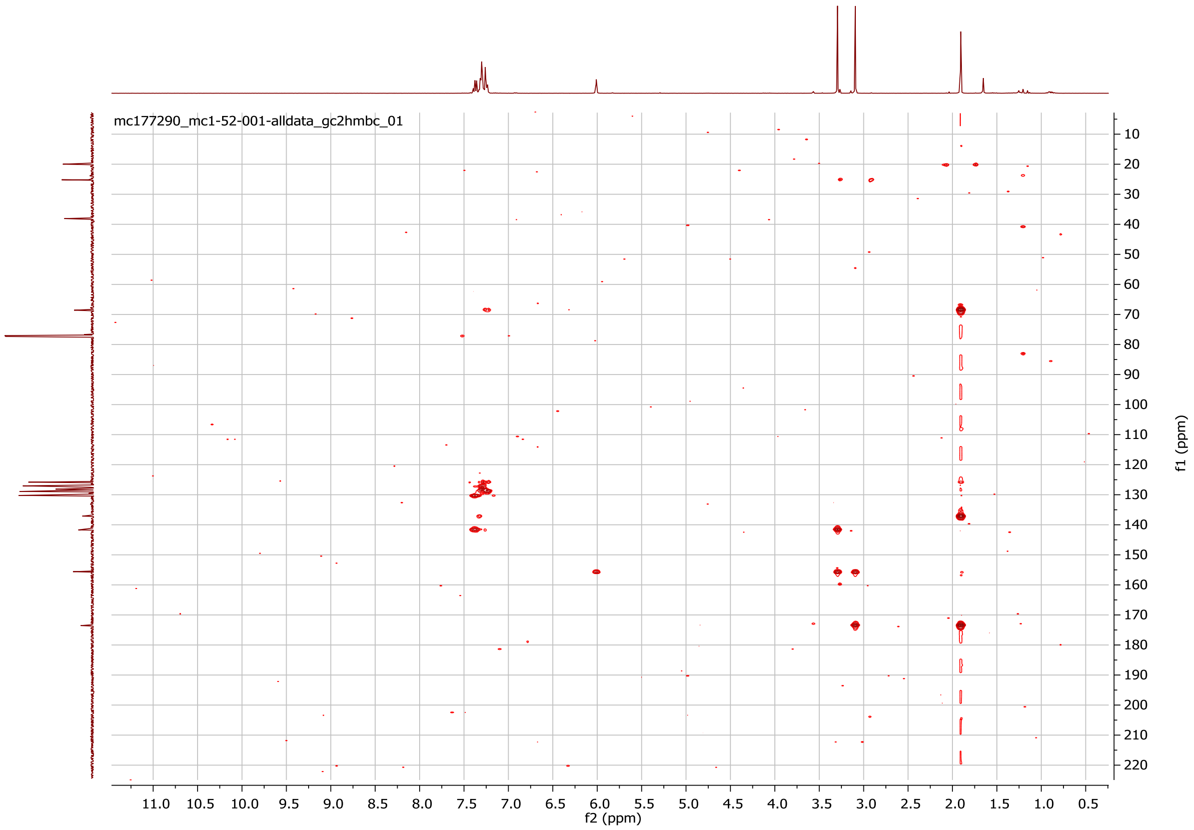

^{1}H NMR

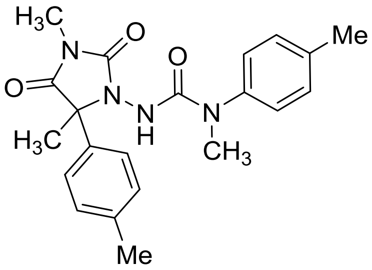

3-(3,5-Dimethyl-2,4-dioxo-5-(p-tolyl)imidazolidin-1-yl)-1-methyl-1-(p-tolyl)urea (4b)

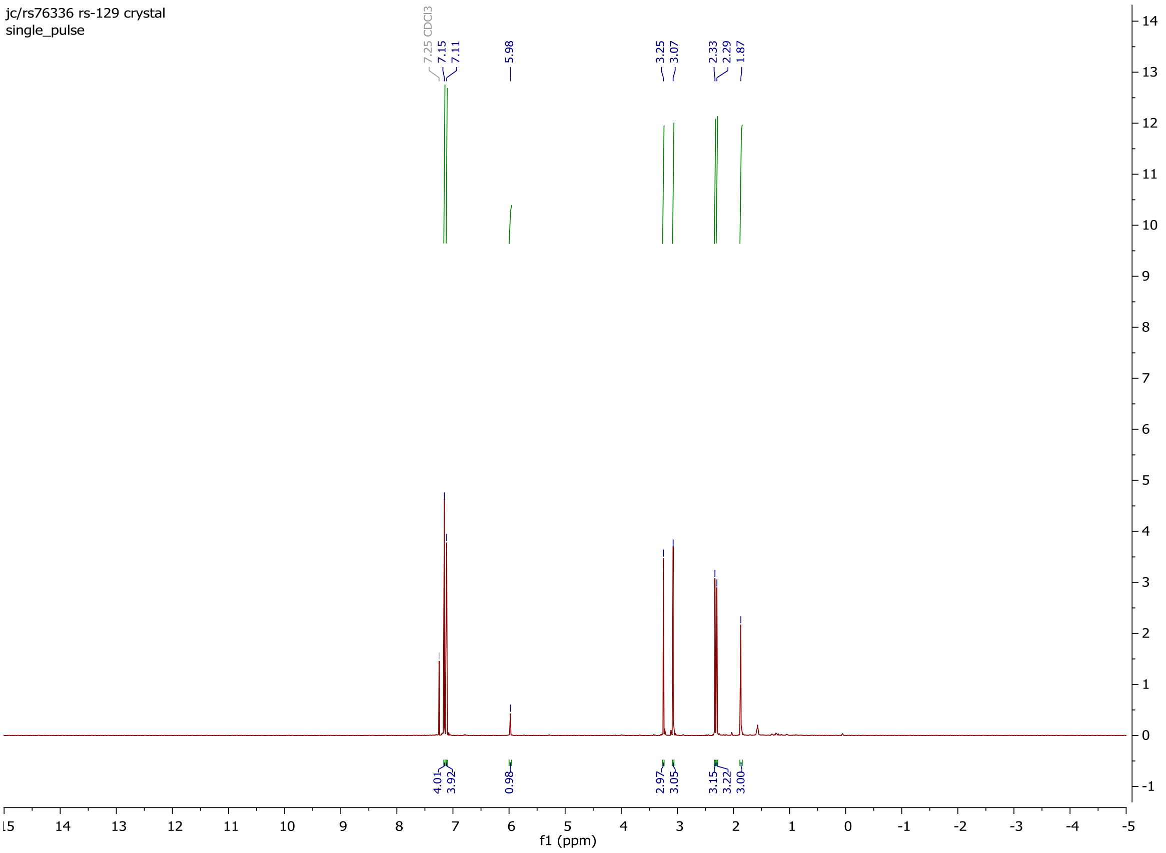

^{13}C NMR

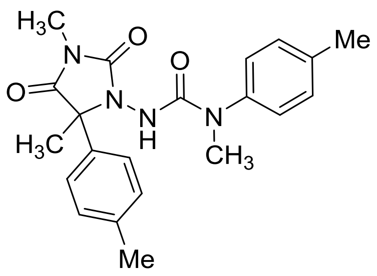

3-(3,5-Dimethyl-2,4-dioxo-5-(p-tolyl)imidazolidin-1-yl)-1-methyl-1-(p-tolyl)urea (4b)

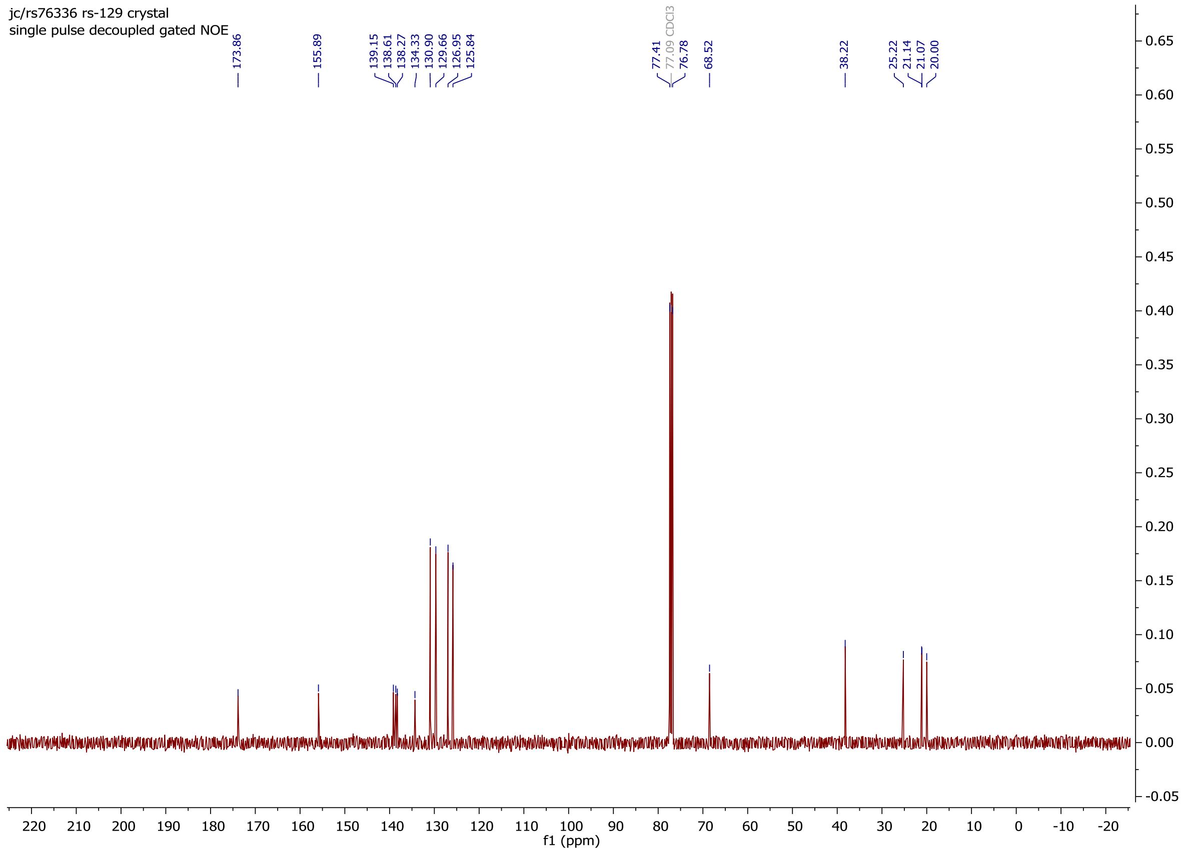

^{1}H NMR

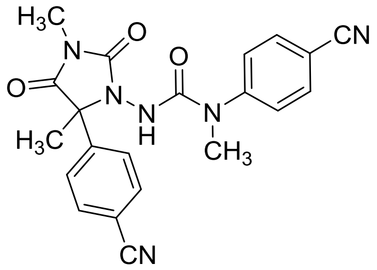

1-(4-Cyanophenyl)-3-(5-(4-cyanophenyl)-3,5-dimethyl-2,4-dioxoimidazolidin-1-yl)-1-methylurea (4c)

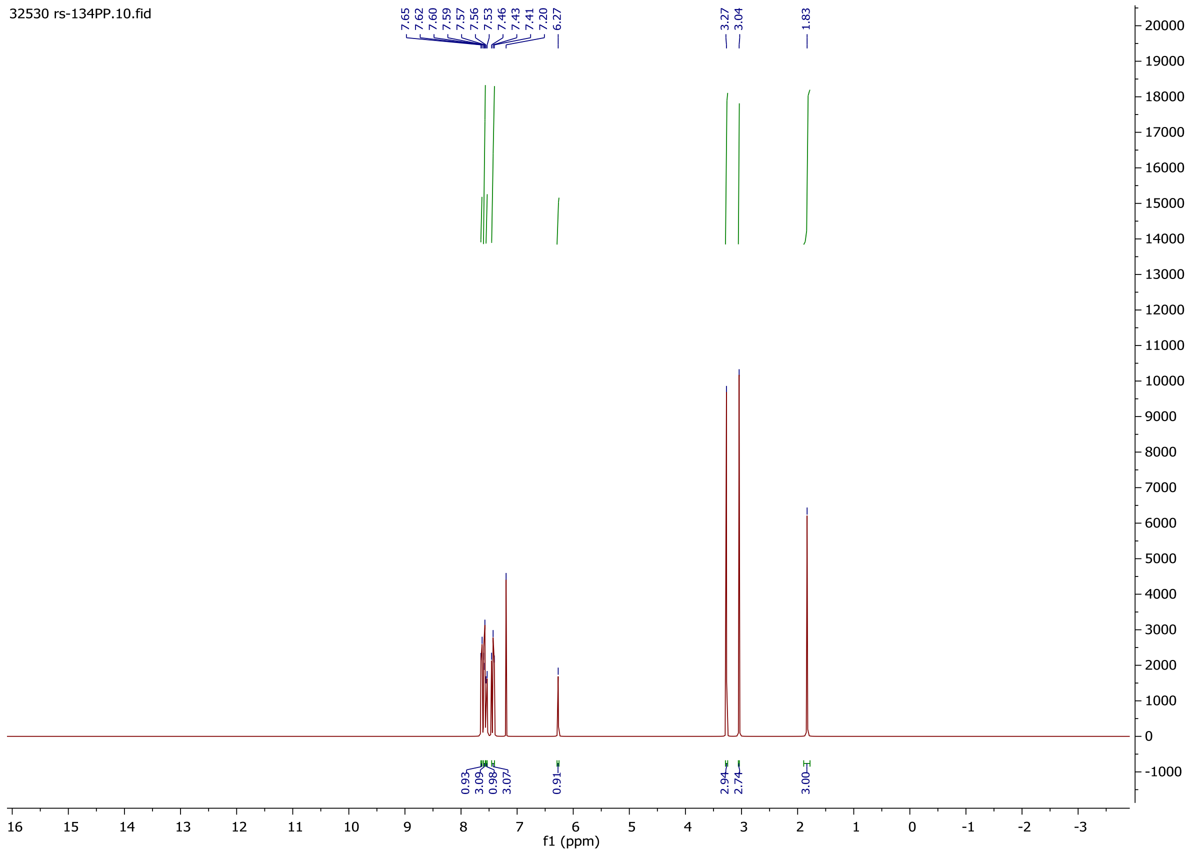

^{13}C NMR

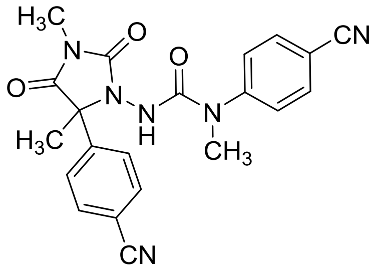

1-(4-Cyanophenyl)-3-(5-(4-cyanophenyl)-3,5-dimethyl-2,4-dioxoimidazolidin-1-yl)-1-methylurea (4c)

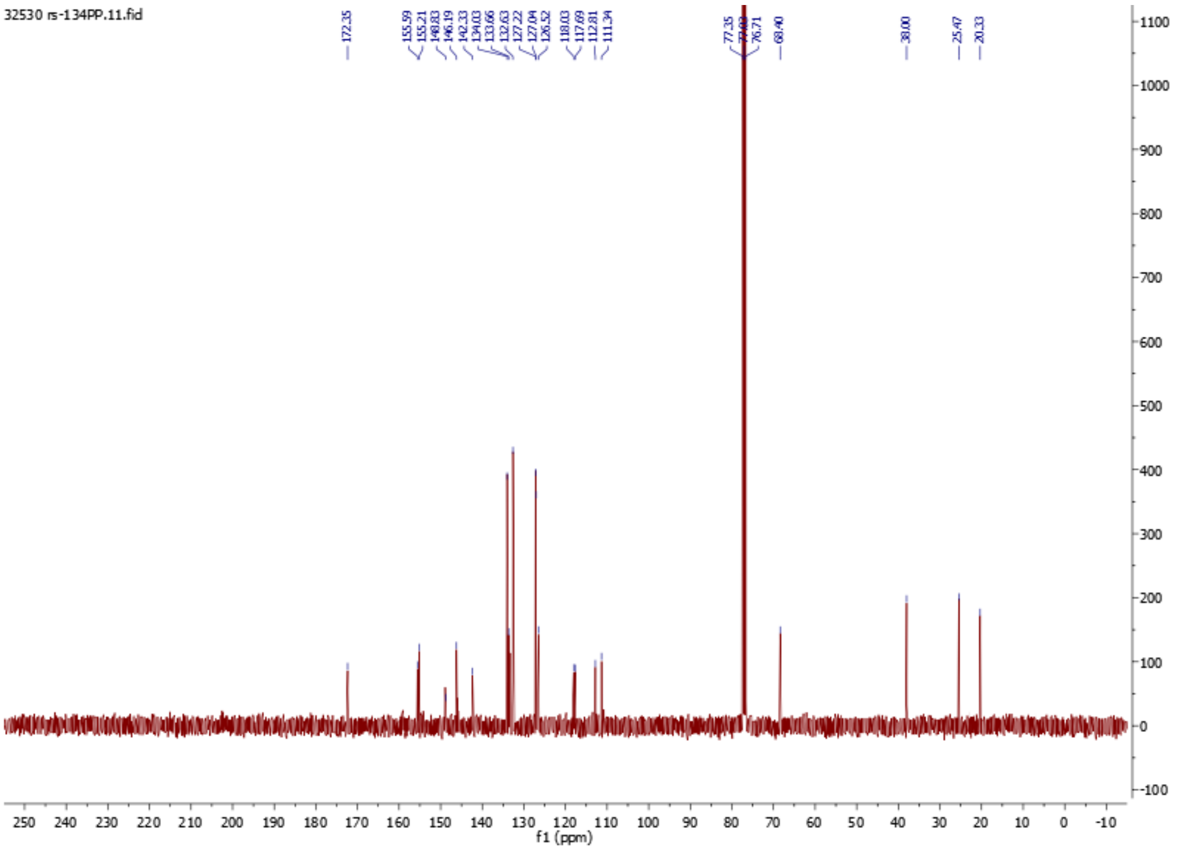

^{1}H NMR

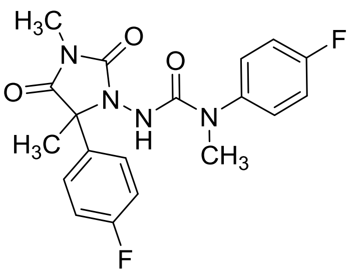

1-(4-Fluorophenyl)-3-(5-(4-fluorophenyl)-3,5-dimethyl-2,4-dioxoimidazolidin-1-yl)-1-methylurea (4d)

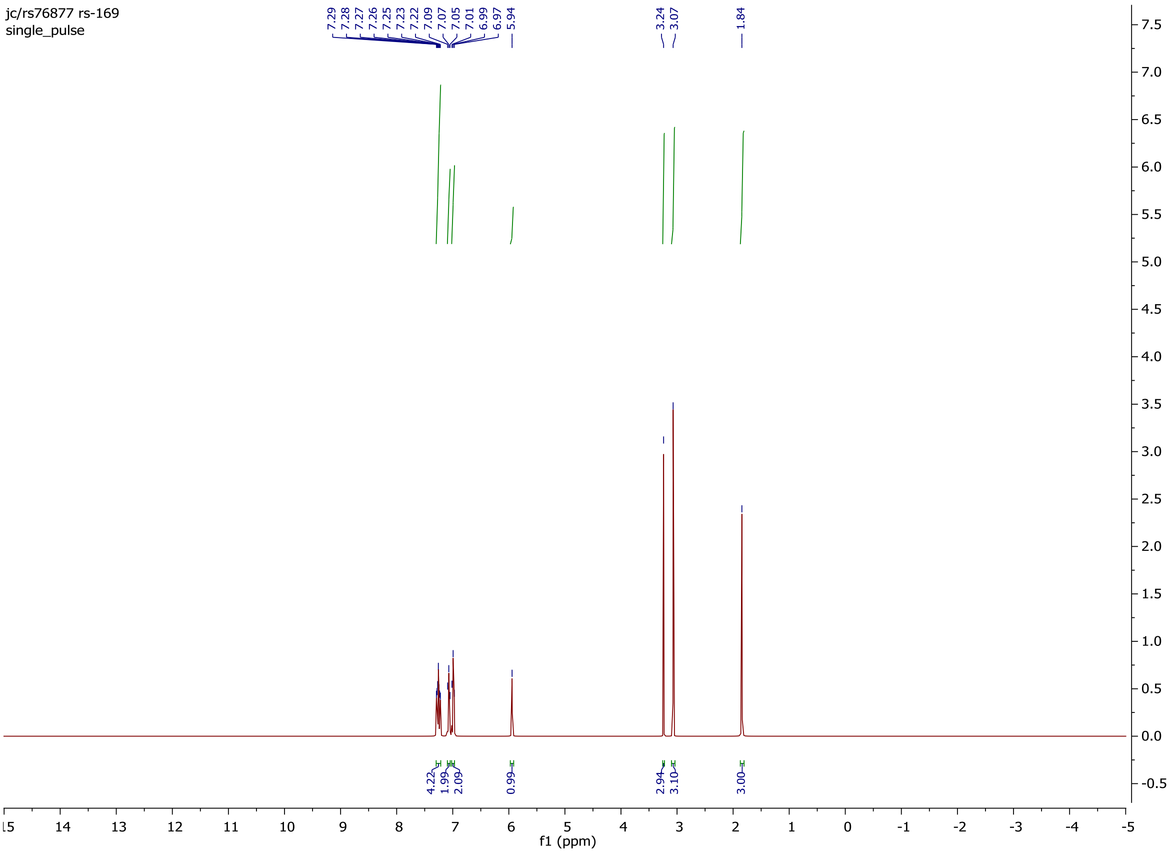

^{13}C NMR

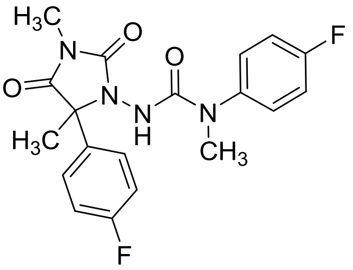

1-(4-Fluorophenyl)-3-(5-(4-fluorophenyl)-3,5-dimethyl-2,4-dioxoimidazolidin-1-yl)-1-methylurea (4d)

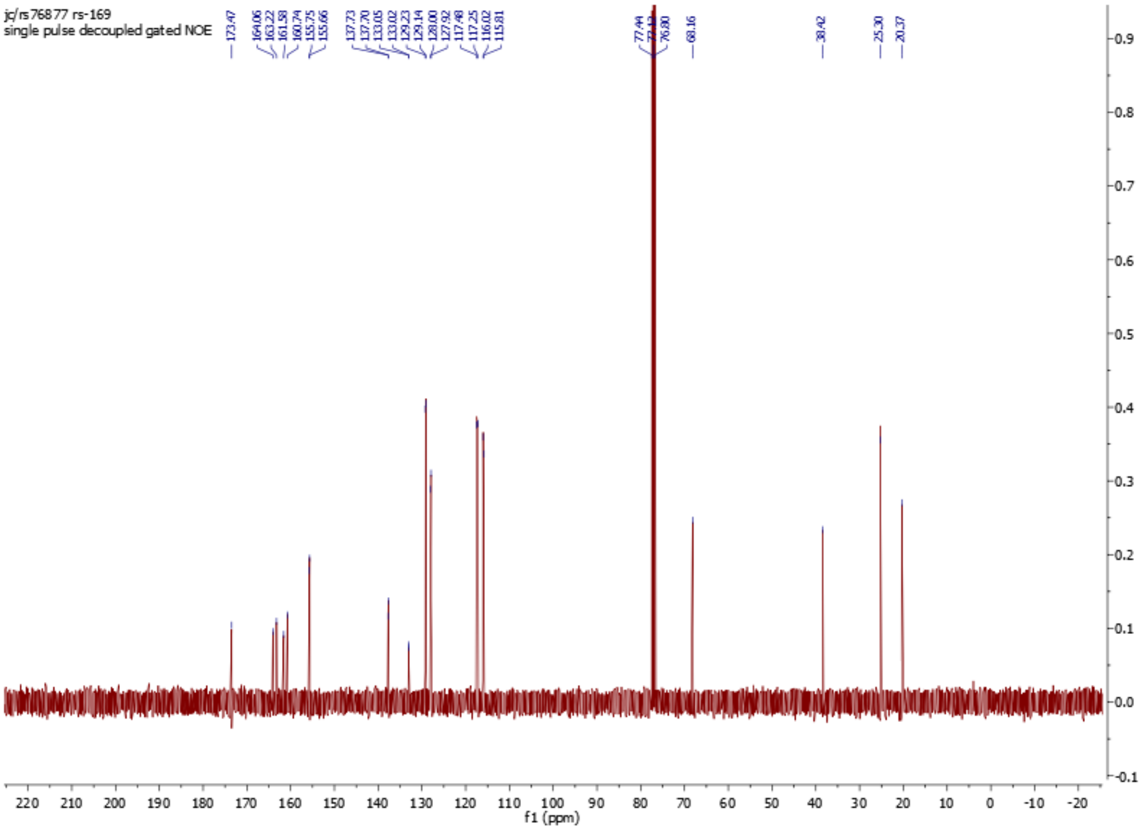

^{1}H NMR

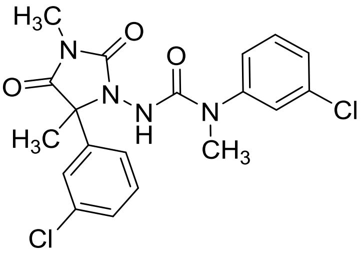

1-(3-Chlorophenyl)-3-(5-(3-chlorophenyl)-3,5-dimethyl-2,4-dioxoimidazolidin-1-yl)-1-methylurea (4e)

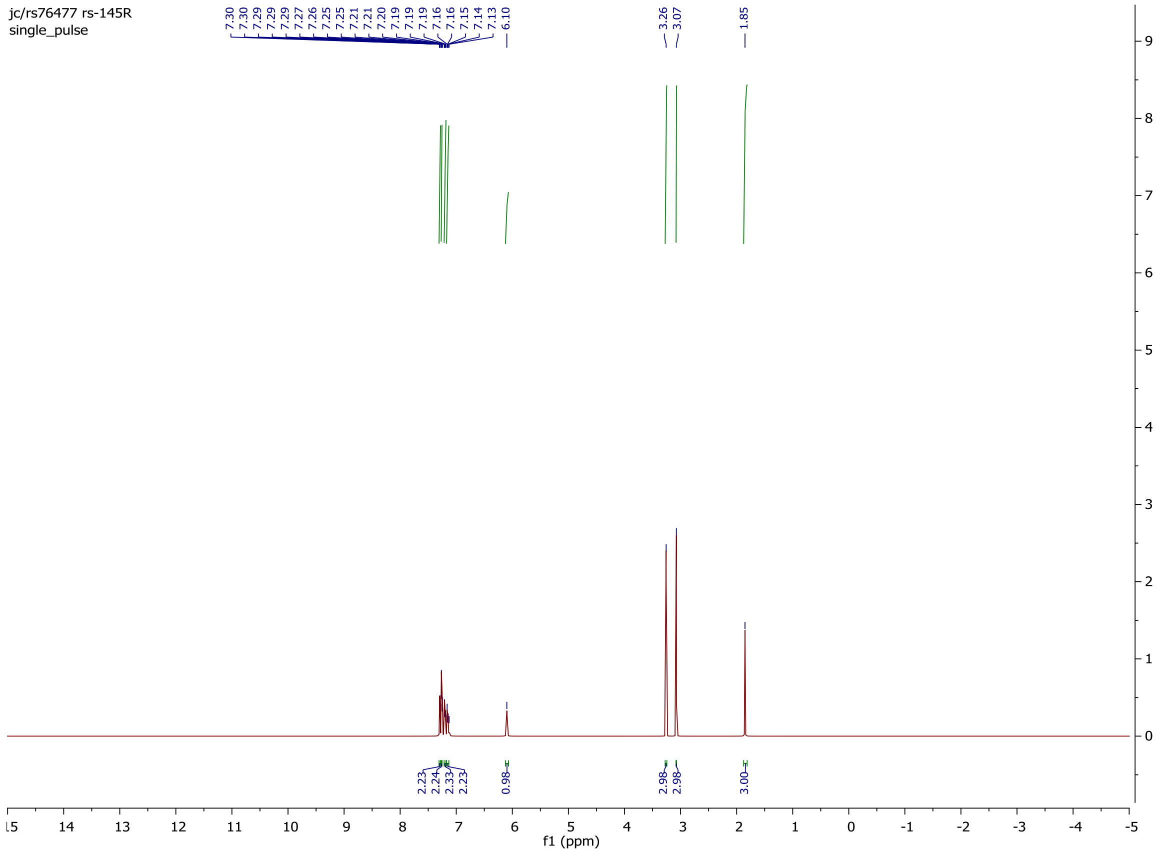

^{13}C NMR

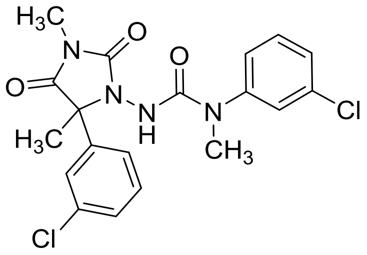

1-(3-Chlorophenyl)-3-(5-(3-chlorophenyl)-3,5-dimethyl-2,4-dioxoimidazolidin-1-yl)-1-methylurea (4e)

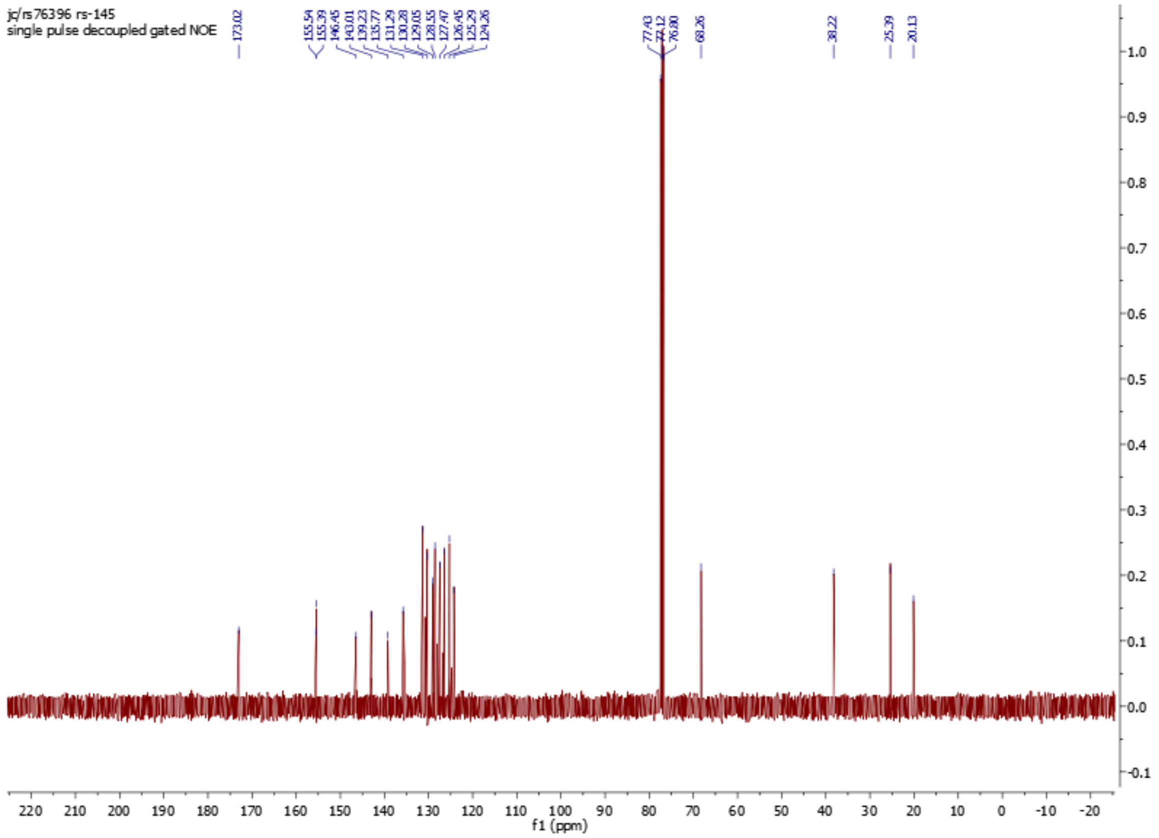

^{1}H NMR

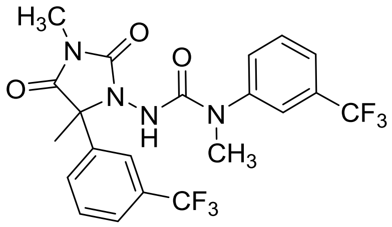

3-(3,5-Dimethyl-2,4-dioxo-5-(3-(trifluoromethyl)phenyl)imidazolidin-1-yl)-1-methyl-1-(3-(trifluoromethyl)phenyl)urea (4f)

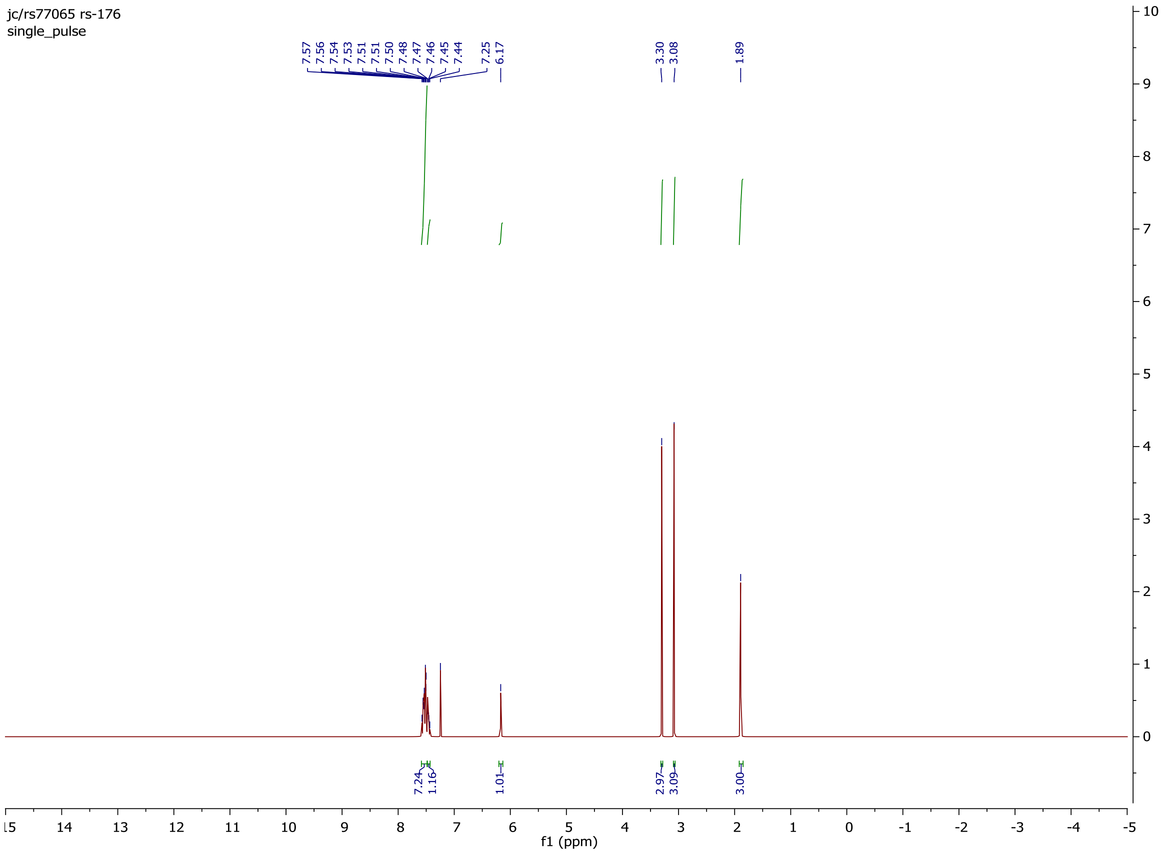

^{13}C NMR

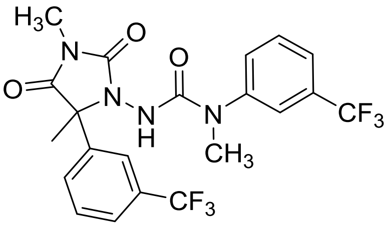

3-(3,5-Dimethyl-2,4-dioxo-5-(3-(trifluoromethyl)phenyl)imidazolidin-1-yl)-1-methyl-1-(3-(trifluoromethyl)phenyl)urea (4f)

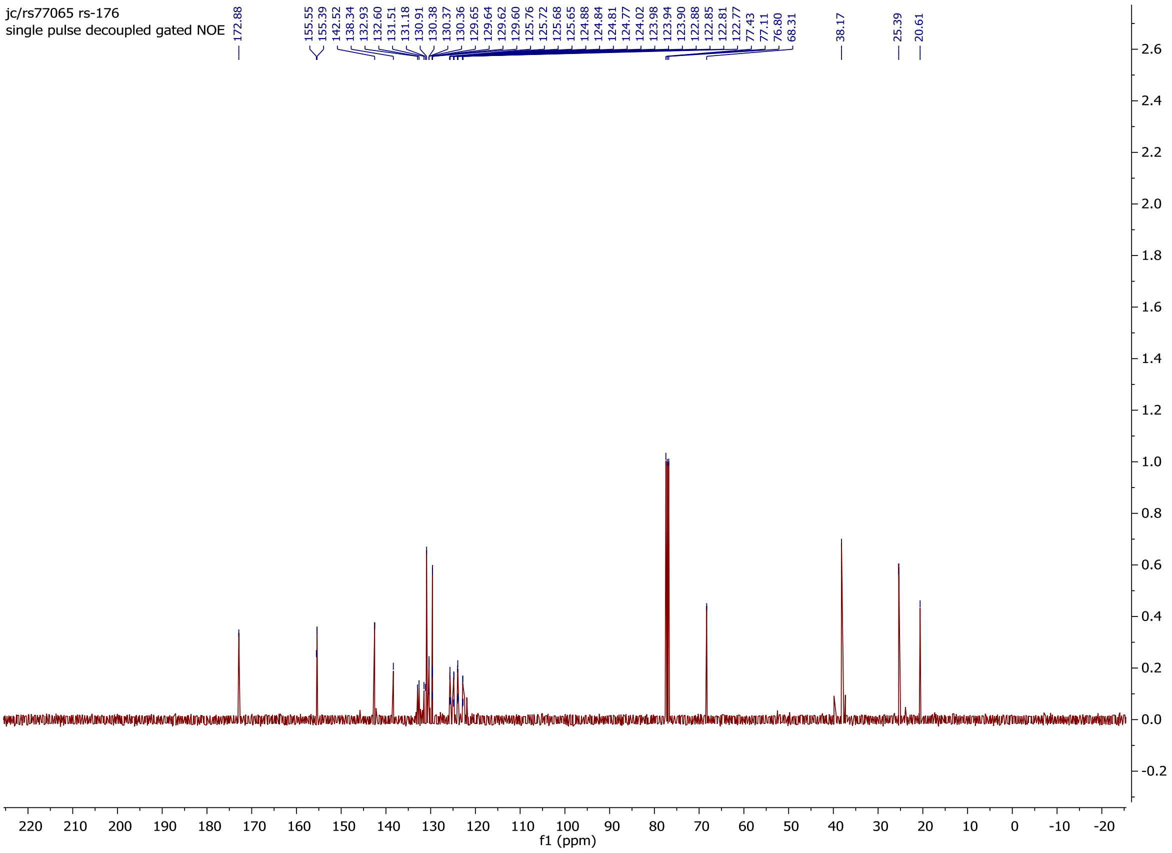

^1H NMR

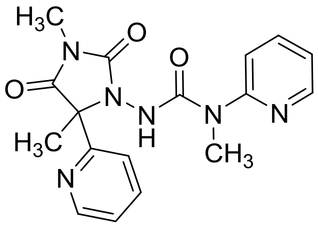

3-(3,5-Dimethyl-2,4-dioxo-5-(pyridin-2-yl)imidazolidin-1-yl)-1-methyl-1-(pyridin-2-yl)urea (4g)

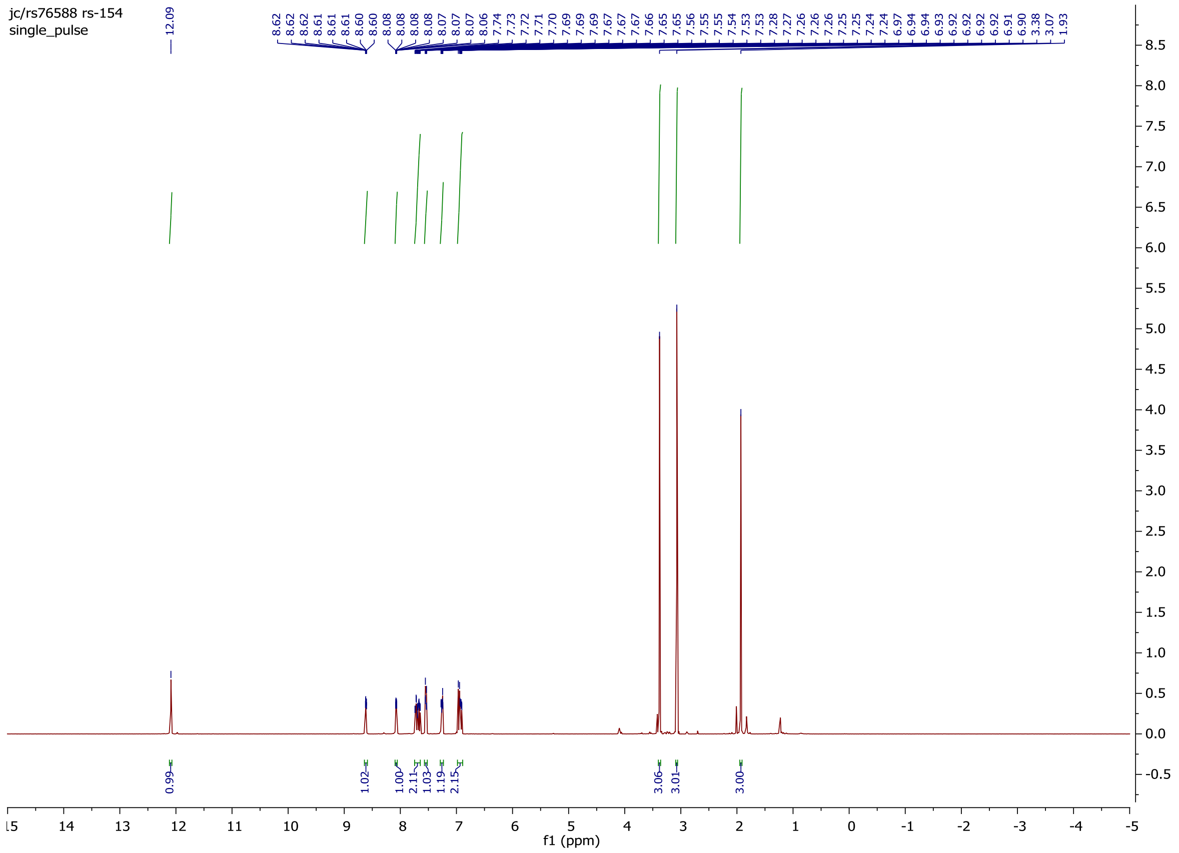

^{13}C NMR

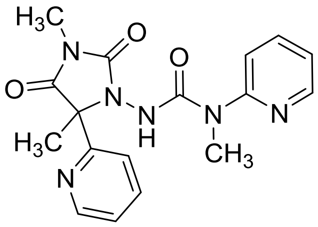

3-(3,5-Dimethyl-2,4-dioxo-5-(pyridin-2-yl)imidazolidin-1-yl)-1-methyl-1-(pyridin-2-yl)urea (4g)

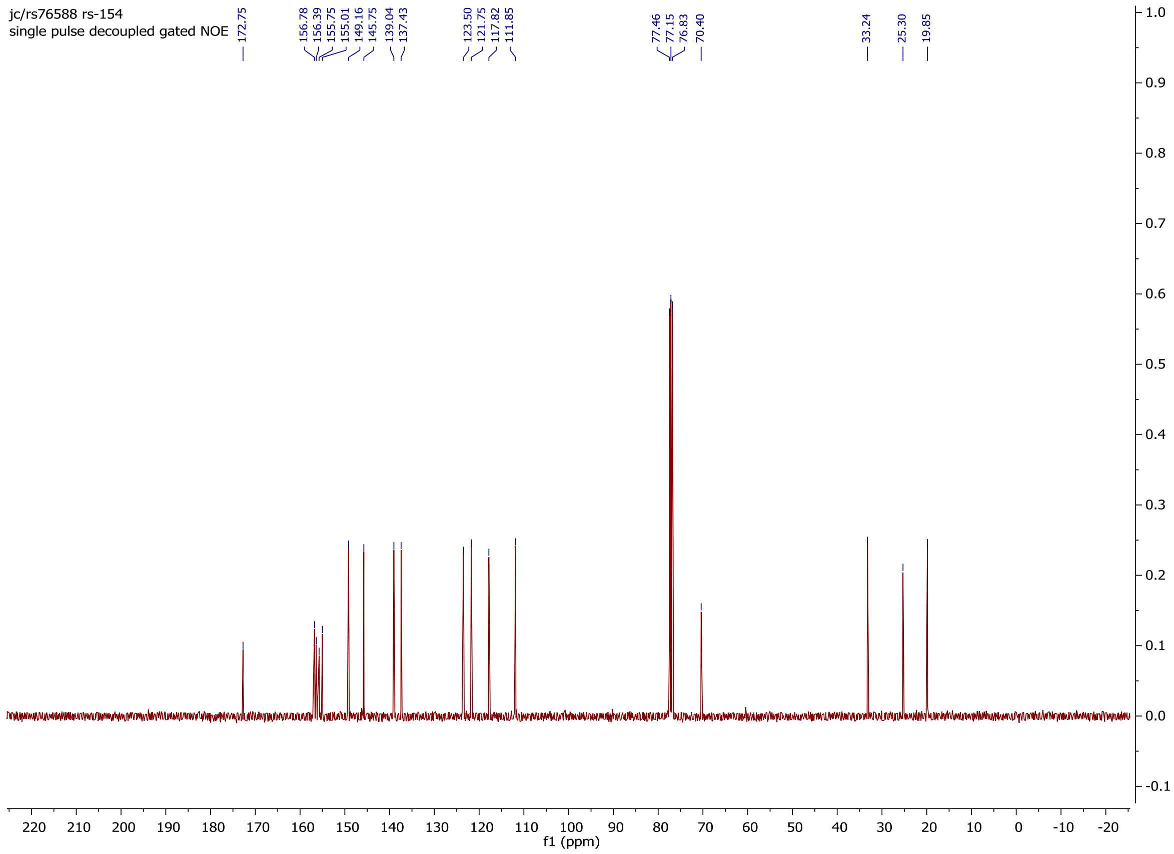

^{1}H NMR

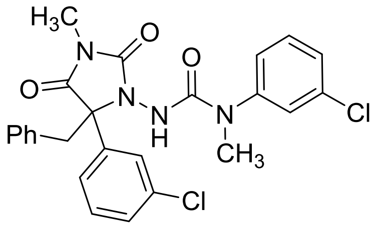

3-(5-Benzyl-5-(3-chlorophenyl)-3-methyl-2,4-dioxoimidazolidin-1-yl)-1-(3-chlorophenyl)-1-methylurea (4h)

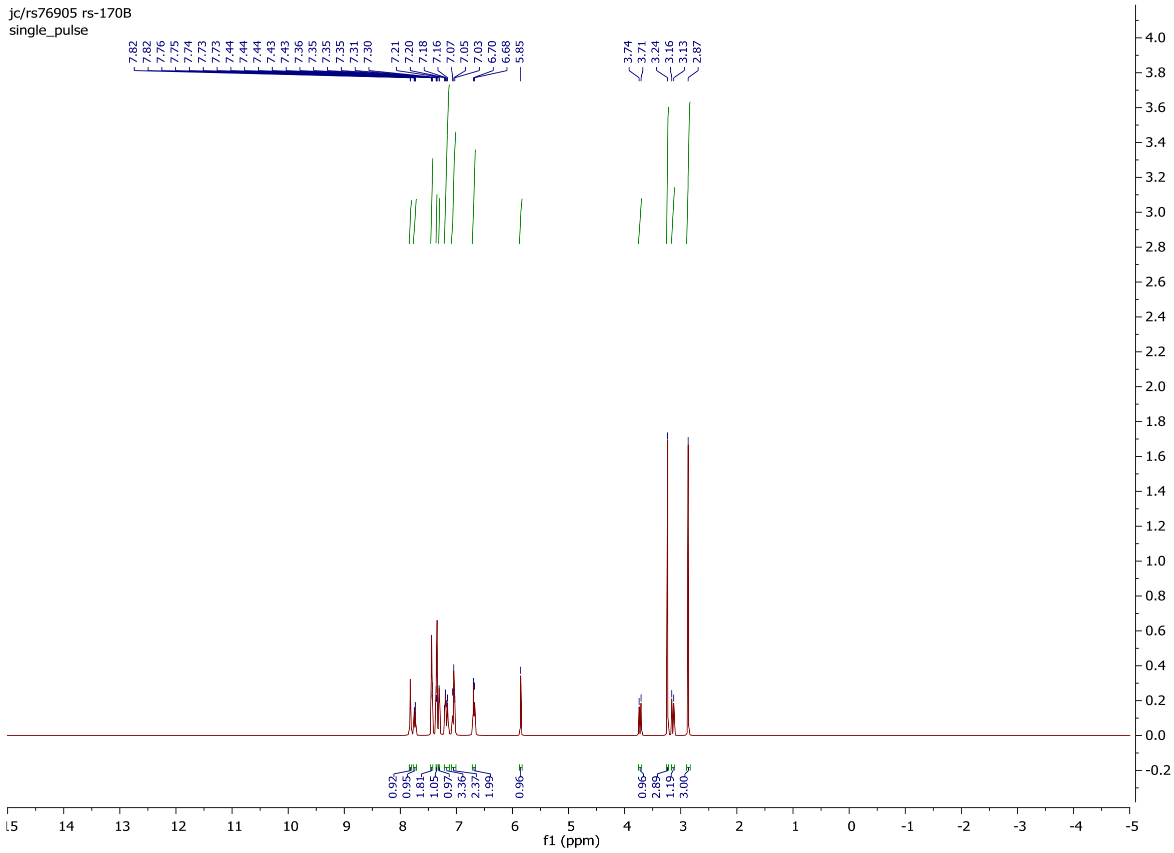

^{13}C NMR

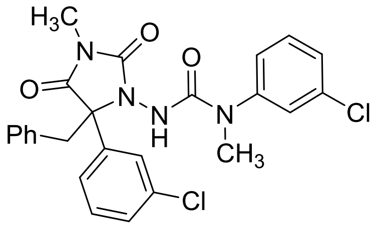

3-(5-Benzyl-5-(3-chlorophenyl)-3-methyl-2,4-dioxoimidazolidin-1-yl)-1-(3-chlorophenyl)-1-methylurea (4h)

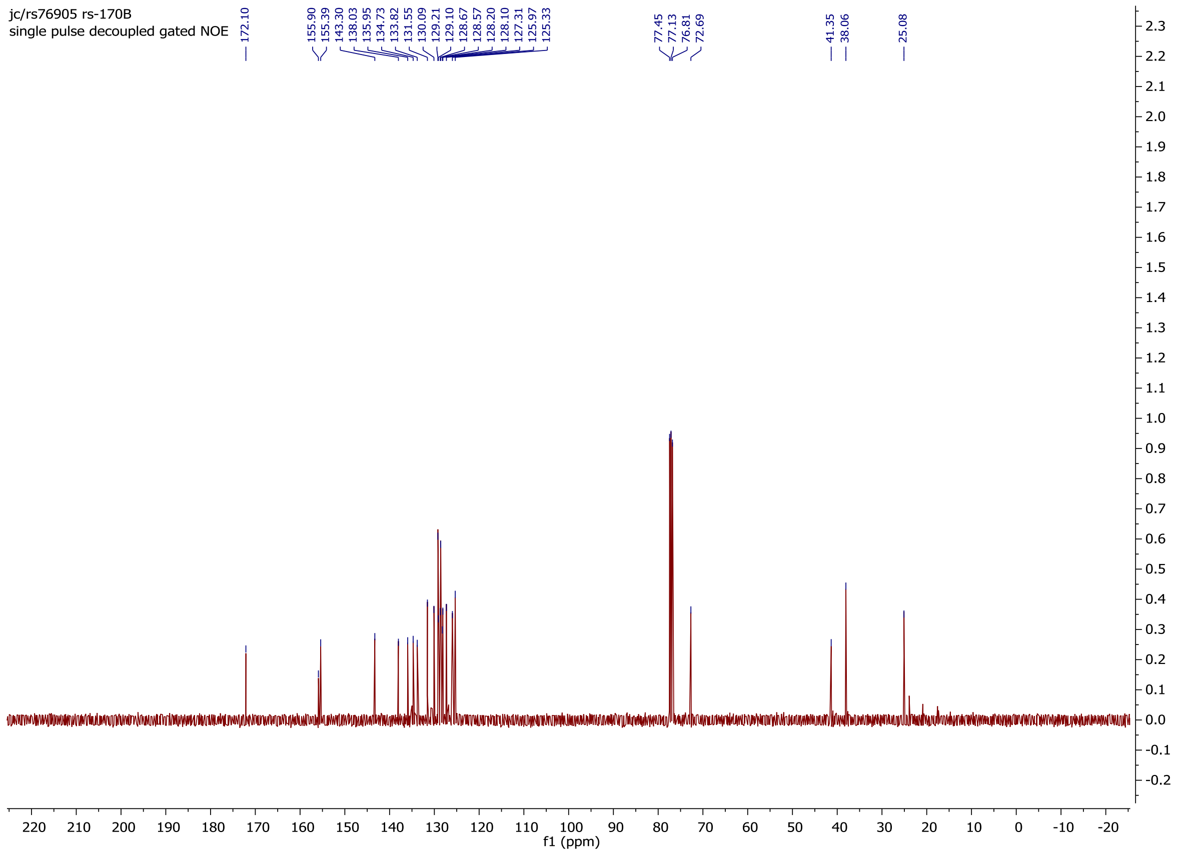

^{1}H NMR

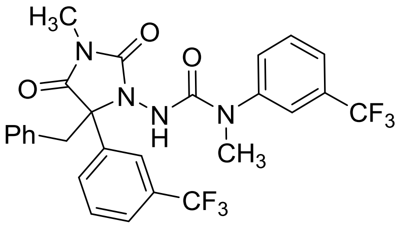

3-(5-Benzyl-3-methyl-2,4-dioxo-5-(3-(trifluoromethyl)phenyl)imidazolidin-1-yl)-1-methyl-1-(3-(trifluoromethyl)phenyl)urea (4i)

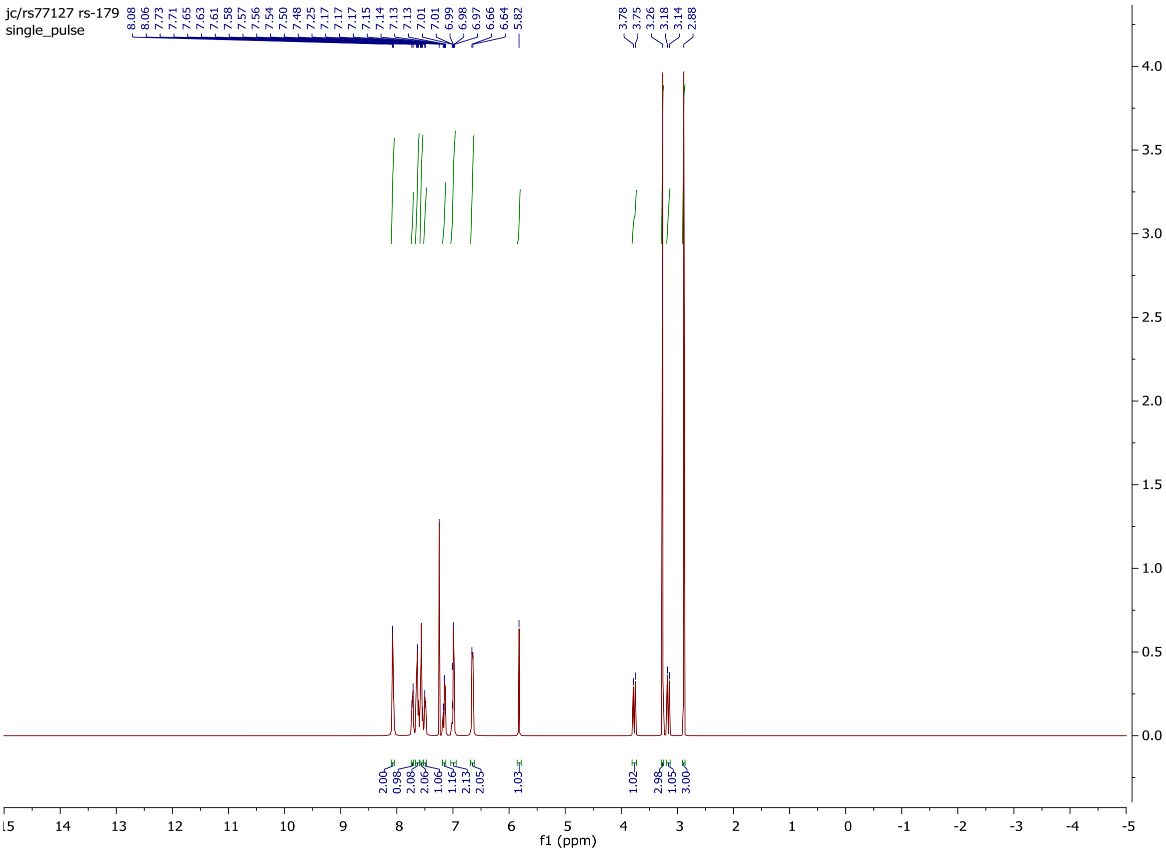

^{13}C NMR

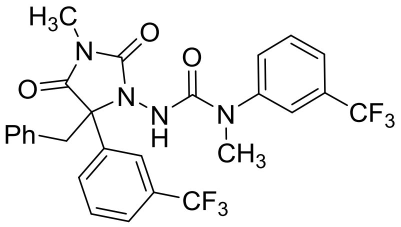

3-(5-Benzyl-3-methyl-2,4-dioxo-5-(3-(trifluoromethyl)phenyl)imidazolidin-1-yl)-1-methyl-1-(3-(trifluoromethyl)phenyl)urea (4i)

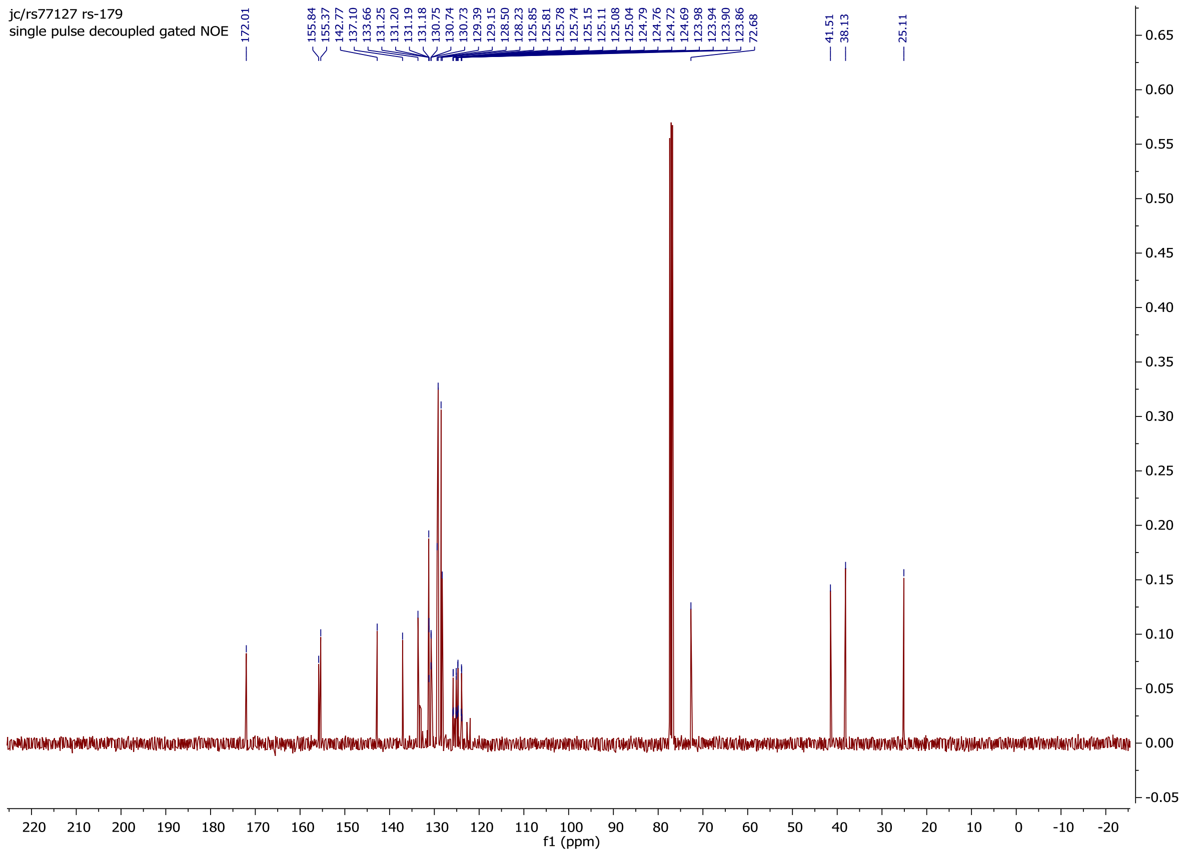

^{1}H NMR

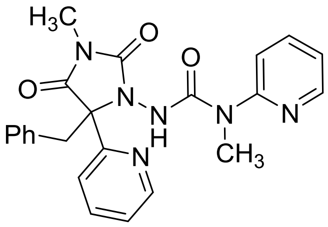

3-(5-Benzyl-3-methyl-2,4-dioxo-5-(pyridin-2-yl)imidazolidin-1-yl)-1-methyl-1-(pyridin-2-yl)urea (4j)

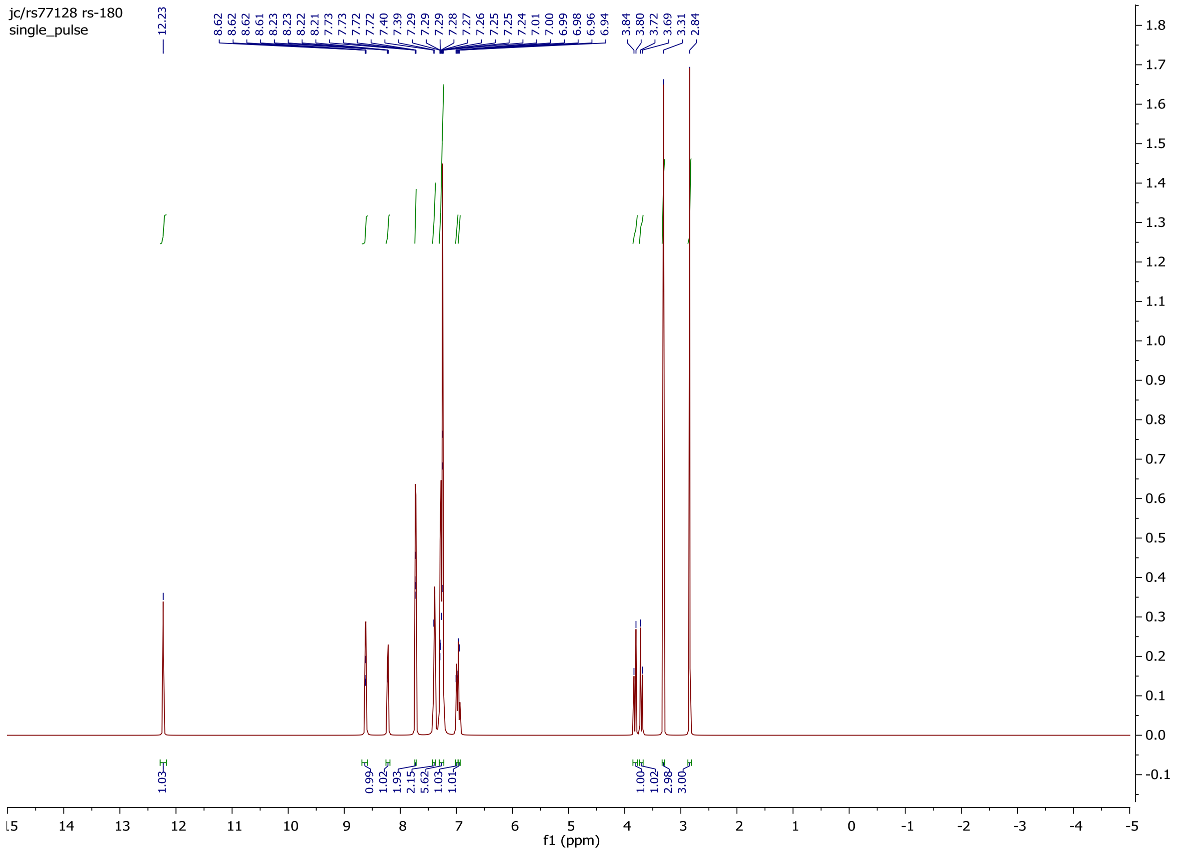

^{13}C NMR

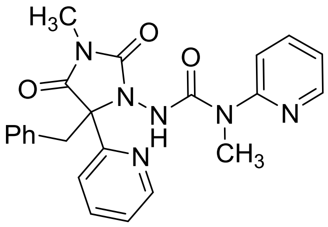

3-(5-Benzyl-3-methyl-2,4-dioxo-5-(pyridin-2-yl)imidazolidin-1-yl)-1-methyl-1-(pyridin-2-yl)urea (4j)

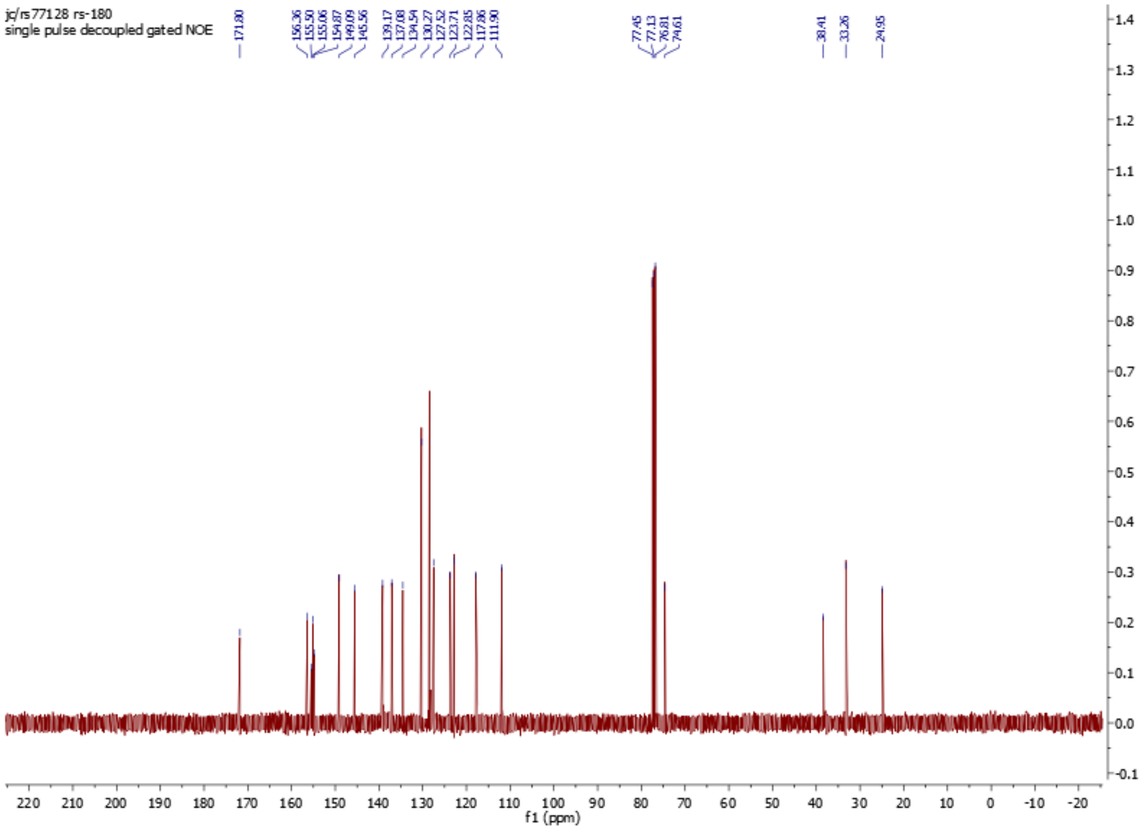

^{1}H NMR

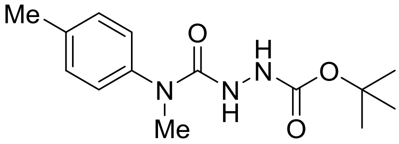

***tert*-Butyl 2-(methyl(p-tolyl)carbamoyl)hydrazine-1-carboxylate (S5a)**

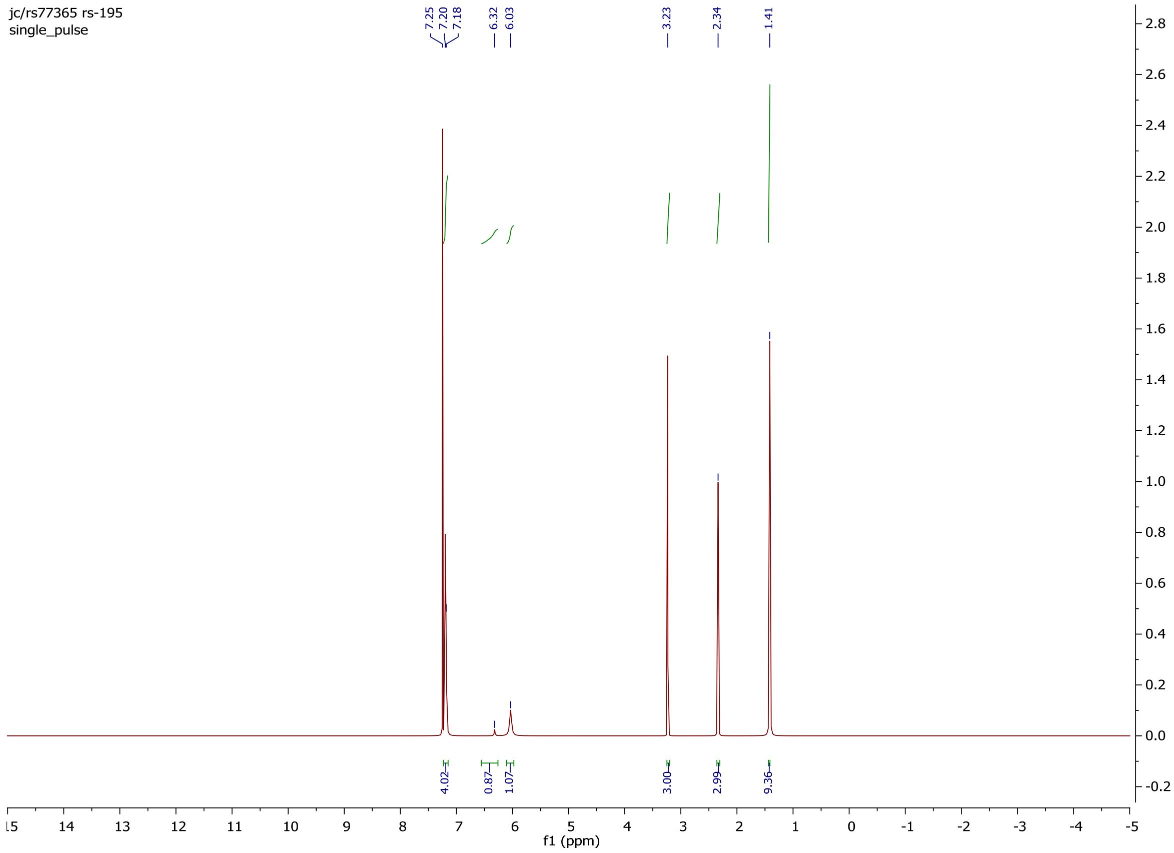

^{13}C NMR

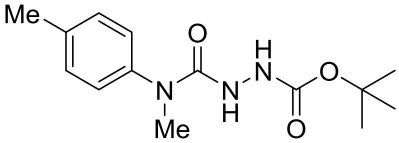

***tert*-Butyl 2-(methyl(p-tolyl)carbamoyl)hydrazine-1-carboxylate (S5a)**

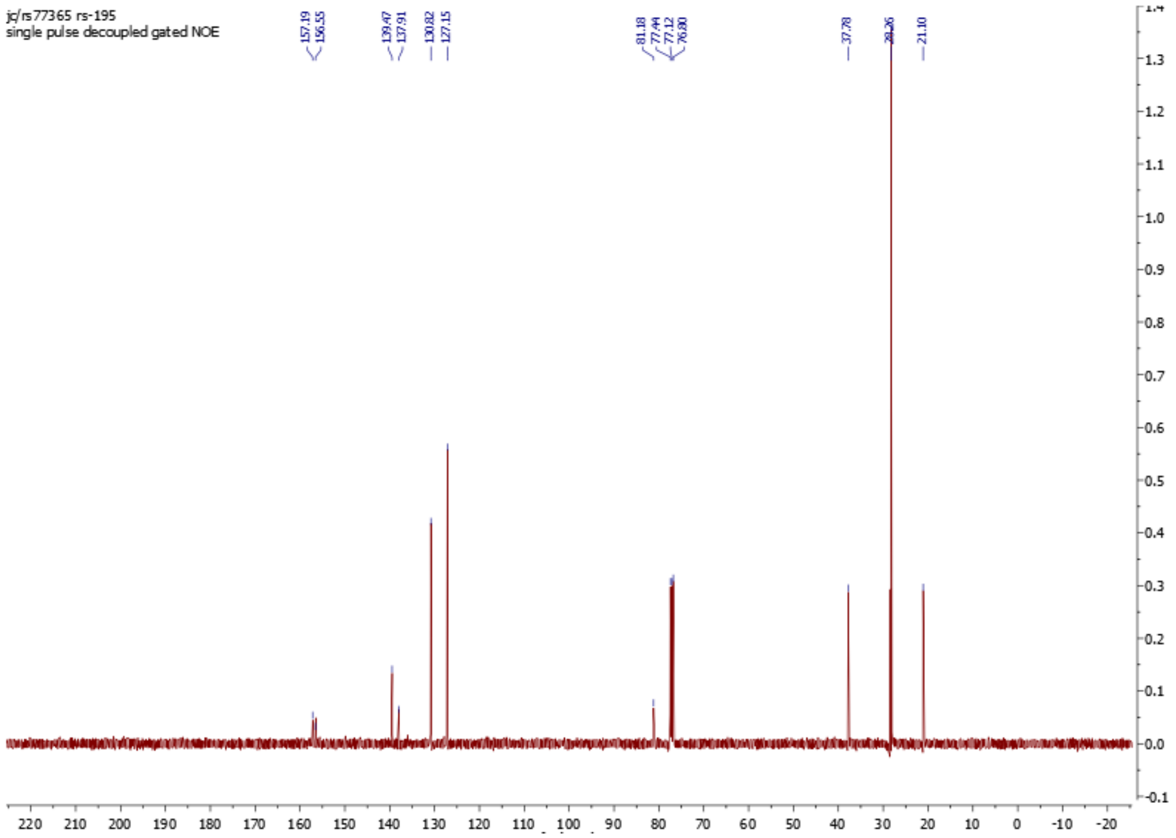

^{1}H NMR

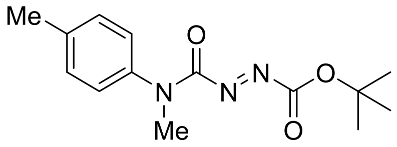

tert-Butyl (E)-2-(methyl(p-tolyl)carbamoyl)diazene-1-carboxylate (5a)

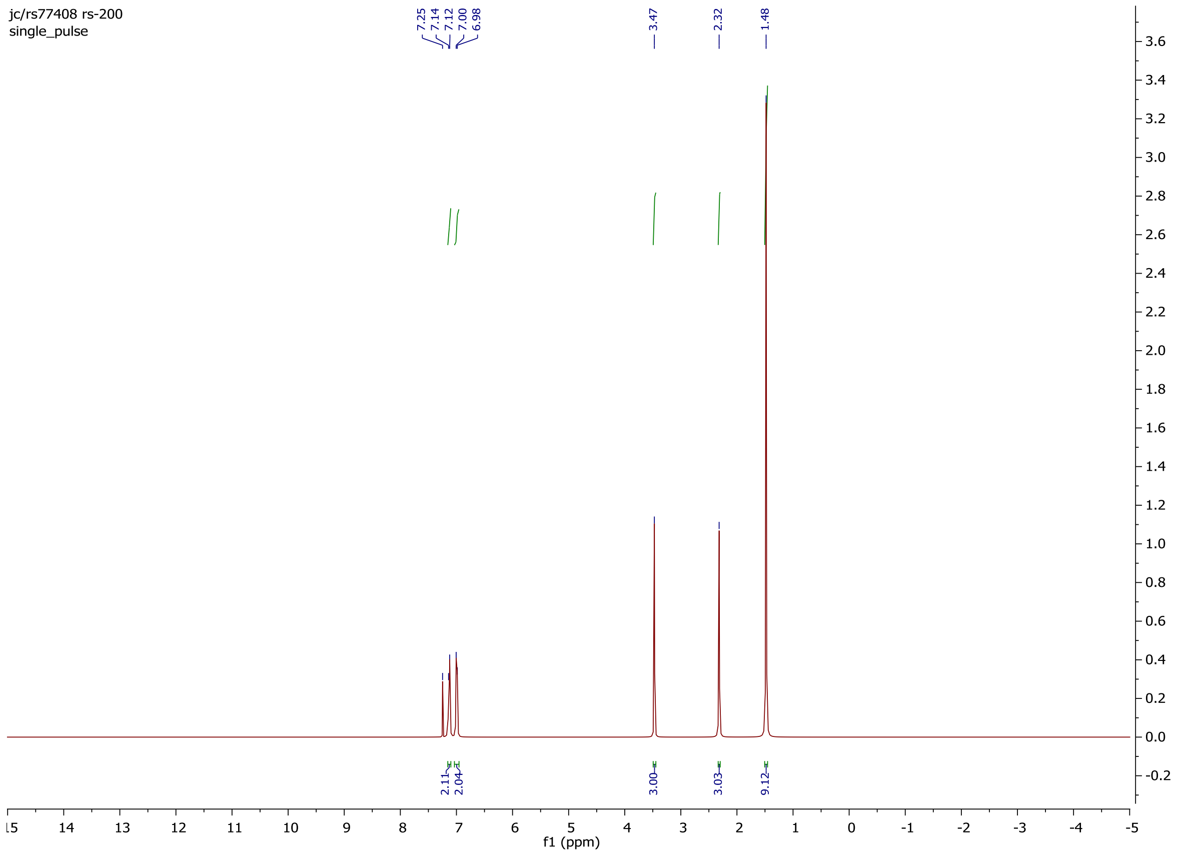

^{13}C NMR

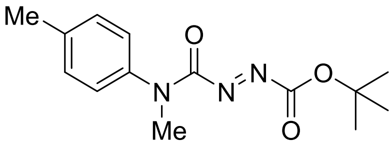

tert-Butyl (E)-2-(methyl(p-tolyl)carbamoyl)diazene-1-carboxylate (5a)

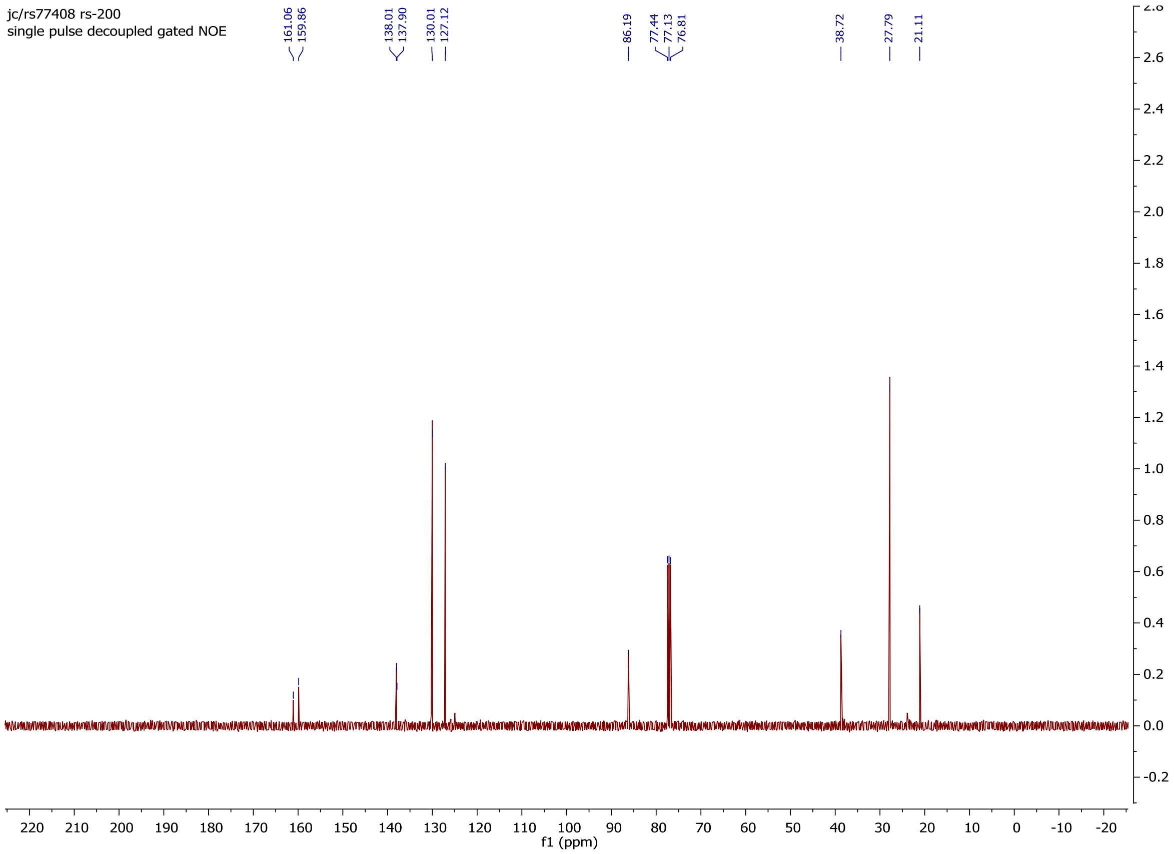

^{1}H NMR

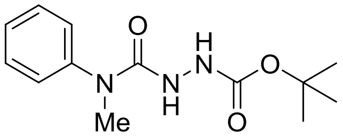

tert-Butyl 2-(methyl(phenyl)carbamoyl)hydrazine-1-carboxylate (S5b)

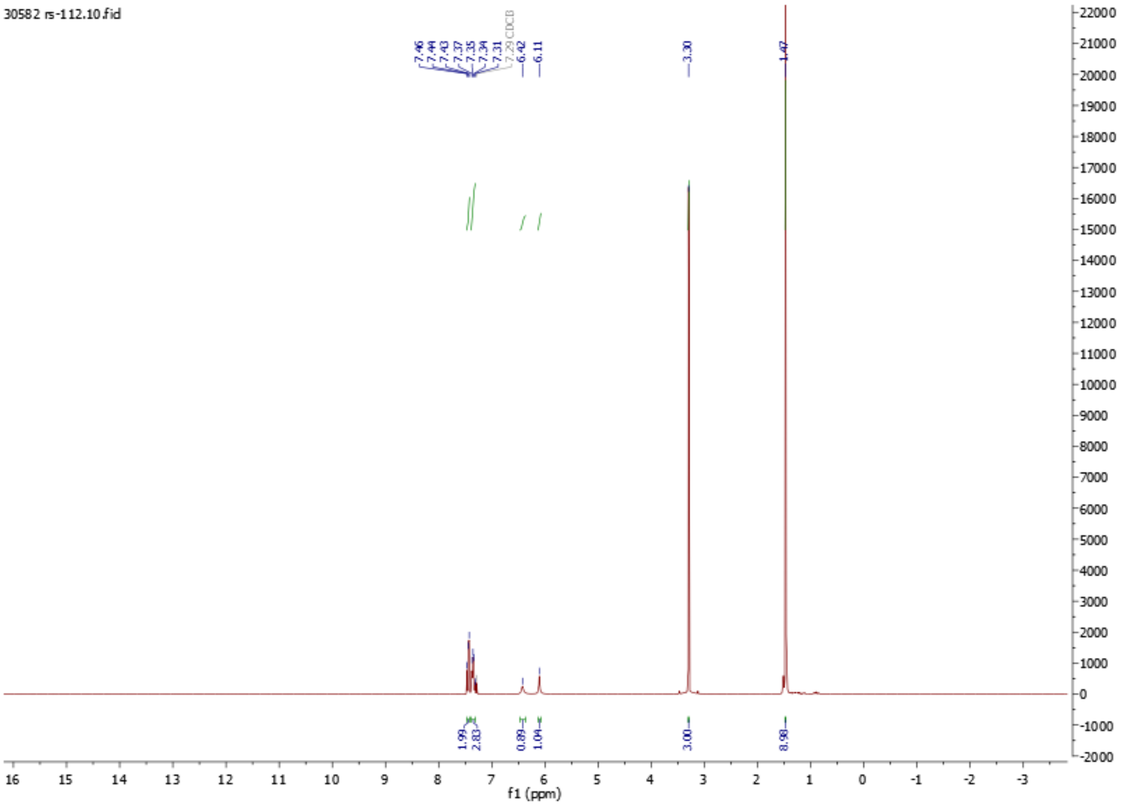

^{13}C NMR

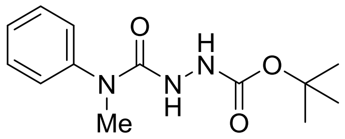

tert-Butyl 2-(methyl(phenyl)carbamoyl)hydrazine-1-carboxylate (S5b)

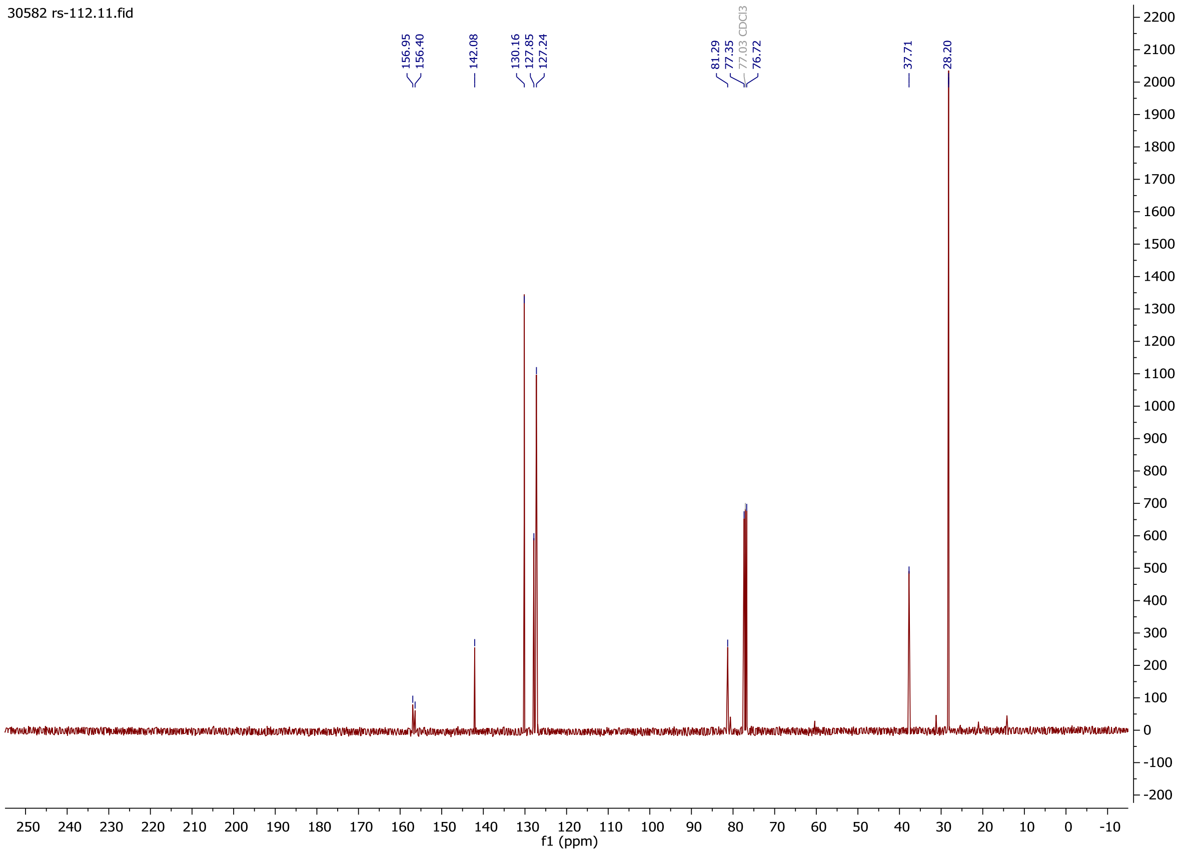

^{1}H NMR

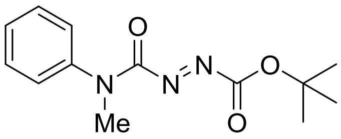

tert-Butyl (_E_)-2-(methyl(phenyl)carbamoyl)diazene-1-carboxylate (5b).

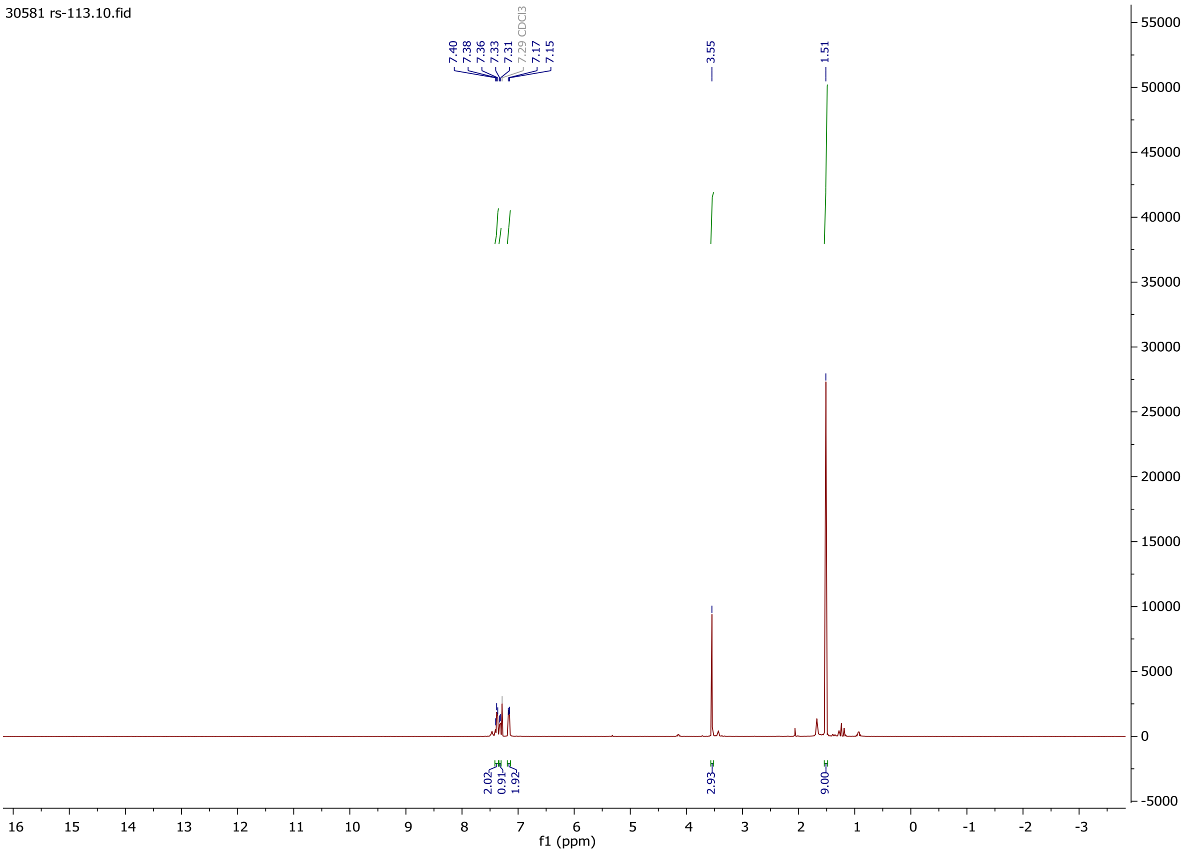

^{13}C NMR

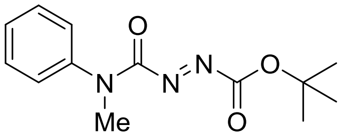

***tert*-Butyl (*E*)-2-(methyl(phenyl)carbamoyl)diazene-1-carboxylate (5b).**

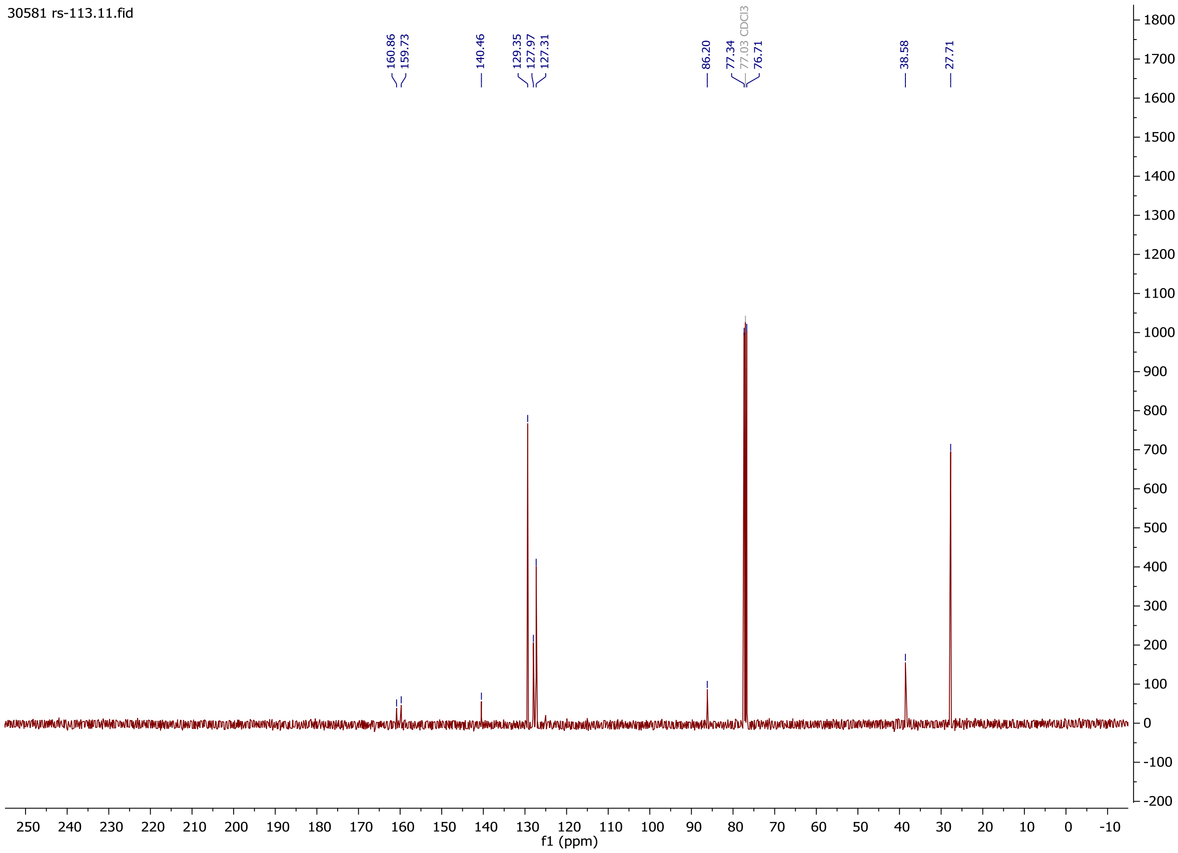

^{1}H NMR

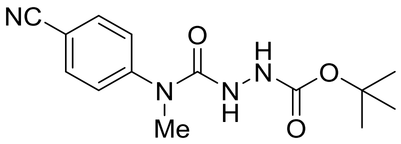

***tert*-Butyl 2-((4-cyanophenyl)(methyl)carbamoyl)hydrazine-1-carboxylate (S5c)**

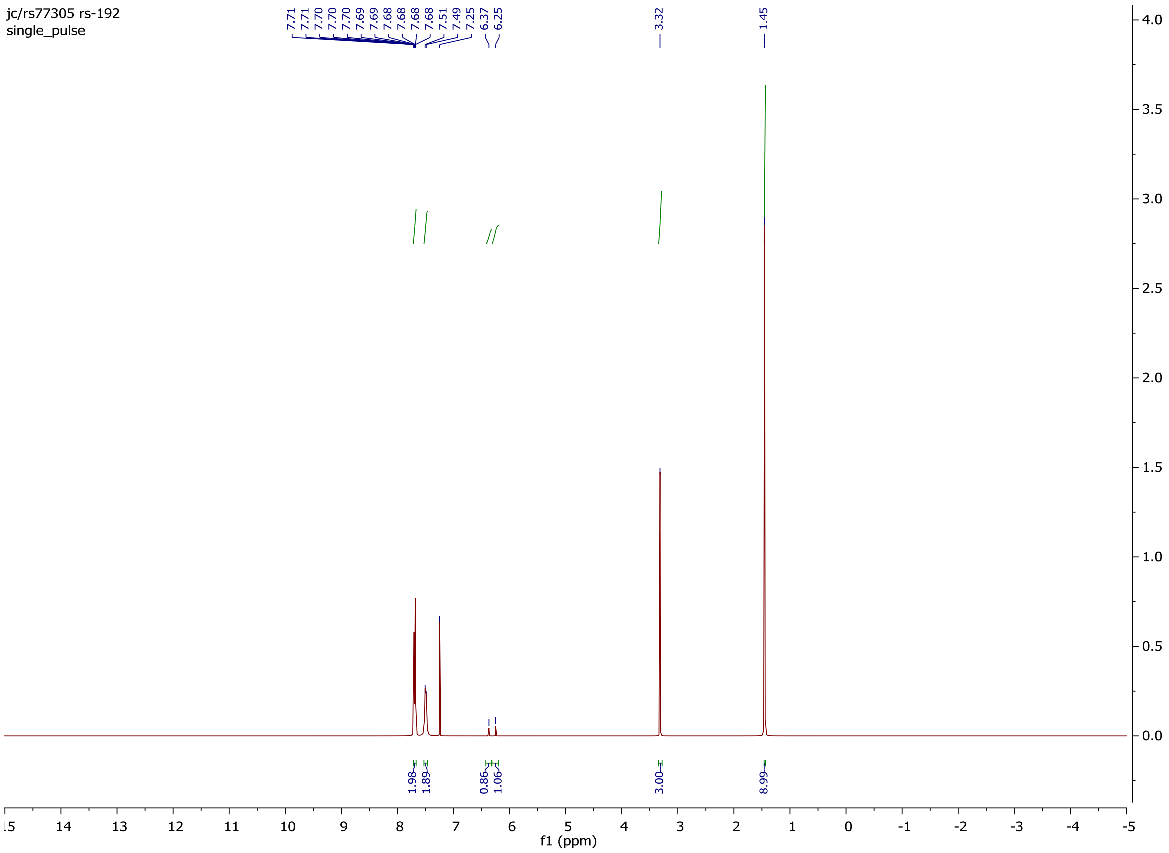

^{13}C NMR

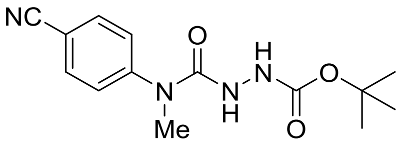

***tert*-Butyl 2-((4-cyanophenyl)(methyl)carbamoyl)hydrazine-1-carboxylate (S5c)**

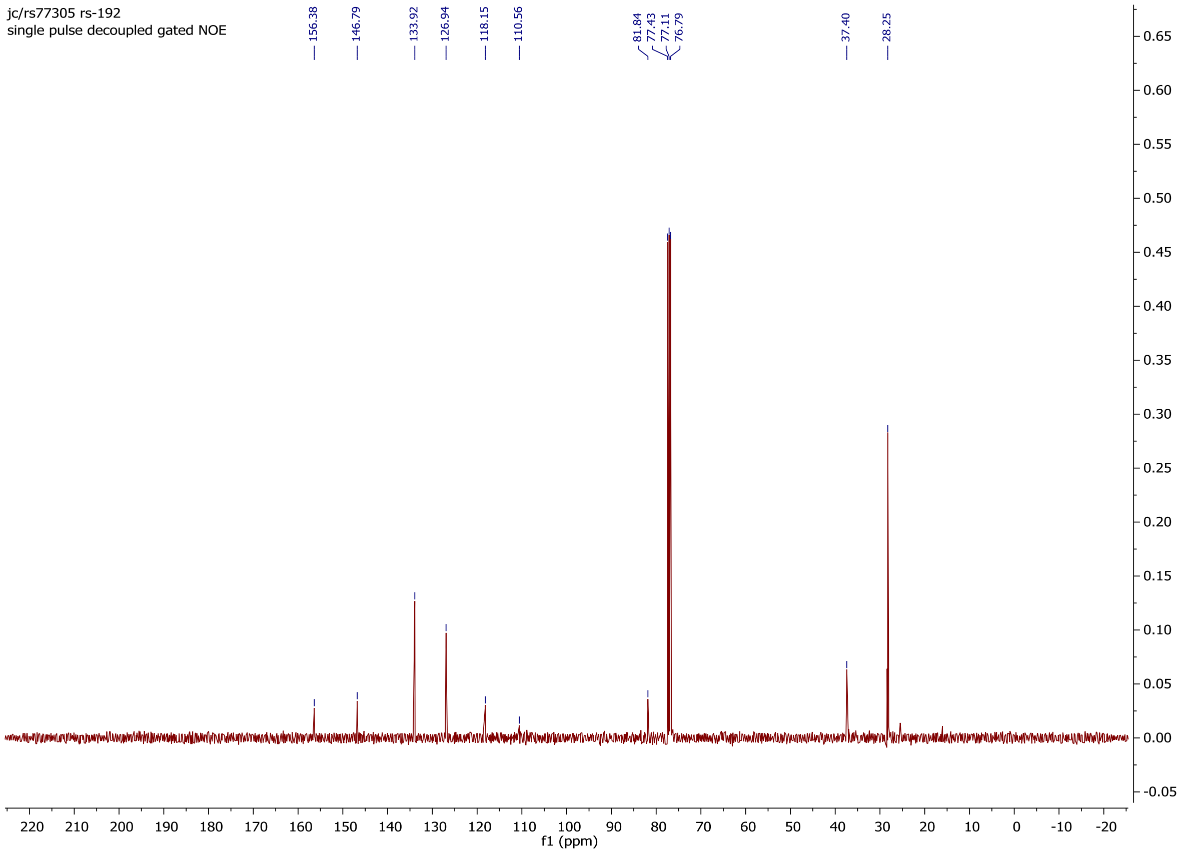

^{1}H NMR

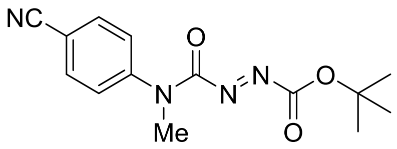

tert-Butyl (E)-2-((4-cyanophenyl)(methyl)carbamoyl)diazene-1-carboxylate (5c).

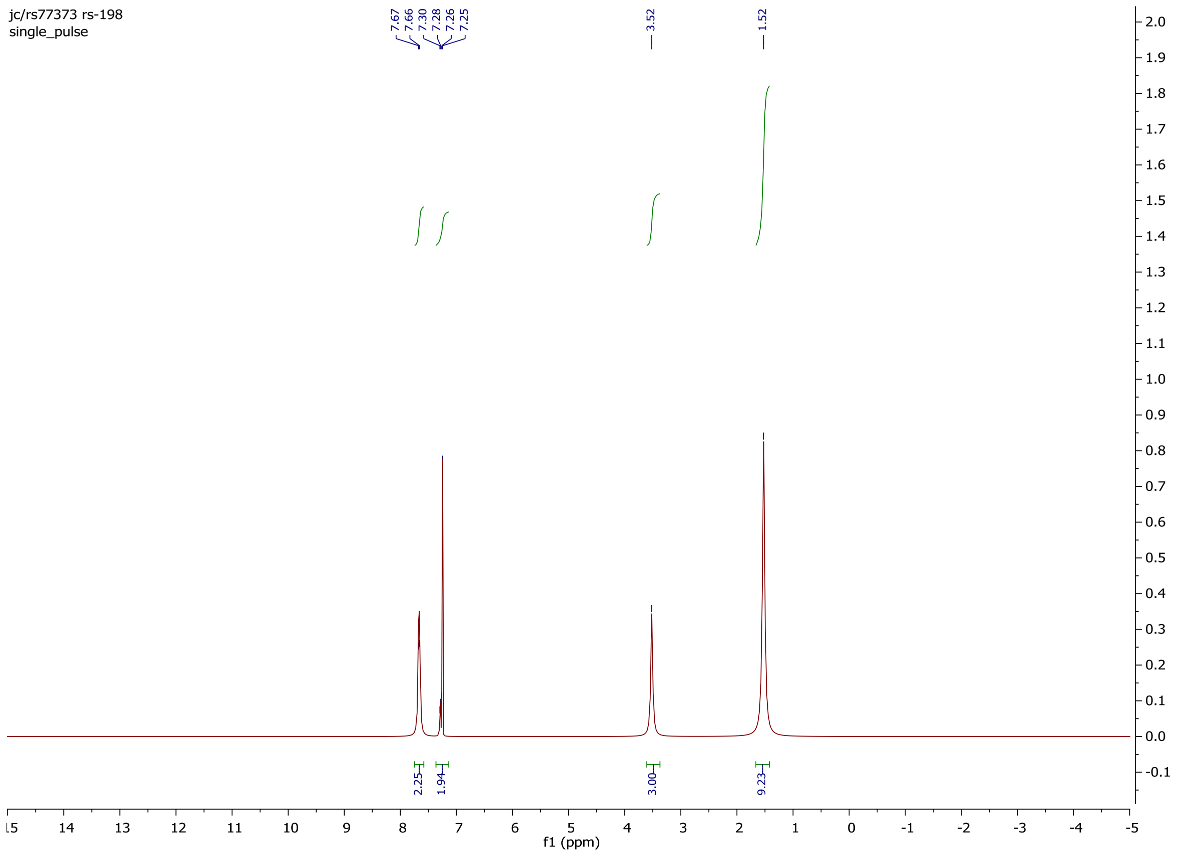

^{13}C NMR

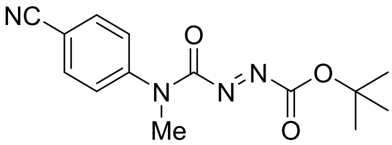

tert-Butyl (E)-2-((4-cyanophenyl)(methyl)carbamoyl)diazene-1-carboxylate (5c).

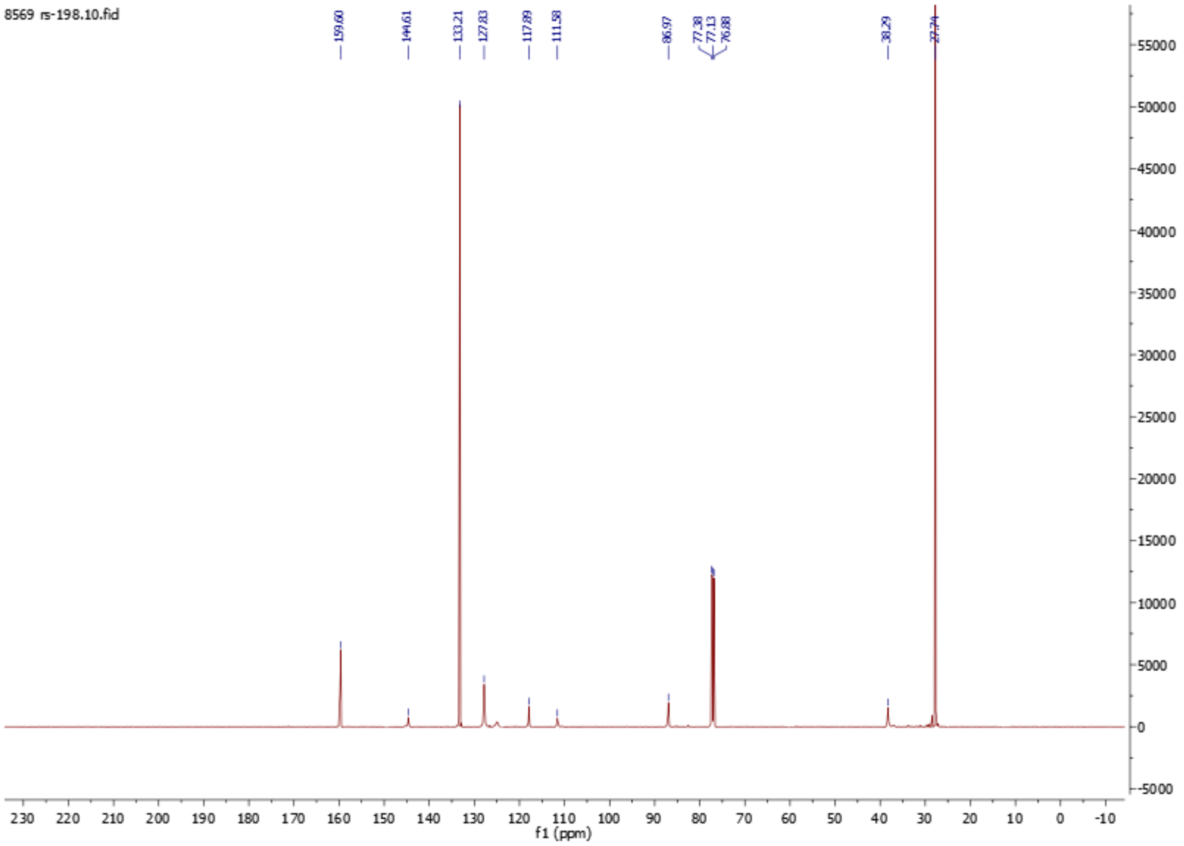

^{1}H NMR

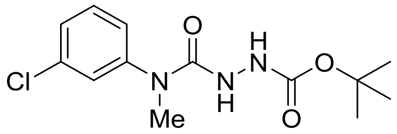

***tert*-Butyl 2-((3-chlorophenyl)(methyl)carbamoyl)hydrazine-1-carboxylate (S5d)**

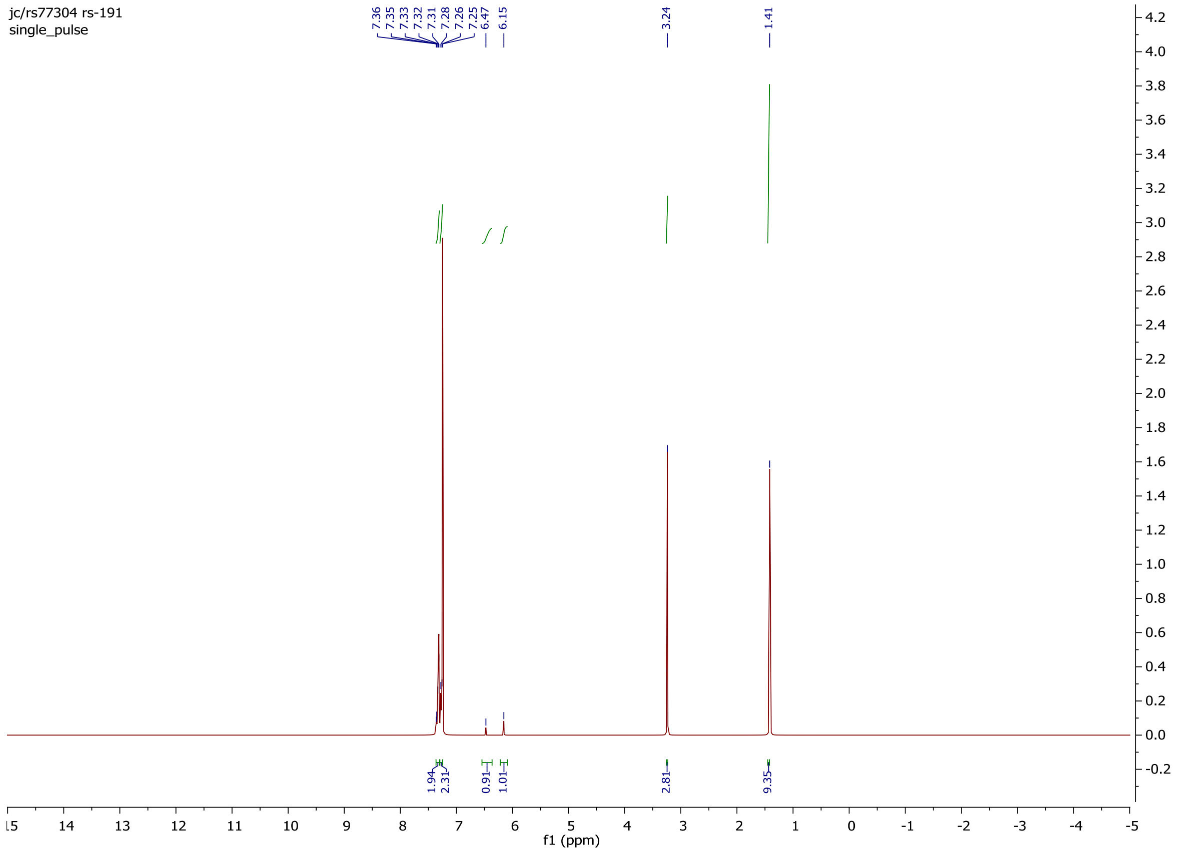

^{13}C NMR

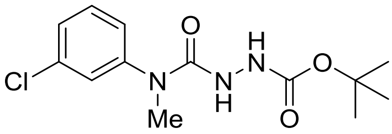

tert-Butyl 2-((3-chlorophenyl)(methyl)carbamoyl)hydrazine-1-carboxylate (S5d)

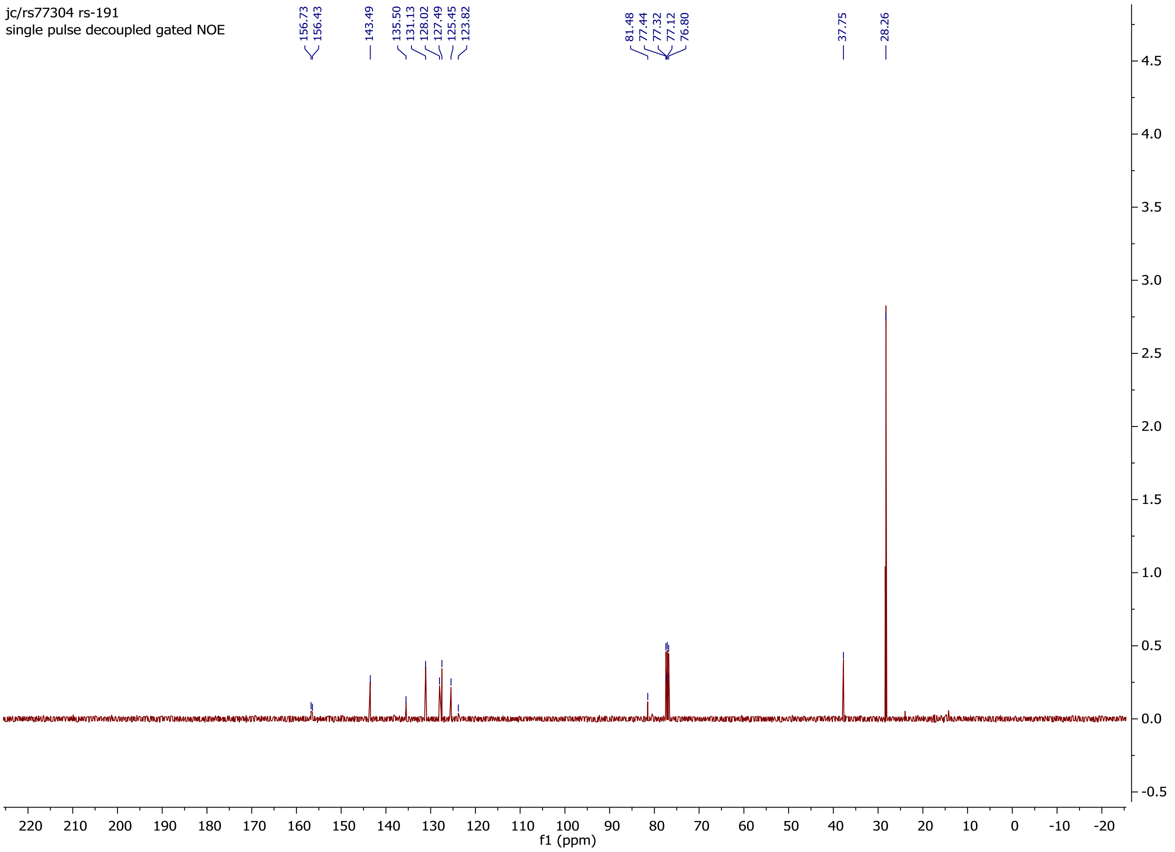

^{1}H NMR

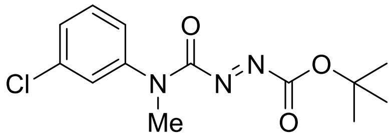

***tert*-Butyl (E)-2-((3-chlorophenyl)(methyl)carbamoyl)diazene-1-carboxylate (5d).**

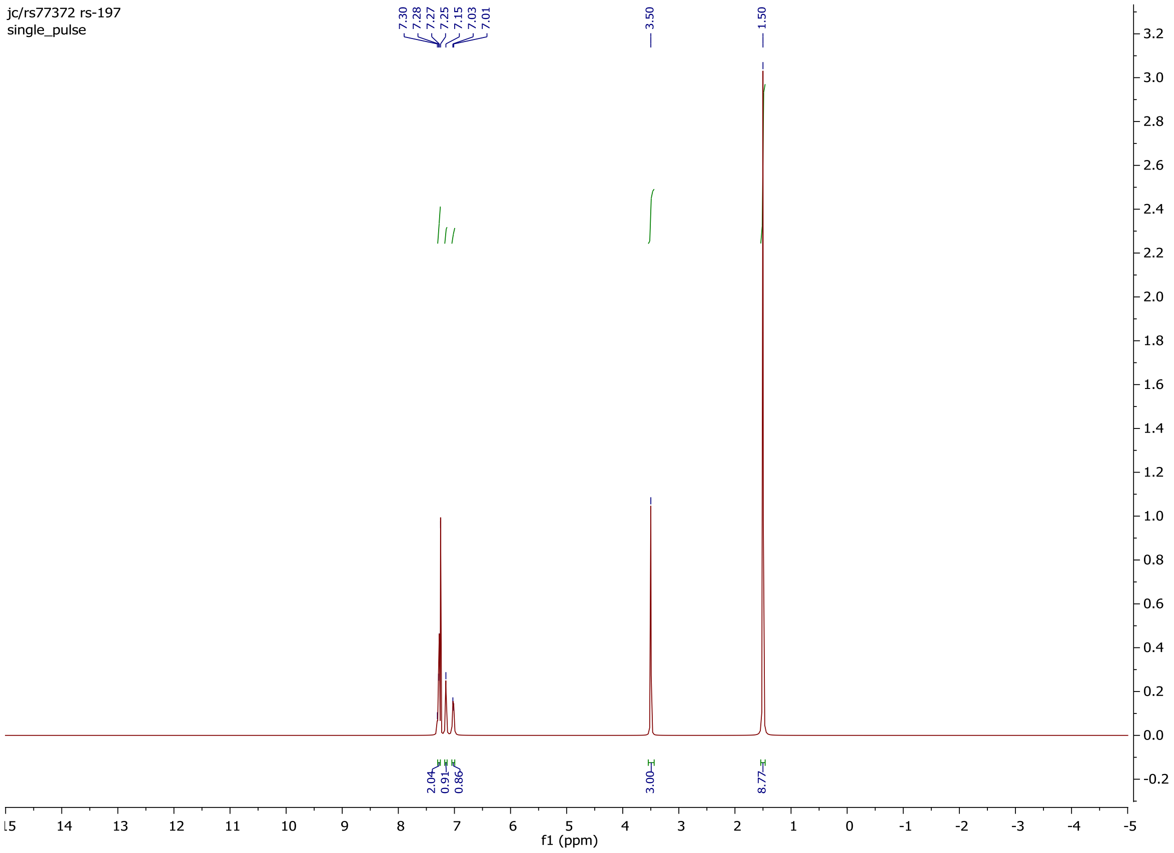

^{13}C NMR

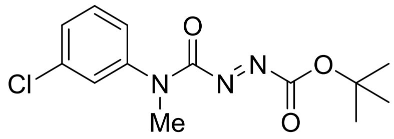

***tert*-Butyl (E)-2-((3-chlorophenyl)(methyl)carbamoyl)diazene-1-carboxylate (5d).**

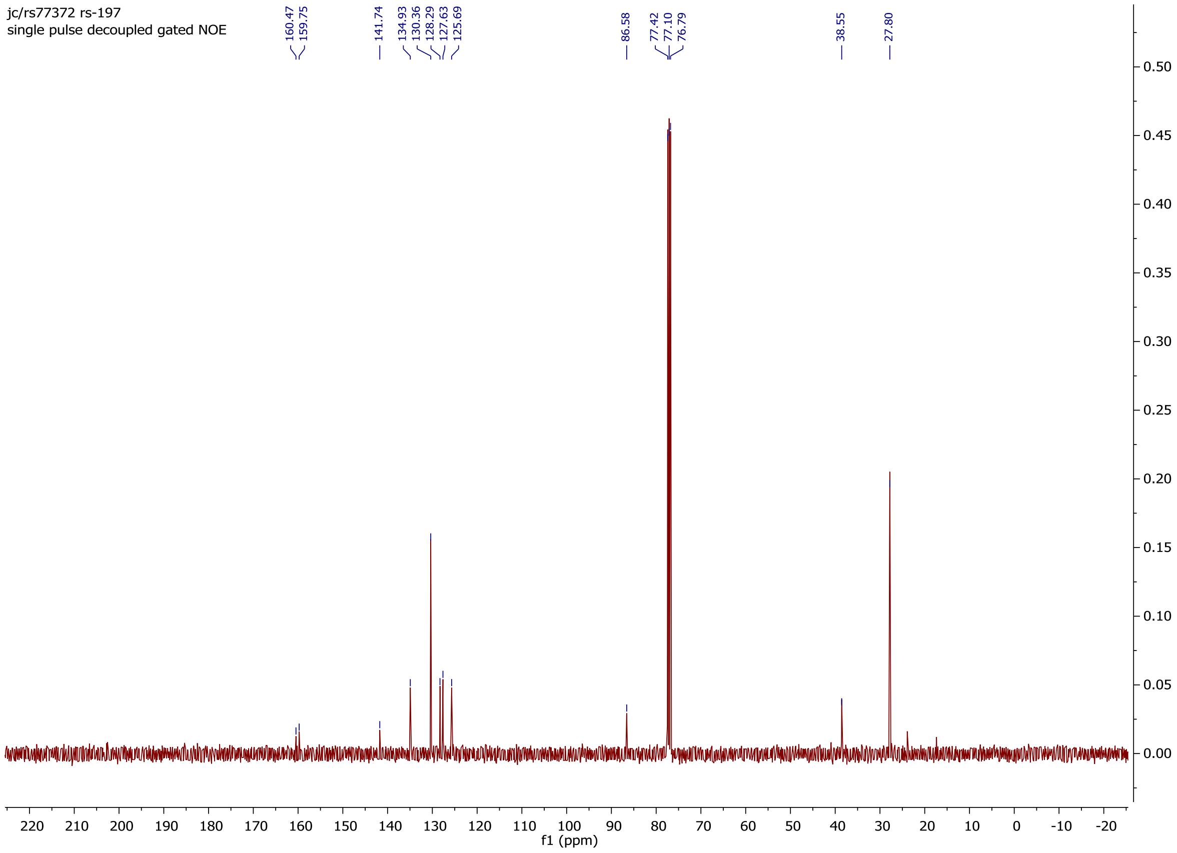

^{1}H NMR

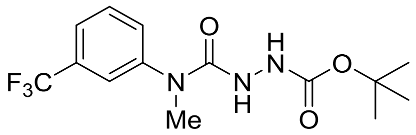

tert-Butyl 2-(methyl(3-(trifluoromethyl)phenyl)carbamoyl)hydrazine-1-carboxylate

(S5e)

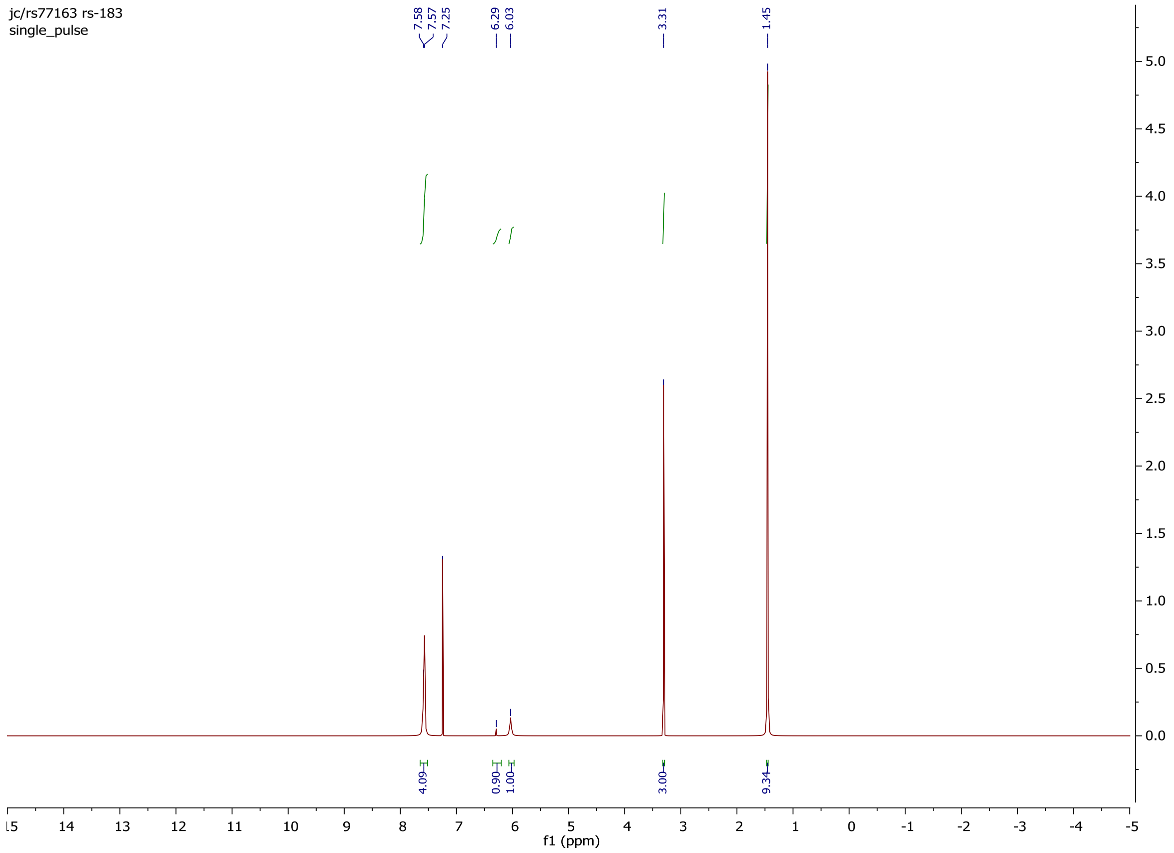

^{13}C NMR

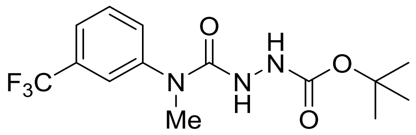

tert-Butyl 2-(methyl(3-(trifluoromethyl)phenyl)carbamoyl)hydrazine-1-carboxylate (S5e)

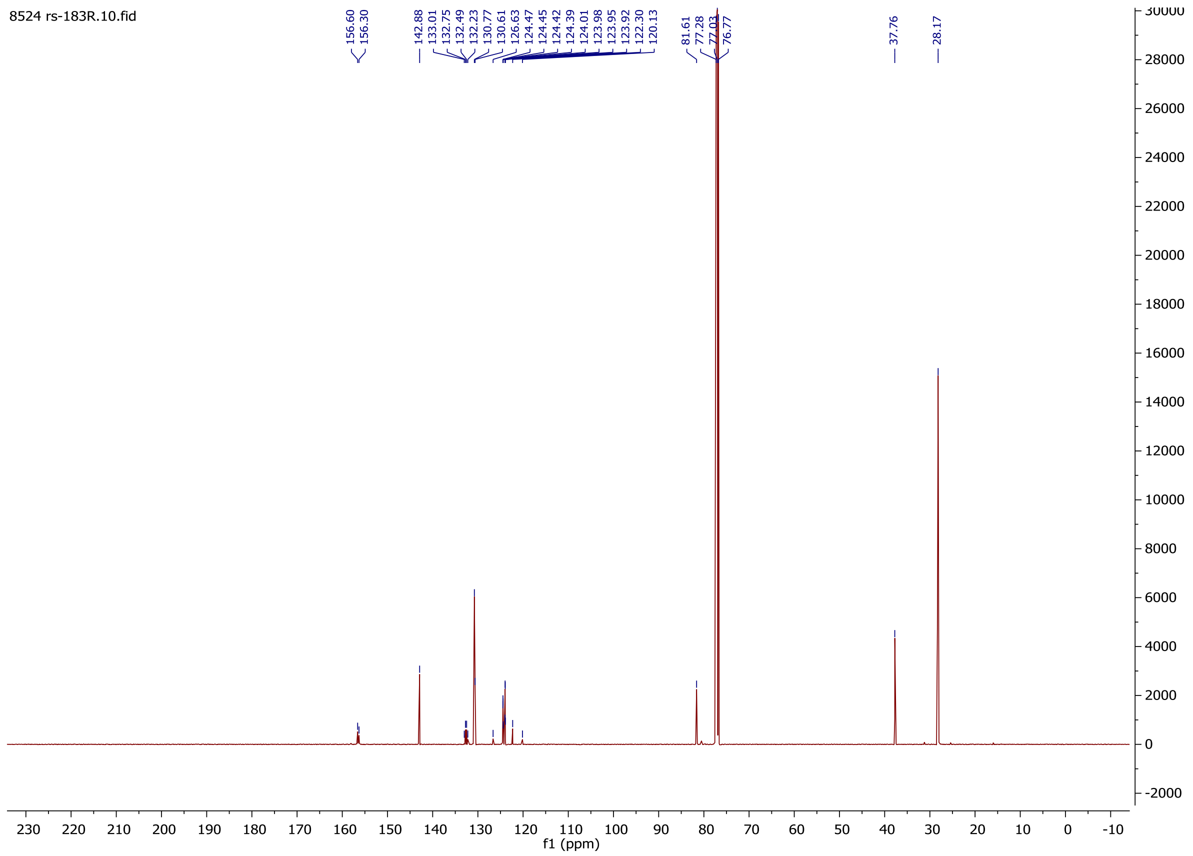

^{1}H NMR

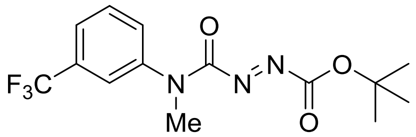

***tert*-Butyl (*E*)-2-(methyl(3-(trifluoromethyl)phenyl)carbamoyl)diazene-1-carboxylate**

(5e).

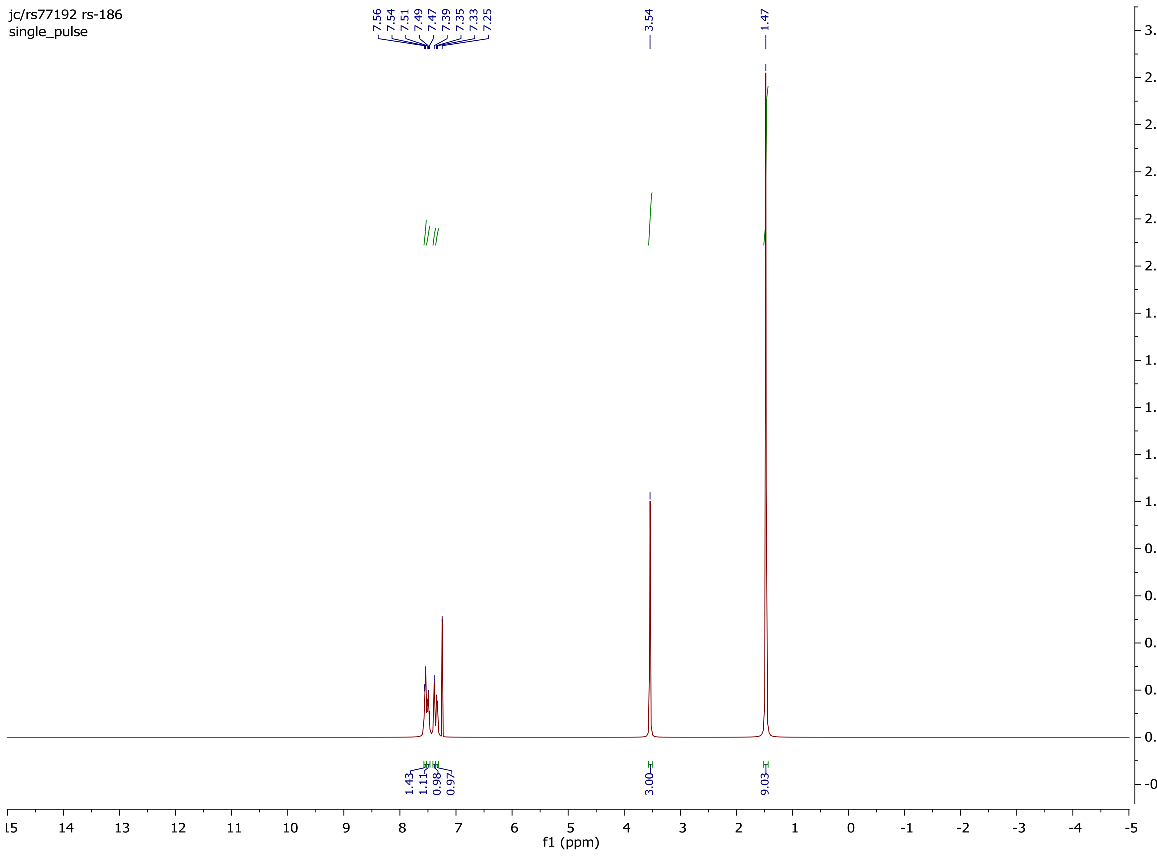

^{13}C NMR

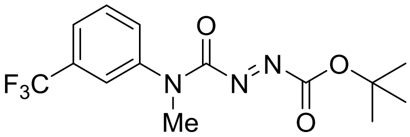

***tert*-Butyl (*E*)-2-(methyl(3-(trifluoromethyl)phenyl)carbamoyl)diazene-1-carboxylate (5e).**

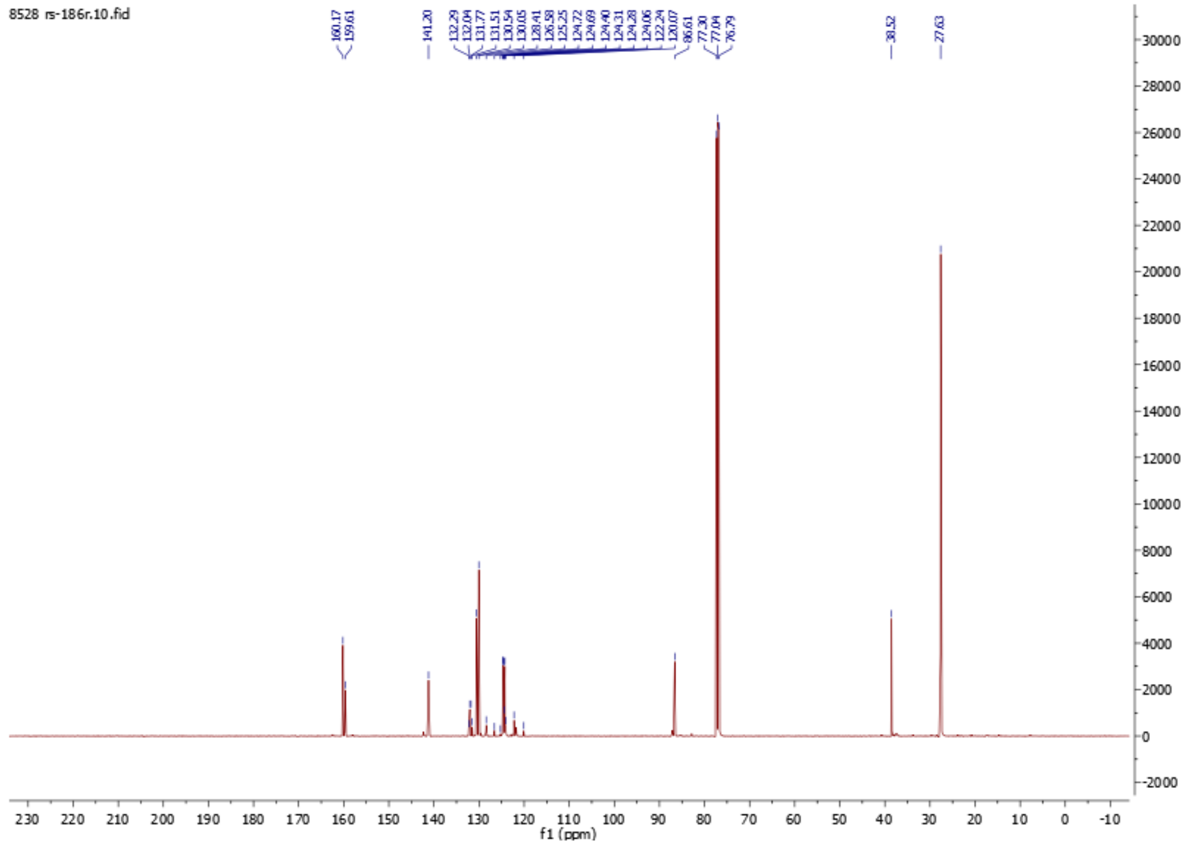

^{1}H NMR

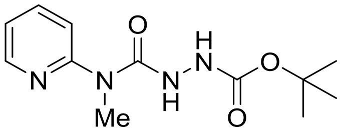

tert-Butyl 2-(methyl(pyridin-2-yl)carbamoyl)hydrazine-1-carboxylate (S5f)

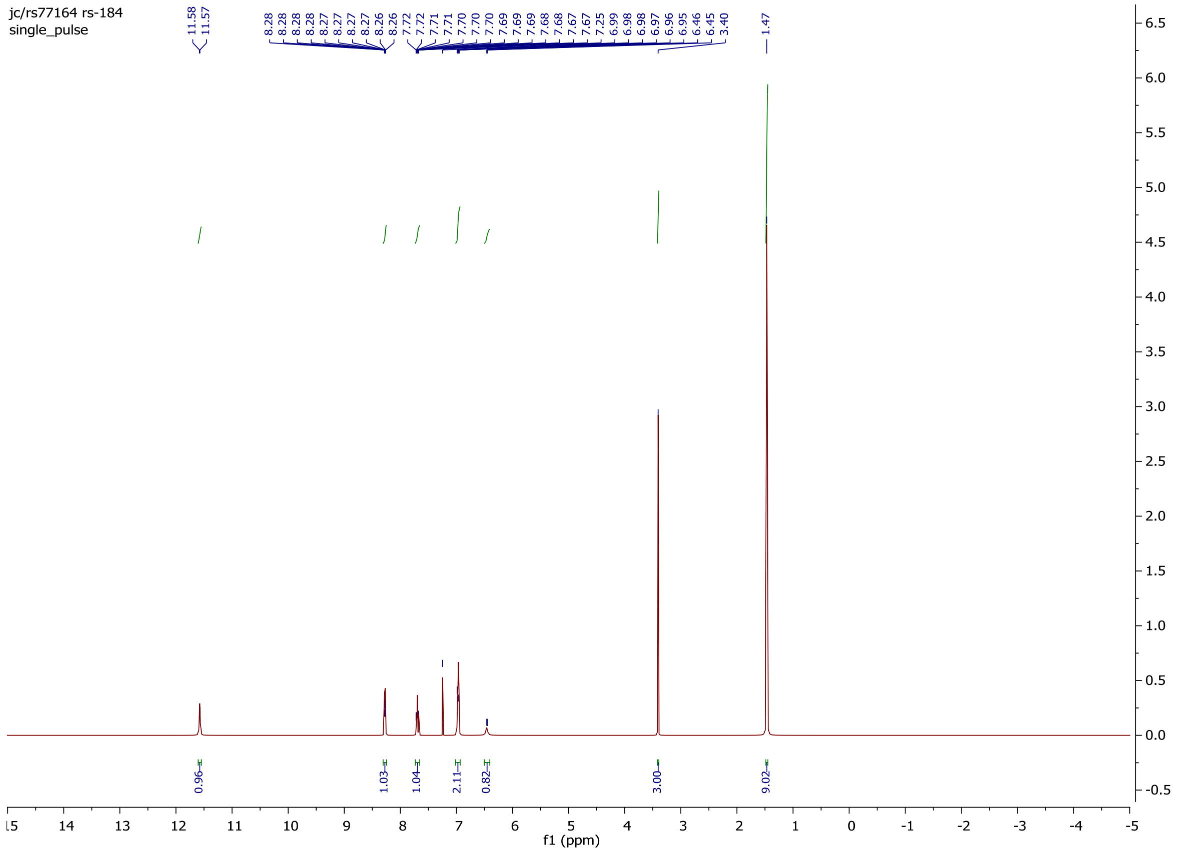

^{13}C NMR

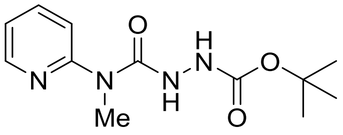

***tert*-Butyl 2-(methyl(pyridin-2-yl)carbamoyl)hydrazine-1-carboxylate (S5f)**

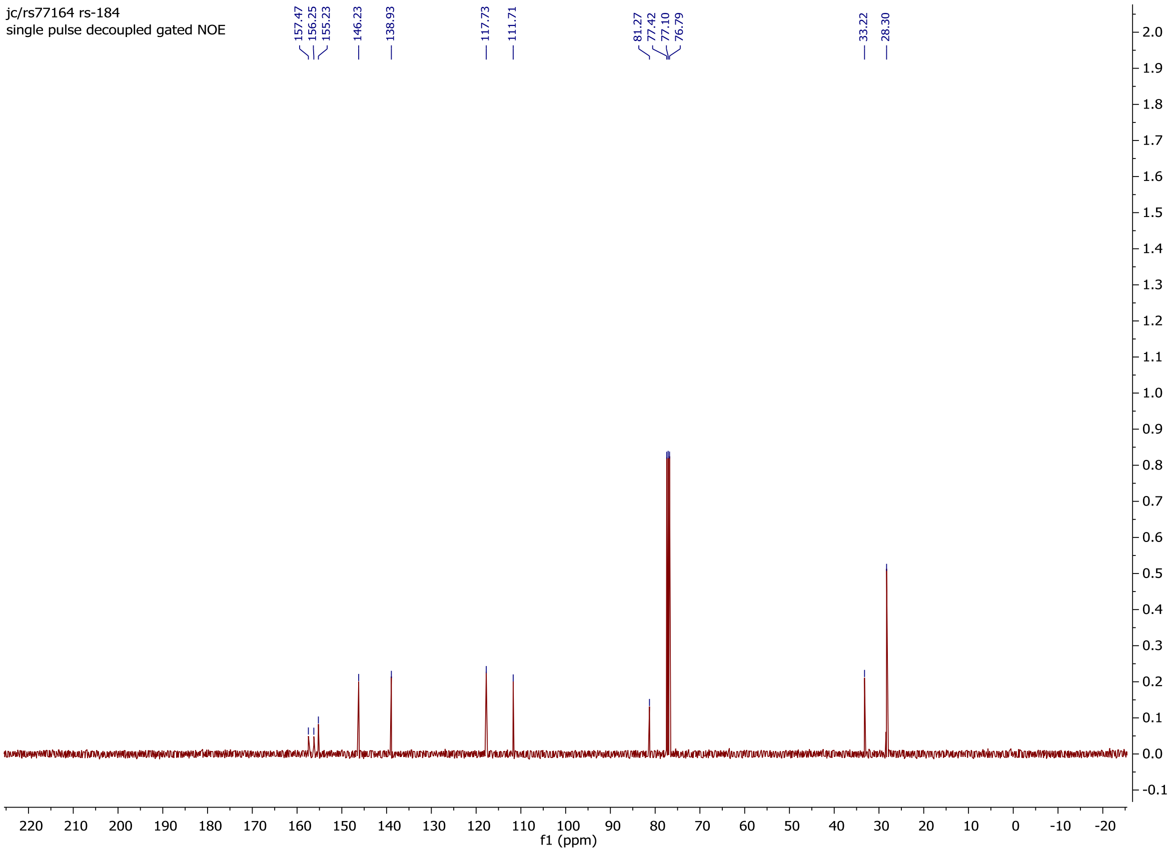

^{1}H NMR

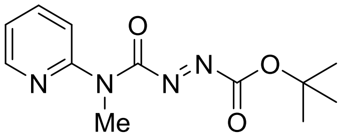

***tert*-Butyl (E)-2-(methyl(pyridin-2-yl)carbamoyl)diazene-1-carboxylate (5f).**

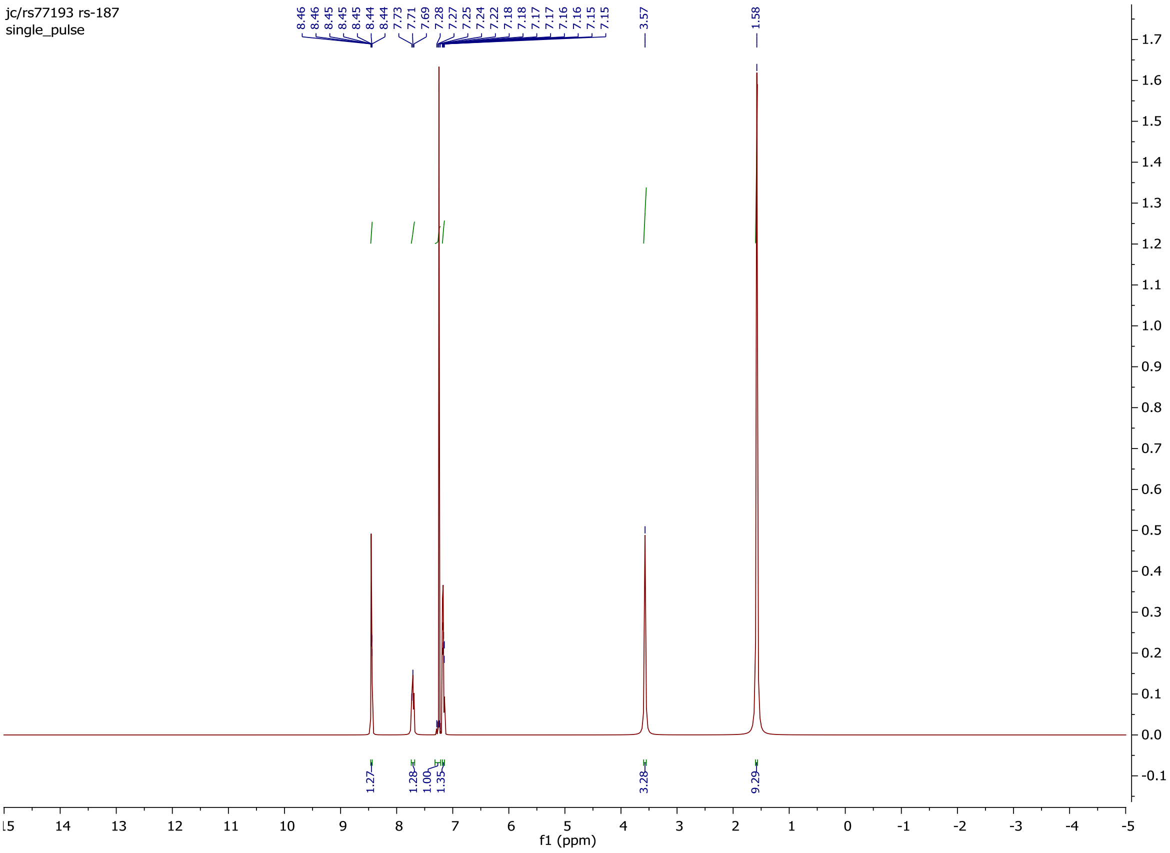

^{13}C NMR

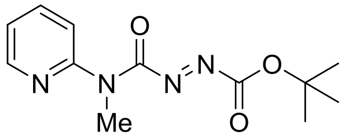

***tert*-Butyl (E)-2-(methyl(pyridin-2-yl)carbamoyl)diazene-1-carboxylate (5f).**

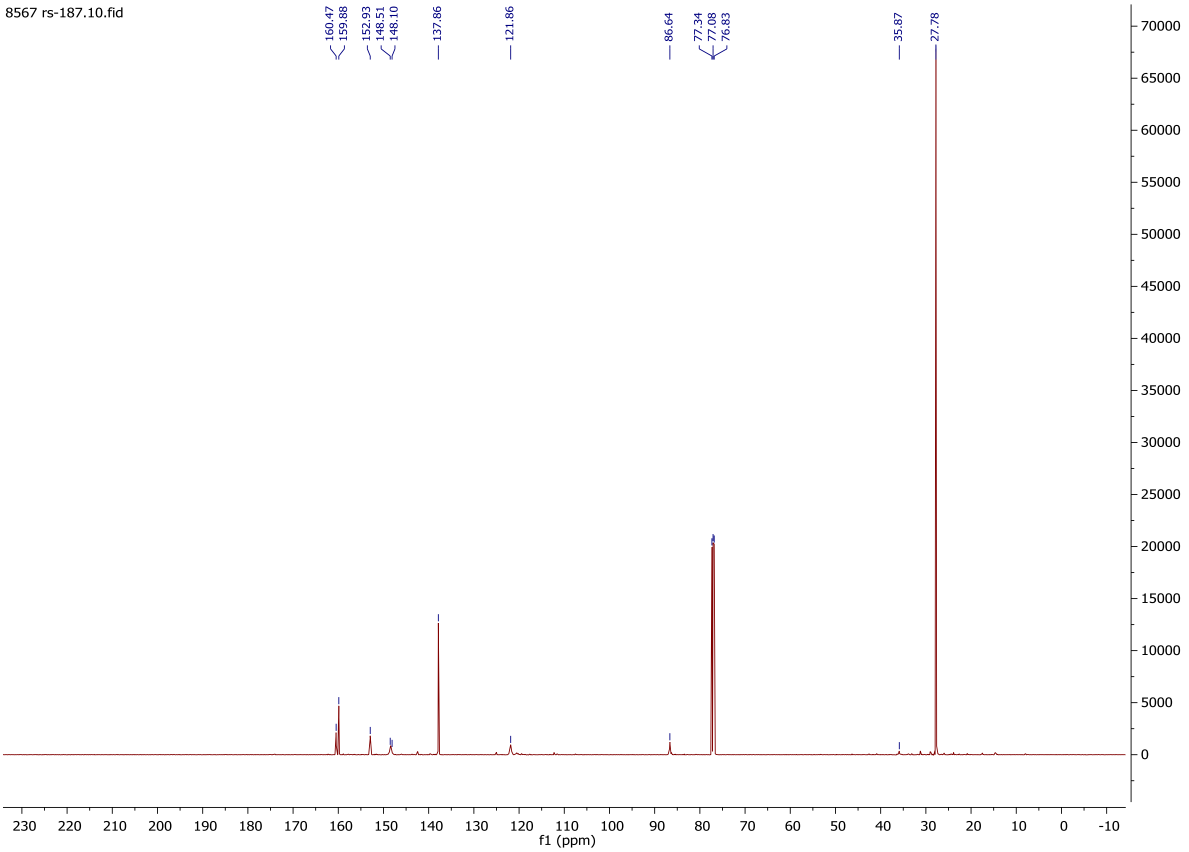

^1H NMR

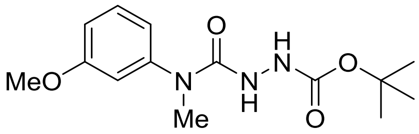

tert-Butyl 2-((3-methoxyphenyl)(methyl)carbamoyl)hydrazine-1-carboxylate (S5g)

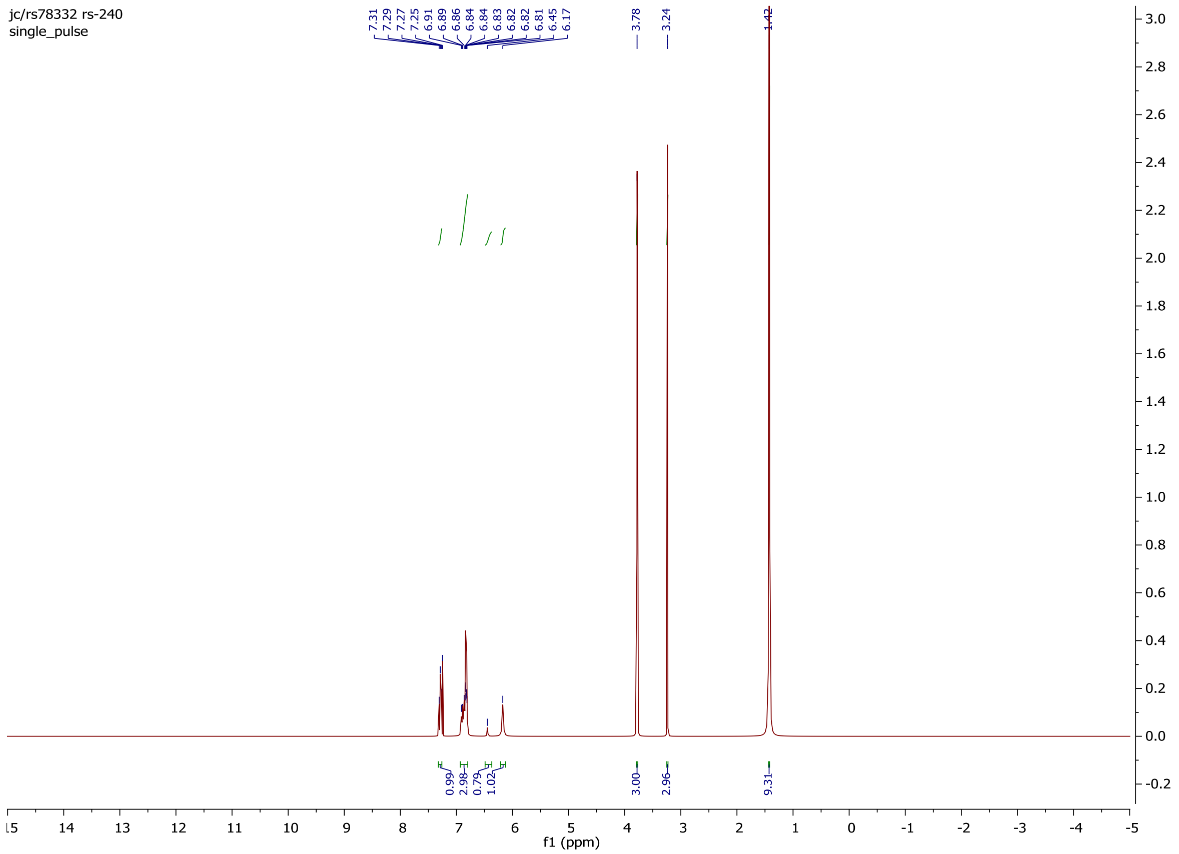

^{13}C NMR

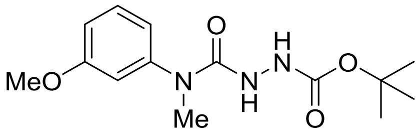

tert-Butyl 2-((3-methoxyphenyl)(methyl)carbamoyl)hydrazine-1-carboxylate (S5g)

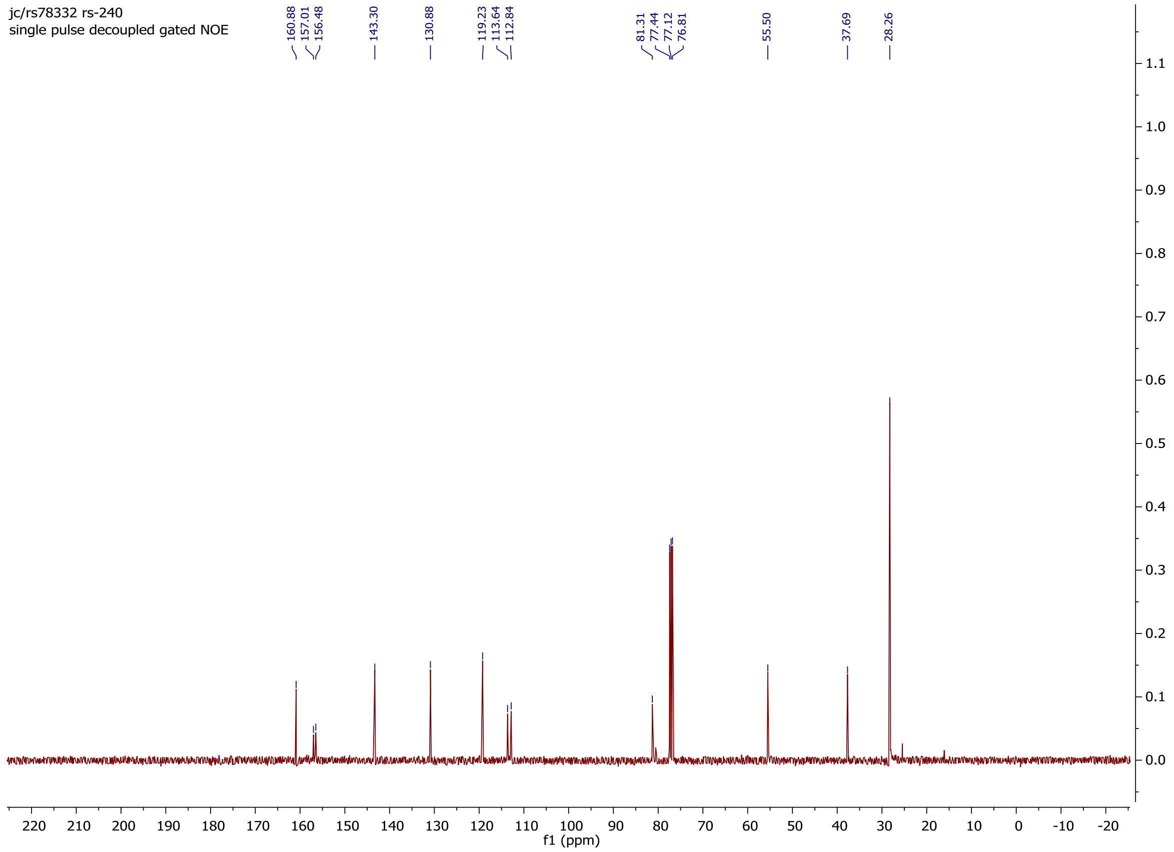

1H NMR

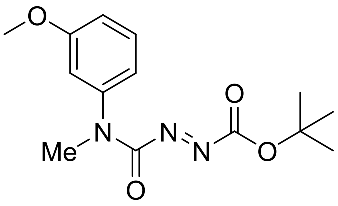

tert-Butyl (*E*)-2-((3-methoxyphenyl)(methyl)carbamoyl)diazene-1-carboxylate (5g).

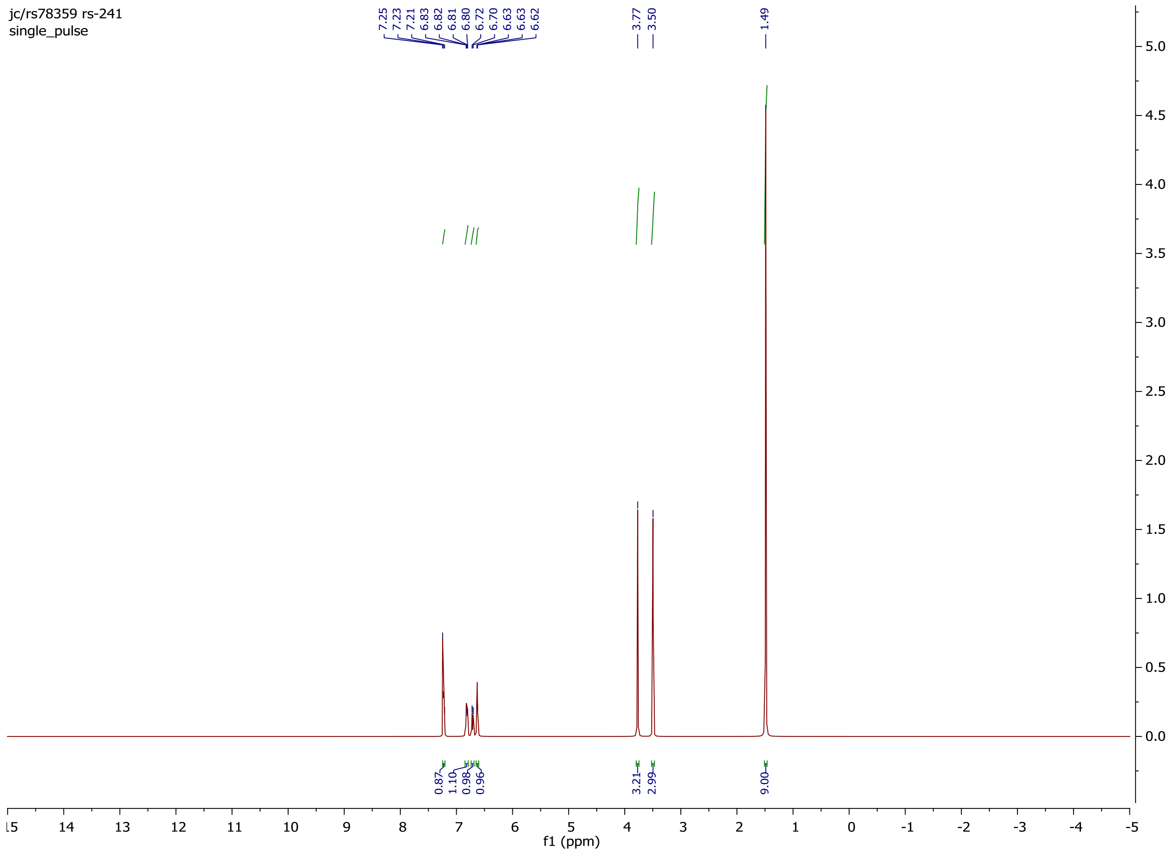

^{13}C NMR

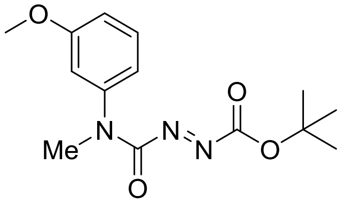

tert-Butyl (*E*)-2-((3-methoxyphenyl)(methyl)carbamoyl)diazene-1-carboxylate (5g).

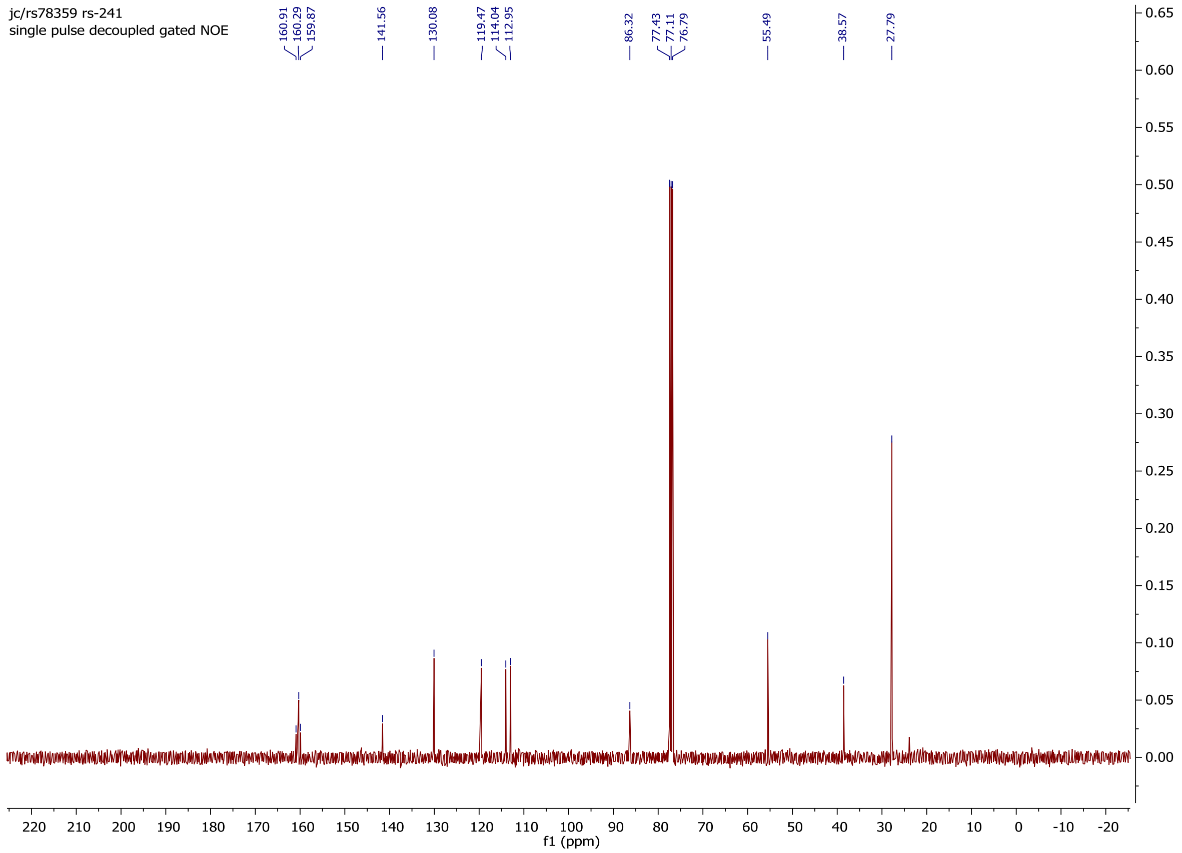

^{1}H NMR

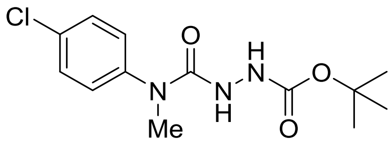

tert-Butyl 2-((4-chlorophenyl)(methyl)carbamoyl)hydrazine-1-carboxylate (S5h)

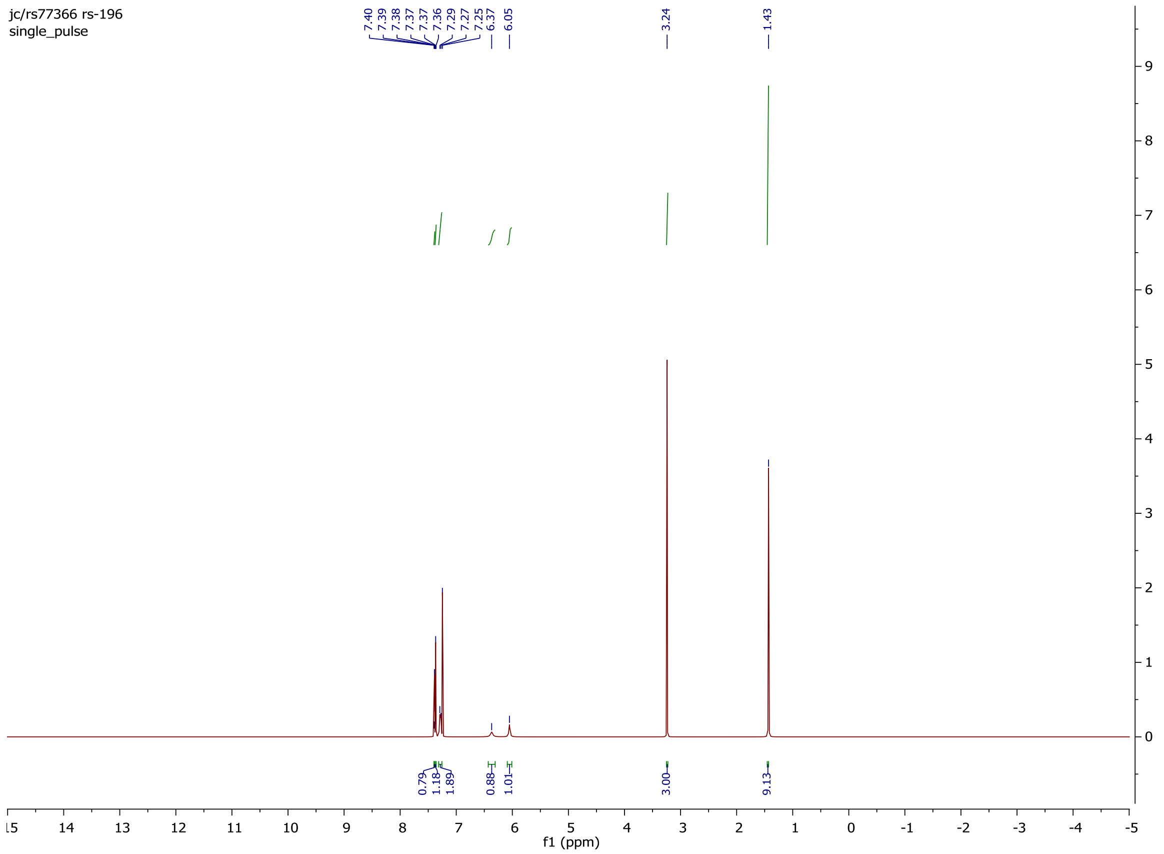

^{13}C NMR

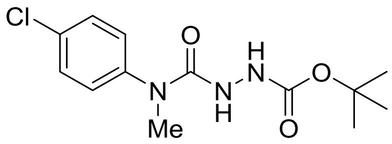

***tert*-Butyl 2-((4-chlorophenyl)(methyl)carbamoyl)hydrazine-1-carboxylate (S5h)**

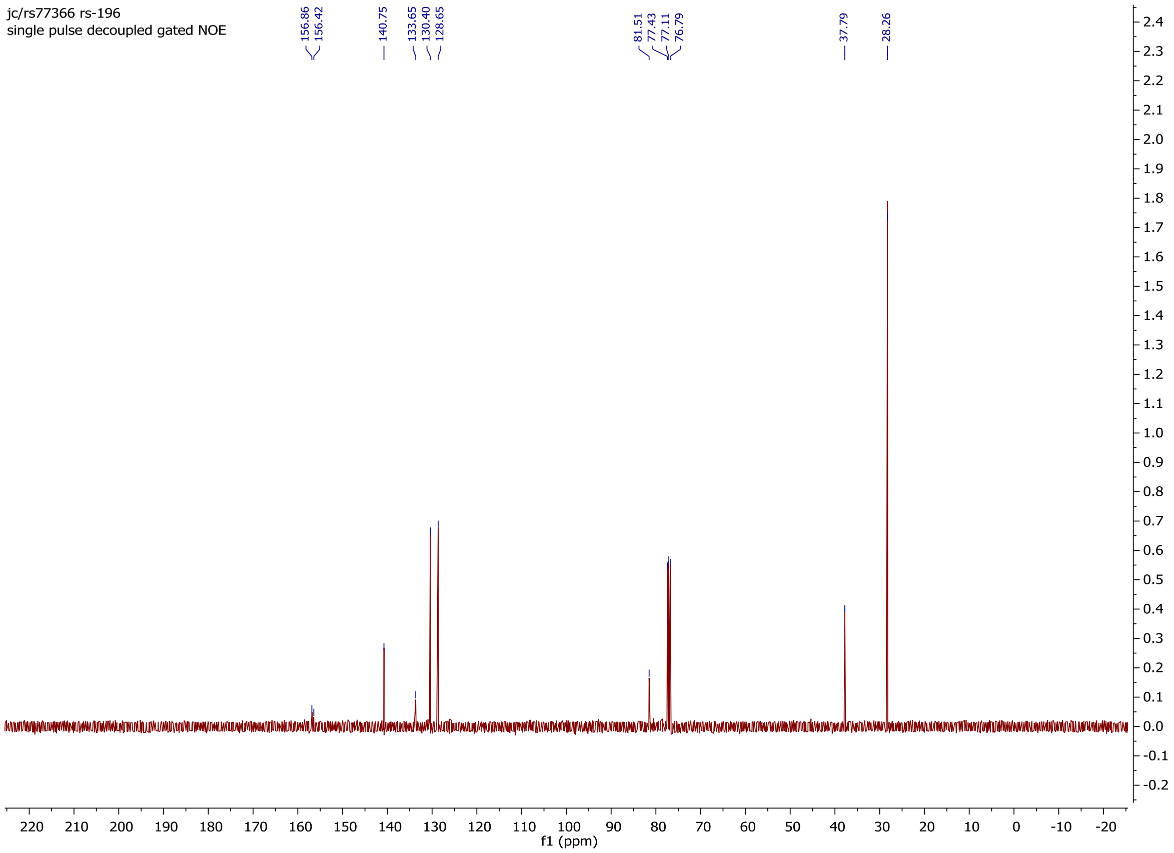

^{1}H NMR

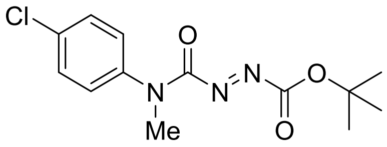

***tert*-Butyl (*E*)-2-((4-chlorophenyl)(methyl)carbamoyl)diazene-1-carboxylate (5h).**

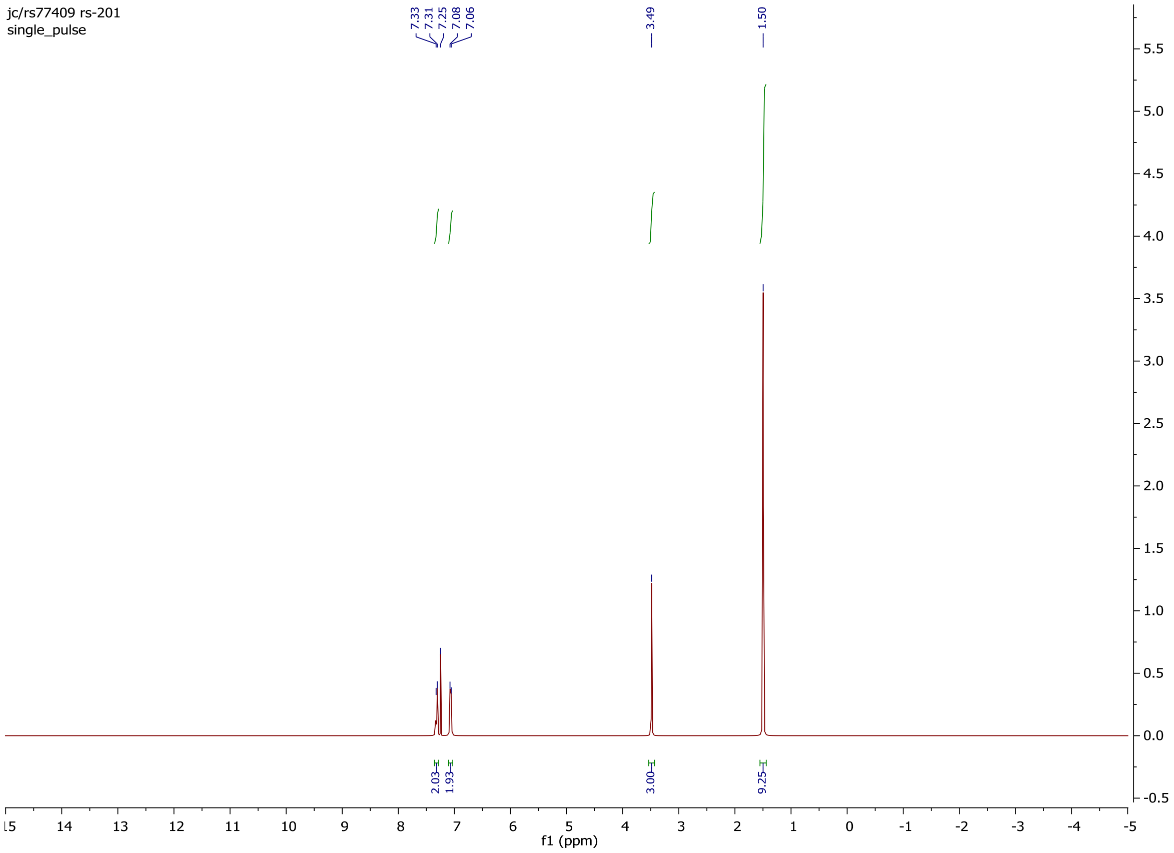

^{13}C NMR

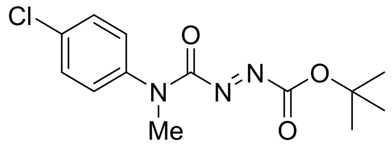

tert-Butyl (*E*)-2-((4-chlorophenyl)(methyl)carbamoyl)diazene-1-carboxylate (5h).

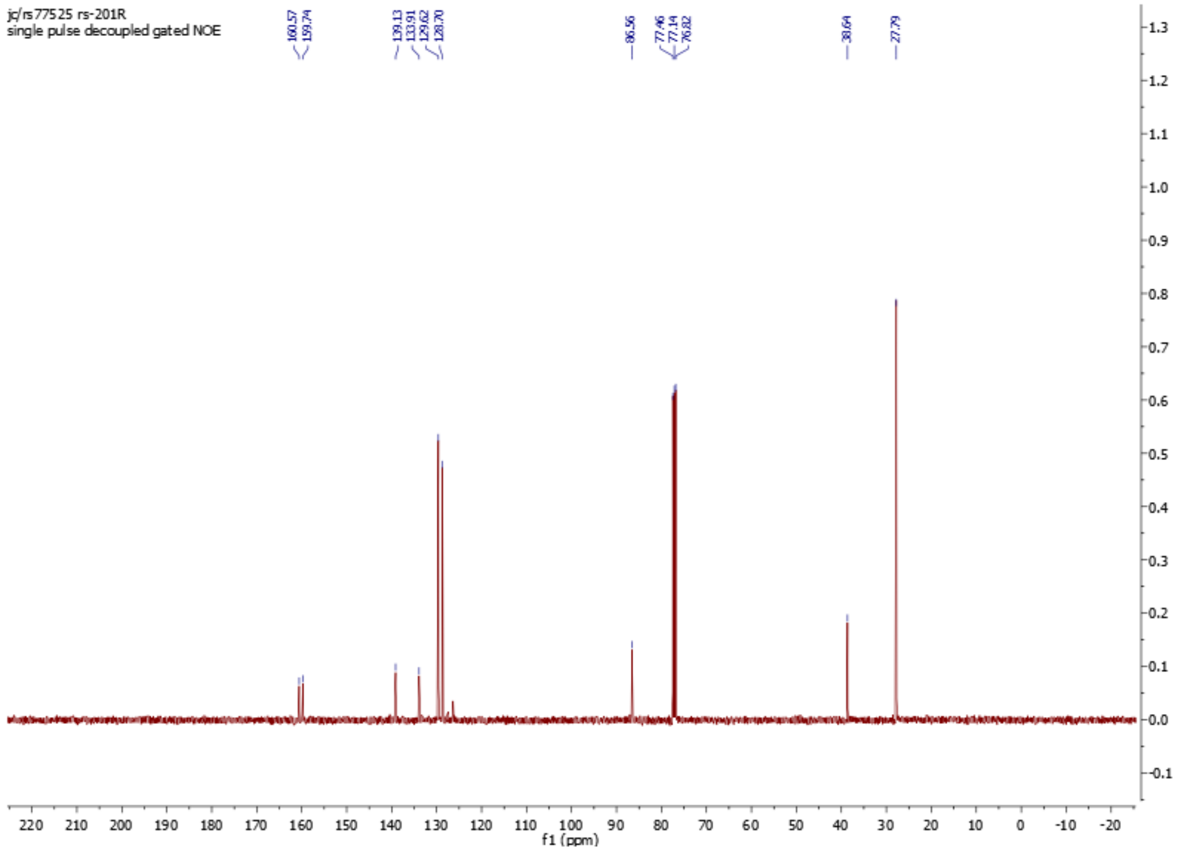

^{1}H NMR

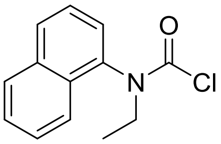

Methyl(naphthalen-1-yl)carbamic chloride SS5i

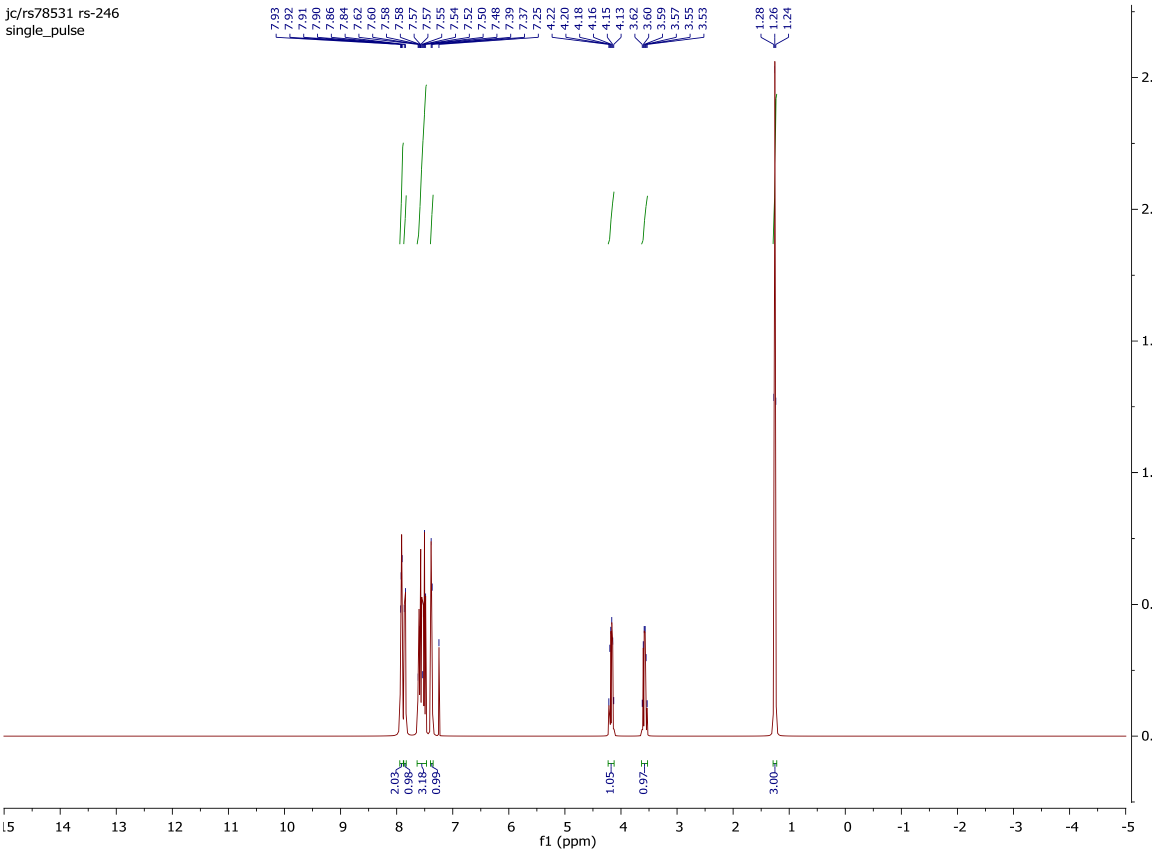

^{13}C NMR

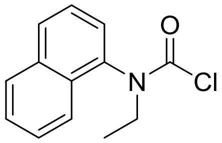

Methyl(naphthalen-1-yl)carbamic chloride (SS5i)

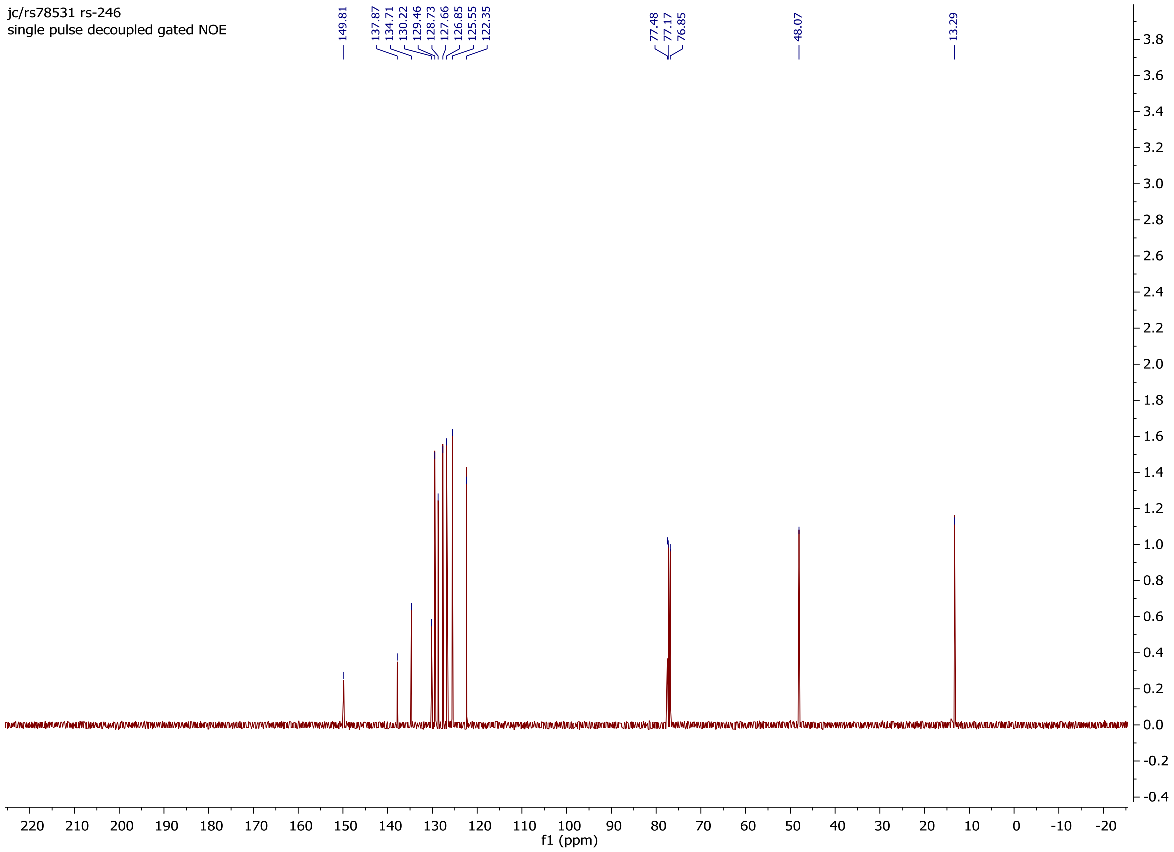

^{1}H NMR

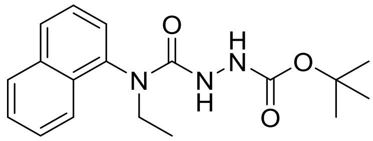

tert-Butyl 2-(ethyl(naphthalen-1-yl)carbamoyl)hydrazine-1-carboxylate (S5i)

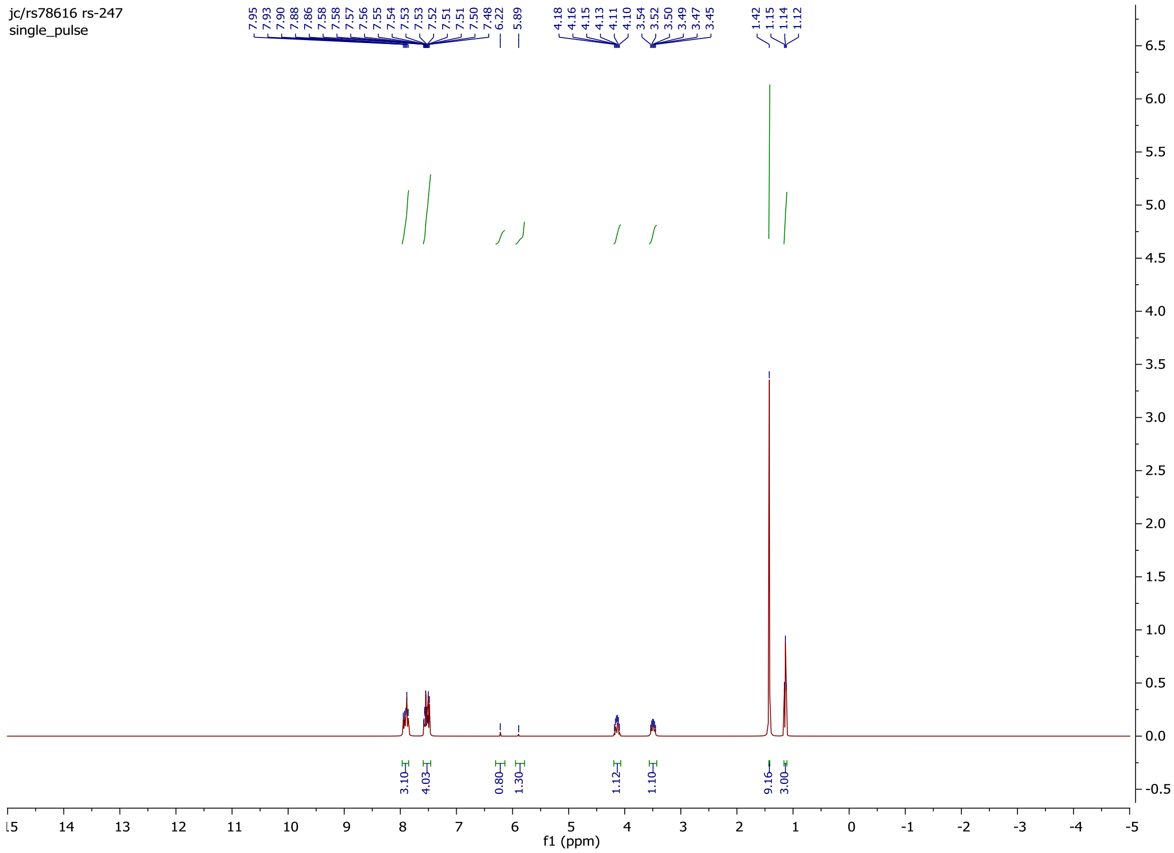

^{13}C NMR

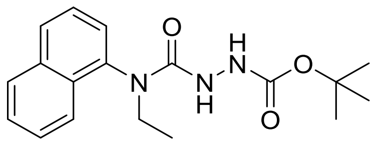

***tert*-Butyl 2-(ethyl(naphthalen-1-yl)carbamoyl)hydrazine-1-carboxylate (S5i)**

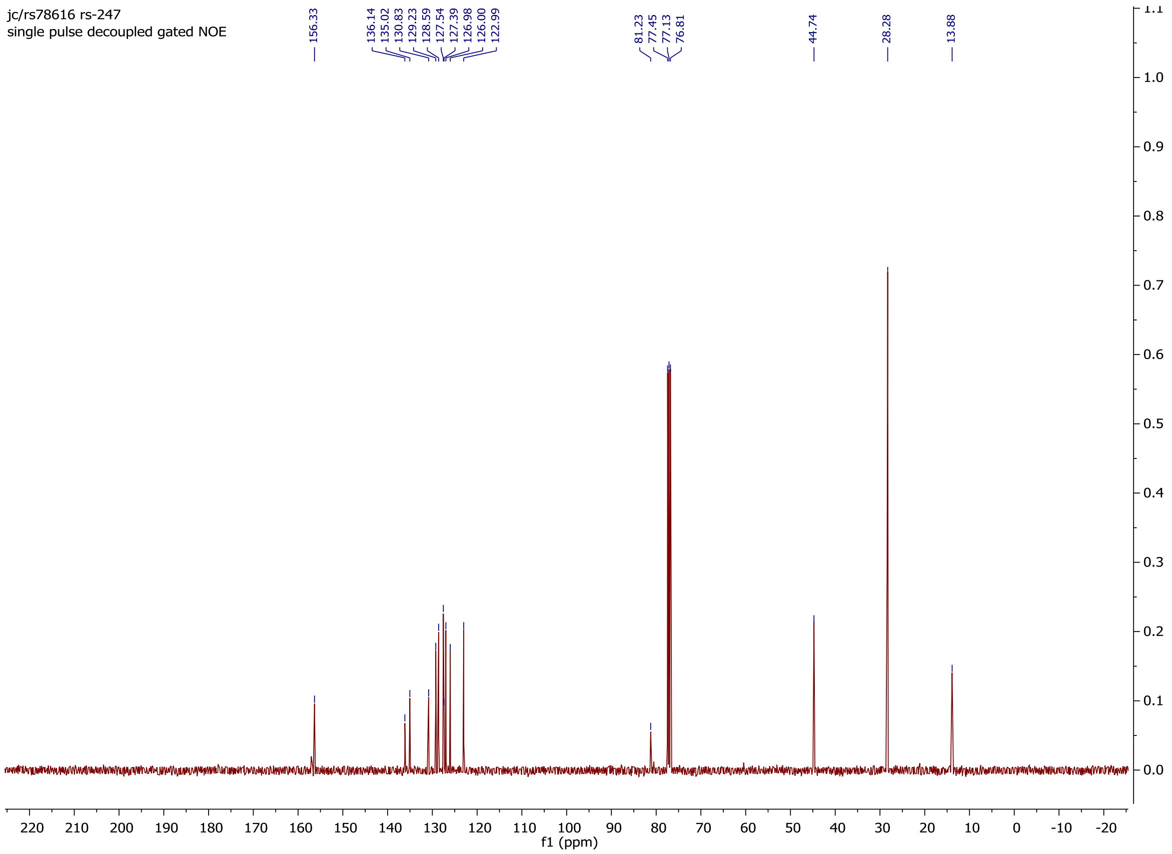

^{1}H NMR

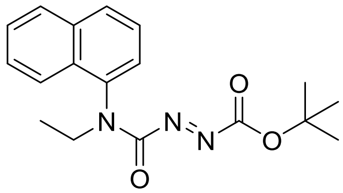

***tert*-Butyl (E)-2-(ethyl(naphthalen-1-yl)carbamoyl)diazene-1-carboxylate (5i).**

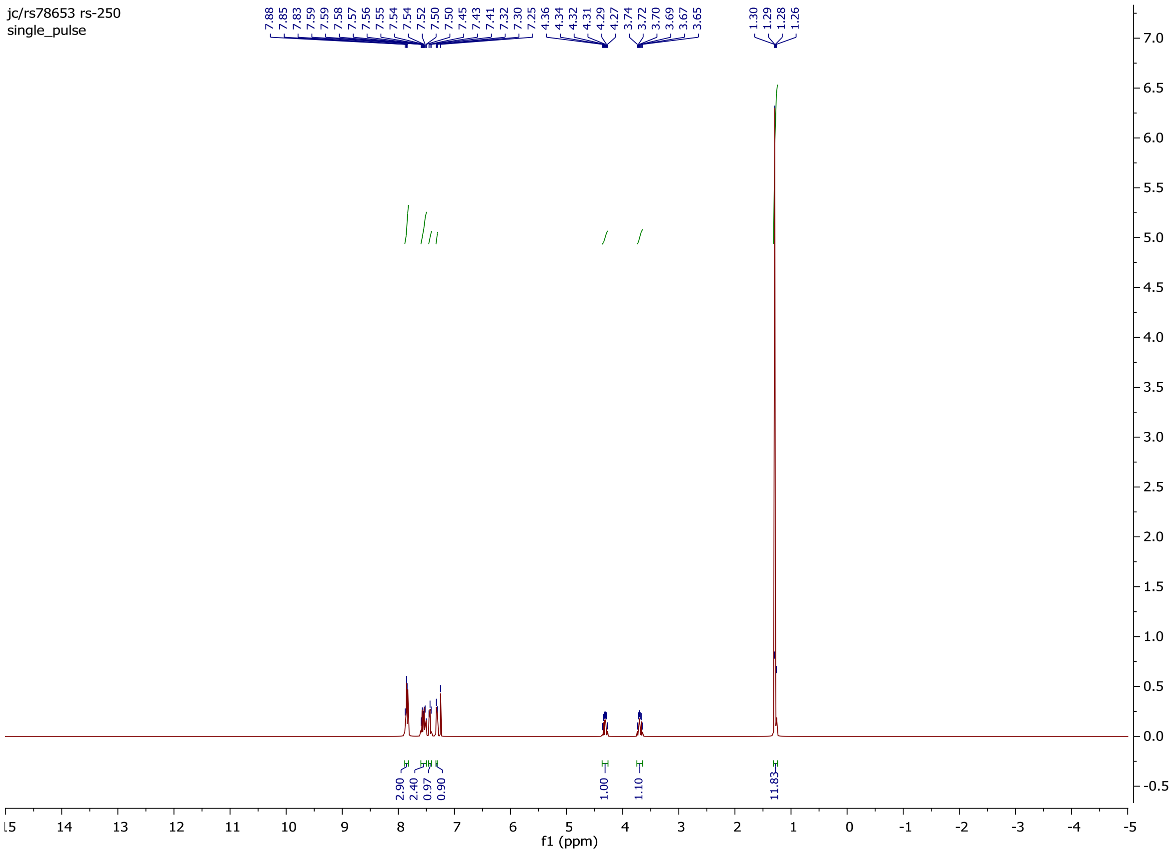

^{13}C NMR

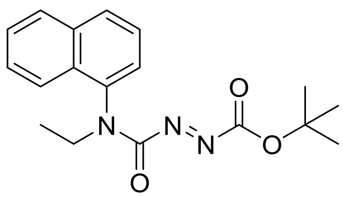

tert-Butyl (E)-2-(ethyl(naphthalen-1-yl)carbamoyl)diazene-1-carboxylate (5i).

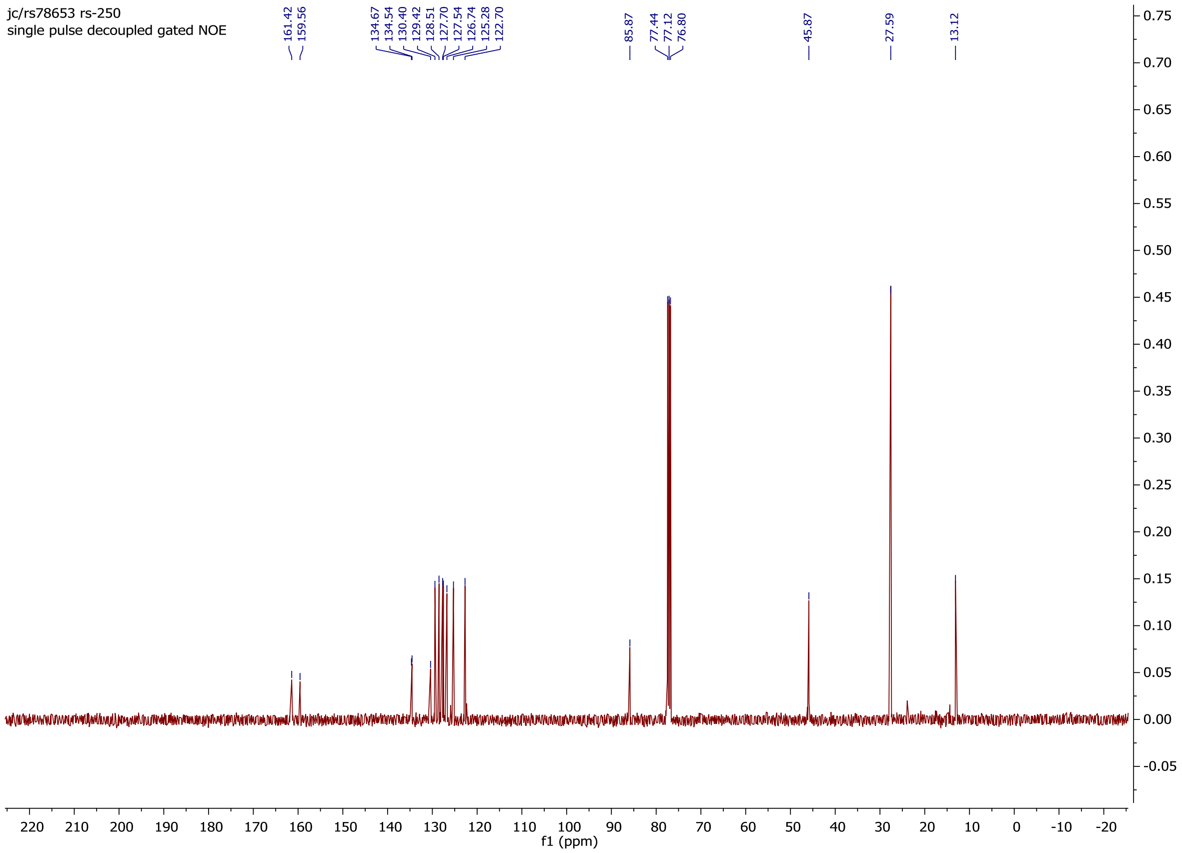

^{1}H NMR

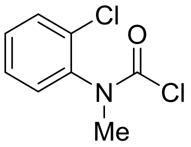

(2-Chlorophenyl)(methyl)carbamic chloride (SS5j)

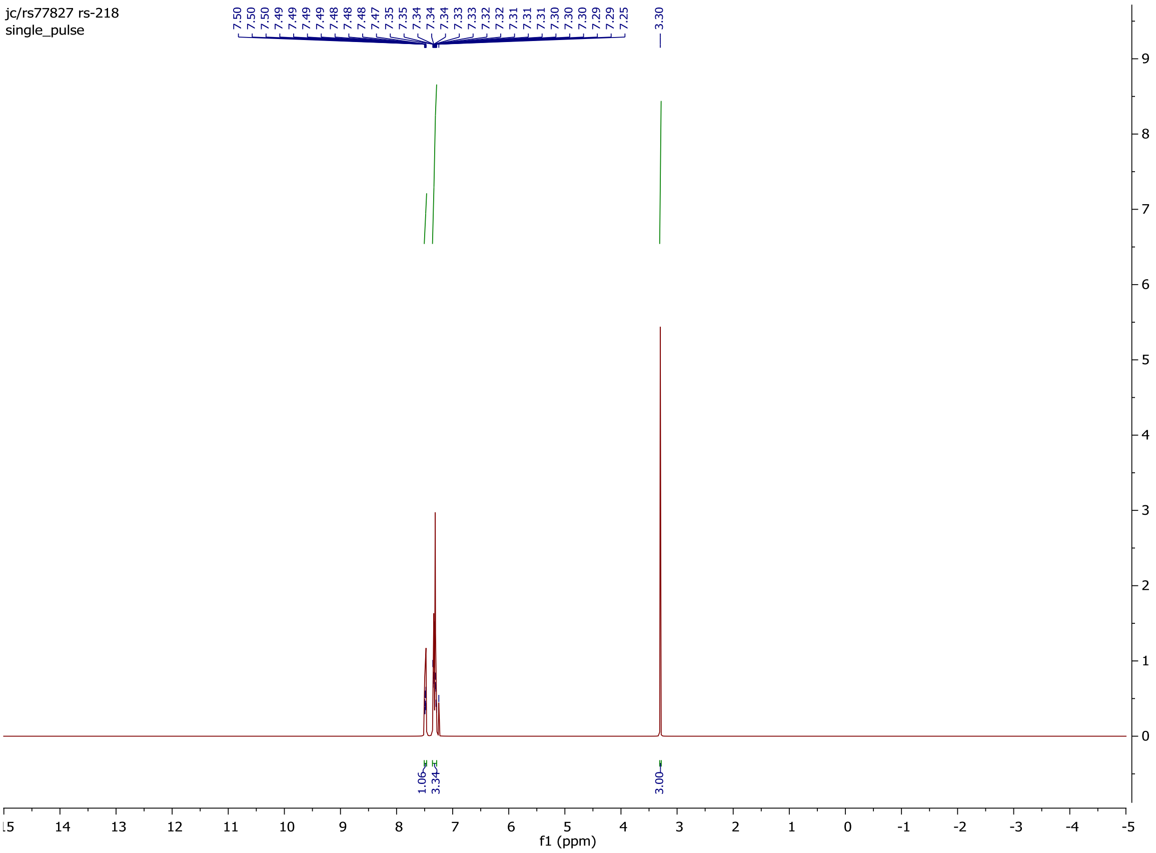

^{13}C NMR

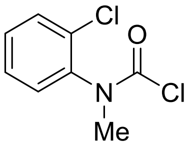

(2-Chlorophenyl)(methyl)carbamic chloride (SS5j)

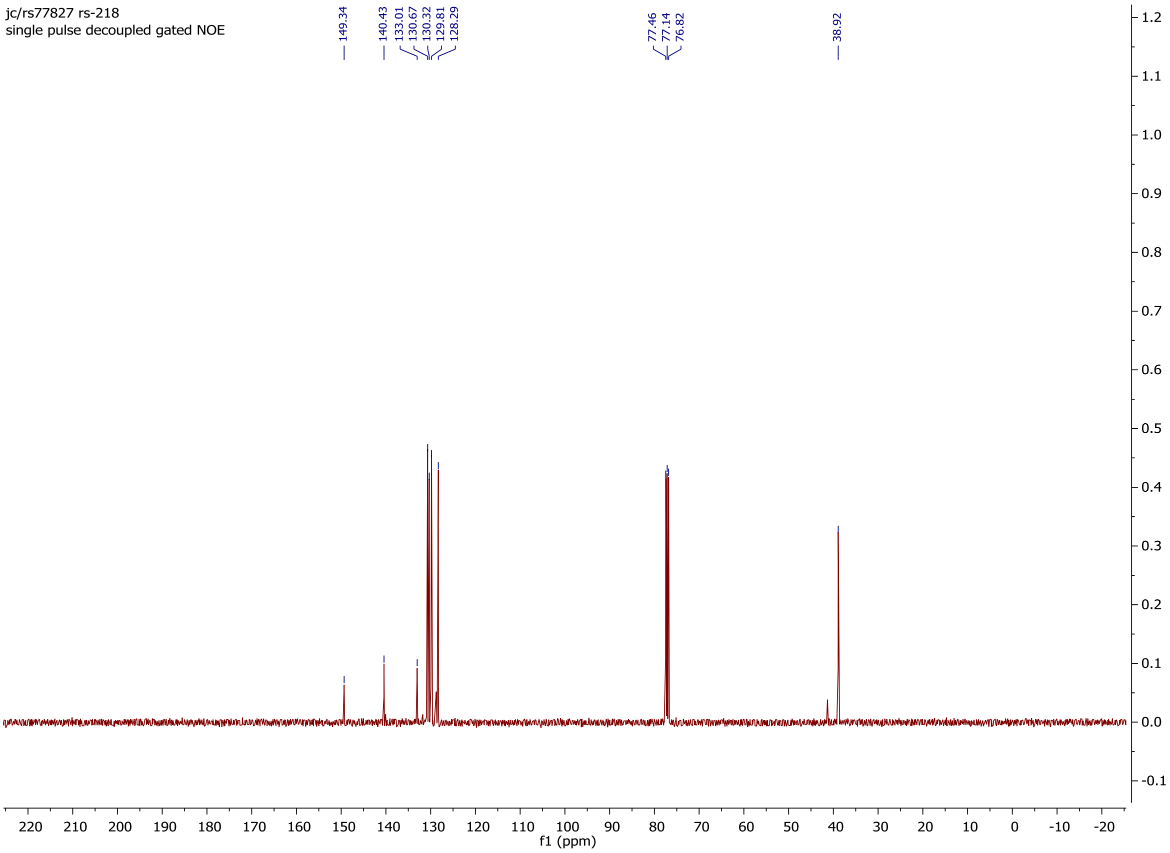

^{1}H NMR

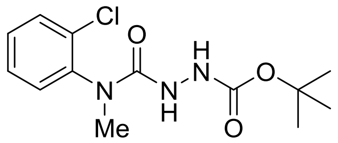

***tert*-Butyl 2-((2-chlorophenyl)(methyl)carbamoyl)hydrazine-1-carboxylate (S5j)**

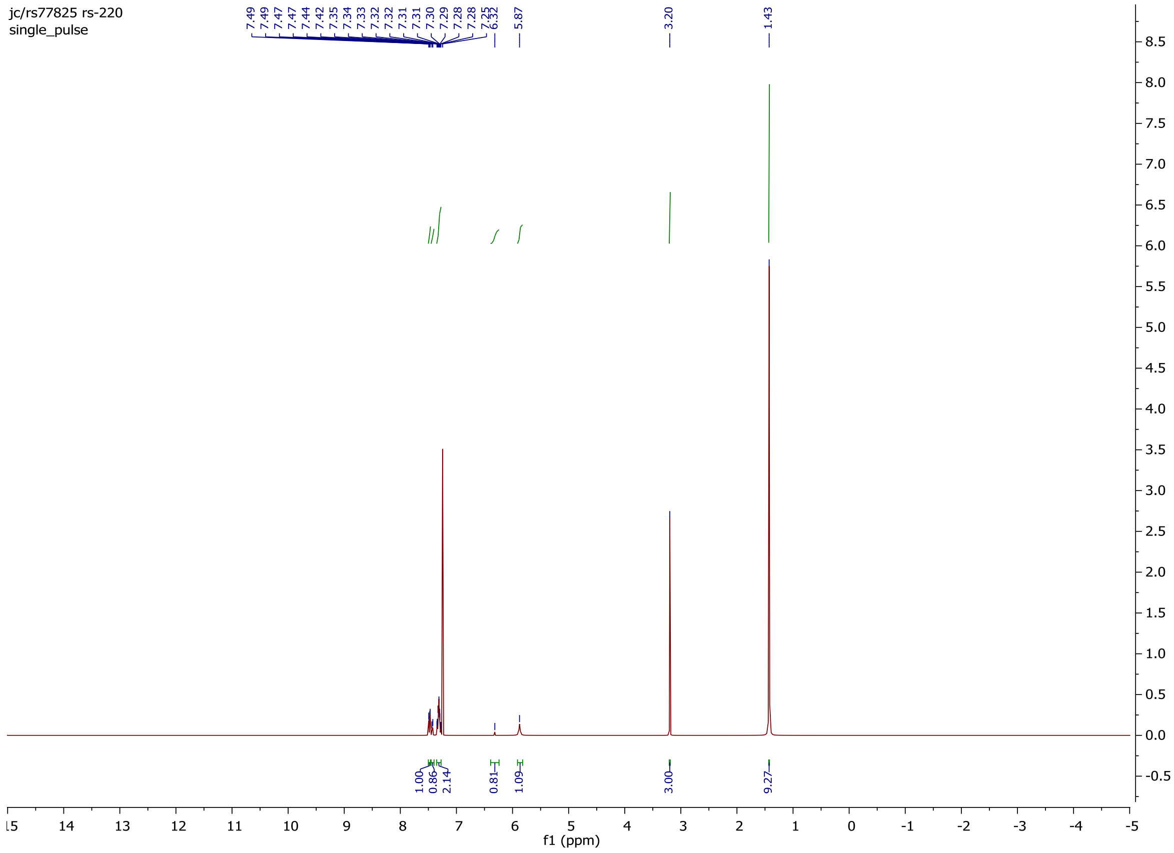

^{13}C NMR

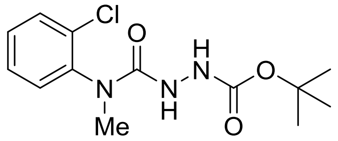

tert-Butyl 2-((2-chlorophenyl)(methyl)carbamoyl)hydrazine-1-carboxylate (S5j)

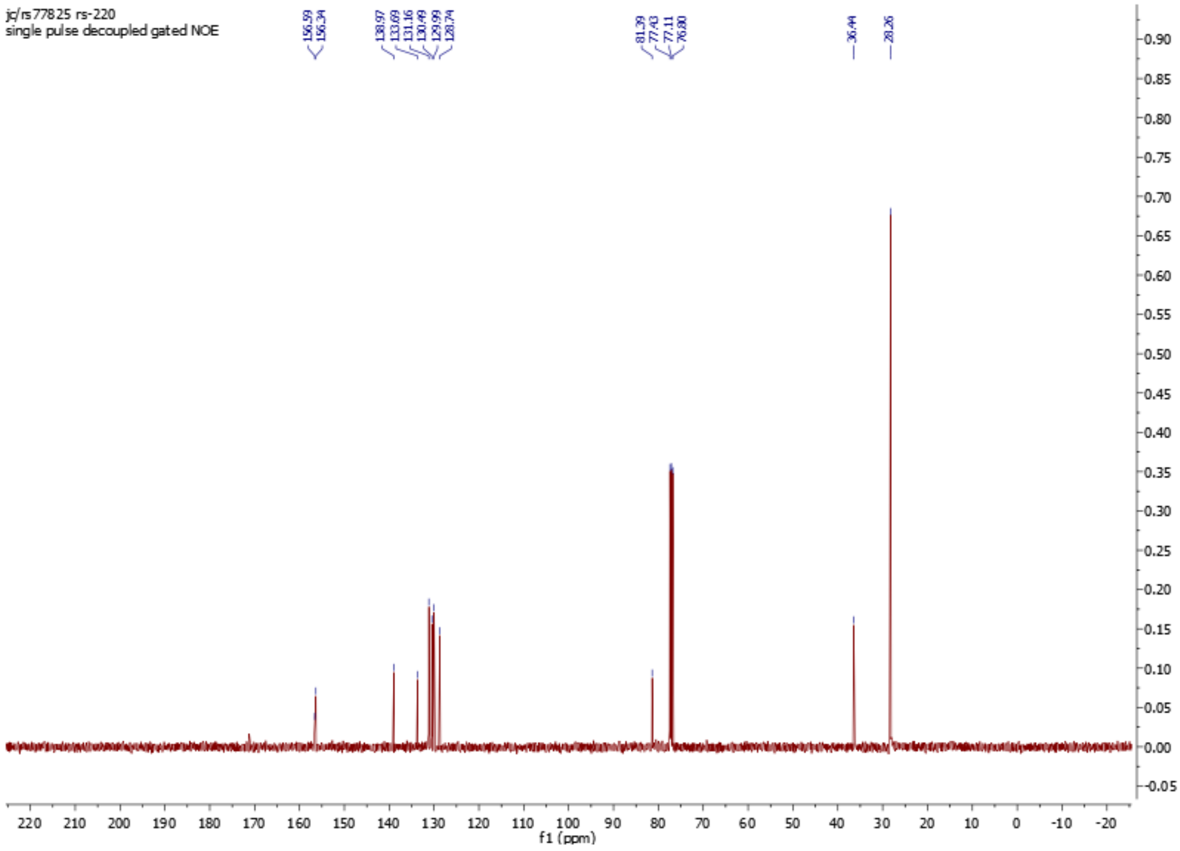

^{1}H NMR

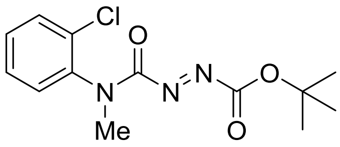

***tert*-Butyl (E)-2-((2-chlorophenyl)(methyl)carbamoyl)diazene-1-carboxylate (5j).**

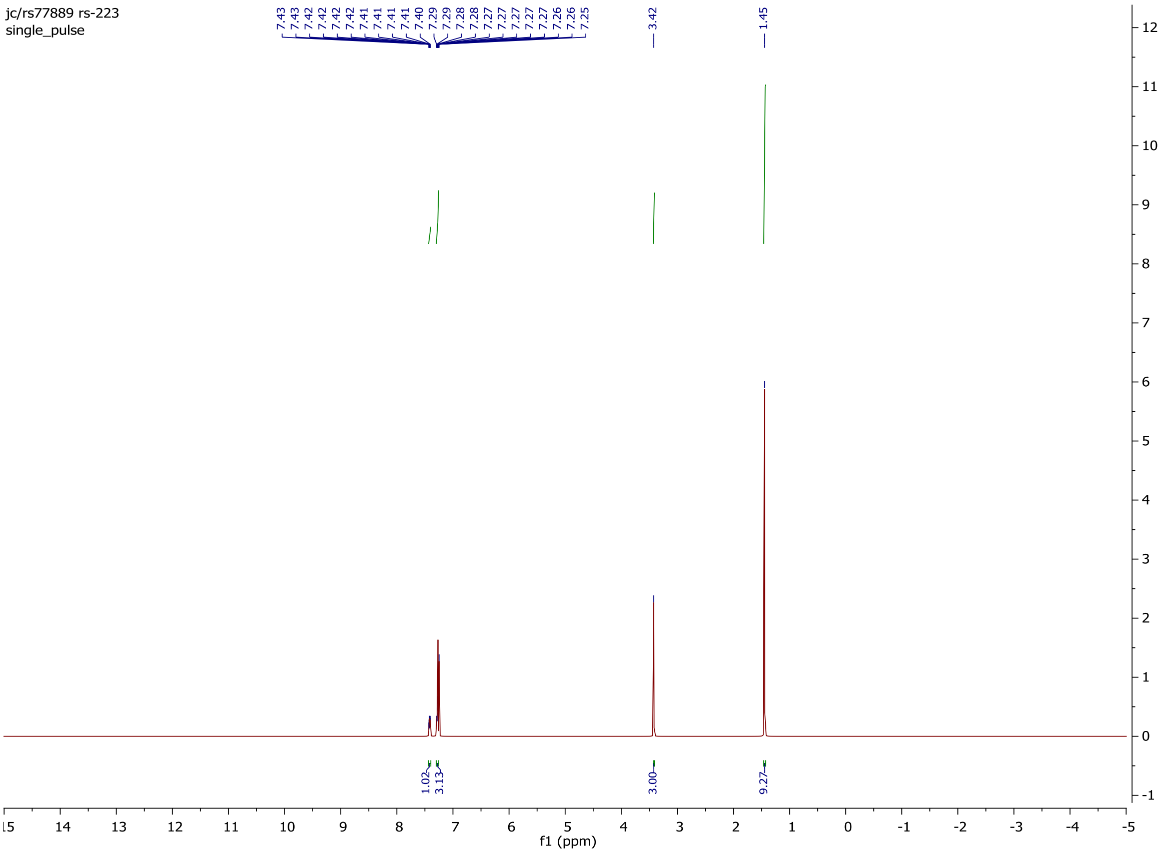

^{13}C NMR

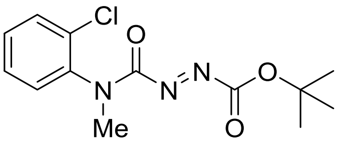

tert-Butyl (E)-2-((2-chlorophenyl)(methyl)carbamoyl)diazene-1-carboxylate (5j).

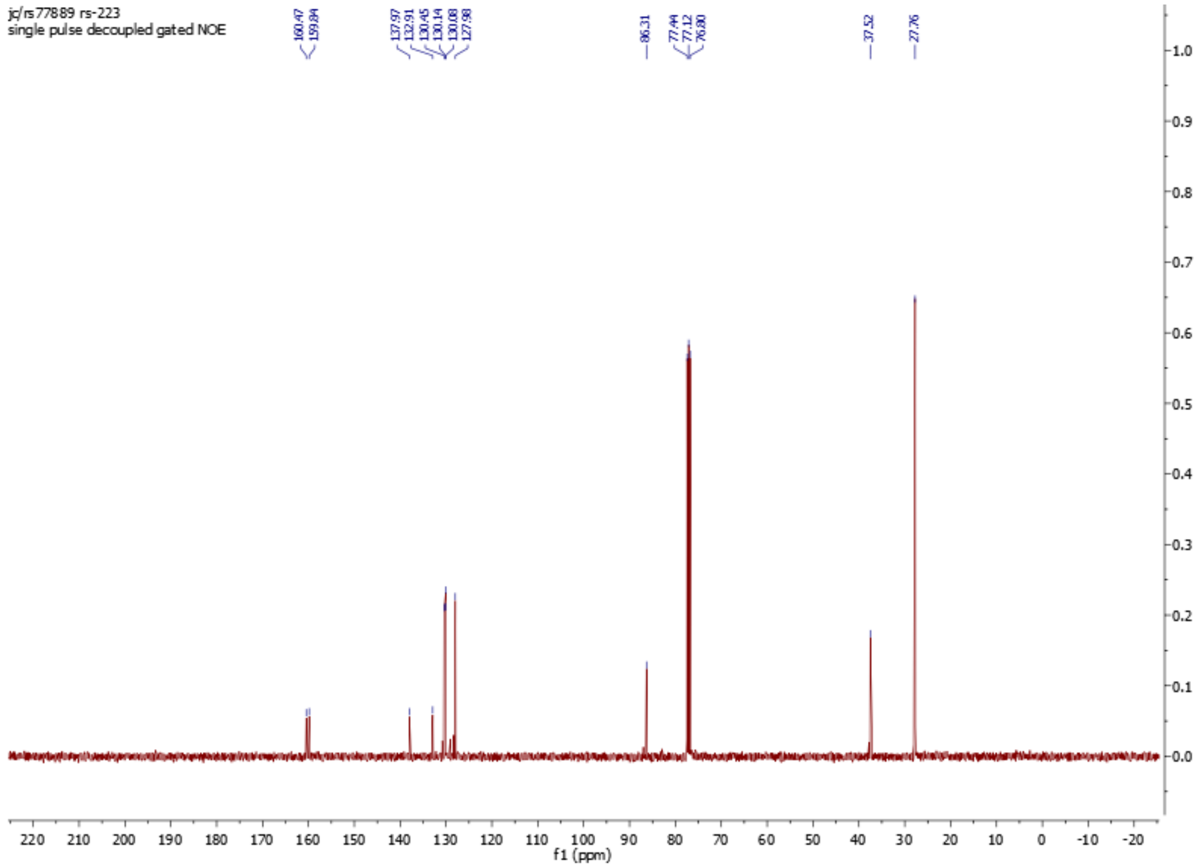

^{1}H NMR

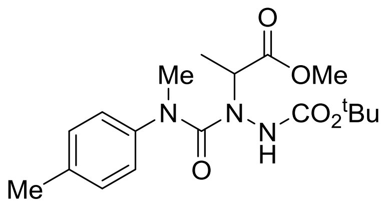

tert-Butyl 2-(1-methoxy-1-oxopropan-2-yl)-2-(methyl(*p*-tolyl)carbamoyl)hydrazinecarboxylate (6)

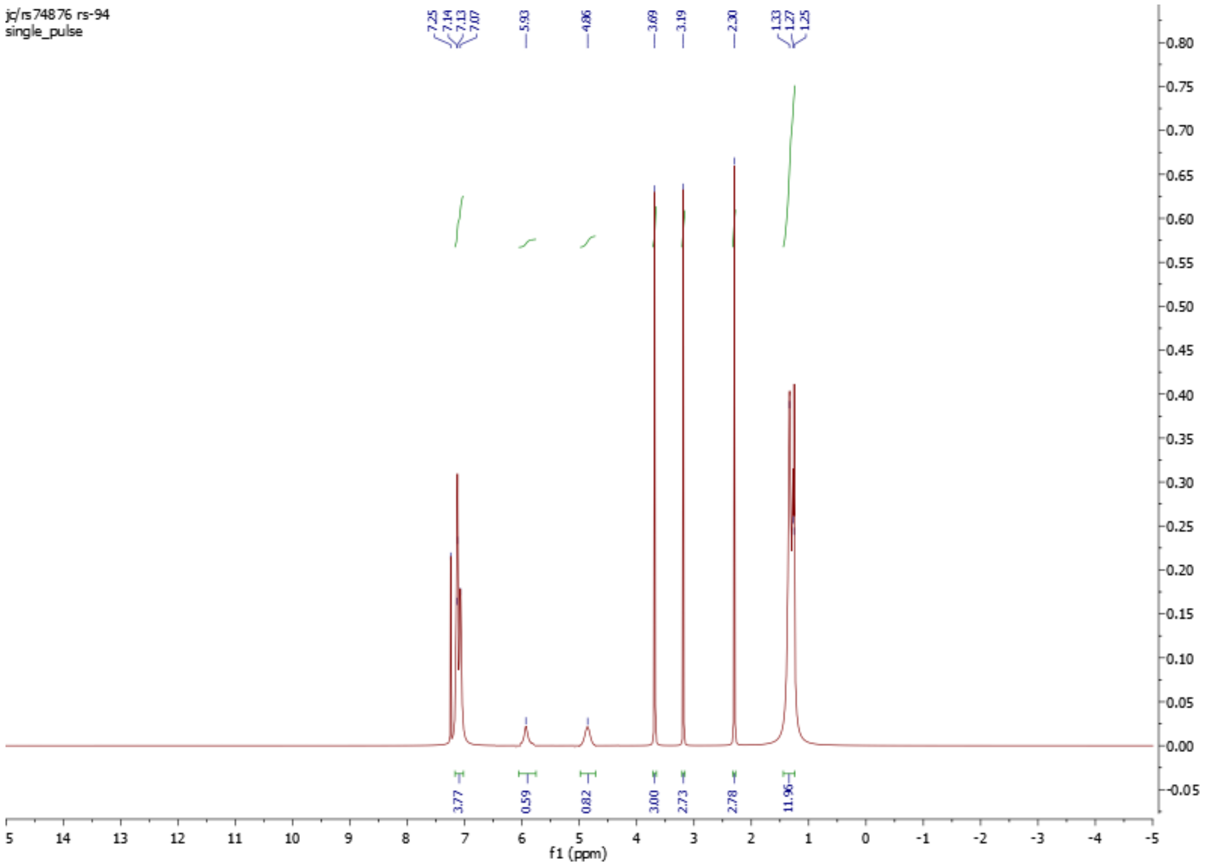

^{13}C NMR

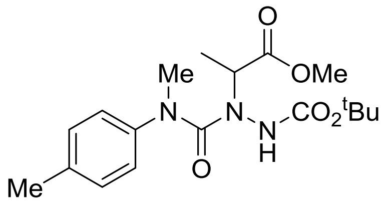

tert-Butyl 2-(1-methoxy-1-oxopropan-2-yl)-2-(methyl(*p*-tolyl)carbamoyl)hydrazinecarboxylate (6)

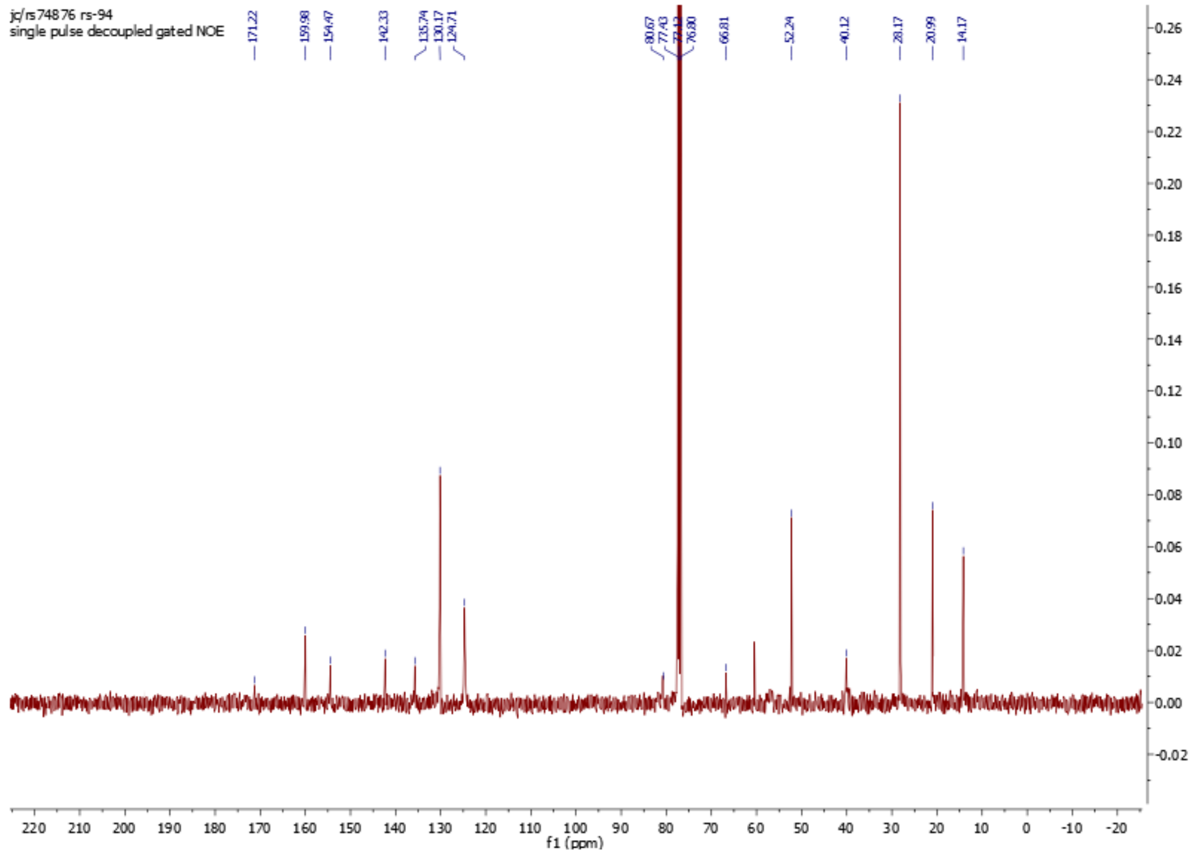

^{1}H NMR

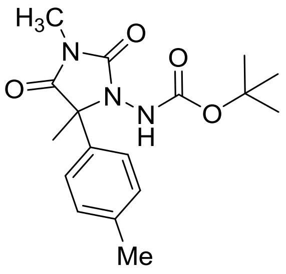

tert-Butyl (3,5-dimethyl-2,4-dioxo-5-(p-tolyl)imidazolidin-1-yl)carbamate (7a)

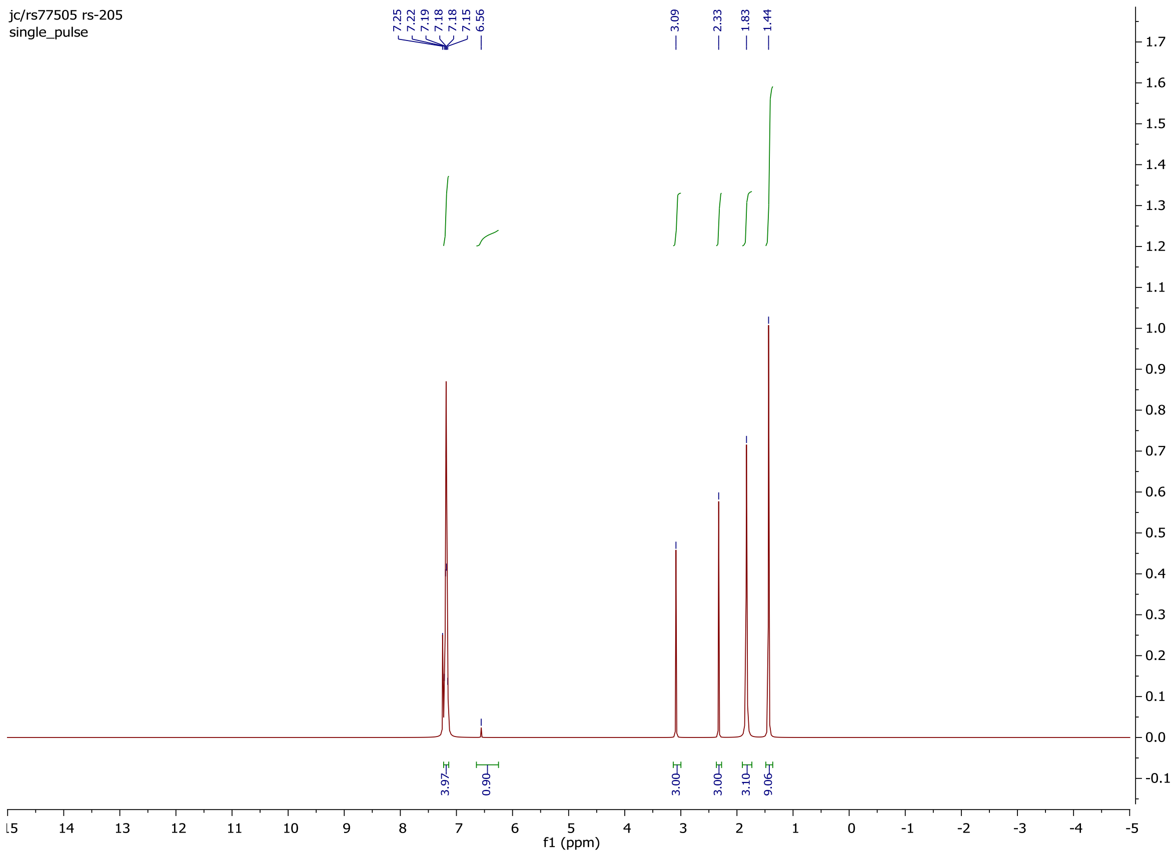

^{13}C NMR

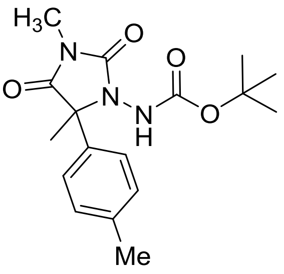

***tert*-Butyl (3,5-dimethyl-2,4-dioxo-5-(p-tolyl)imidazolidin-1-yl)carbamate (7a)**

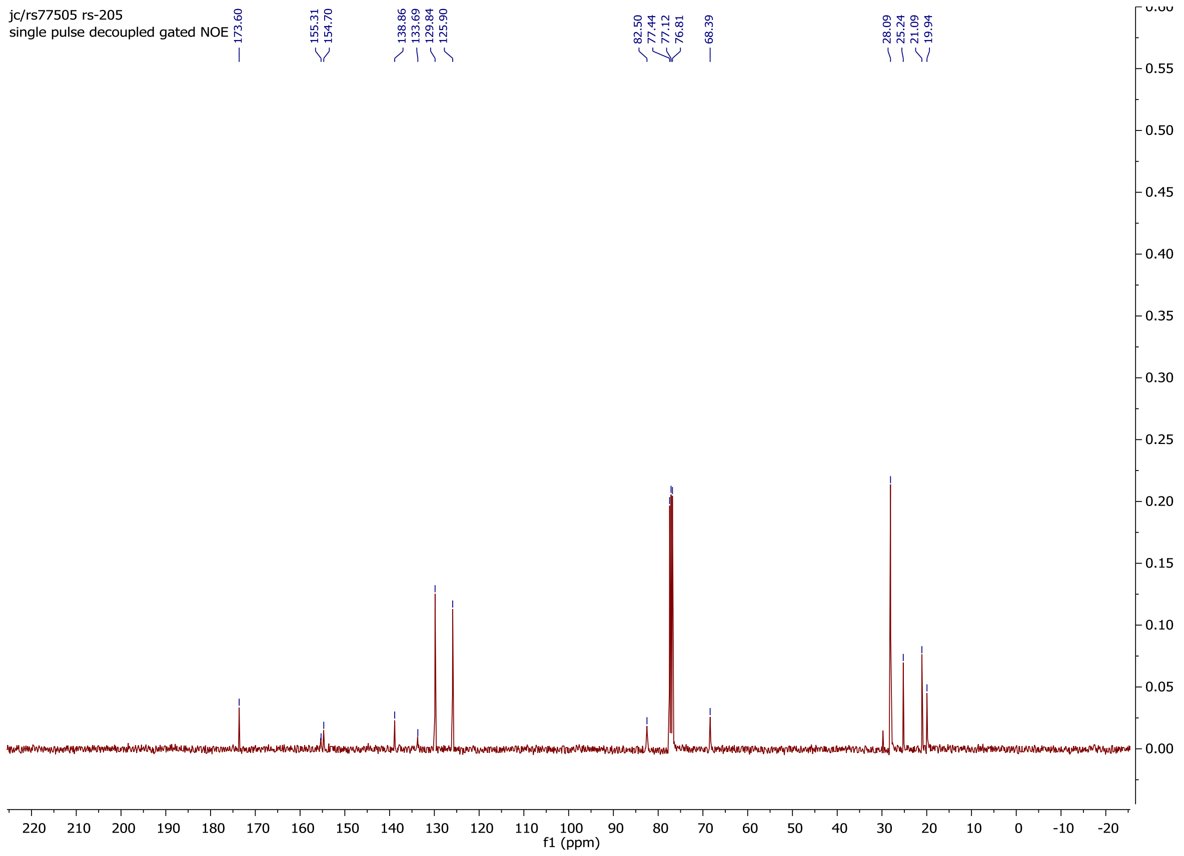

^{1}H NMR

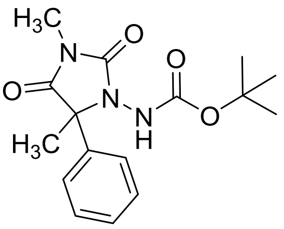

***tert*-Butyl (3,5-dimethyl-2,4-dioxo-5-phenylimidazolidin-1-yl)carbamate (7b)**

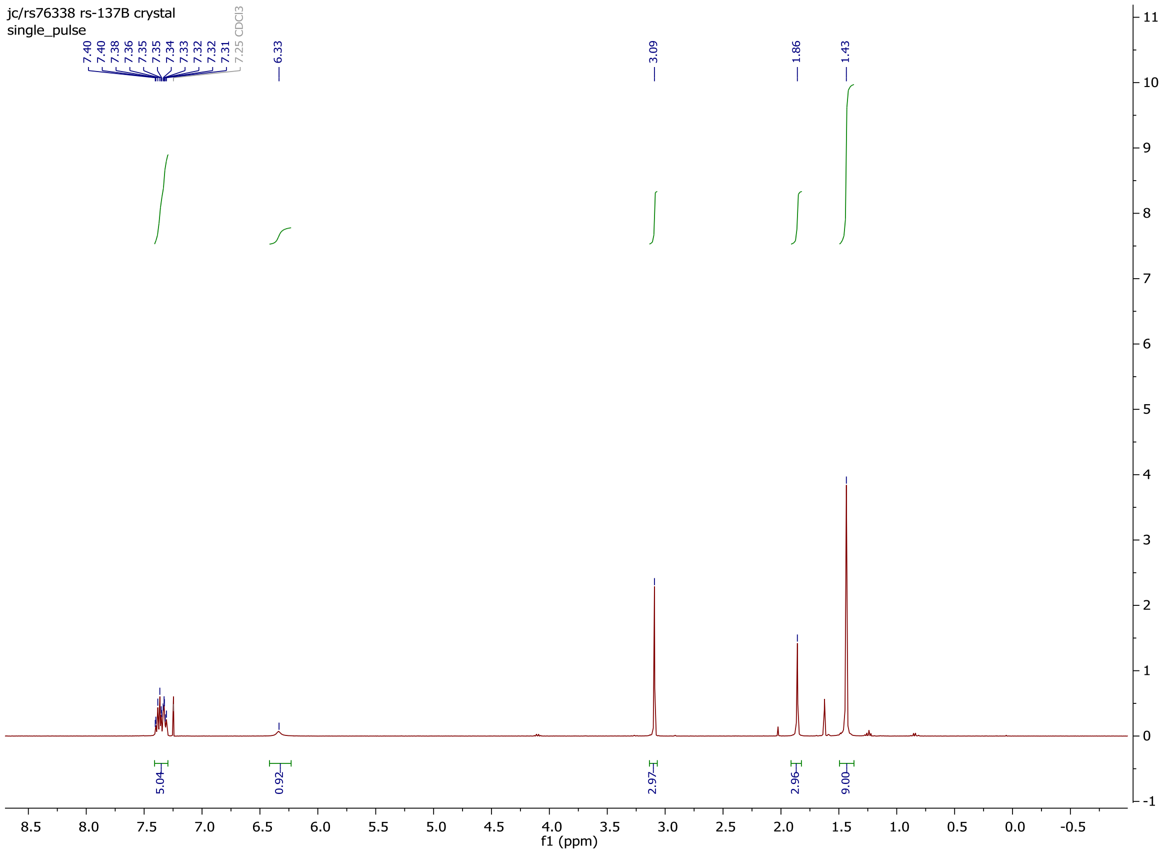

^{13}C NMR

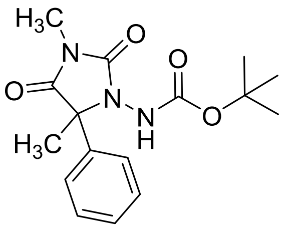

***tert*-Butyl (3,5-dimethyl-2,4-dioxo-5-phenylimidazolidin-1-yl)carbamate (7b)**

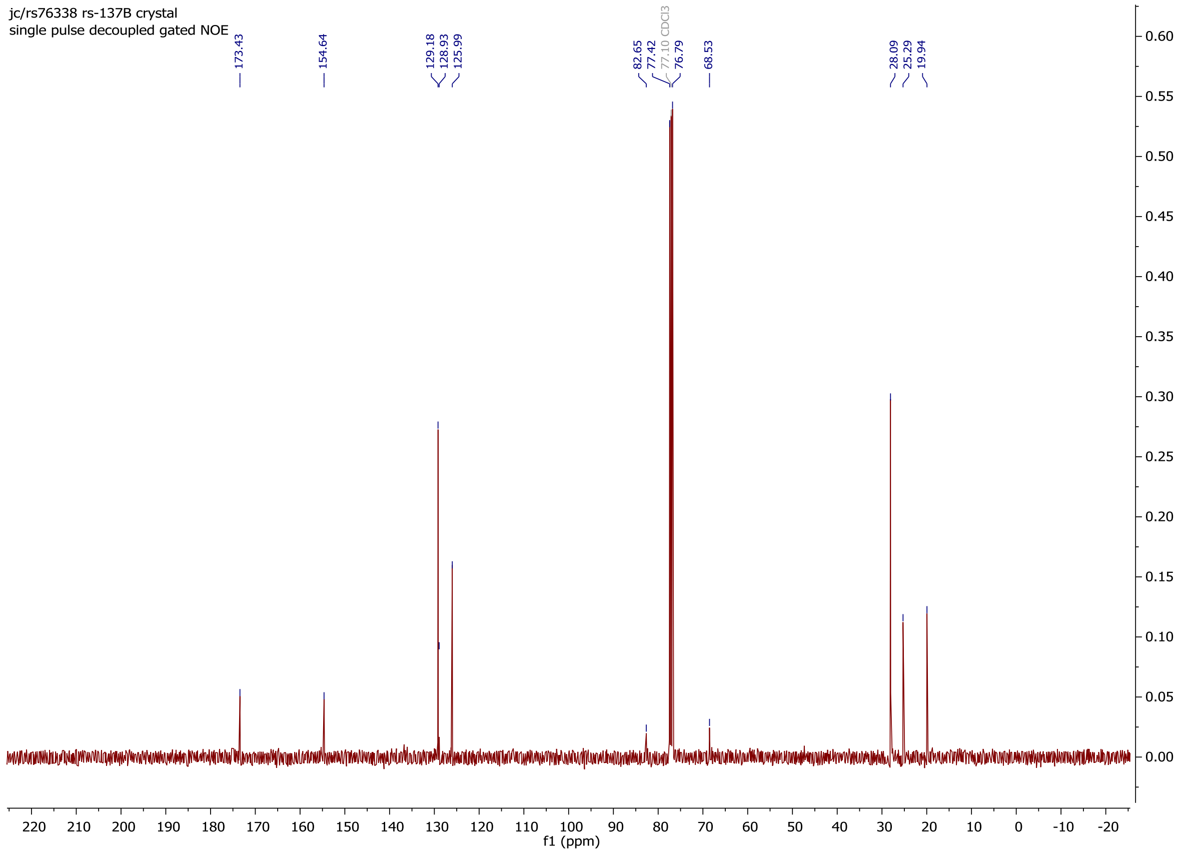

^{1}H NMR

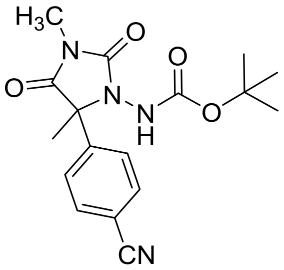

tert-Butyl (5-(4-cyanophenyl)-3,5-dimethyl-2,4-dioxoimidazolidin-1-yl)carbamate (7c)

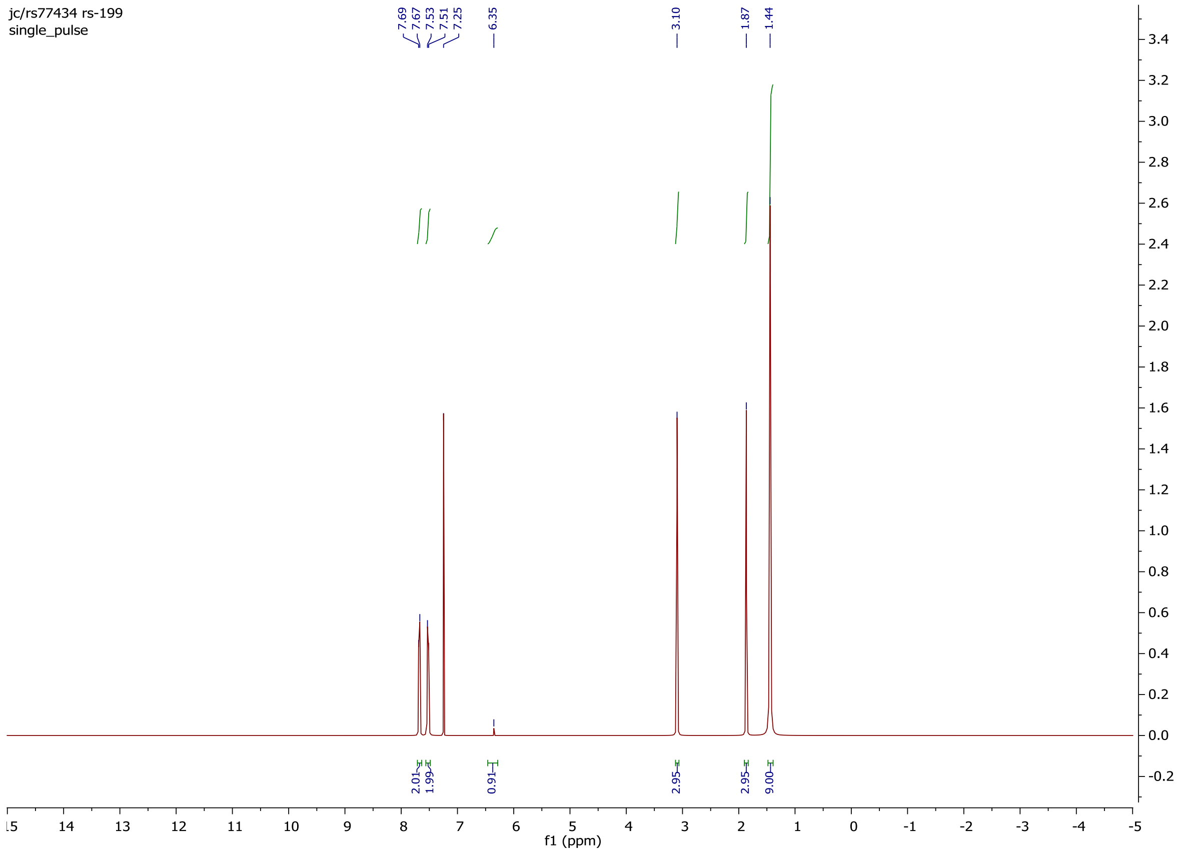

^{13}C NMR

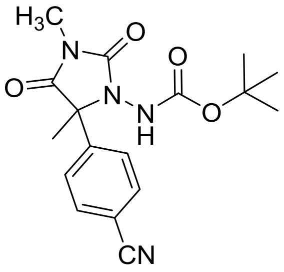

***tert*-Butyl (5-(4-cyanophenyl)-3,5-dimethyl-2,4-dioxoimidazolidin-1-yl)carbamate (7c)**

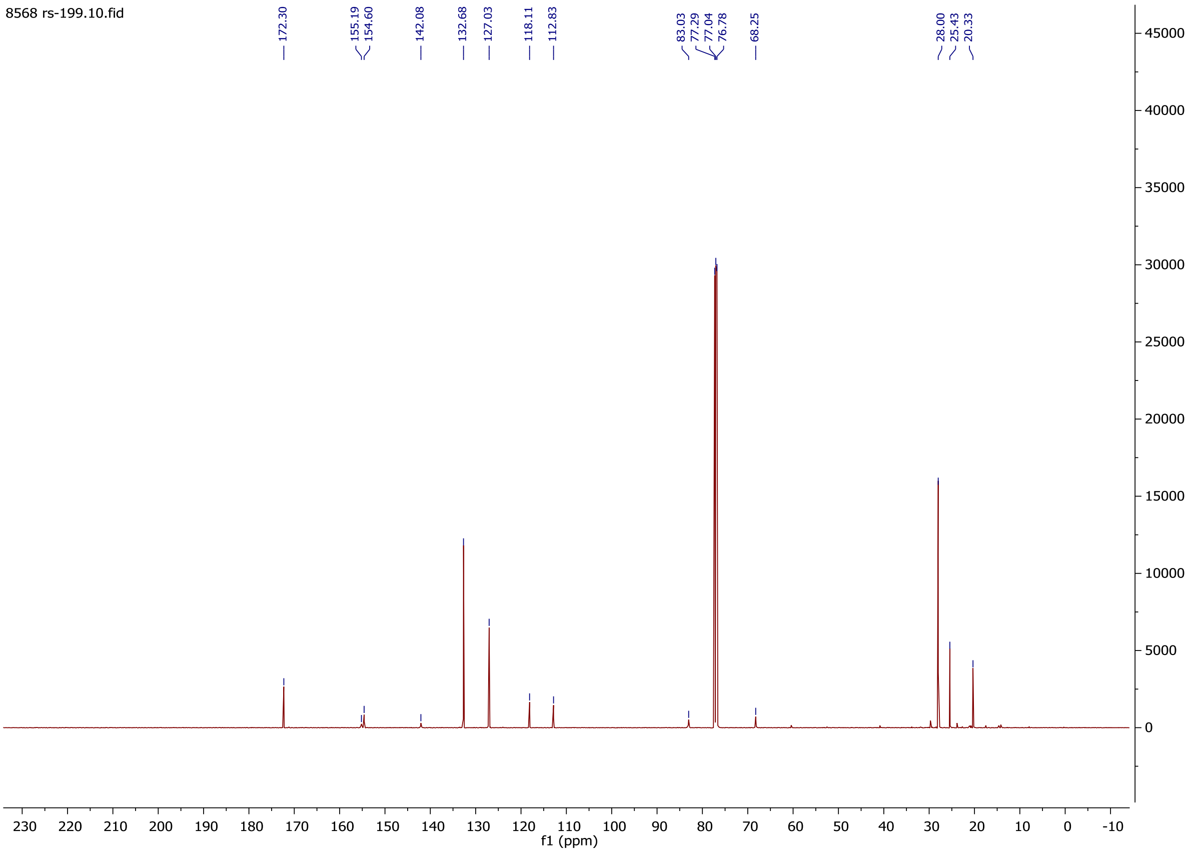

^{1}H NMR

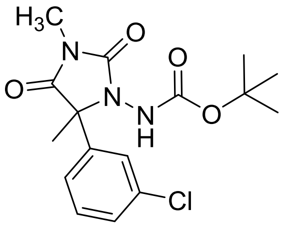

tert-Butyl (5-(3-chlorophenyl)-3,5-dimethyl-2,4-dioxoimidazolidin-1-yl)carbamate (7d)

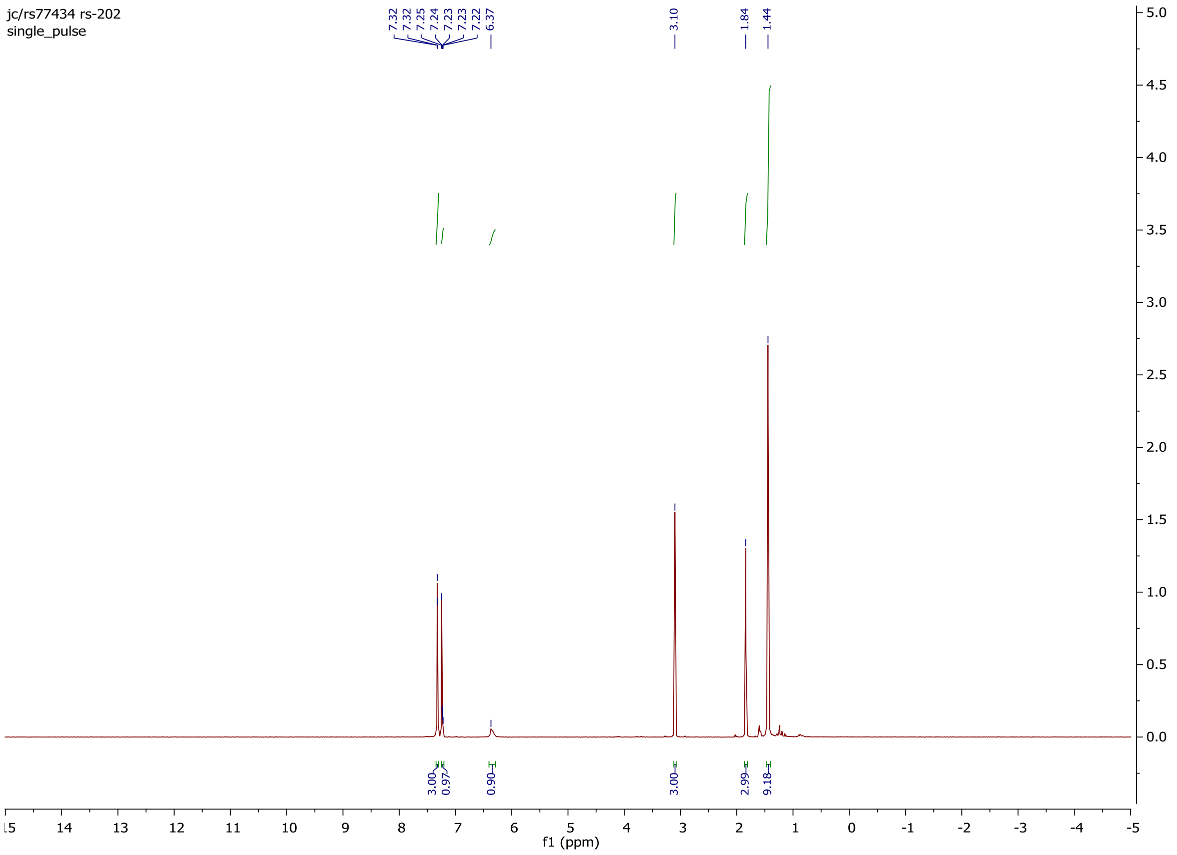

^{13}C NMR

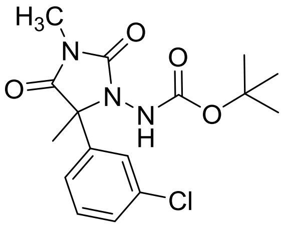

tert-Butyl (5-(3-chlorophenyl)-3,5-dimethyl-2,4-dioxoimidazolidin-1-yl)carbamate (7d)

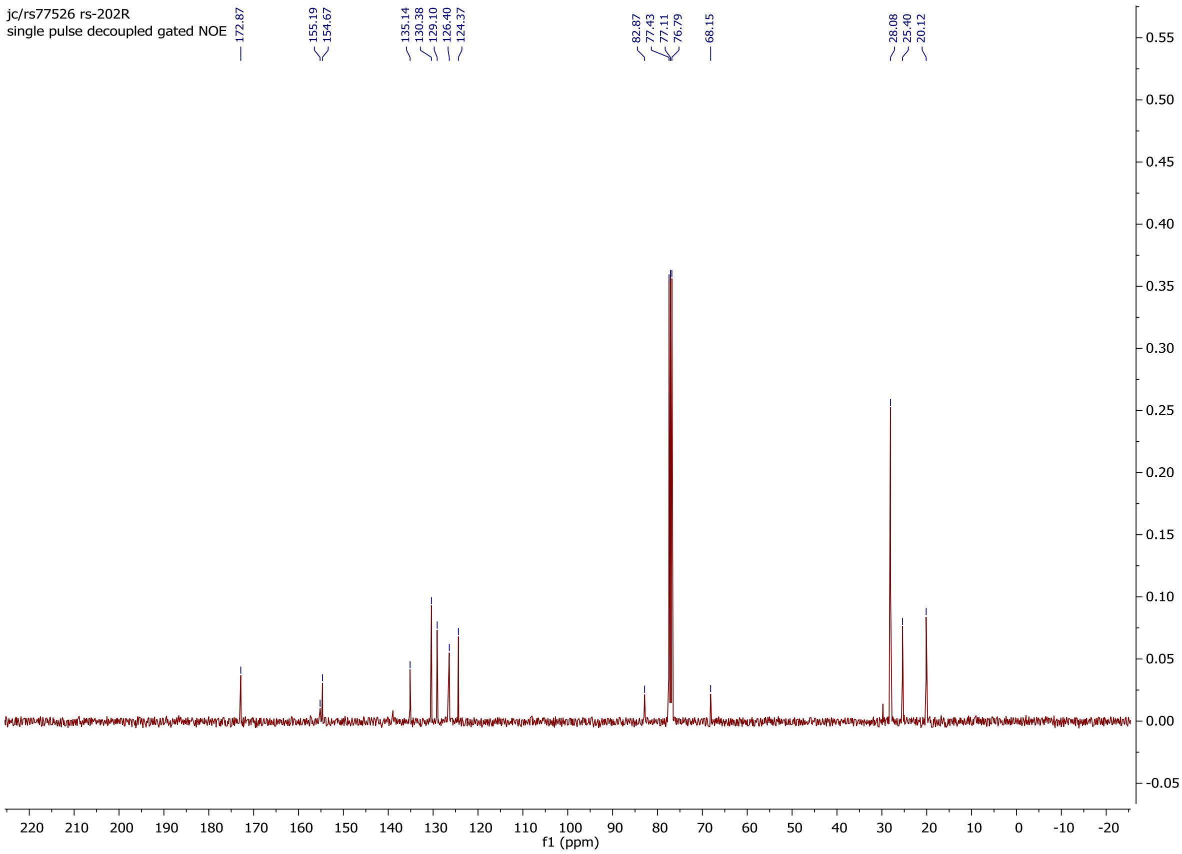

1H NMR

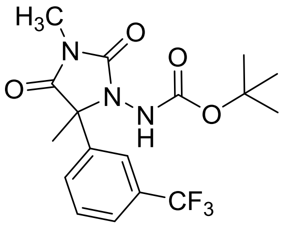

***tert*-Butyl (3,5-dimethyl-2,4-dioxo-5-(3-(trifluoromethyl)phenyl)imidazolidin-1-yl)carbamate (7e)**

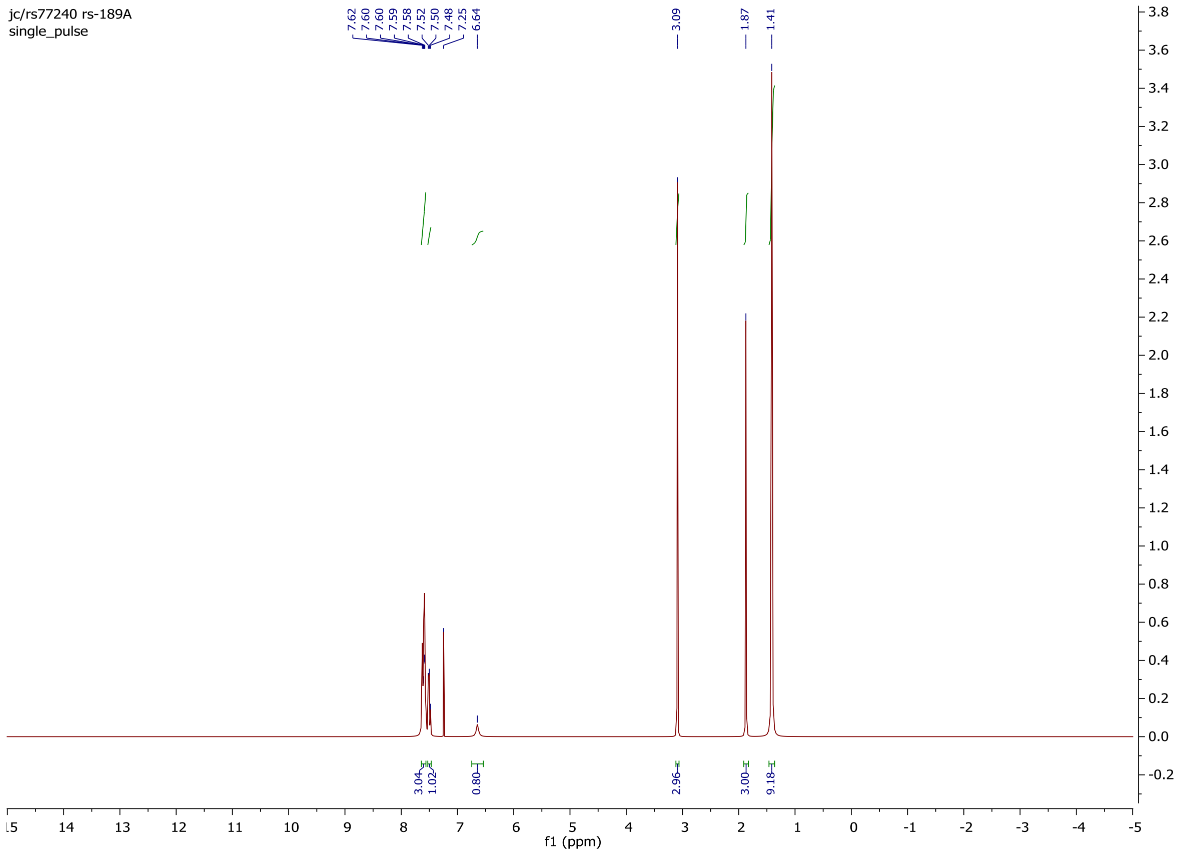

^{13}C NMR

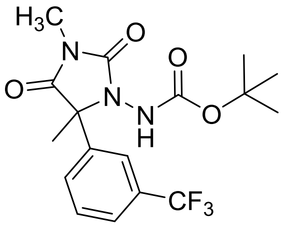

***tert*-Butyl (3,5-dimethyl-2,4-dioxo-5-(3-(trifluoromethyl)phenyl)imidazolidin-1-yl)carbamate (7e)**

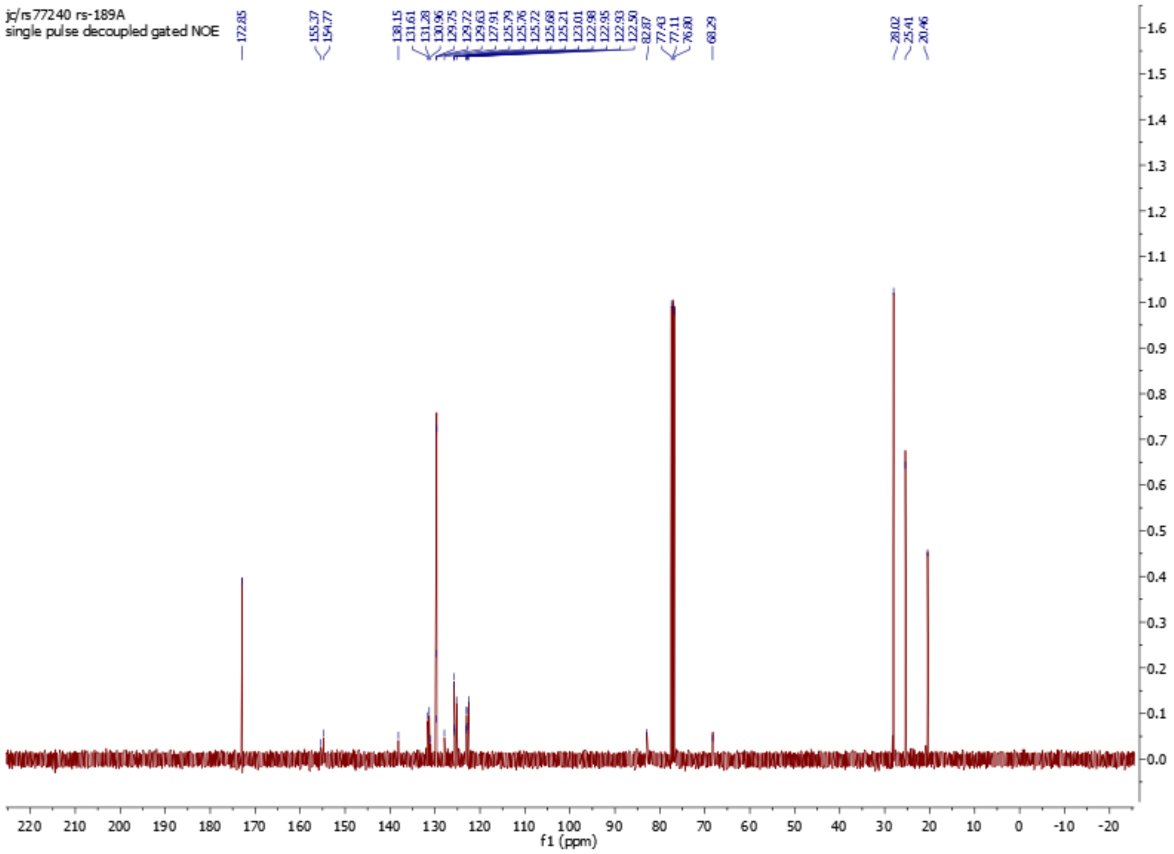

^{1}H NMR

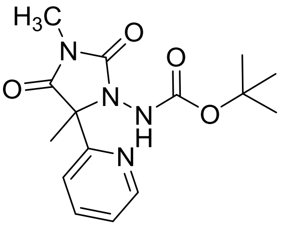

***tert*-Butyl (3,5-dimethyl-2,4-dioxo-5-(pyridin-2-yl)imidazolidin-1-yl)carbamate (7f)**

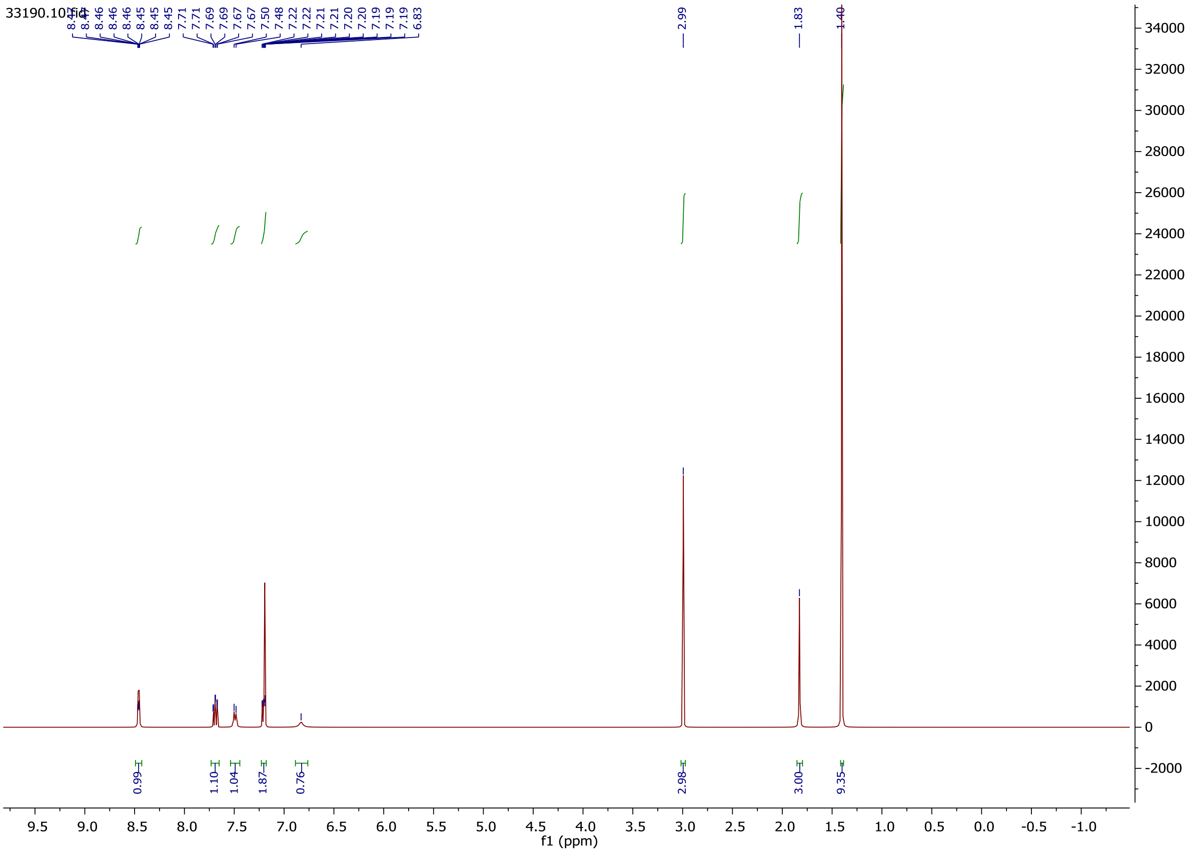

^{13}C NMR

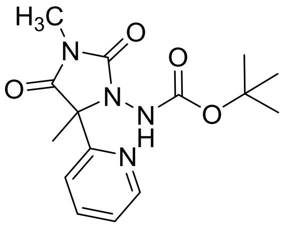

***tert*-Butyl (3,5-dimethyl-2,4-dioxo-5-(pyridin-2-yl)imidazolidin-1-yl)carbamate (7f)**

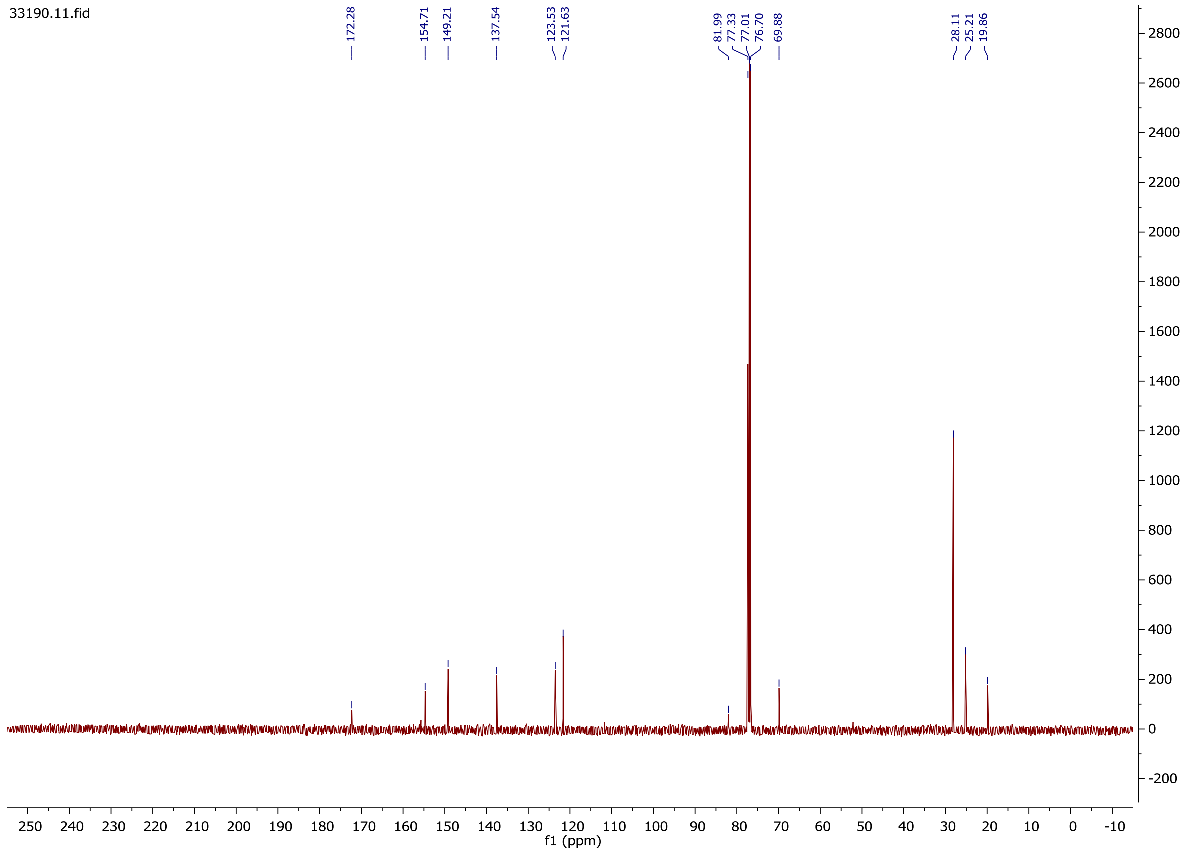

^1H NMR

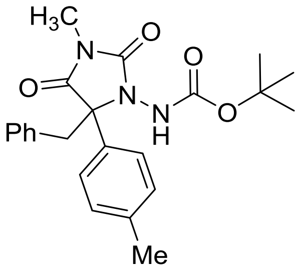

***tert*-Butyl (5-benzyl-3-methyl-2,4-dioxo-5-(*p*-tolyl)imidazolidin-1-yl)carbamate (7g)**

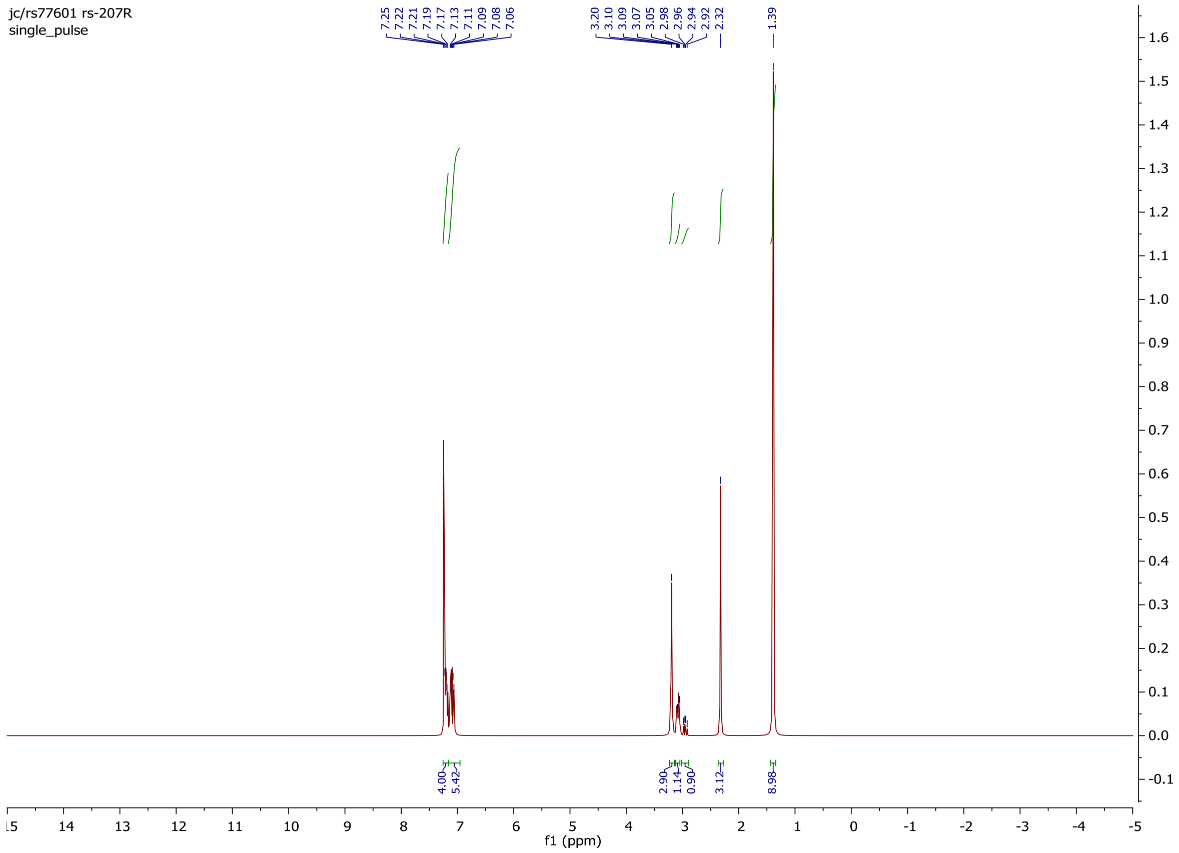

^{13}C NMR

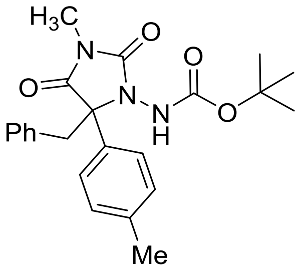

tert-Butyl (5-benzyl-3-methyl-2,4-dioxo-5-(p-tolyl)imidazolidin-1-yl)carbamate (7g)

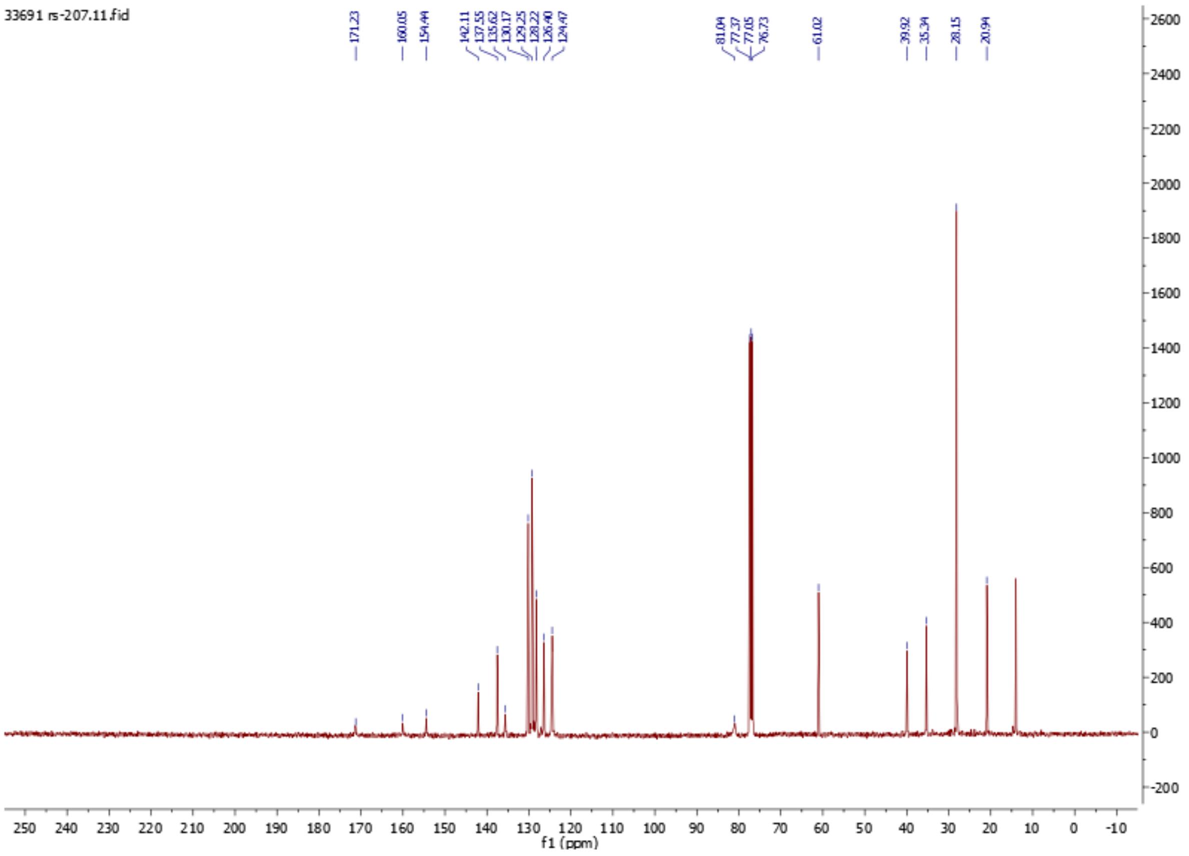

^{1}H NMR

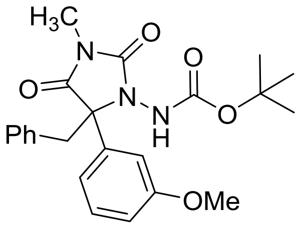

***tert*-Butyl (5-benzyl-5-(3-methoxyphenyl)-3-methyl-2,4-dioxoimidazolidin-1-yl)carbamate**

(7h)

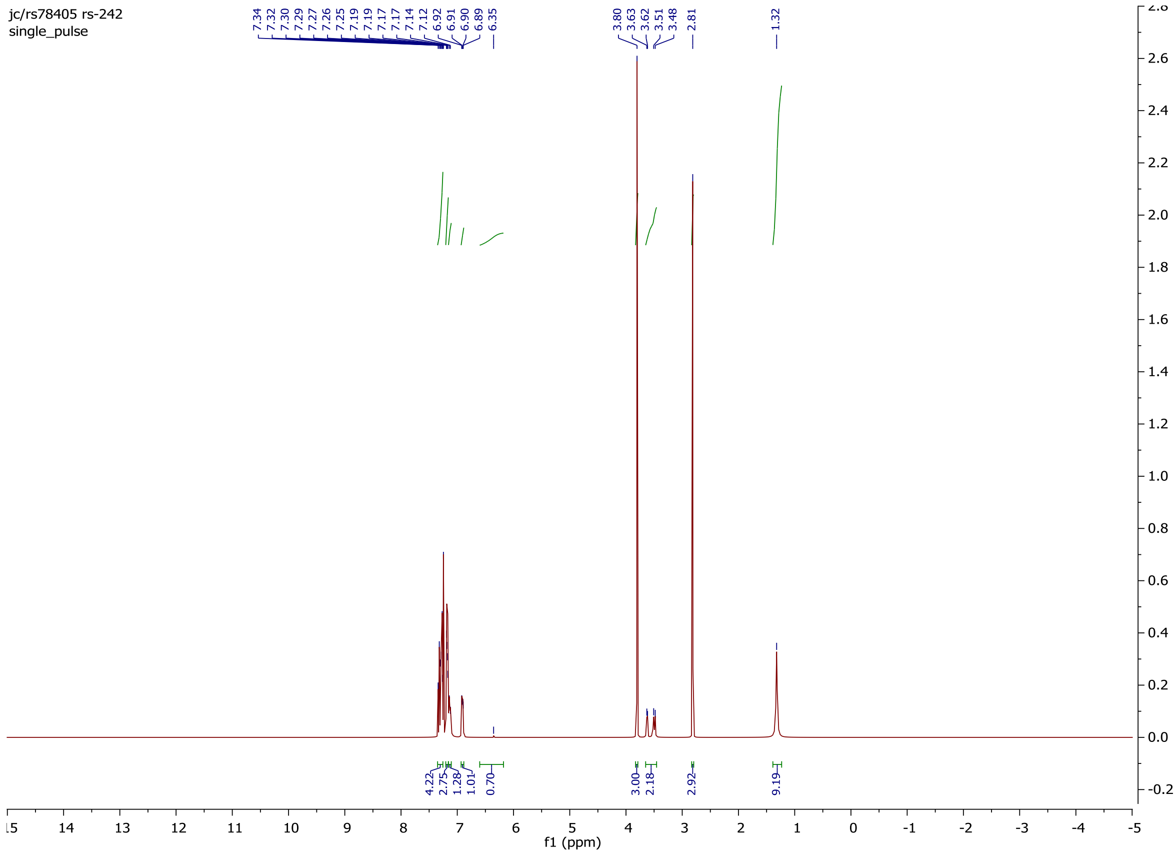

^{13}C NMR

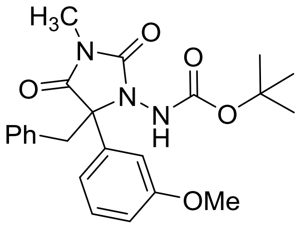

tert-Butyl (5-benzyl-5-(3-methoxyphenyl)-3-methyl-2,4-dioxoimidazolidin-1-yl)carbamate (7h)

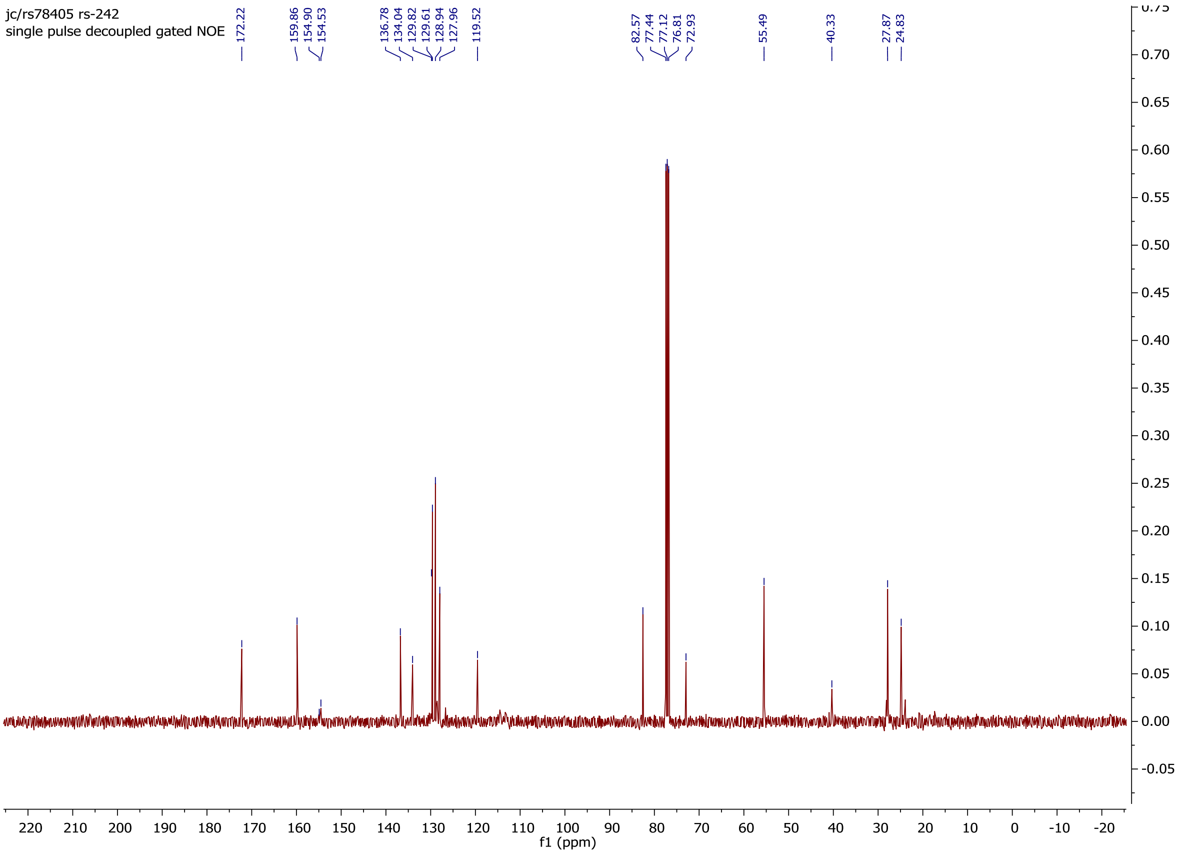

^{1}H NMR

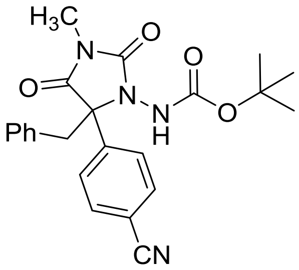

tert-Butyl (5-benzyl-5-(4-cyanophenyl)-3-methyl-2,4-dioxoimidazolidin-1-yl)carbamate (7i)

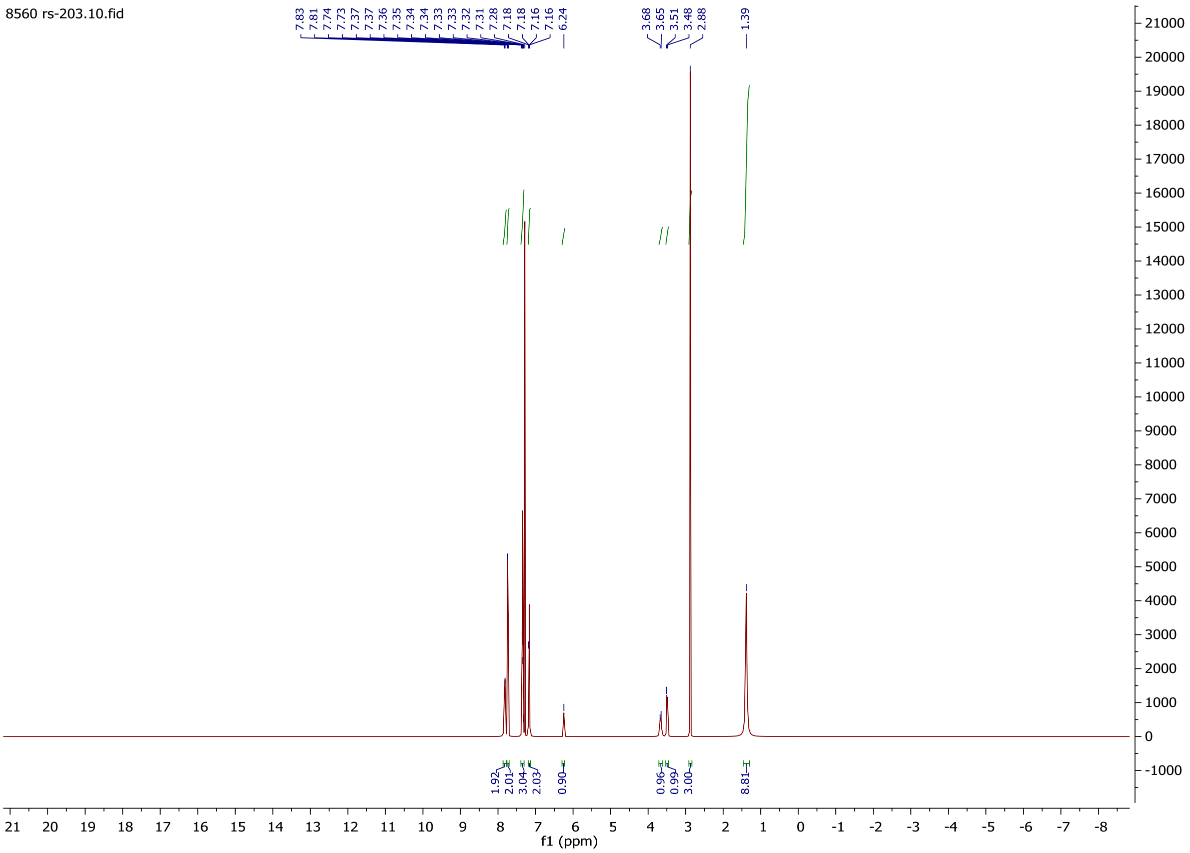

^{13}C NMR

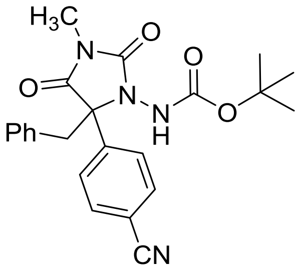

tert-Butyl (5-benzyl-5-(4-cyanophenyl)-3-methyl-2,4-dioxoimidazolidin-1-yl)carbamate

(7i)

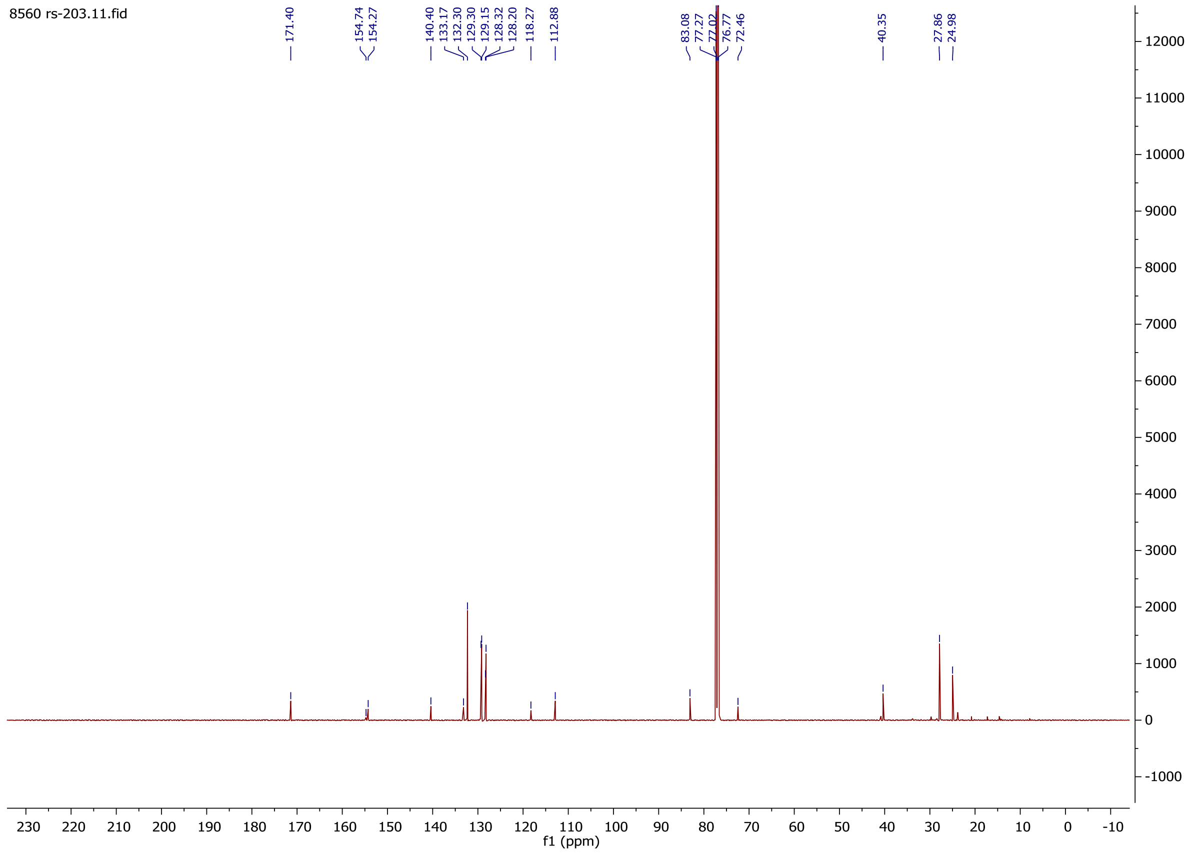

^{1}H NMR

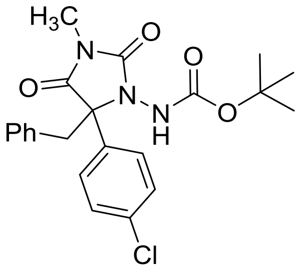

***tert*-Butyl (5-benzyl-5-(4-chlorophenyl)-3-methyl-2,4-dioxoimidazolidin-1-yl)carbamate**

(7j)

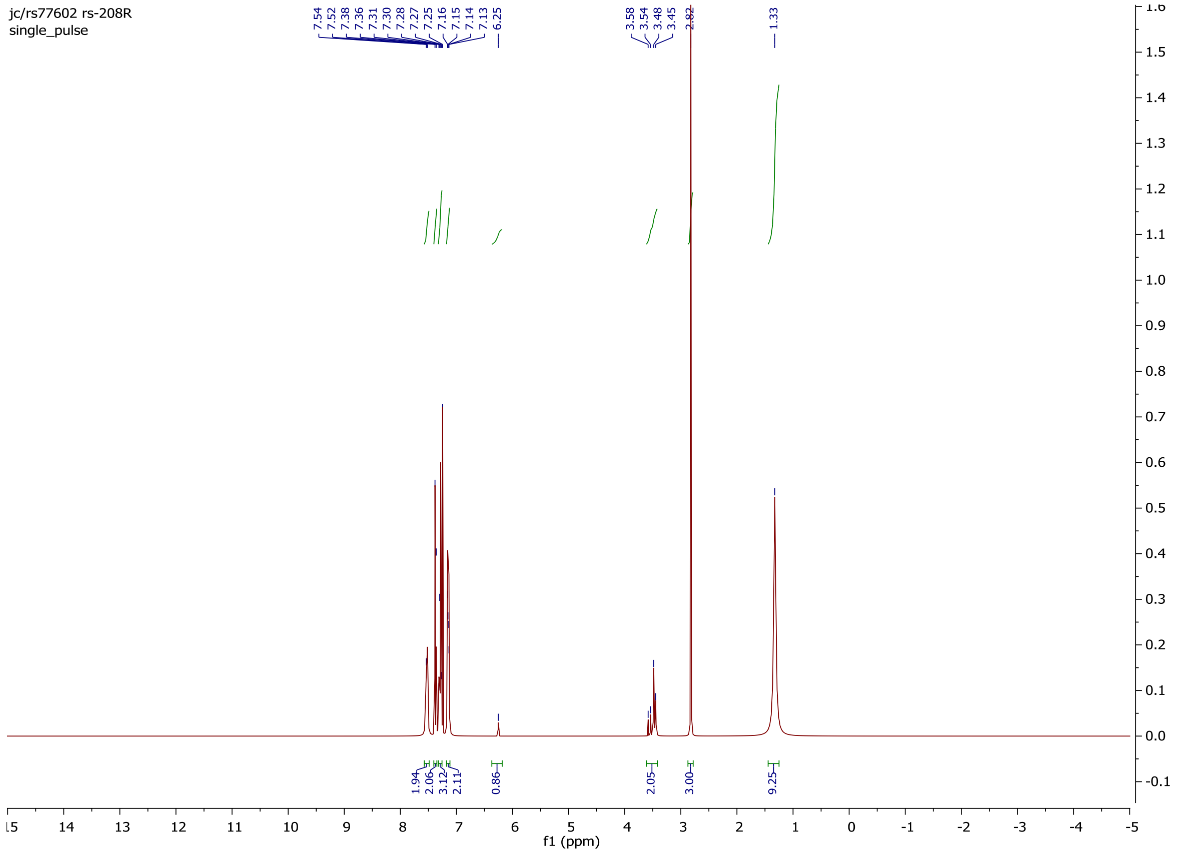

^{13}C NMR

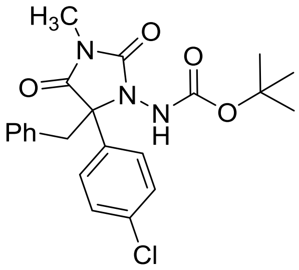

***tert*-Butyl (5-benzyl-5-(4-chlorophenyl)-3-methyl-2,4-dioxoimidazolidin-1-yl)carbamate**

(7j)

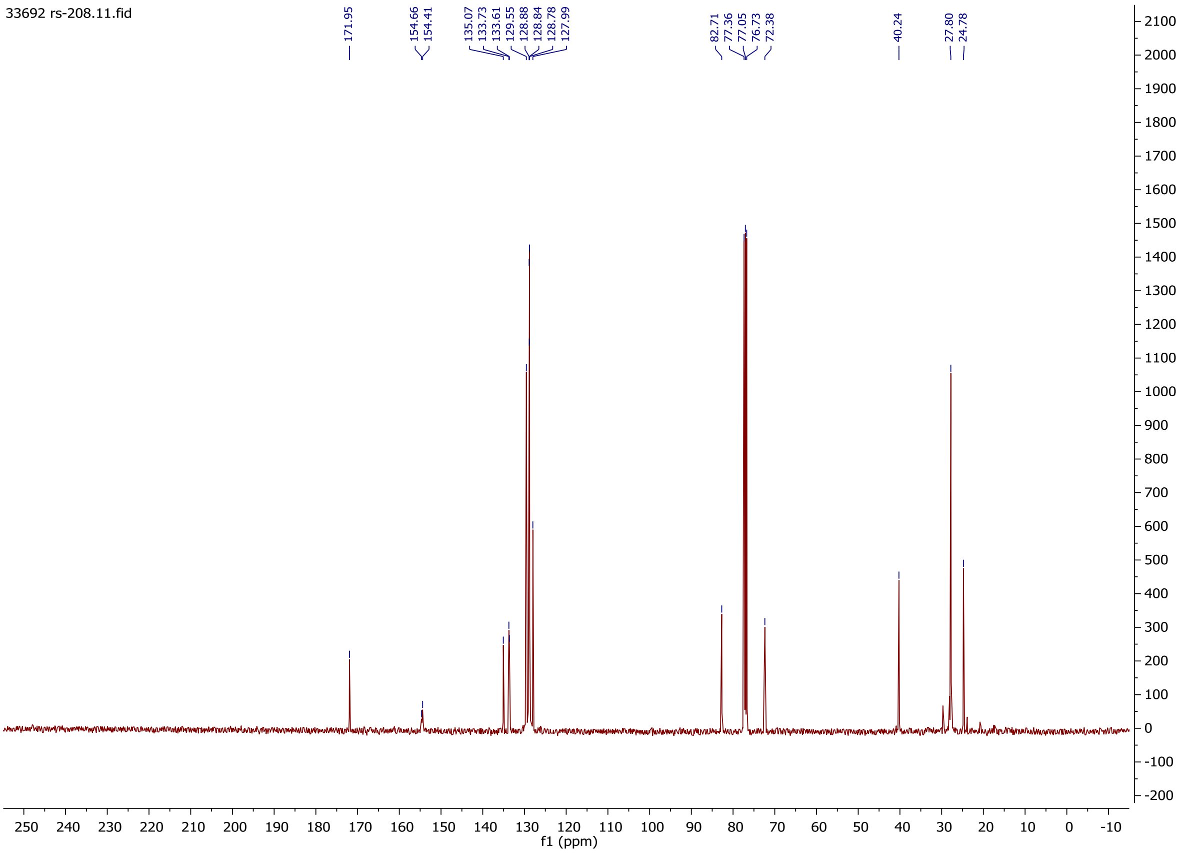

^{1}H NMR

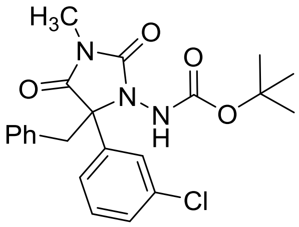

***tert*-Butyl (5-benzyl-5-(3-chlorophenyl)-3-methyl-2,4-dioxoimidazolidin-1-yl)carbamate**

(7k)

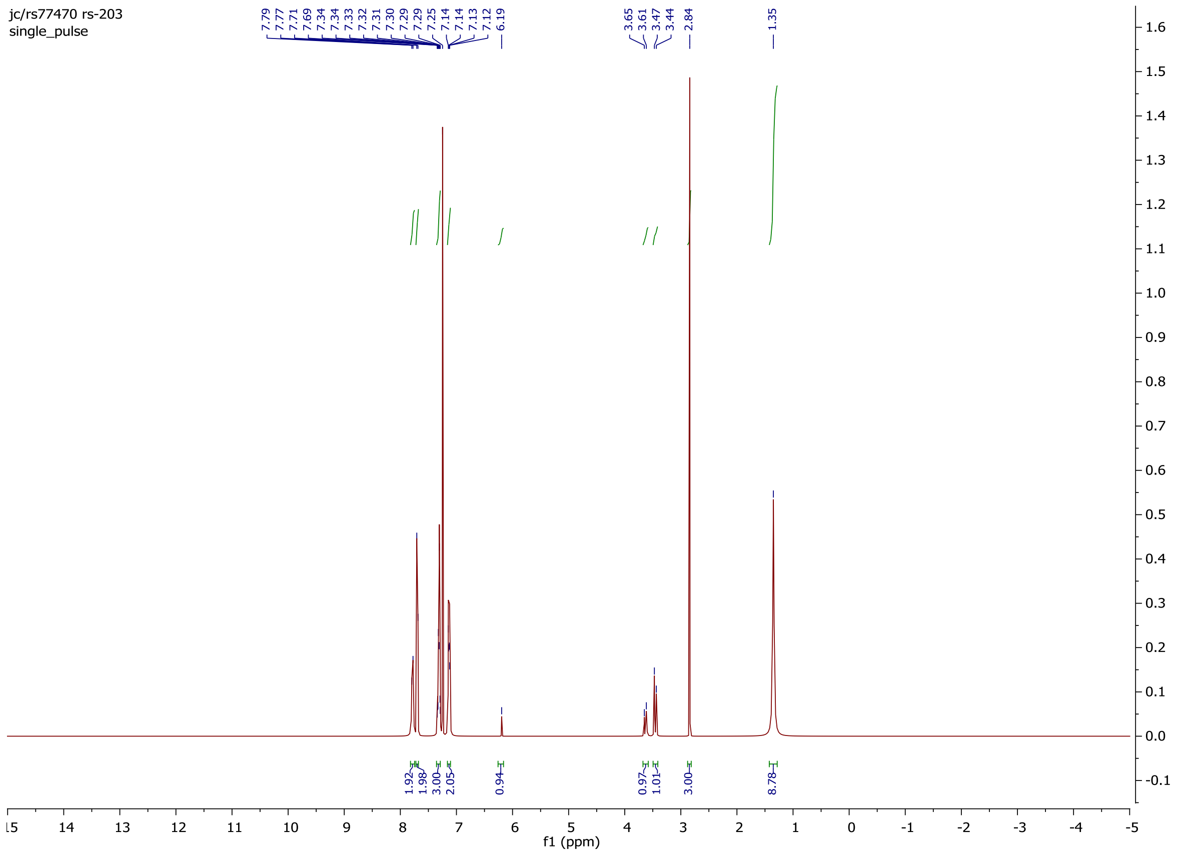

^{13}C NMR

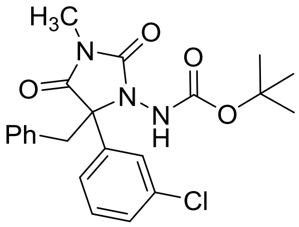

tert-Butyl (5-benzyl-5-(3-chlorophenyl)-3-methyl-2,4-dioxoimidazolidin-1-yl)carbamate

(7k)

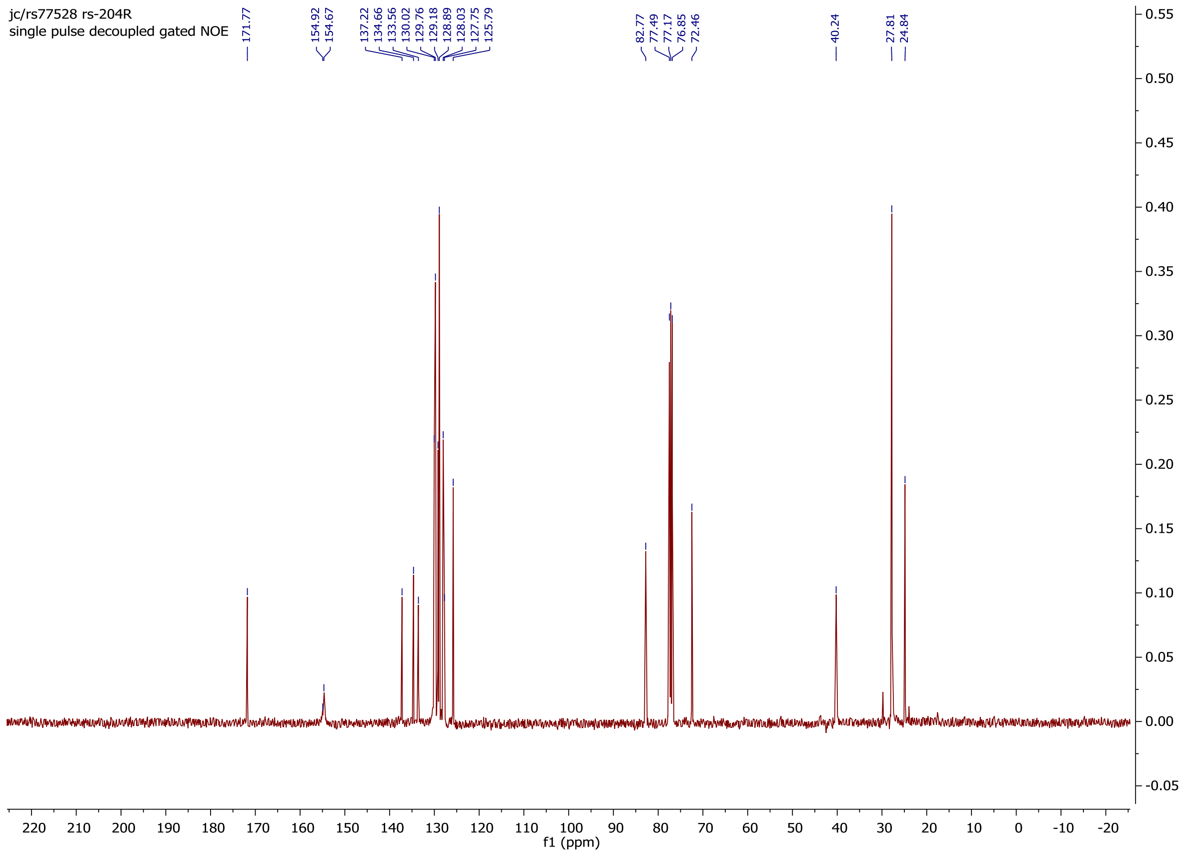

^{1}H NMR

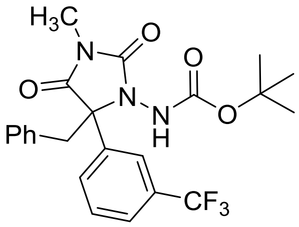

tert-Butyl (5-benzyl-3-methyl-2,4-dioxo-5-(3-(trifluoromethyl)phenyl)imidazolidin-1-yl)carbamate (7l)

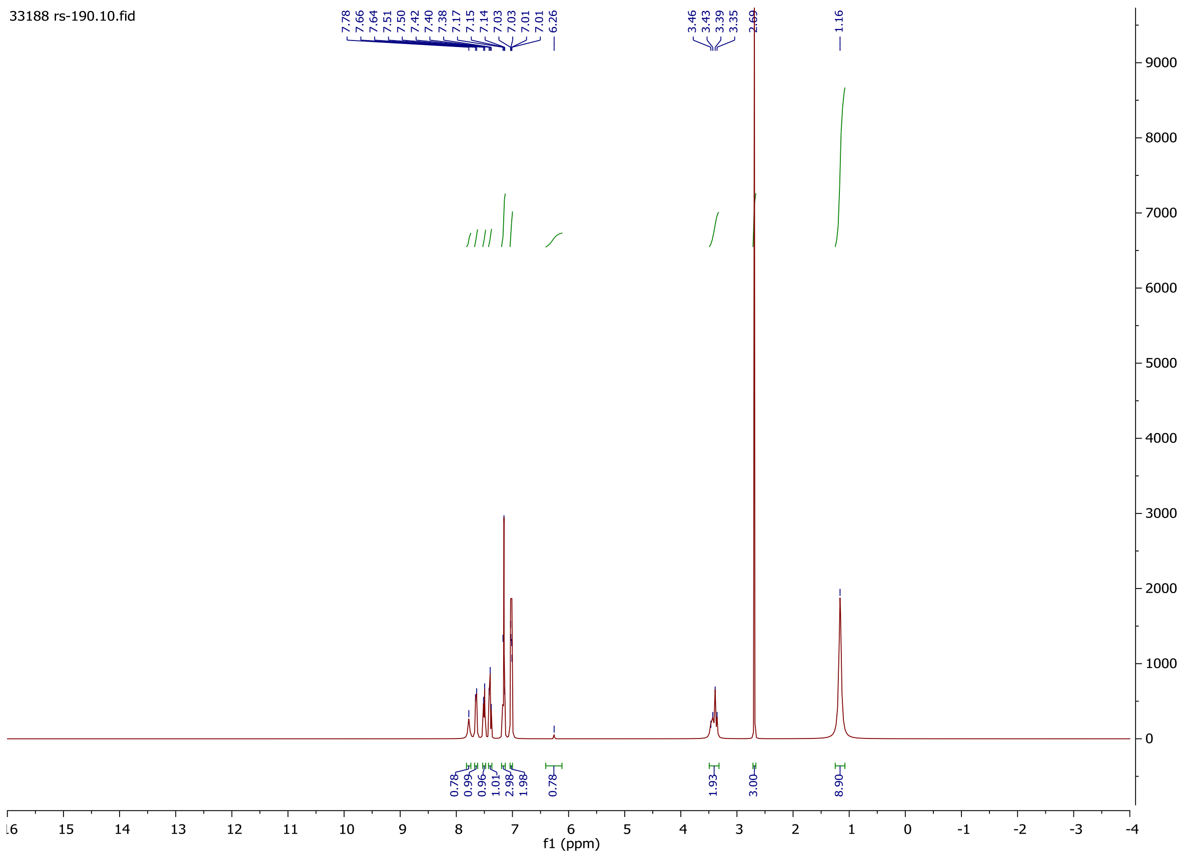

^{13}C NMR

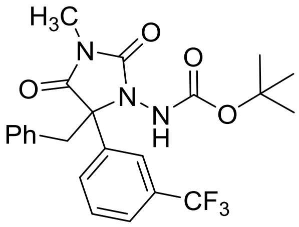

***tert*-Butyl (5-benzyl-3-methyl-2,4-dioxo-5-(3-(trifluoromethyl)phenyl)imidazolidin-1-yl)carbamate (7l)**

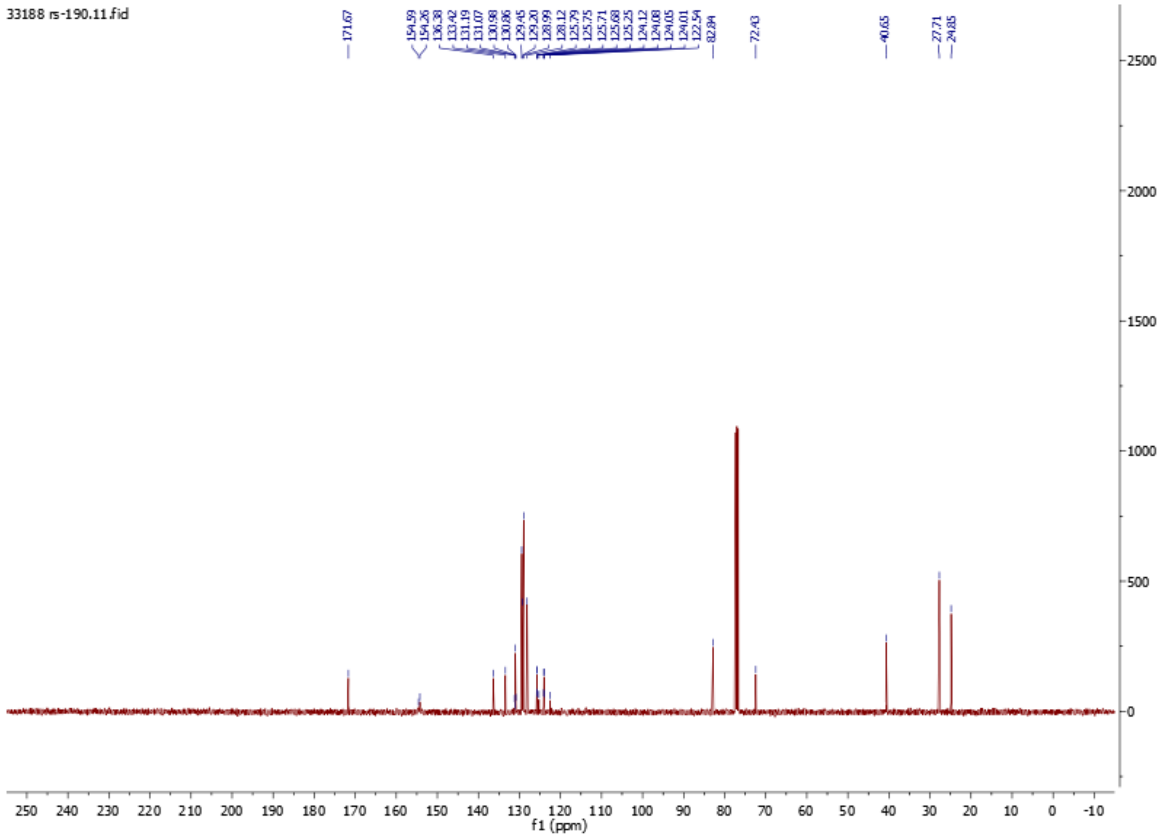

^{1}H NMR

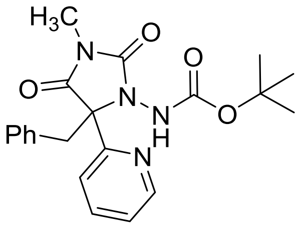

***tert*-Butyl (5-benzyl-3-methyl-2,4-dioxo-5-(3-(trifluoromethyl)phenyl)imidazolidin-1-yl) carbamate (7m)**

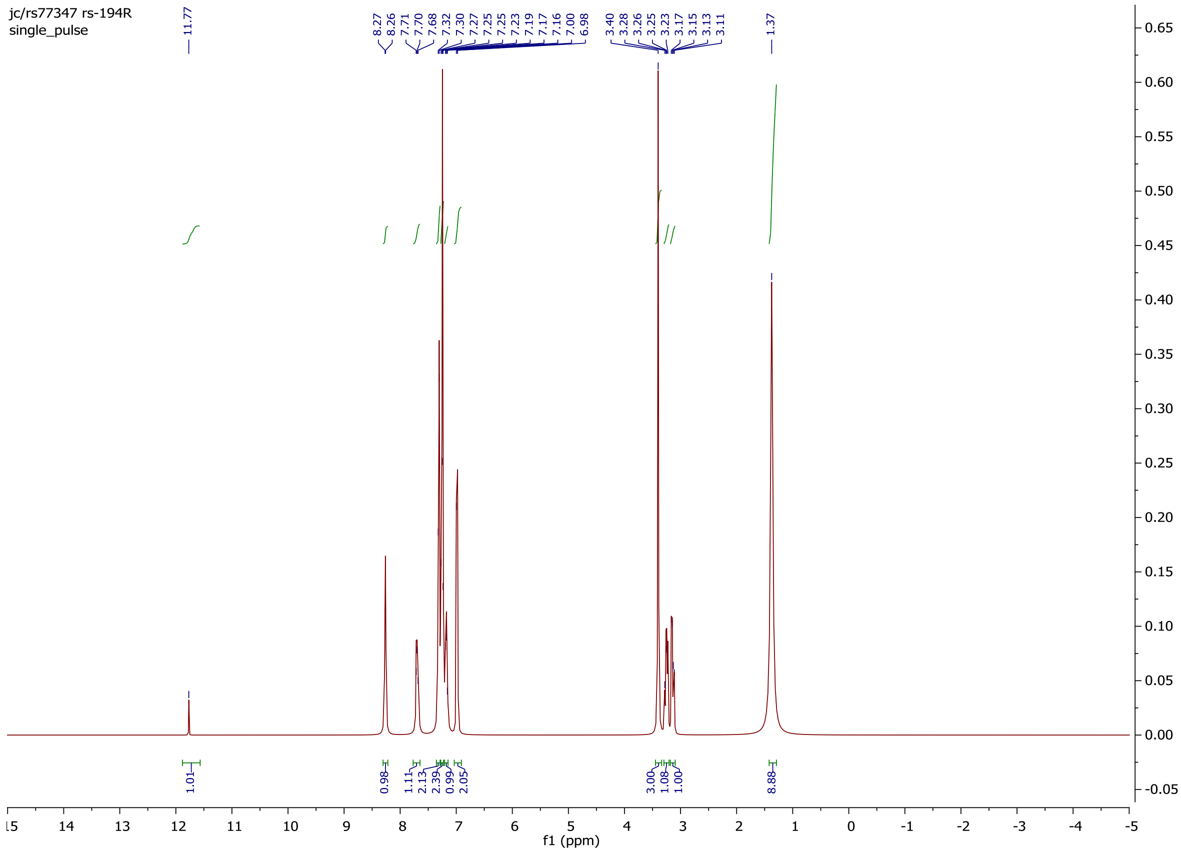

^{13}C NMR

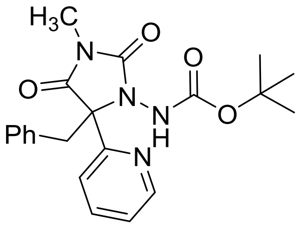

tert-Butyl (5-benzyl-3-methyl-2,4-dioxo-5-(3-(trifluoromethyl)phenyl)imidazolidin-1-yl) carbamate (7m)

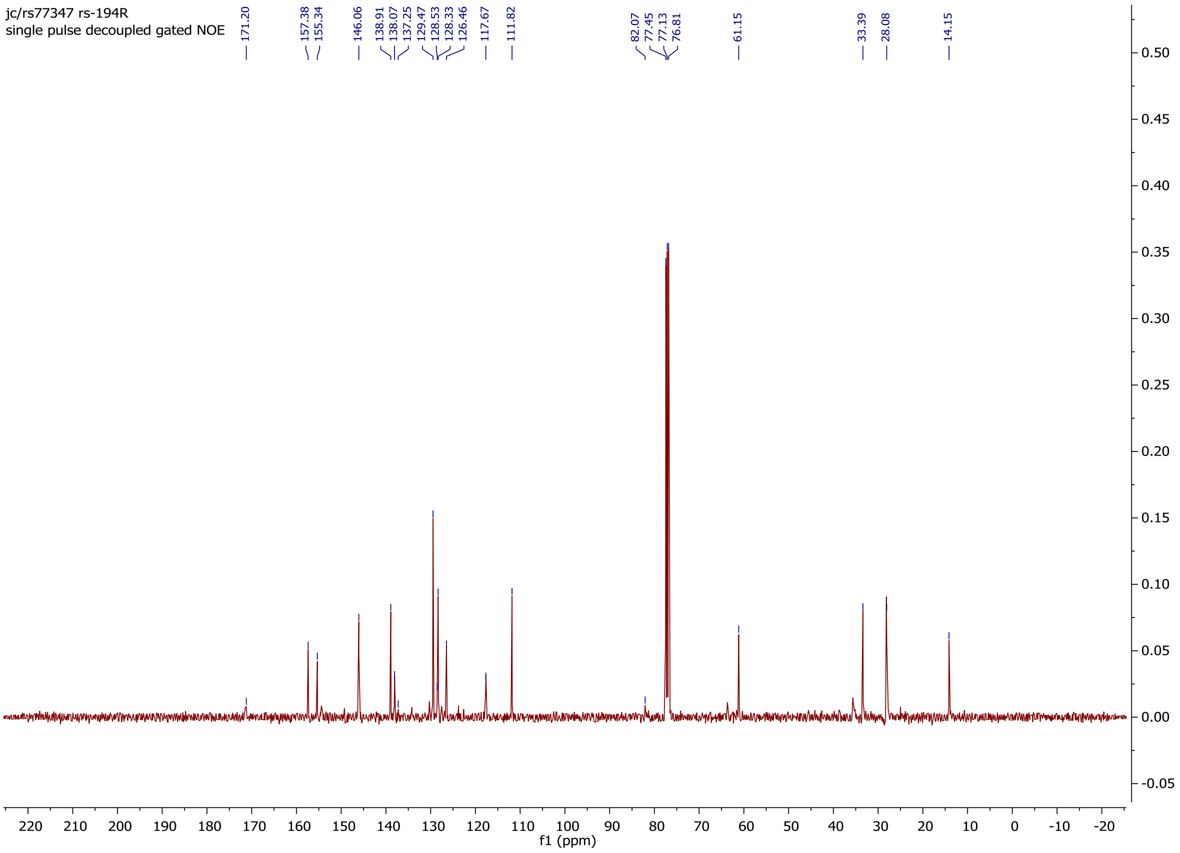

^{1}H NMR

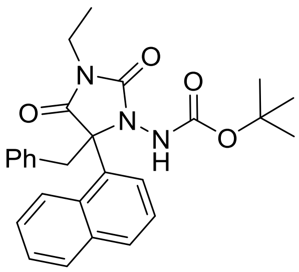

***tert*-Butyl (5-benzyl-3-methyl-5-(naphthalen-1-yl)-2,4-dioxoimidazolidin-1-yl)carbamate**

(7n)

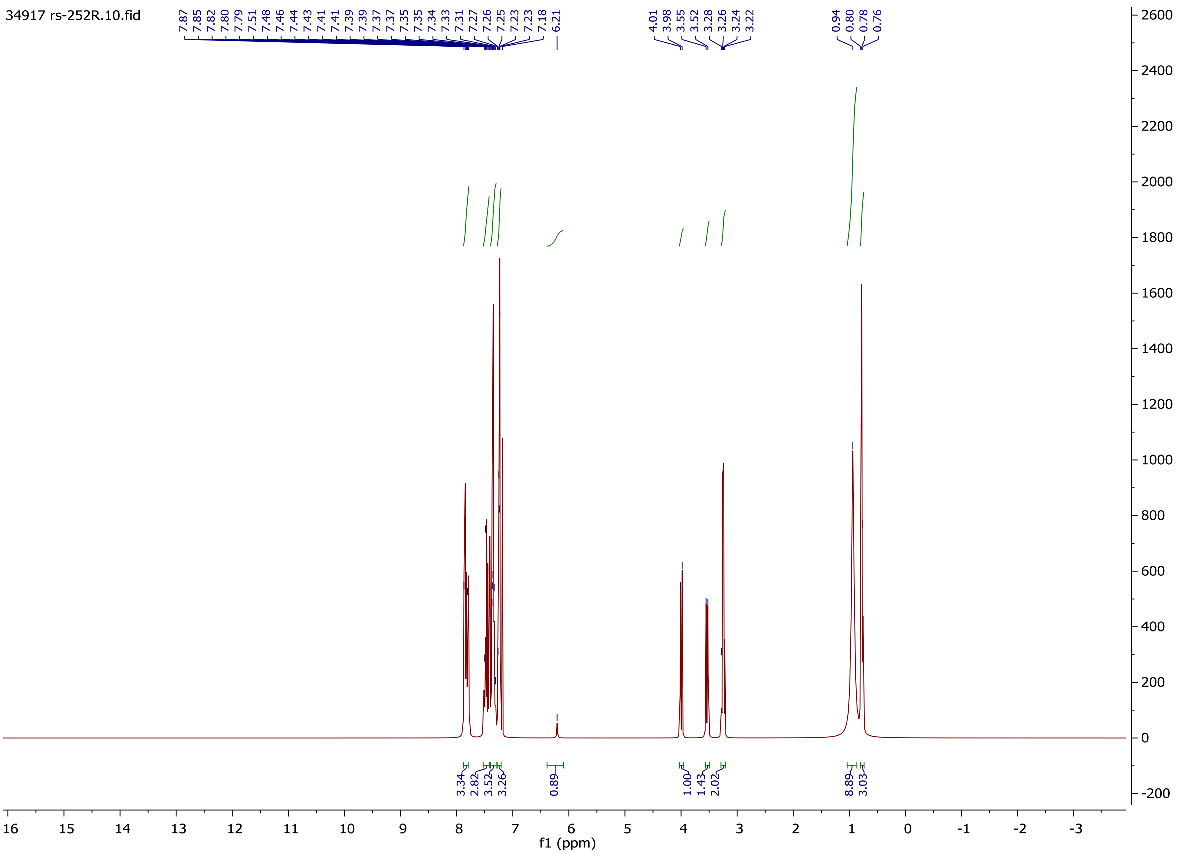

^{13}C NMR

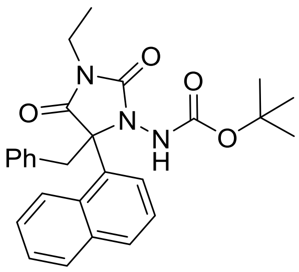

***tert*-Butyl (5-benzyl-3-methyl-5-(naphthalen-1-yl)-2,4-dioxoimidazolidin-1-yl)carbamate**

(7n)

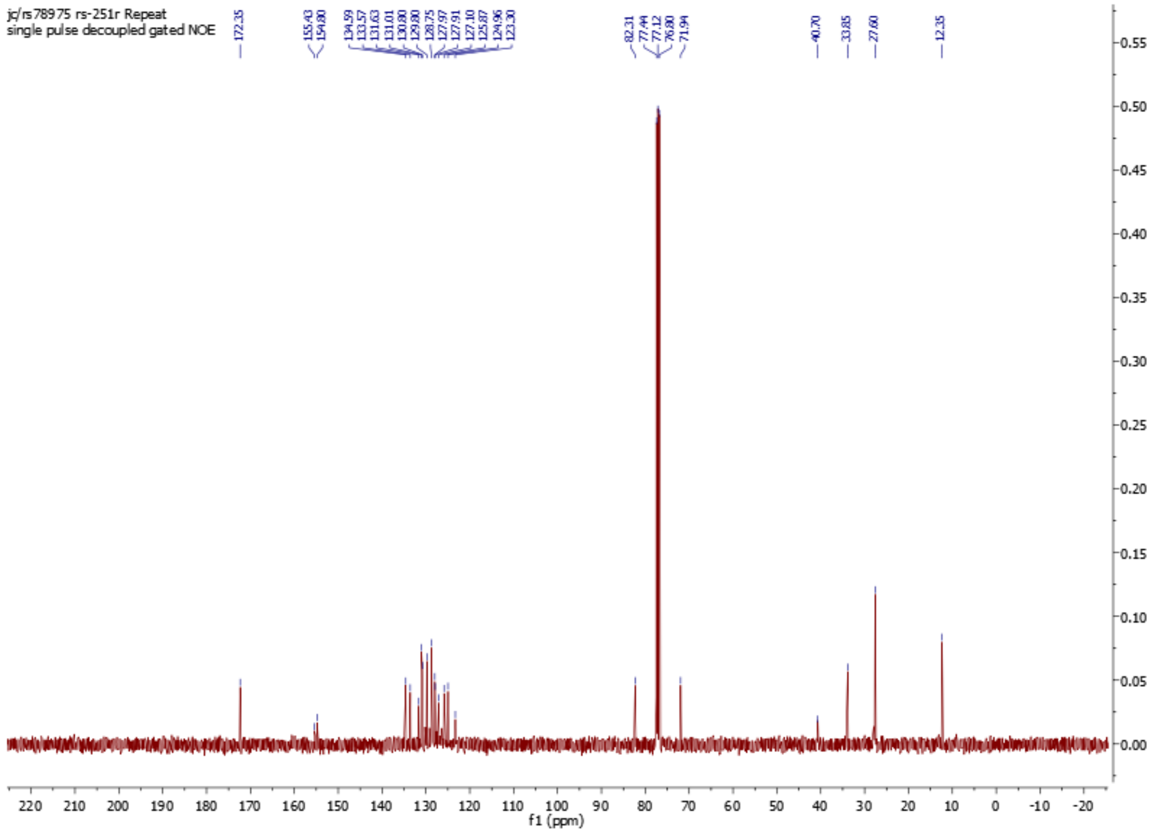

^{1}H NMR

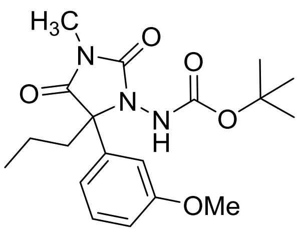

tert*-Butyl *(5-(3-methoxyphenyl)-3-methyl-2,4-dioxo-5-propylimidazolidin-1-yl)carbamate

(7o)

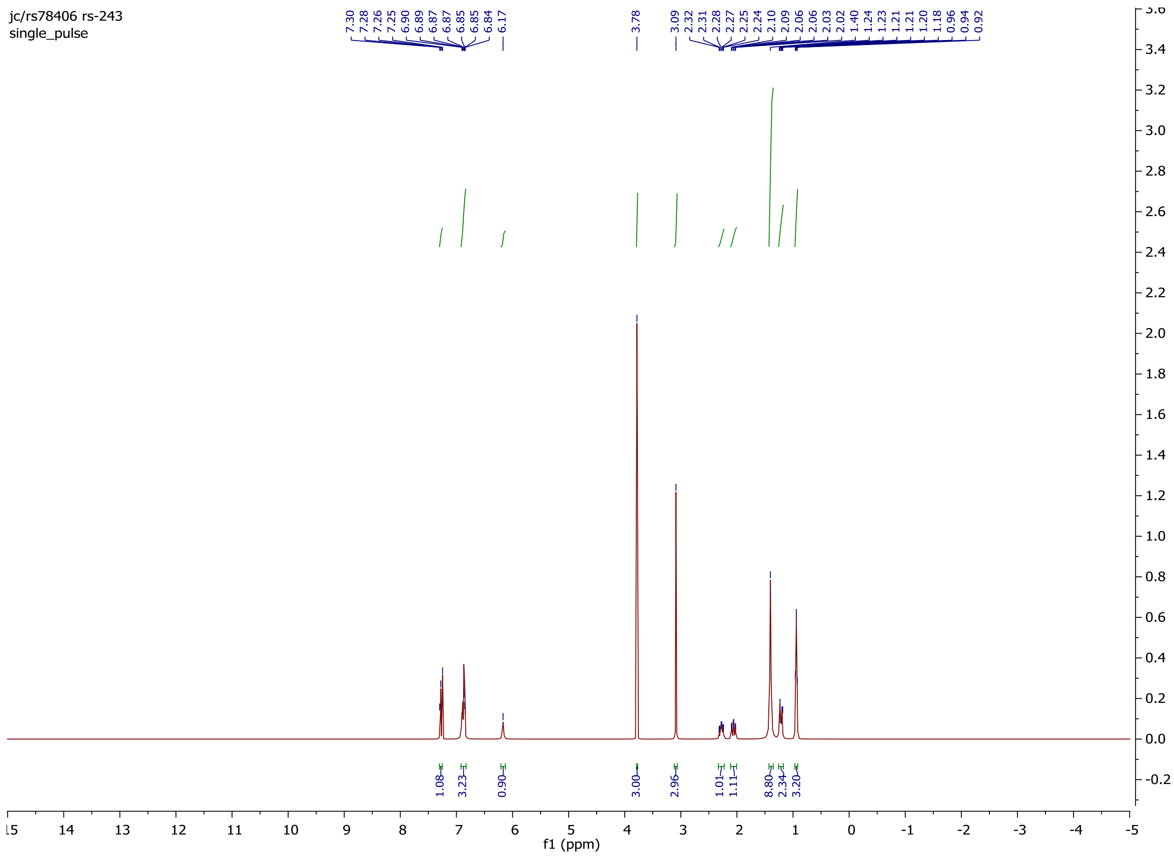

^{13}C NMR

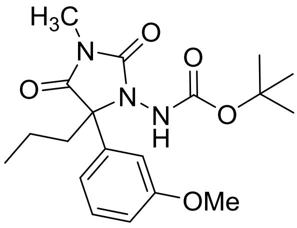

***tert*-Butyl (5-(3-methoxyphenyl)-3-methyl-2,4-dioxo-5-propylimidazolidin-1-yl)carbamate**

(7o)

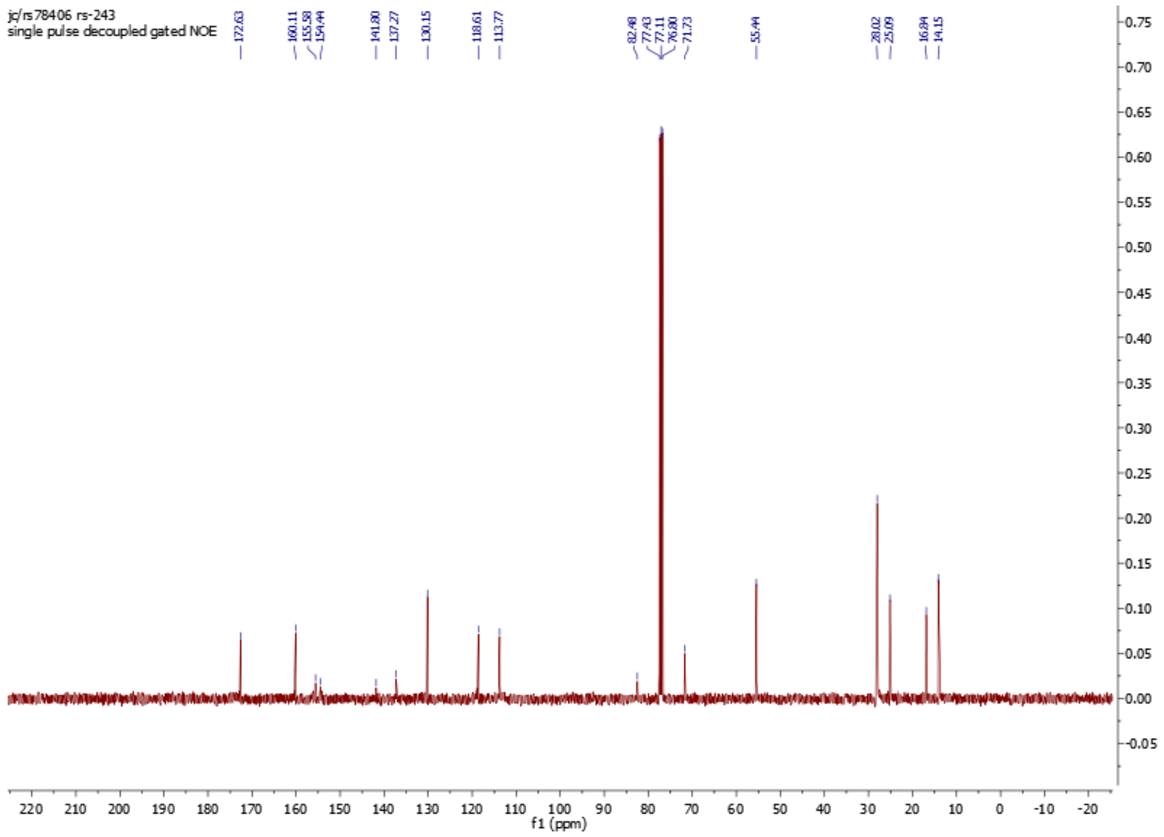

^{1}H NMR

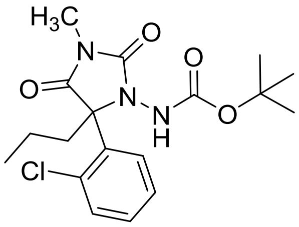

***tert*-Butyl (5-(2-chlorophenyl)-3-methyl-2,4-dioxo-5-propylimidazolidin-1-yl)carbamate**

(7p)

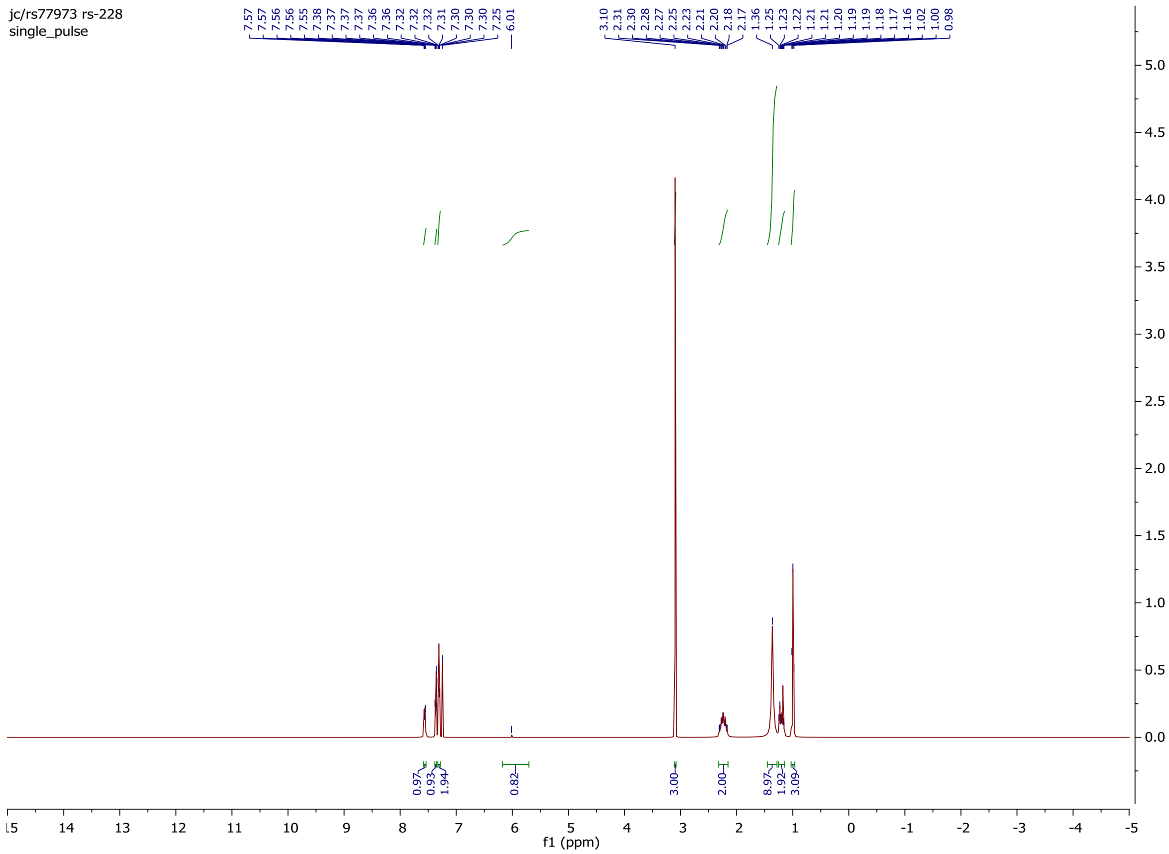

^{13}C NMR

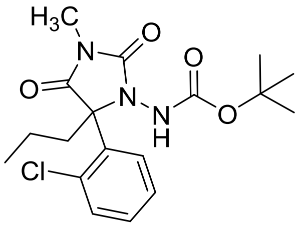

***tert*-Butyl (5-(2-chlorophenyl)-3-methyl-2,4-dioxo-5-propylimidazolidin-1-yl)carbamate**

(7p)

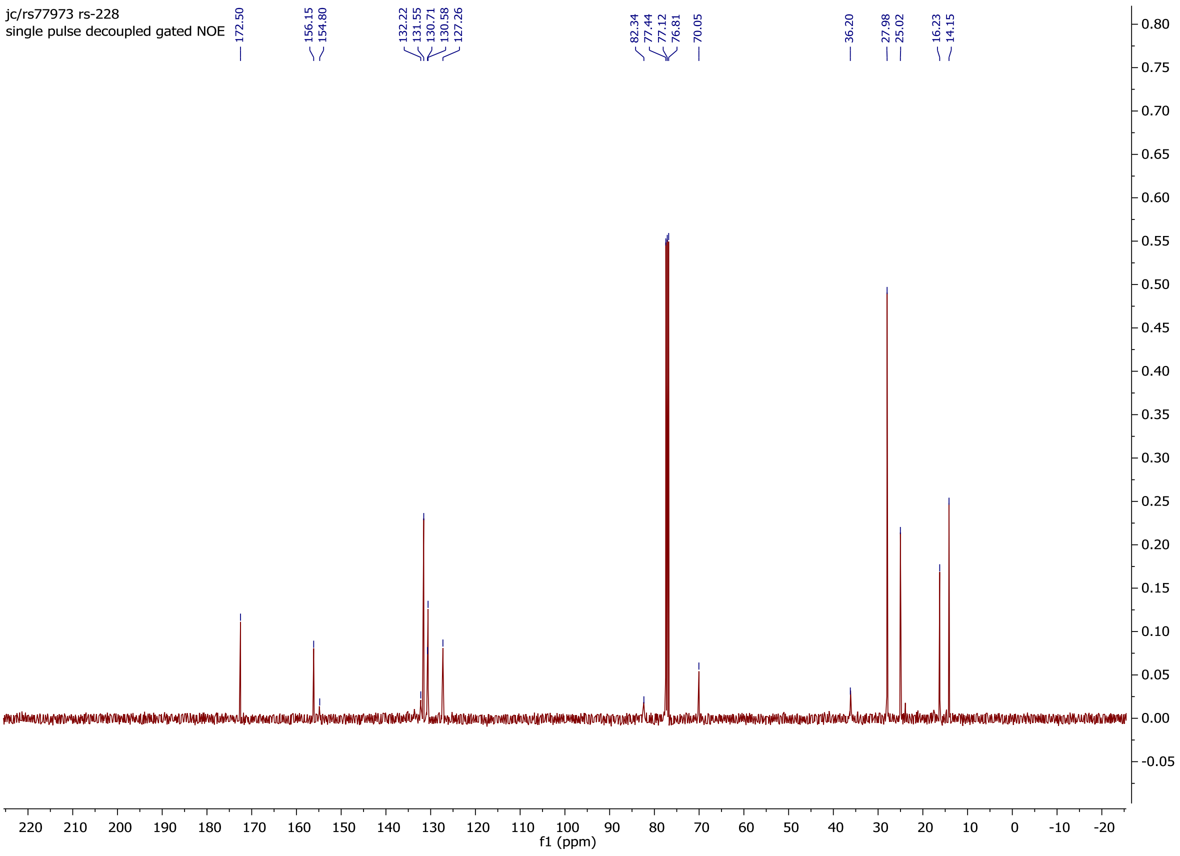

^{1}H NMR

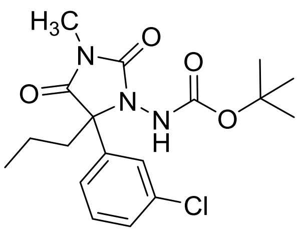

***tert*-Butyl (5-(3-chlorophenyl)-3-methyl-2,4-dioxo-5-propylimidazolidin-1-yl)carbamate (7q)**

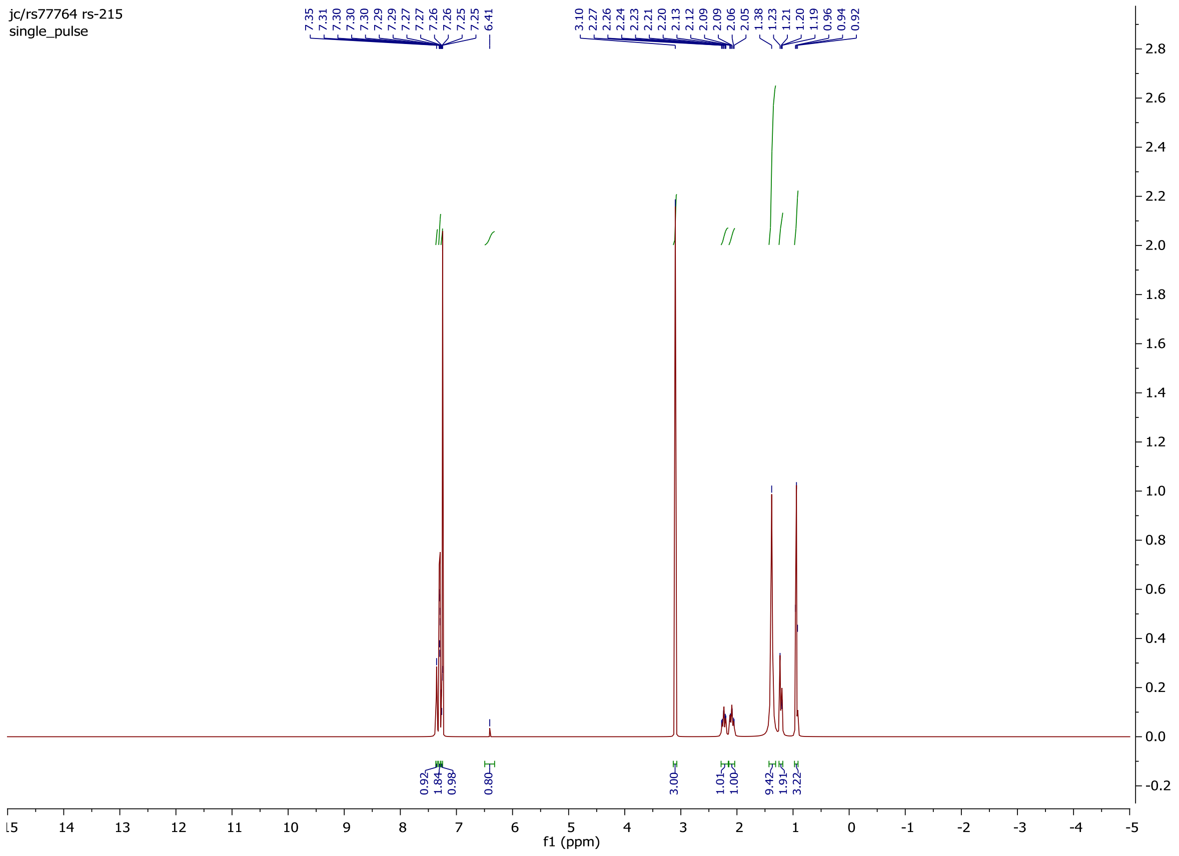

^{13}C NMR

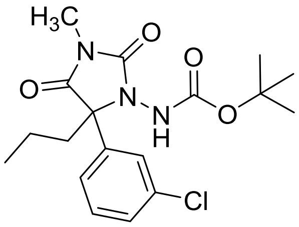

***tert*-Butyl (5-(3-chlorophenyl)-3-methyl-2,4-dioxo-5-propylimidazolidin-1-yl)carbamate**

(7q)

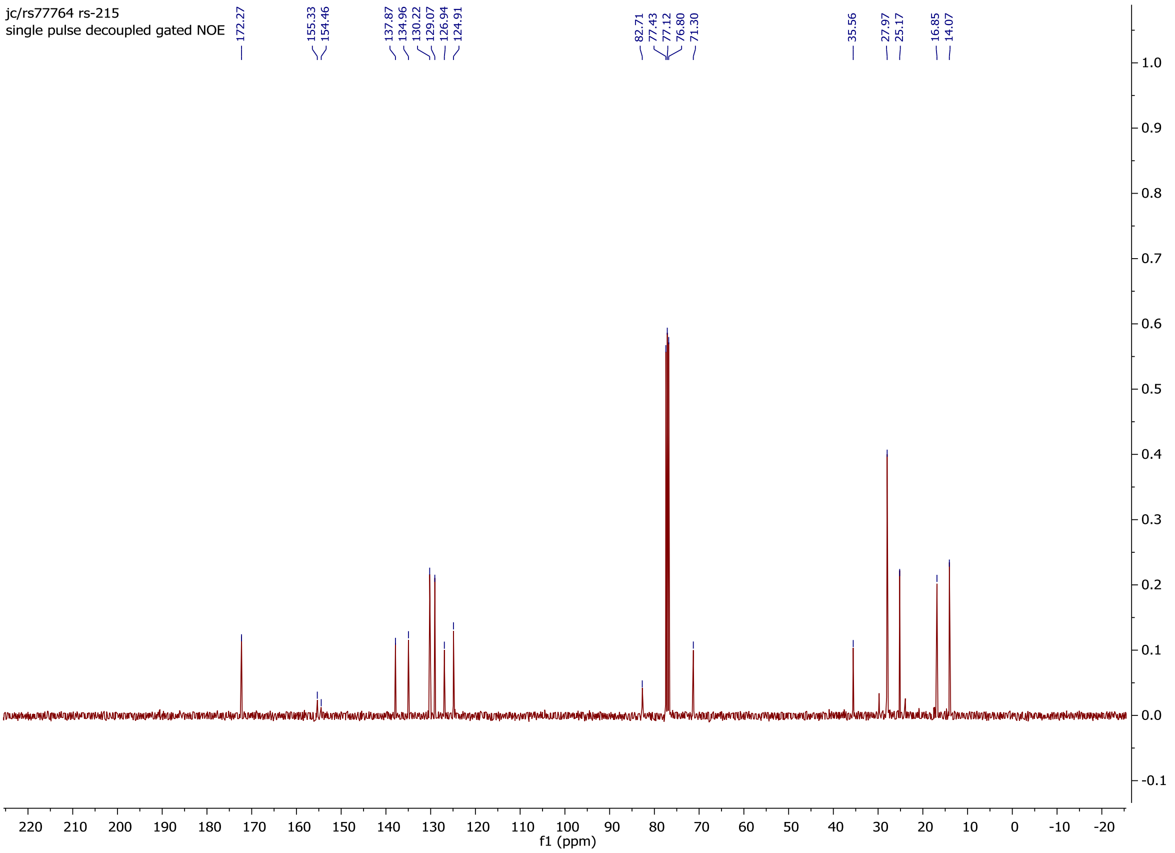

^{1}H NMR

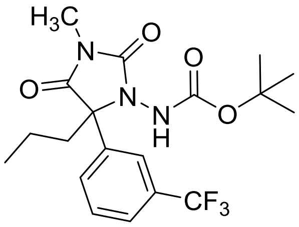

***tert*-Butyl (3-methyl-2,4-dioxo-5-propyl-5-(3-(trifluoromethyl)phenyl)imidazolidin-1-yl)carbamate (7r)**

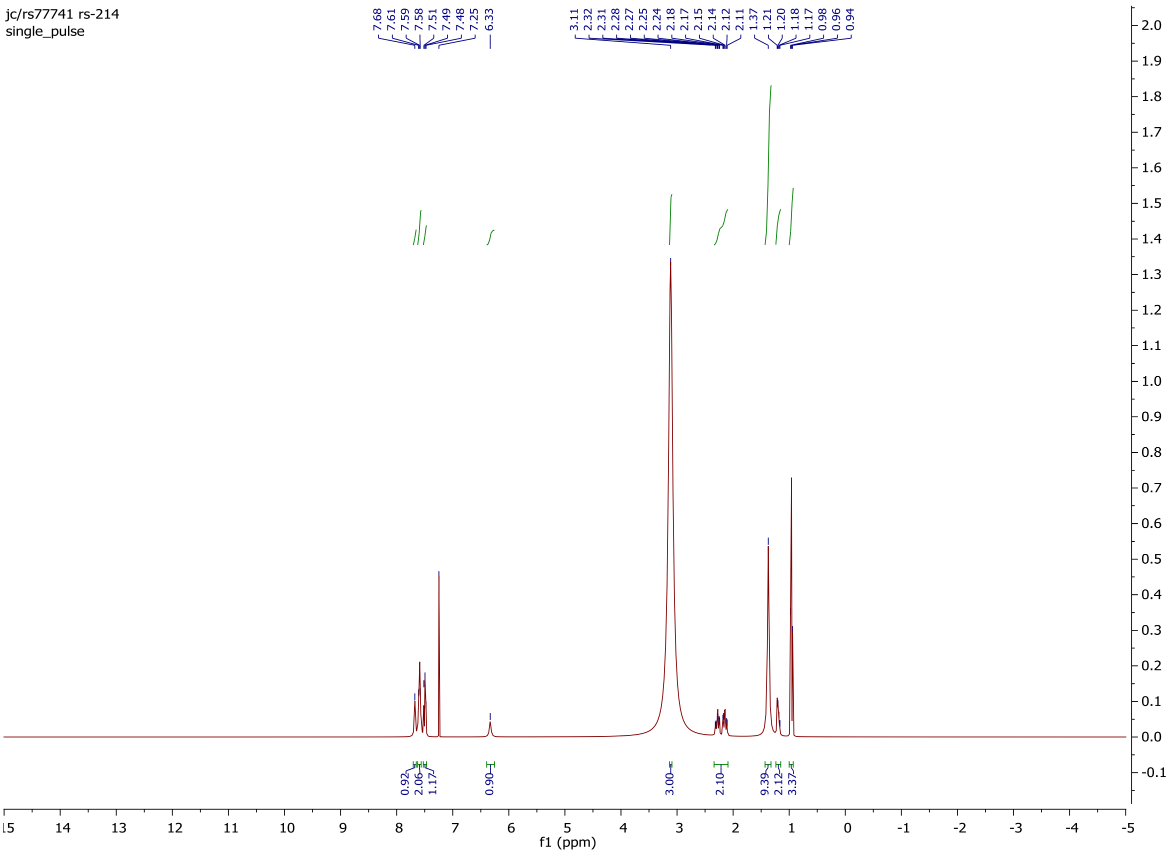

^{13}C NMR

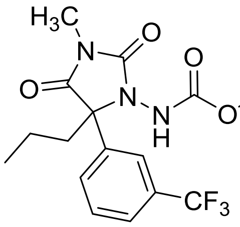

tert-Butyl (3-methyl-2,4-dioxo-5-propyl-5-(3-(trifluoromethyl)phenyl)imidazolidin-1-yl)carbamate (7r)

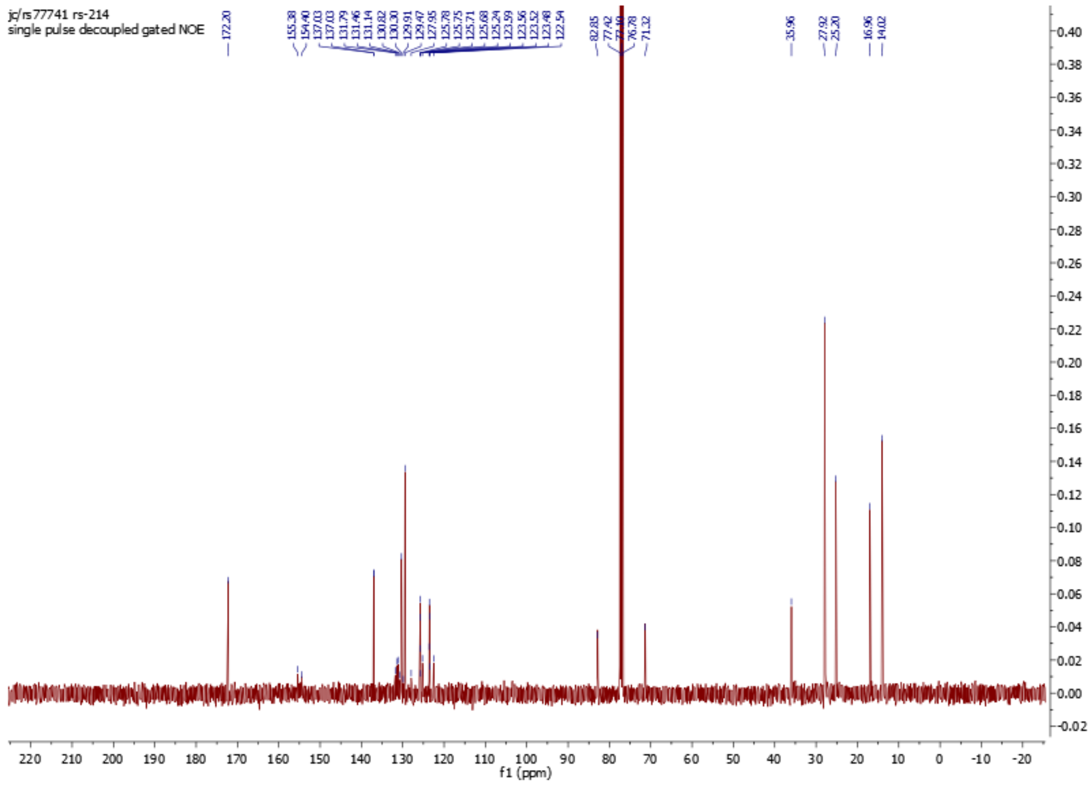

^{1}H NMR

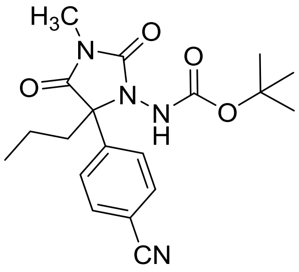

***tert*-Butyl (5-(4-cyanophenyl)-3-methyl-2,4-dioxo-5-propylimidazolidin-1-yl)carbamate**

(7s)

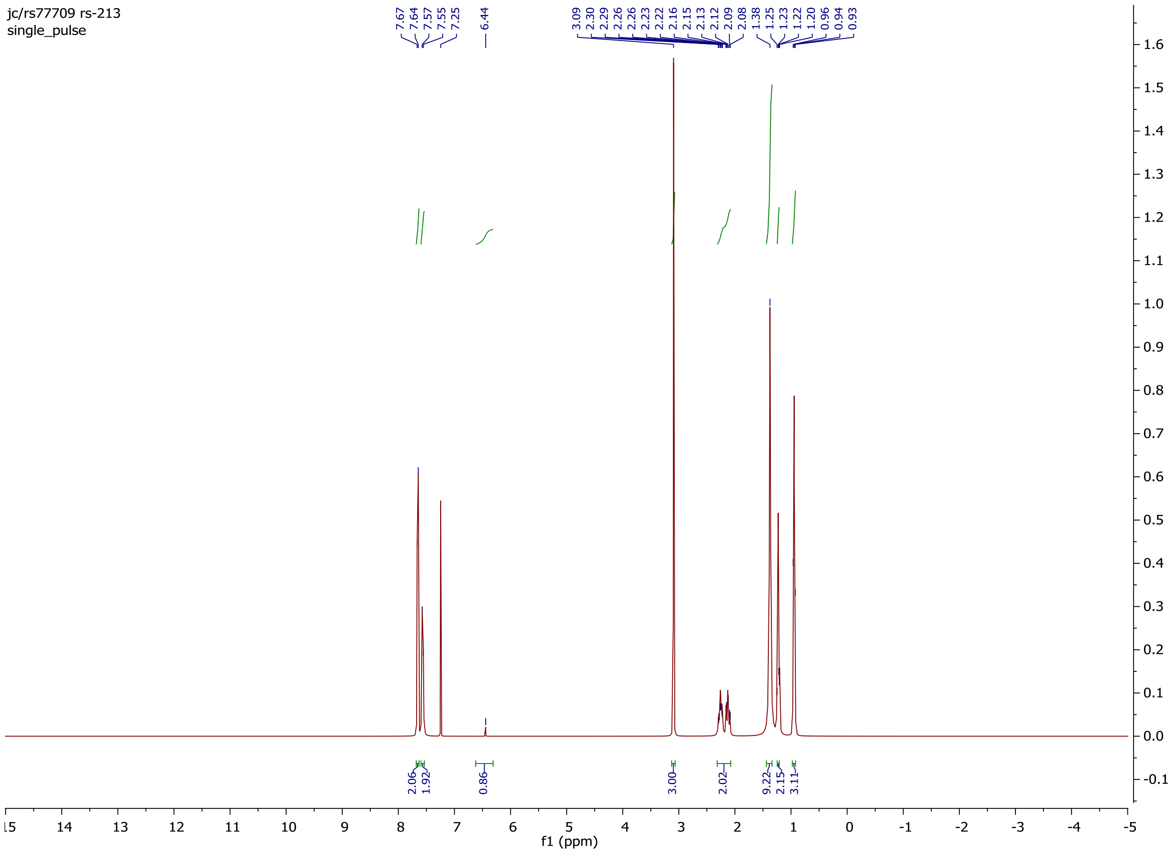

^{13}C NMR

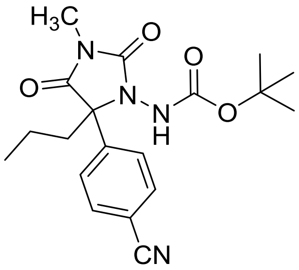

***tert*-Butyl (5-(4-cyanophenyl)-3-methyl-2,4-dioxo-5-propylimidazolidin-1-yl)carbamate**

(7s)

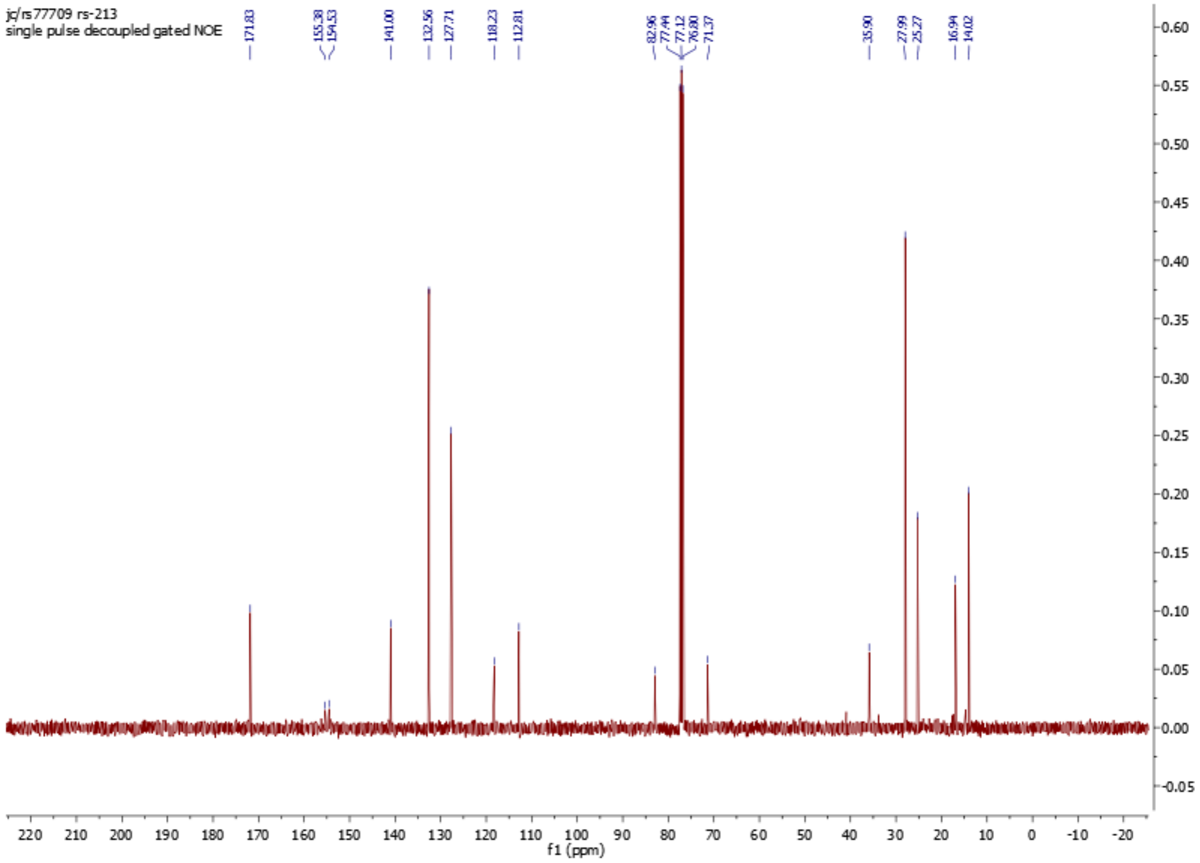

^{1}H NMR

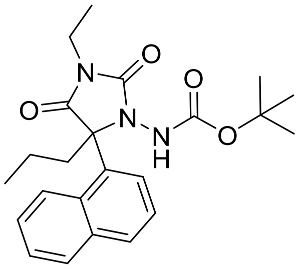

***tert*-Butyl (3-ethyl-5-(naphthalen-1-yl)-2,4-dioxo-5-propylimidazolidin-1-yl)carbamate**

(7t)

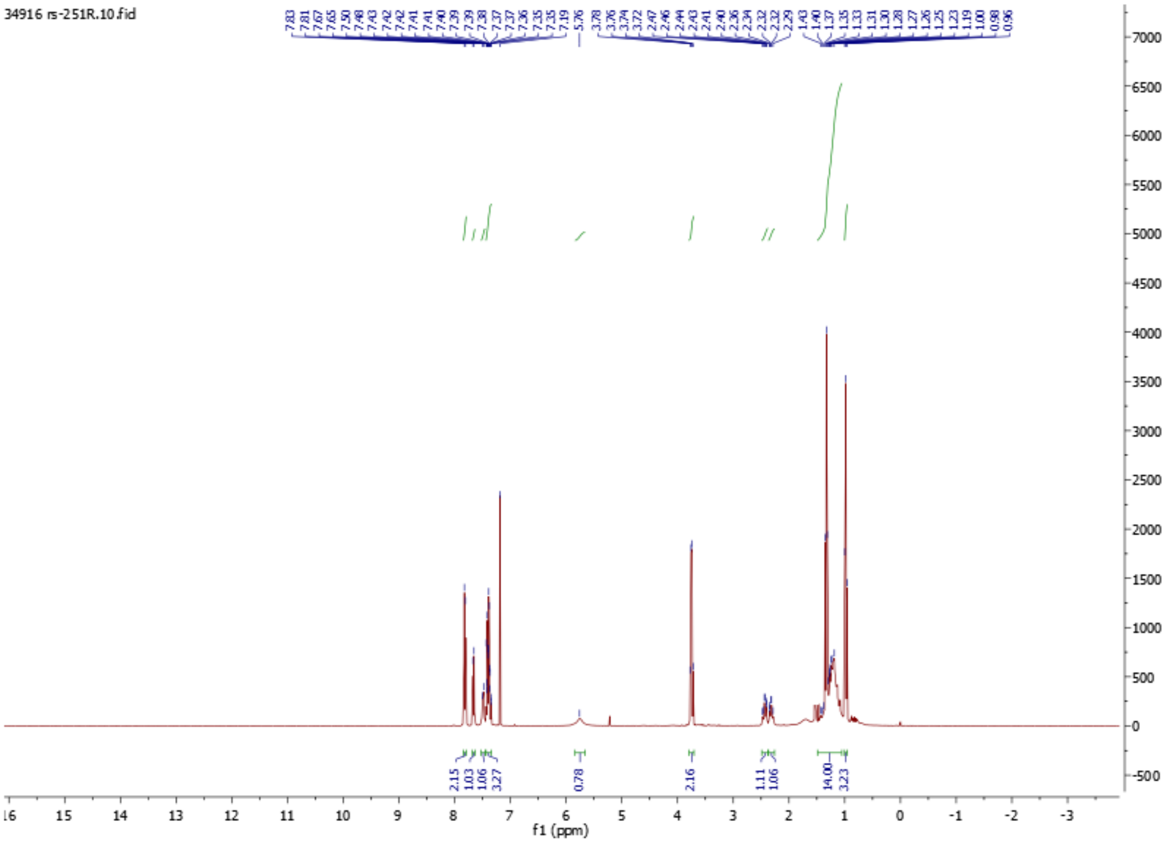

^{13}C NMR

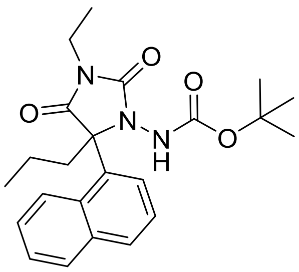

***tert*-Butyl (3-ethyl-5-(naphthalen-1-yl)-2,4-dioxo-5-propylimidazolidin-1-yl)carbamate**

(7t)

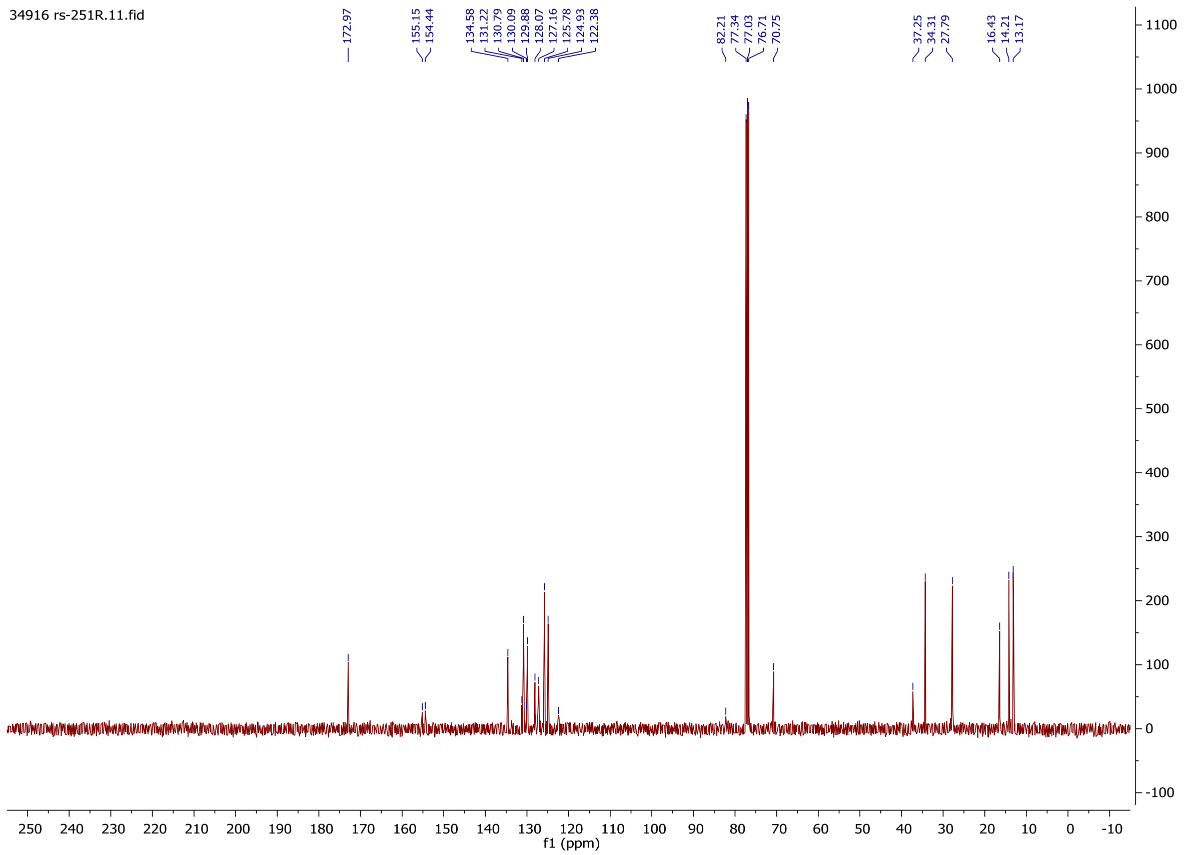

1H NMR

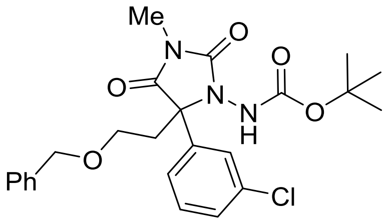

***tert*-Butyl (5-(2-(benzyloxy)ethyl)-5-(3-chlorophenyl)-3-methyl-2,4-dioxoimidazolidin-1-yl)carbamate (7u)**

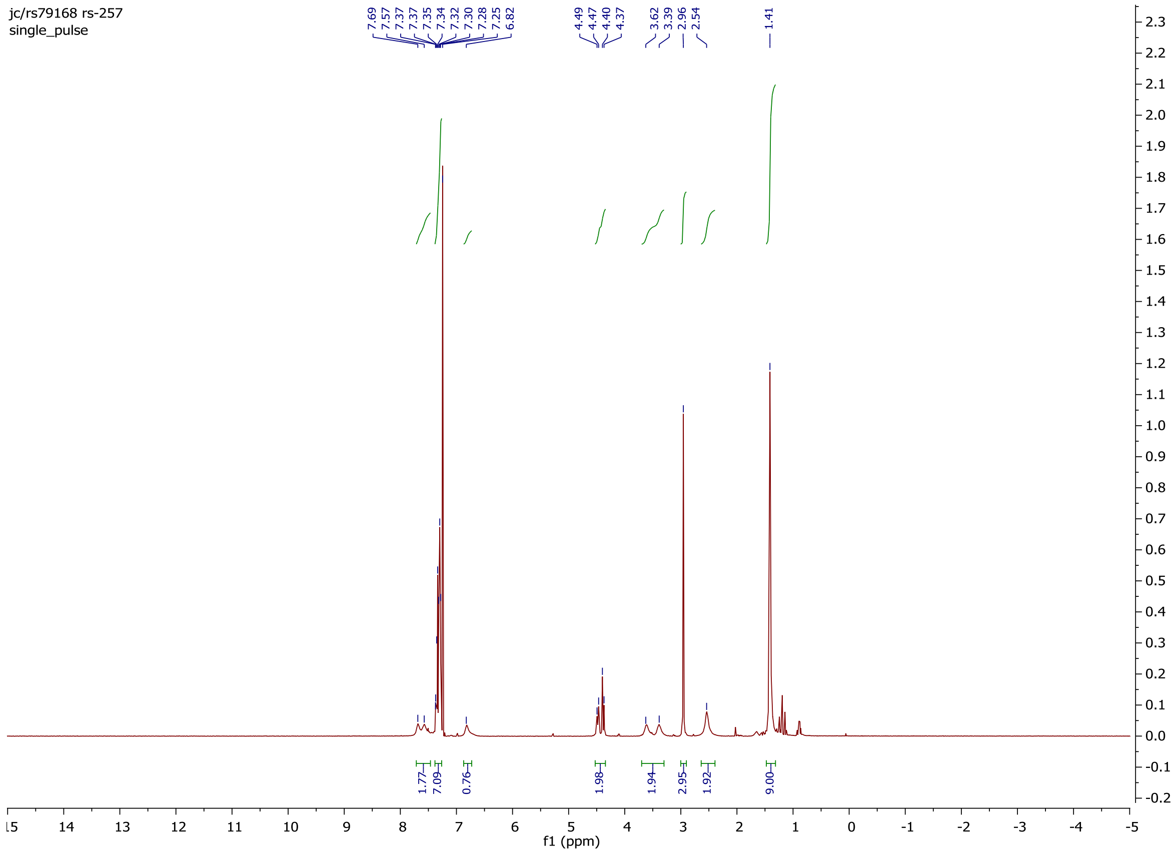

^{13}CNMR

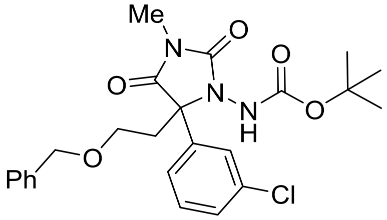

tert-Butyl (5-(2-(benzyloxy)ethyl)-5-(3-chlorophenyl)-3-methyl-2,4-dioxoimidazolidin-1-yl)carbamate (7u)

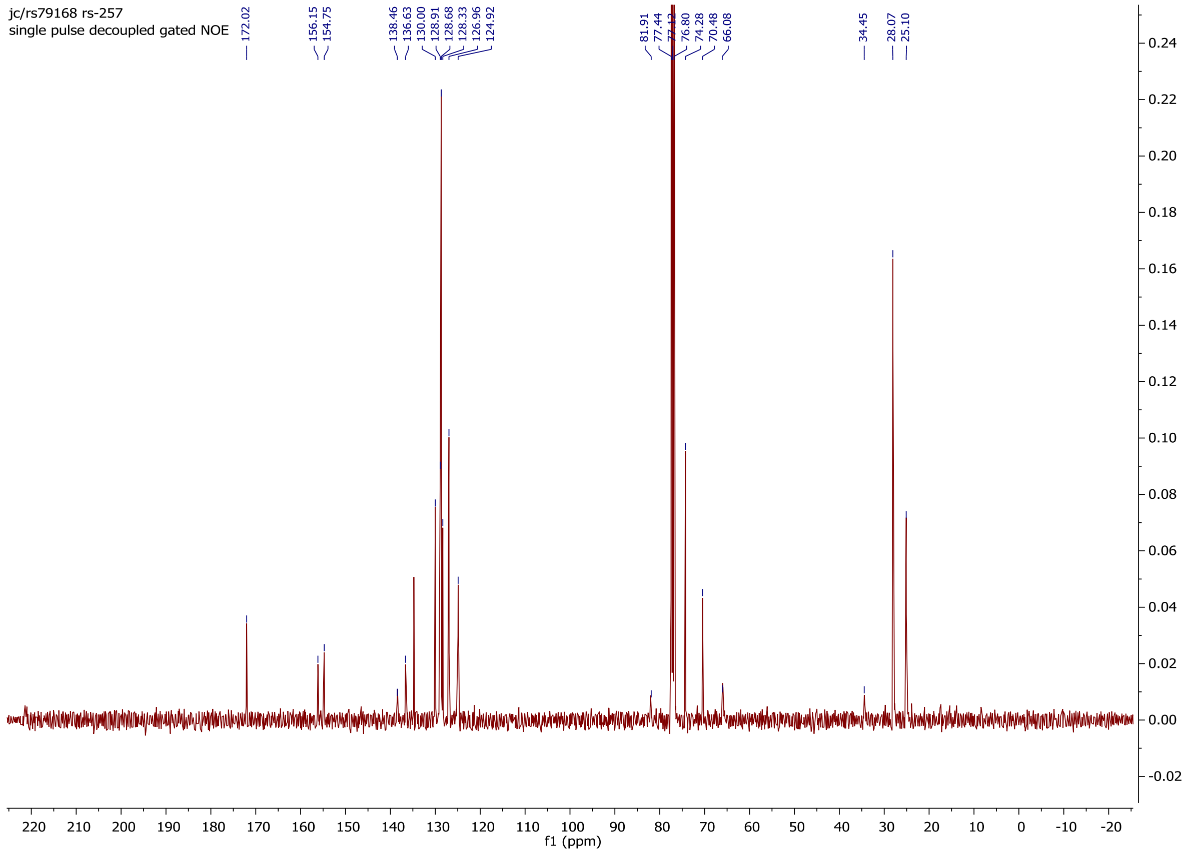

^{1}H NMR

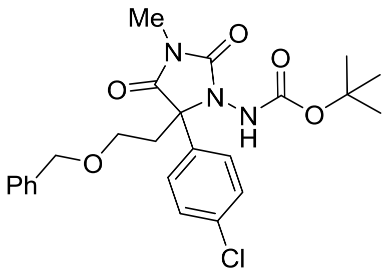

tert-Butyl (5-(2-(benzyloxy)ethyl)-5-(4-chlorophenyl)-3-methyl-2,4-dioxoimidazolidin-1-yl)carbamate (7v)

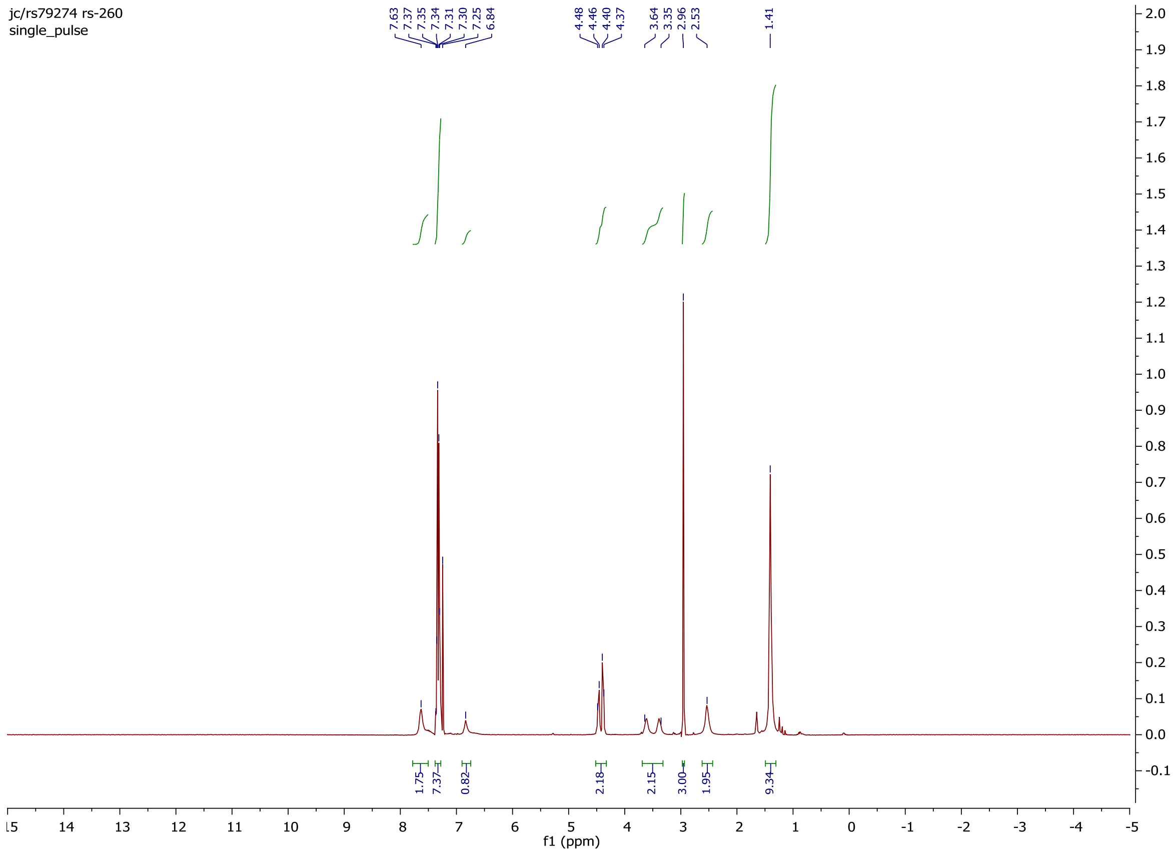

^{13}C NMR

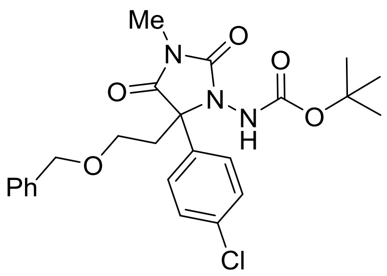

tert-Butyl (5-(2-(benzyloxy)ethyl)-5-(4-chlorophenyl)-3-methyl-2,4-dioxoimidazolidin-1-yl)carbamate (7v)

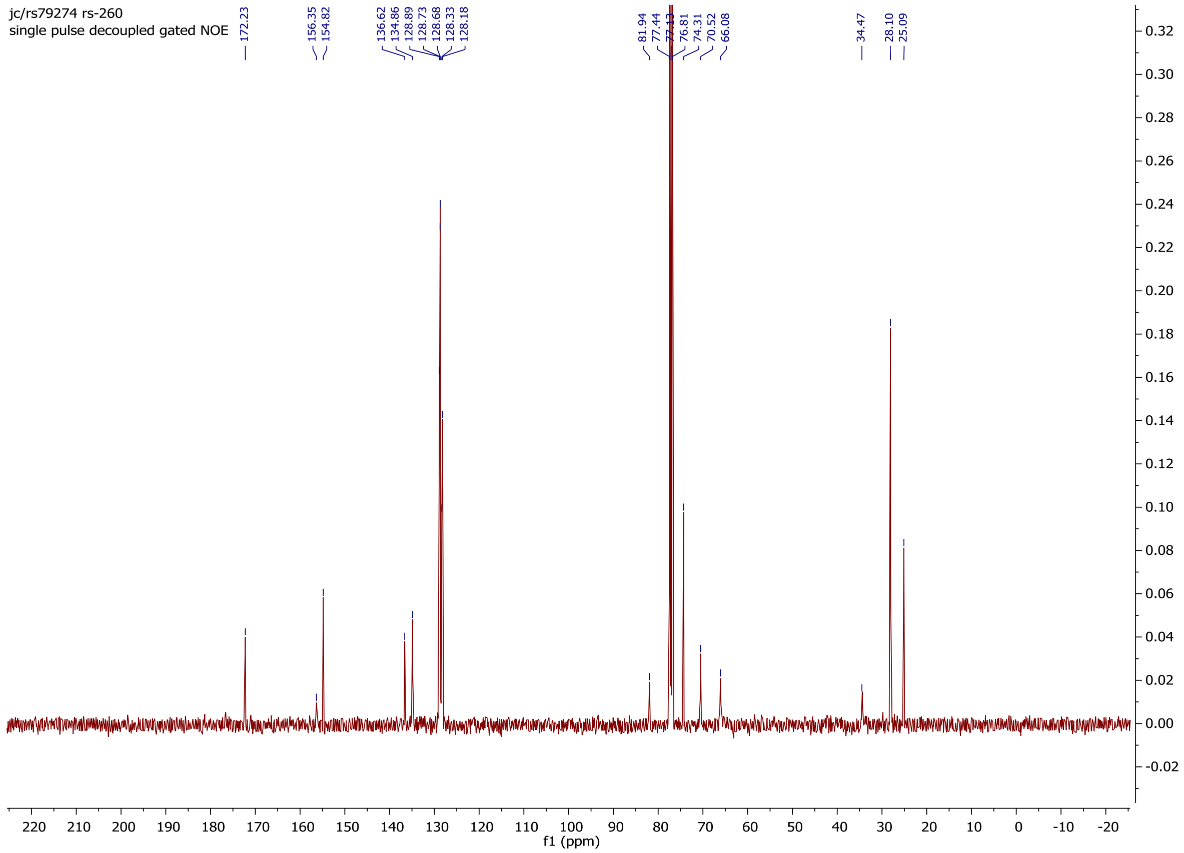

1H NMR

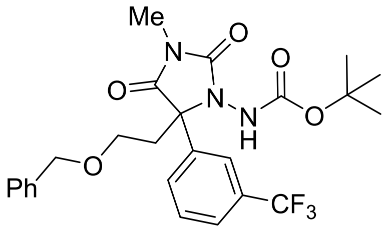

tert-Butyl (5-(2-(benzyloxy)ethyl)-3-methyl-2,4-dioxo-5-(3-(trifluoromethyl)phenyl)imidazolidin-1-yl)carbamate (7w)

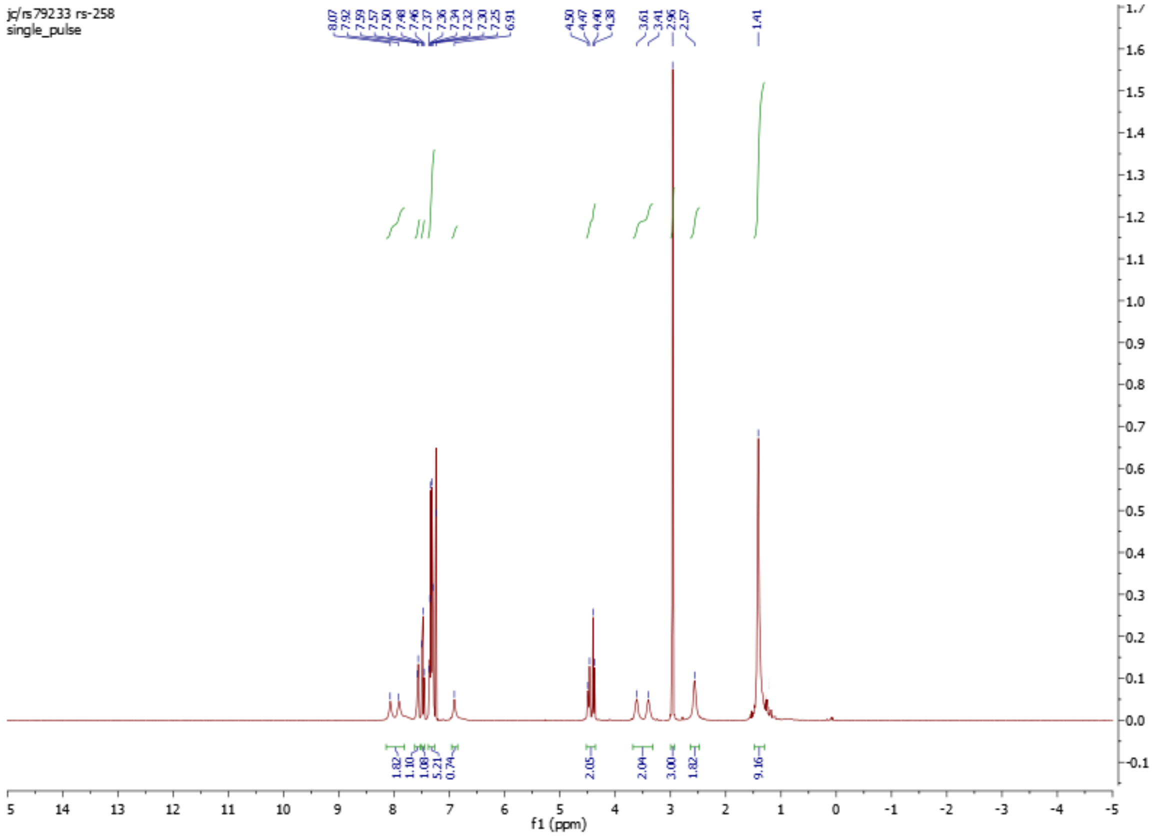

^{13}C NMR

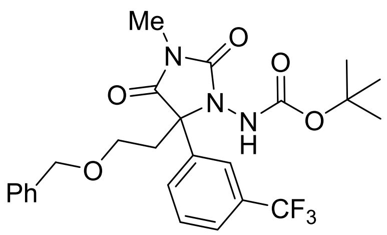

tert-Butyl (5-(2-(benzyloxy)ethyl)-3-methyl-2,4-dioxo-5-(3-(trifluoromethyl)phenyl)imidazolidin-1-yl)carbamate (7w)

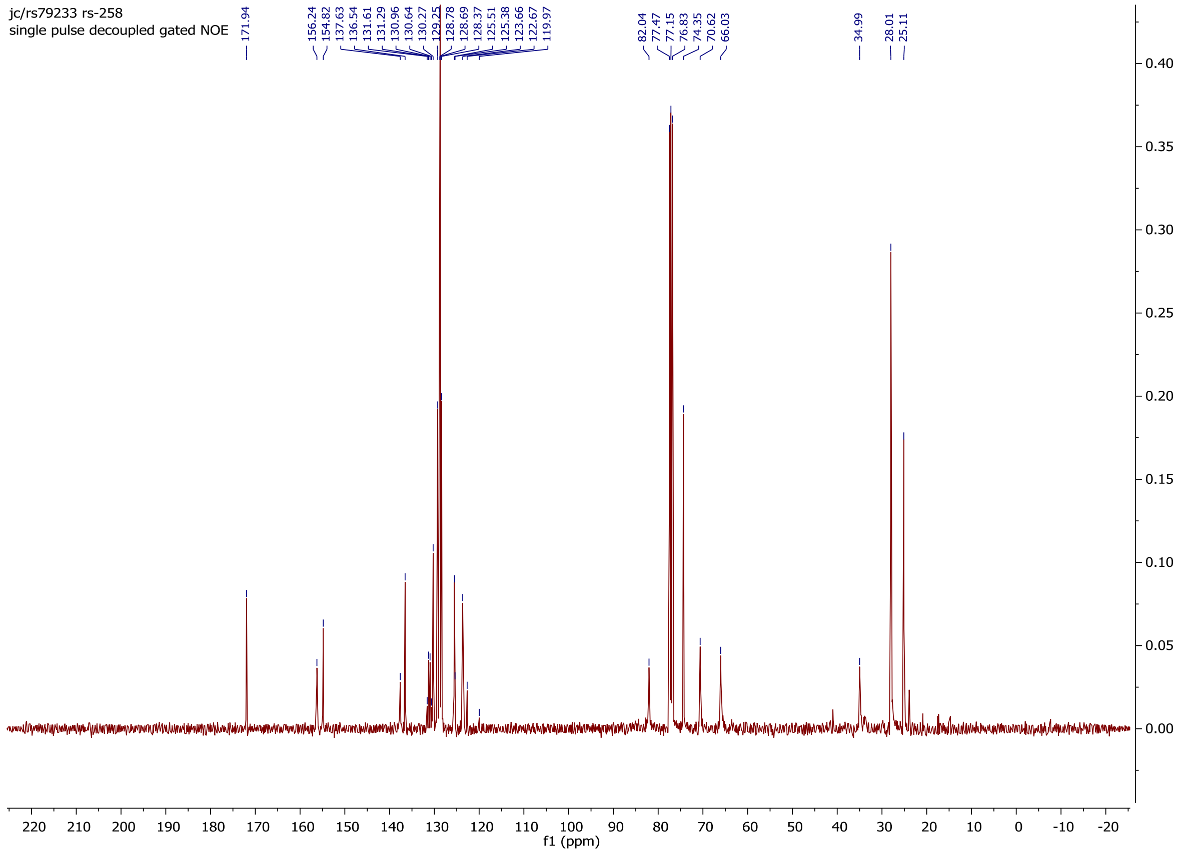

^{1}H NMR

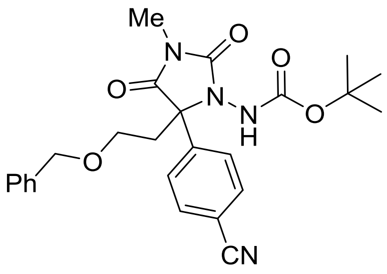

tert-Butyl (5-(2-(benzyloxy)ethyl)-5-(4-cyanophenyl)-3-methyl-2,4-dioxoimidazolidin-1-yl)carbamate (7x)

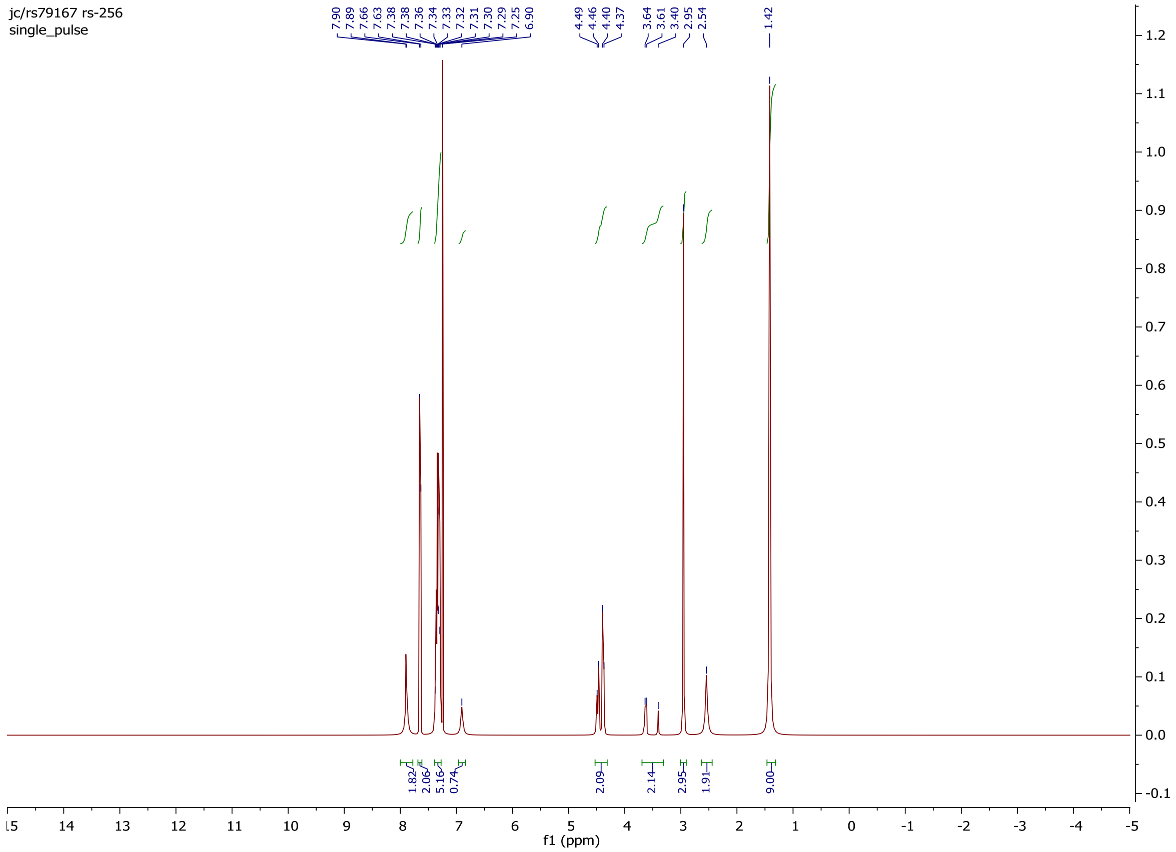

^{13}C NMR

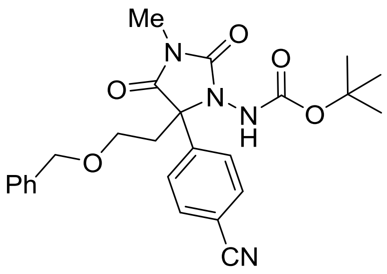

***tert*-Butyl (5-(2-(benzyloxy)ethyl)-5-(4-cyanophenyl)-3-methyl-2,4-dioxoimidazolidin-1-yl)carbamate (7x)**

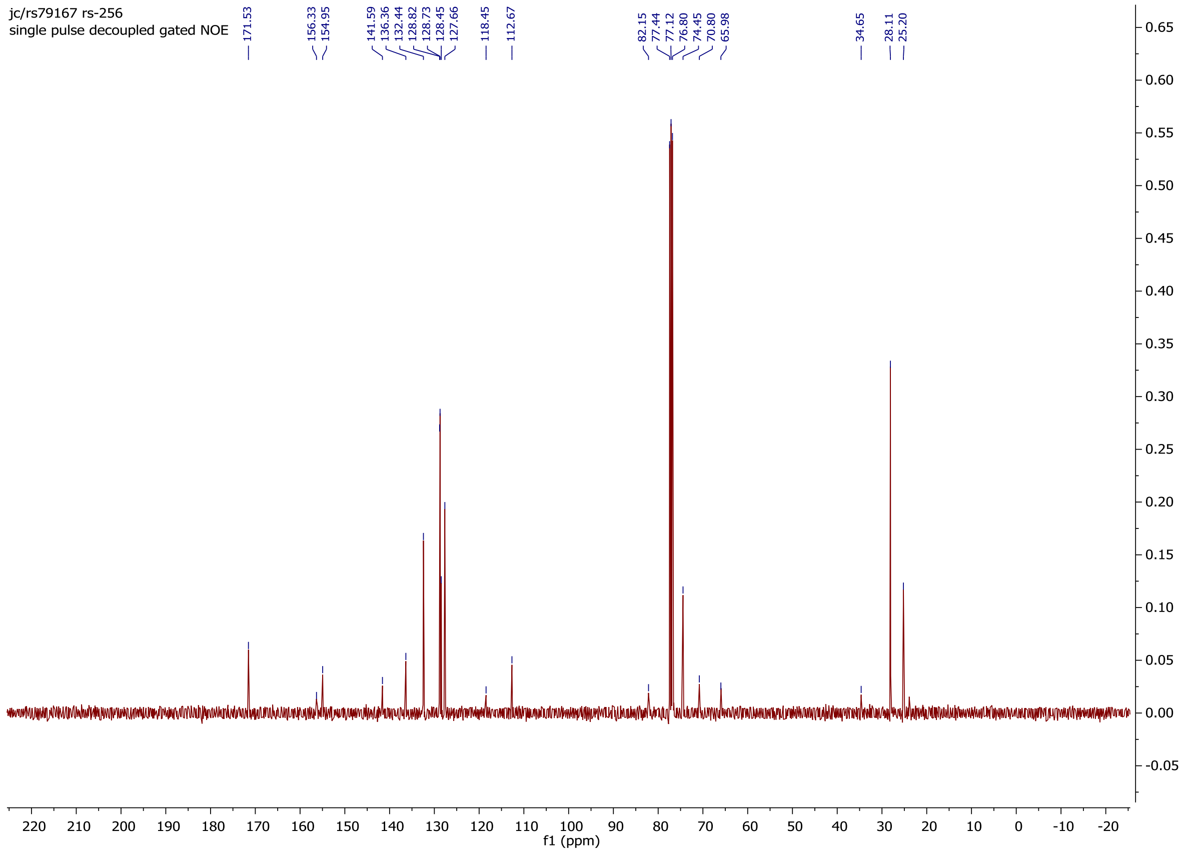

^{1}H NMR

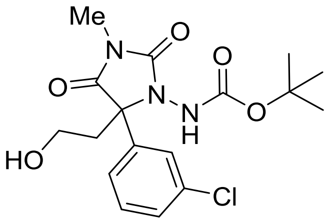

***tert*-Butyl (5-(3-chlorophenyl)-5-(2-hydroxyethyl)-3-methyl-2,4-dioxoimidazolidin-1-yl)carbamate (7y)**

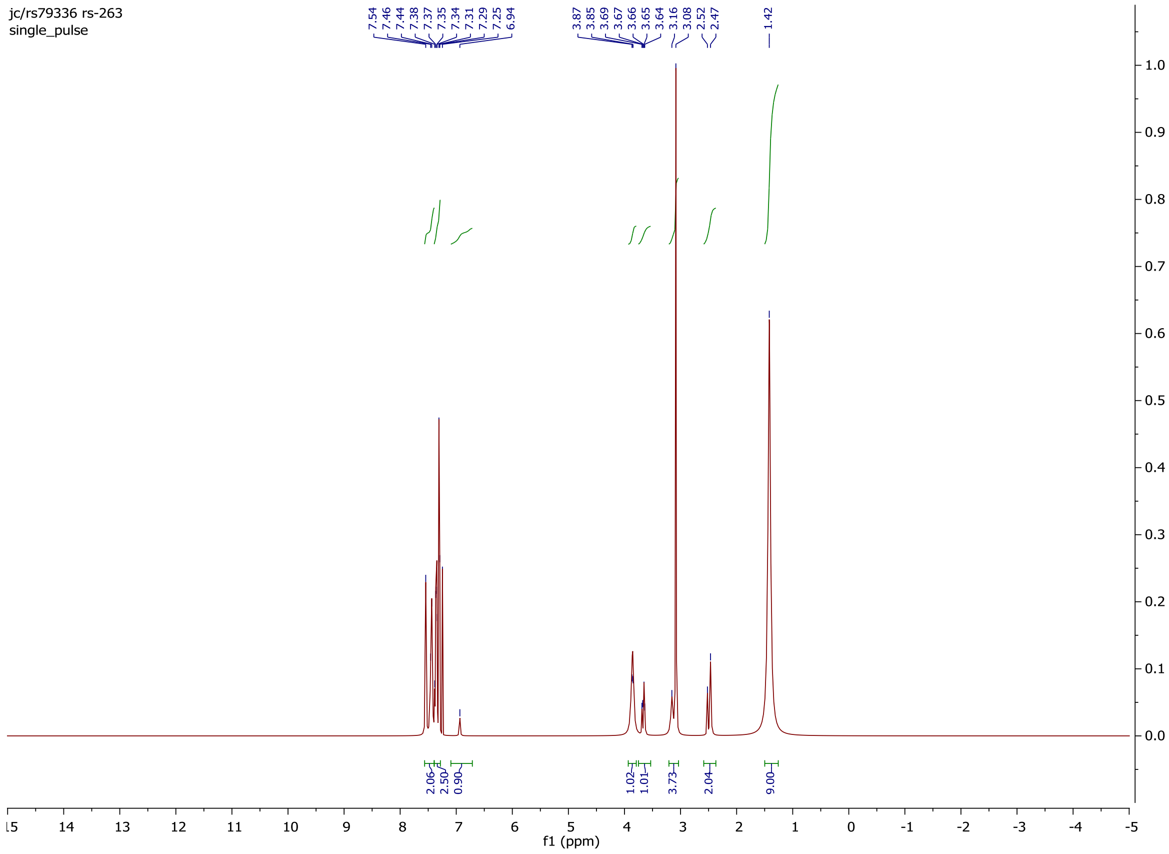

^{13}C NMR

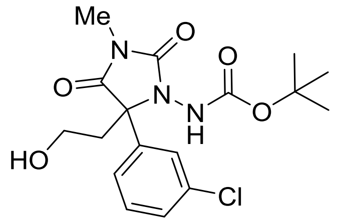

***tert*-Butyl (5-(3-chlorophenyl)-5-(2-hydroxyethyl)-3-methyl-2,4-dioxoimidazolidin-1-yl)carbamate (7y)**

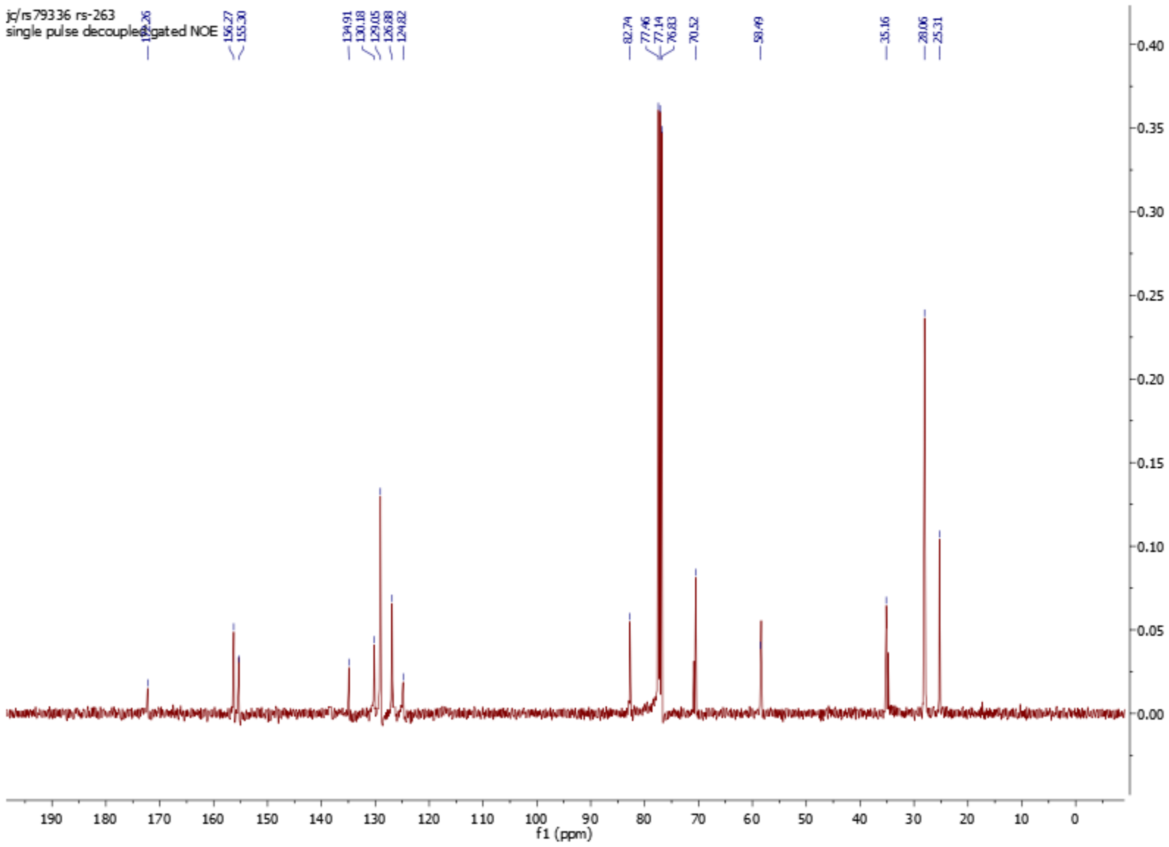

^{1}H NMR

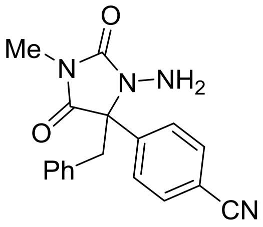

4-(3-Amino-4-benzyl-1-methyl-2,5-dioxoimidazolidin-4-yl)benzonitrile (8a)

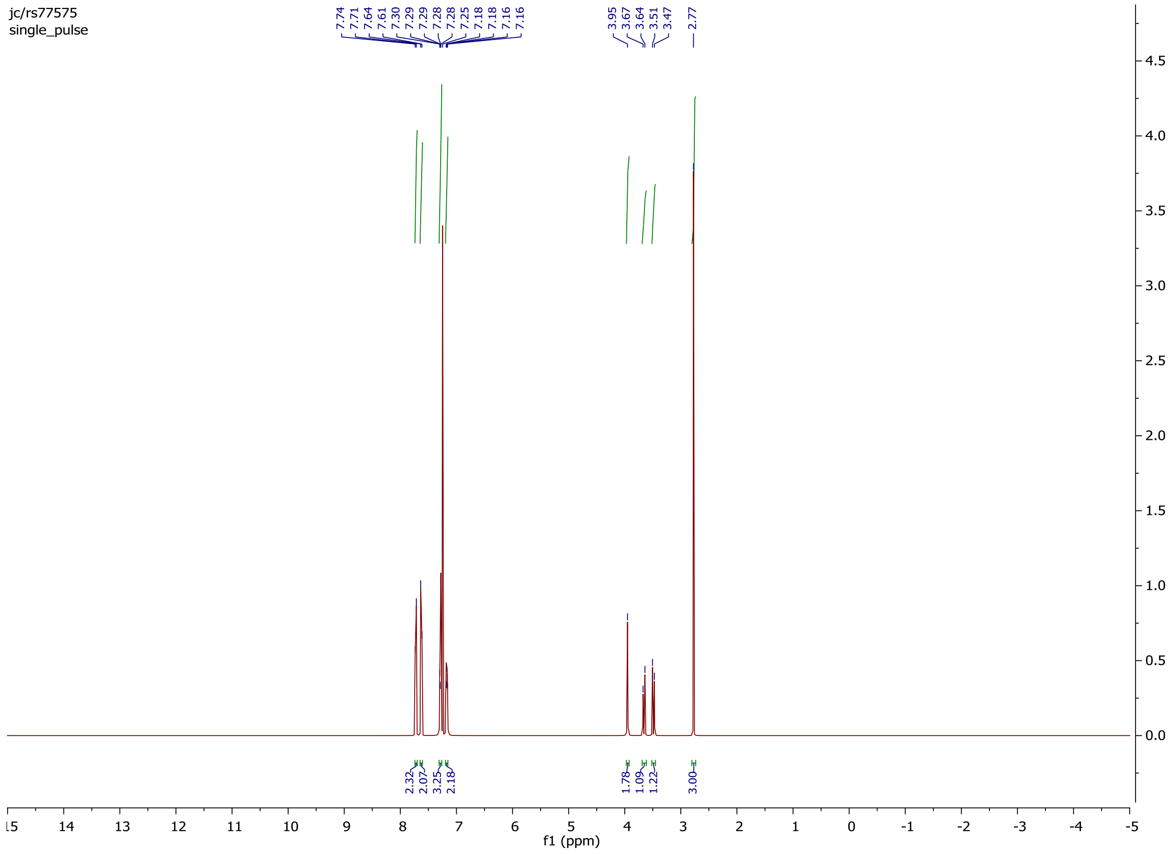

^{13}C NMR

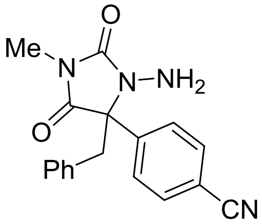

4-(3-Amino-4-benzyl-1-methyl-2,5-dioxoimidazolidin-4-yl)benzonitrile (8a)

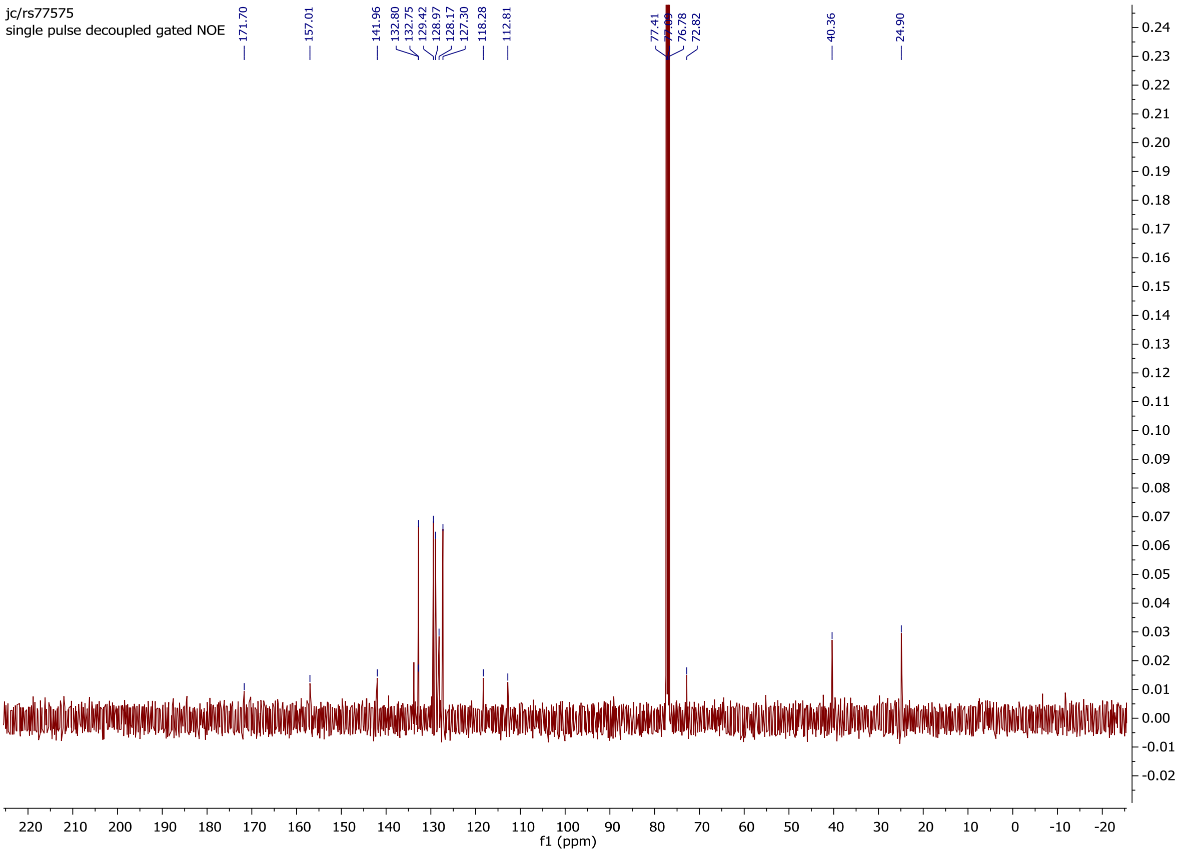

^{1}H NMR

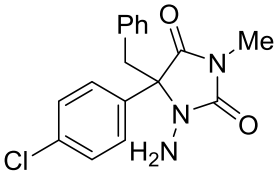

1-Amino-5-benzyl-5-(4-chlorophenyl)-3-methylimidazolidine-2,4-dione (8b)

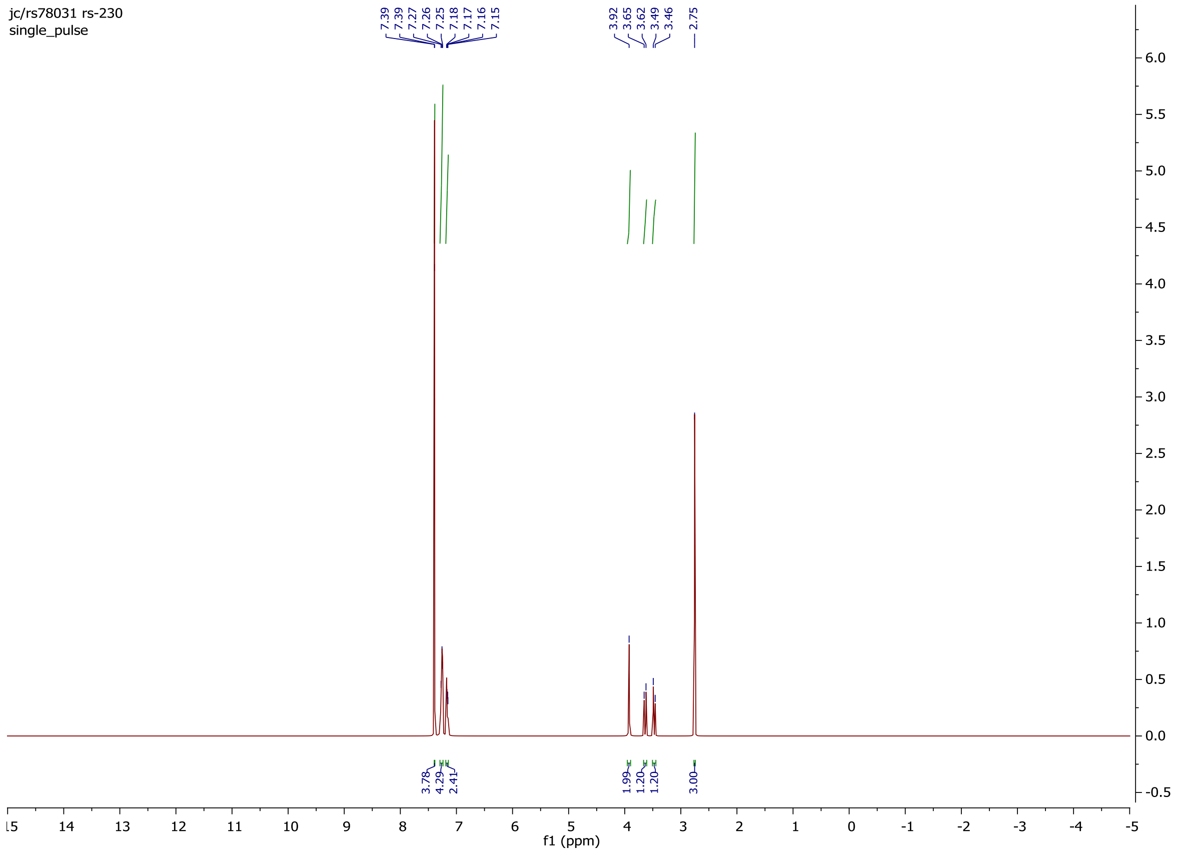

^{13}C NMR

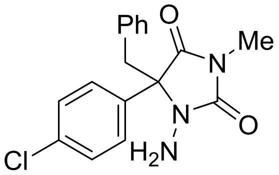

1-Amino-5-benzyl-5-(4-chlorophenyl)-3-methylimidazolidine-2,4-dione (8b)

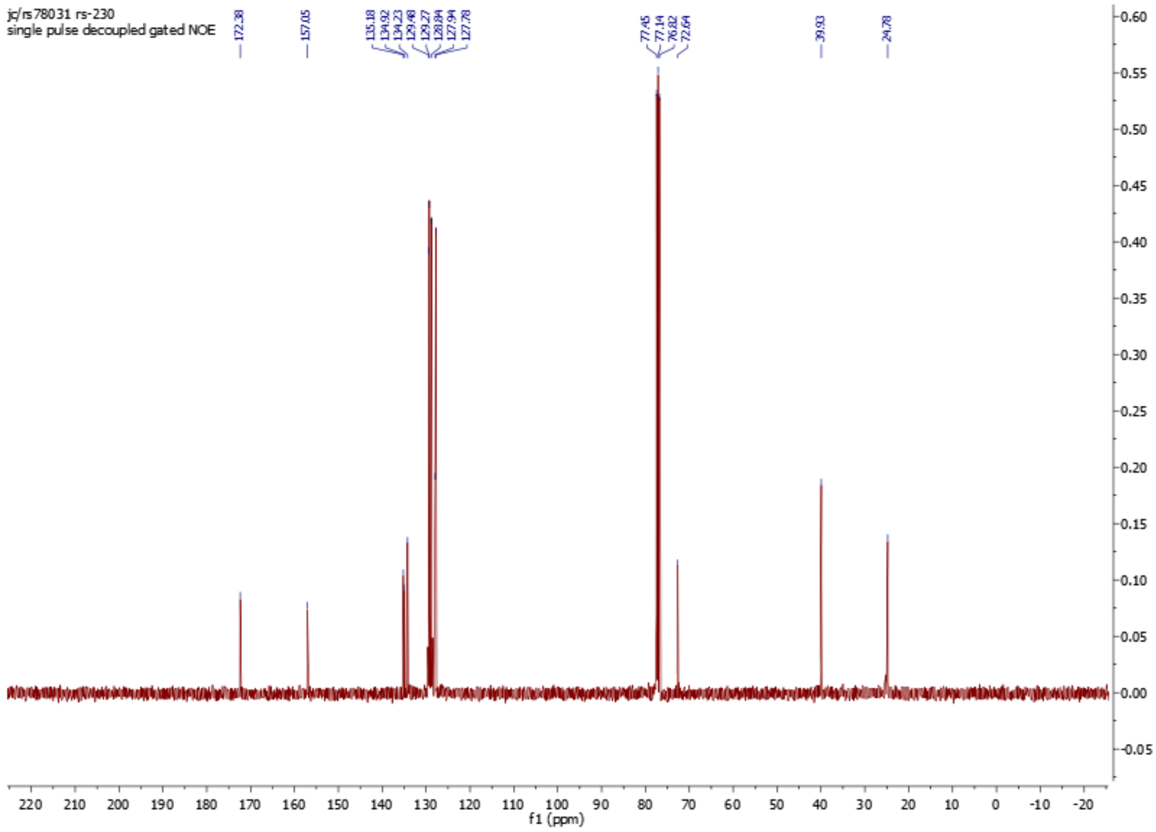

^{1}H NMR

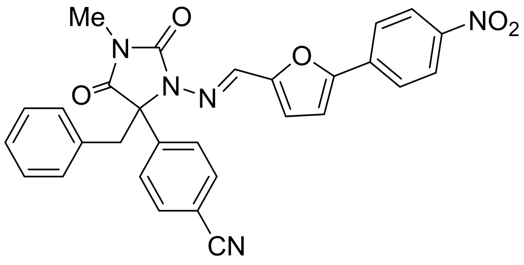

(*E*)-4-(4-Benzyl-1-methyl-3-(((5-(4-nitrophenyl)furan-2-yl)methylene)amino)-2,5-dioxoimidazolidin-4-yl)benzonitrile (9a)

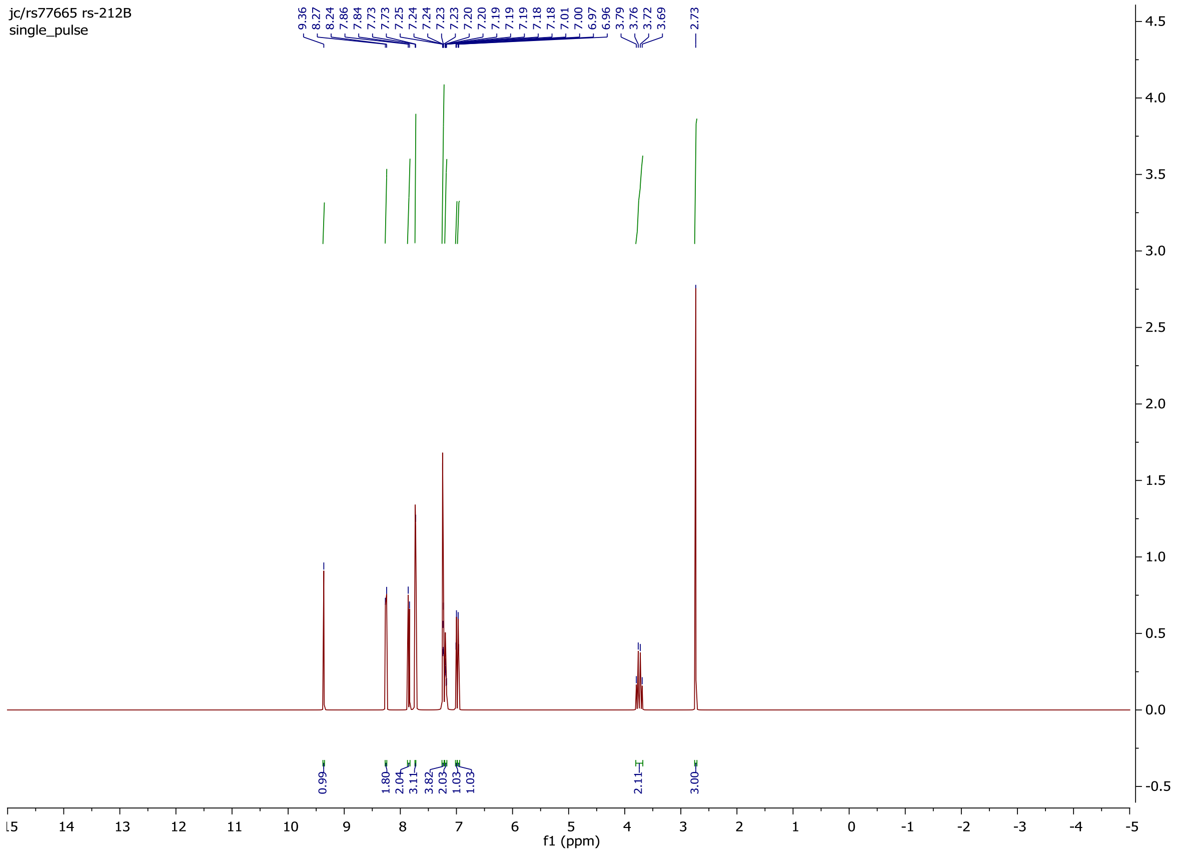

^{13}C NMR

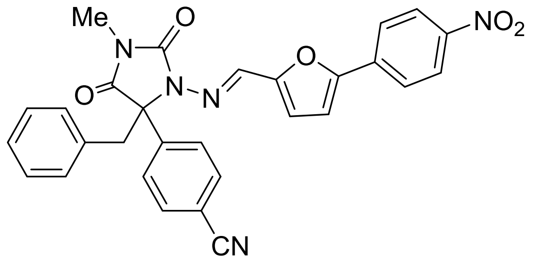

(*E*)-4-(4-Benzyl-1-methyl-3-(((5-(4-nitrophenyl)furan-2-yl)methylene)amino)-2,5-dioxoimidazolidin-4-yl)benzonitrile (9a)

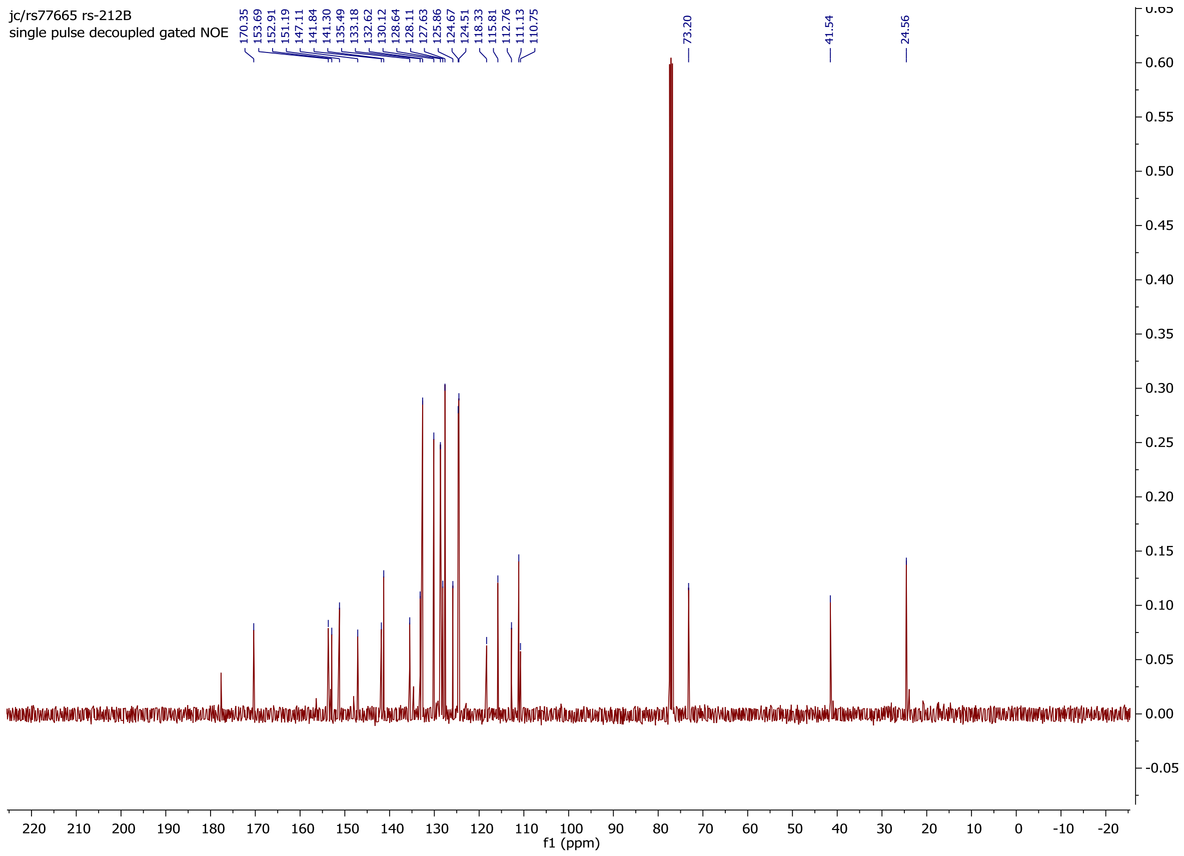

^{1}H NMR

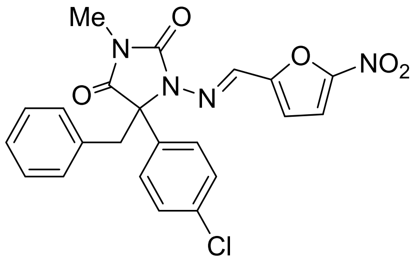

(*E*)-5-Benzyl-5-(4-chlorophenyl)-3-methyl-1-(((5-nitrofuran-2-yl)methylene)amino) imidazolidine-2,4-dione (9b)

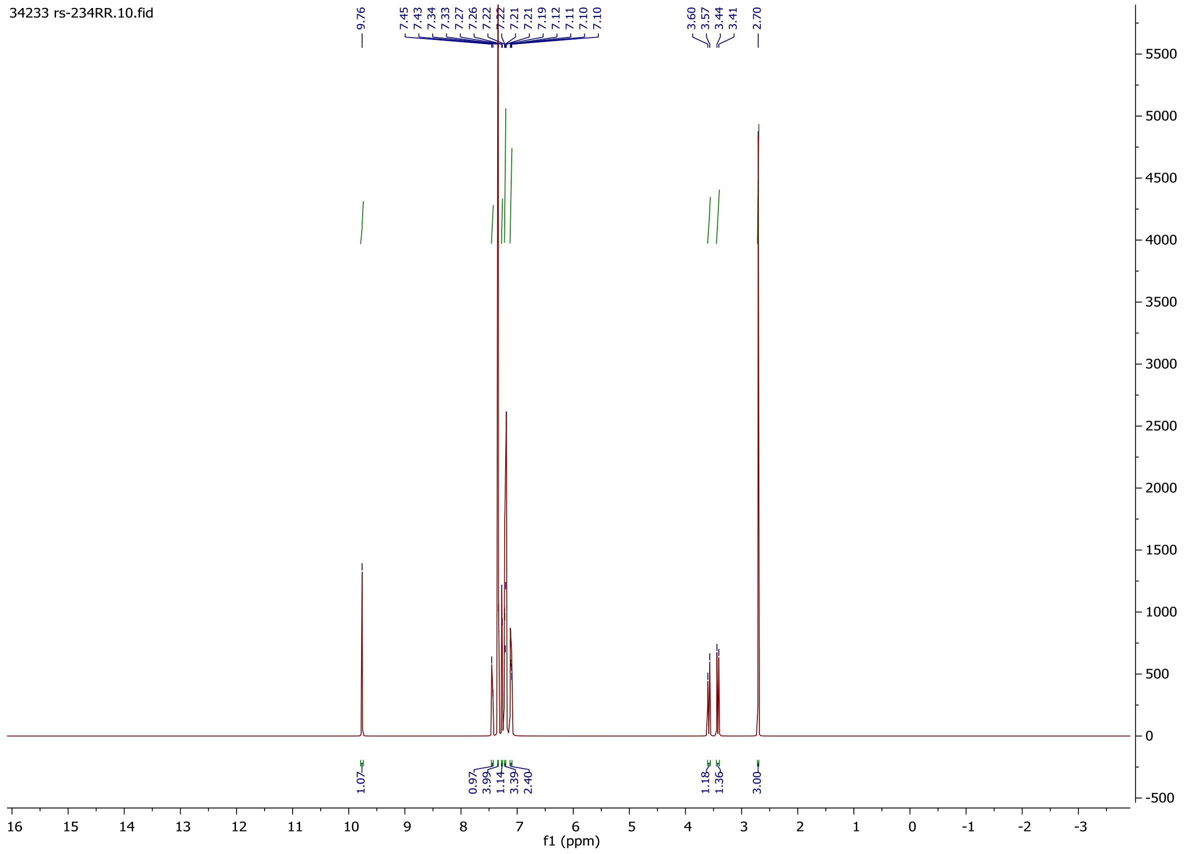

^{13}C NMR

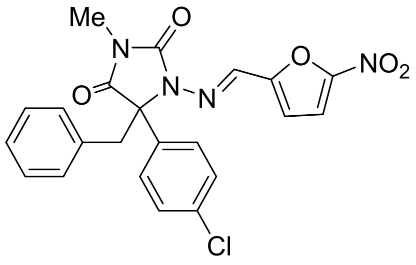

(*E*)-5-Benzyl-5-(4-chlorophenyl)-3-methyl-1-(((5-nitrofuran-2-yl)methylene)amino) imidazolidine-2,4-dione (9b)

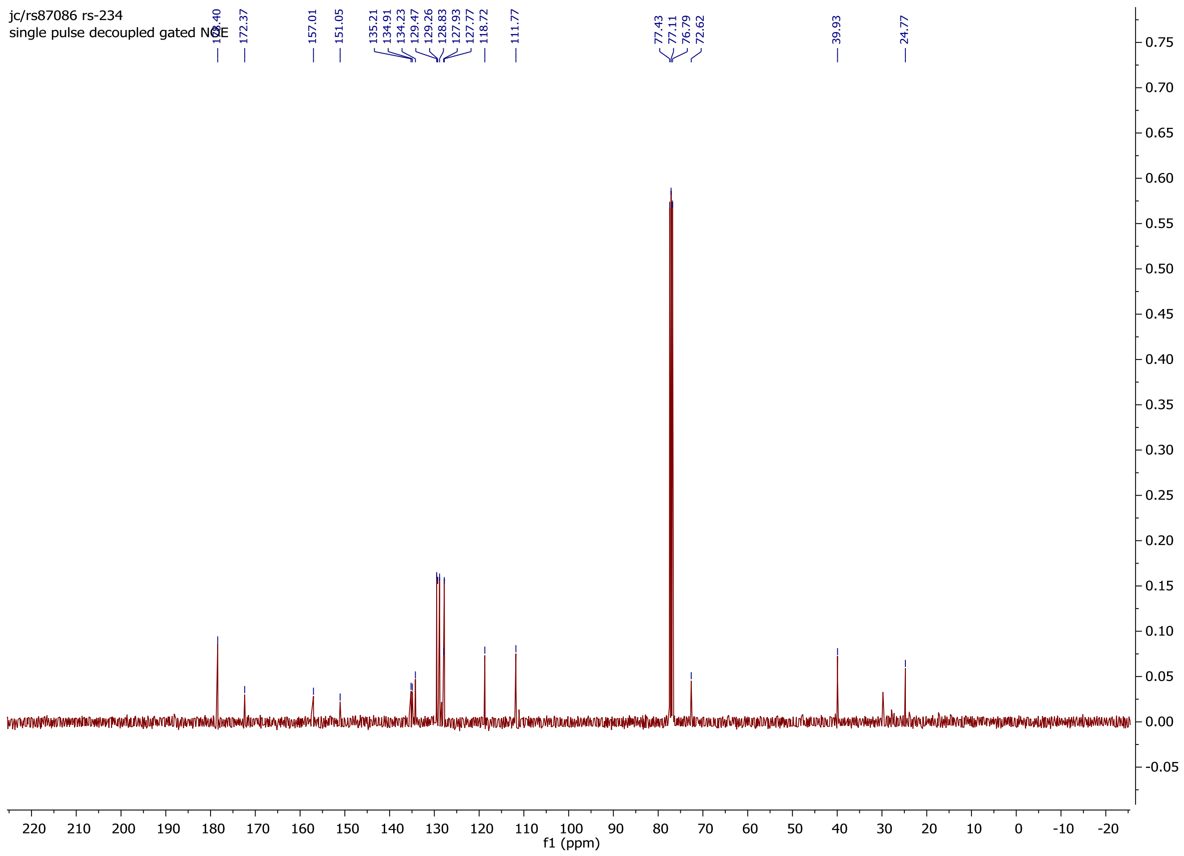

^{1}H NMR of 10a

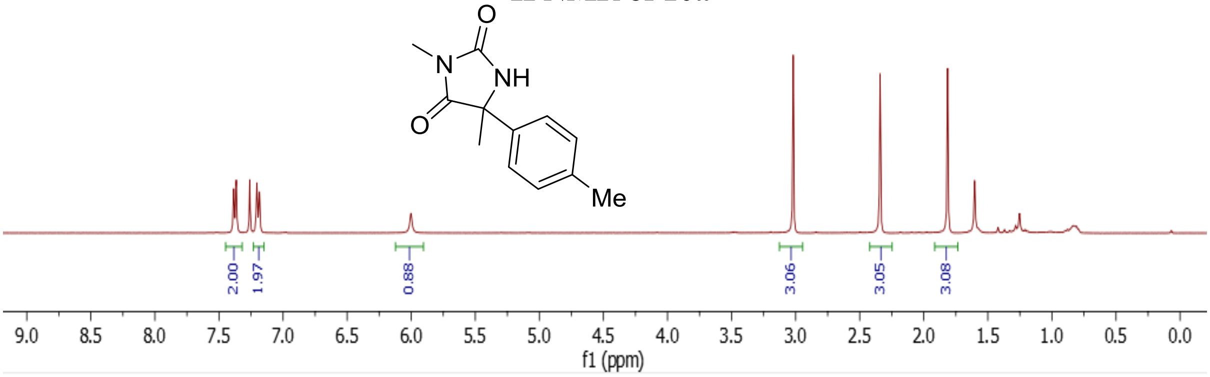

^{13}C NMR of 10a

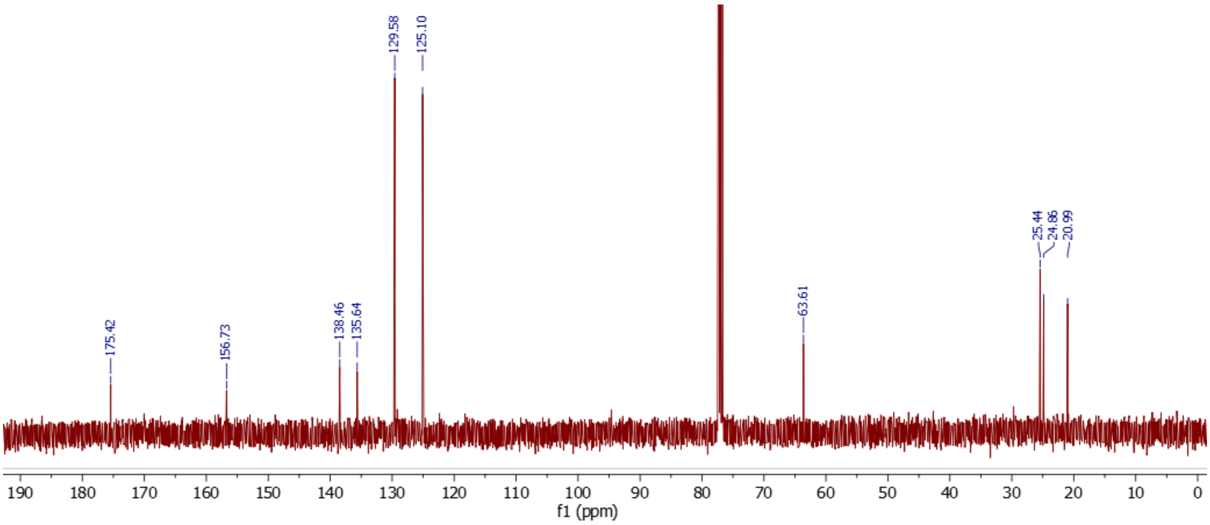

HSQC of 10a

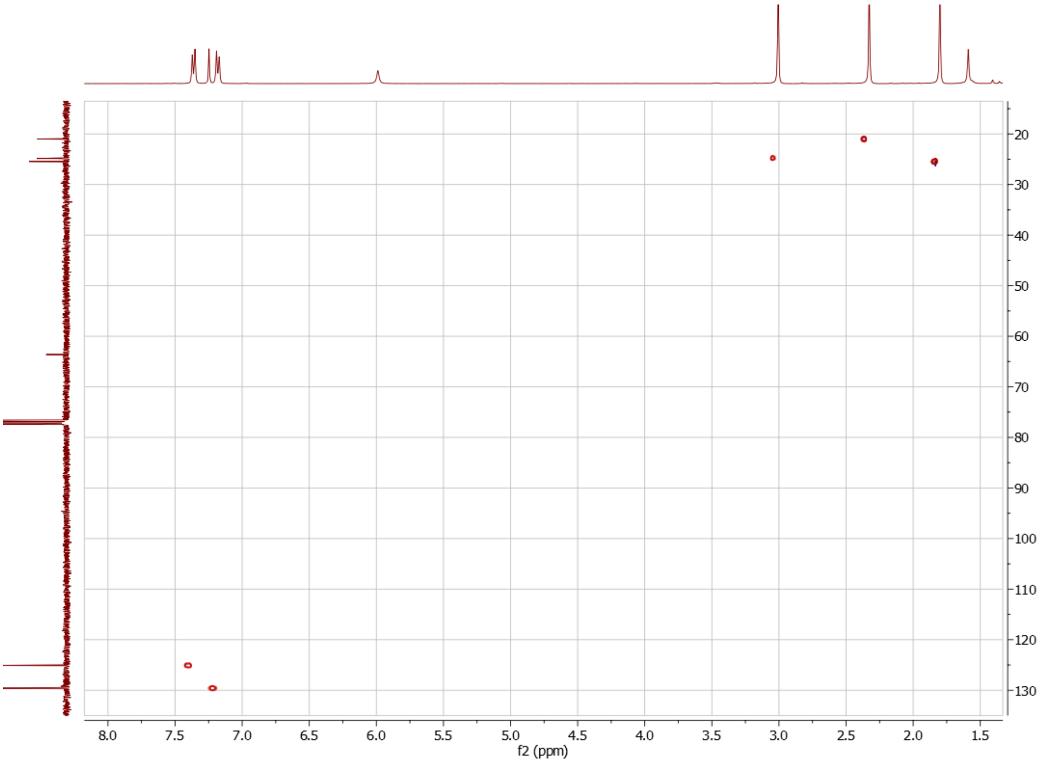

^{1}H NMR of 10b

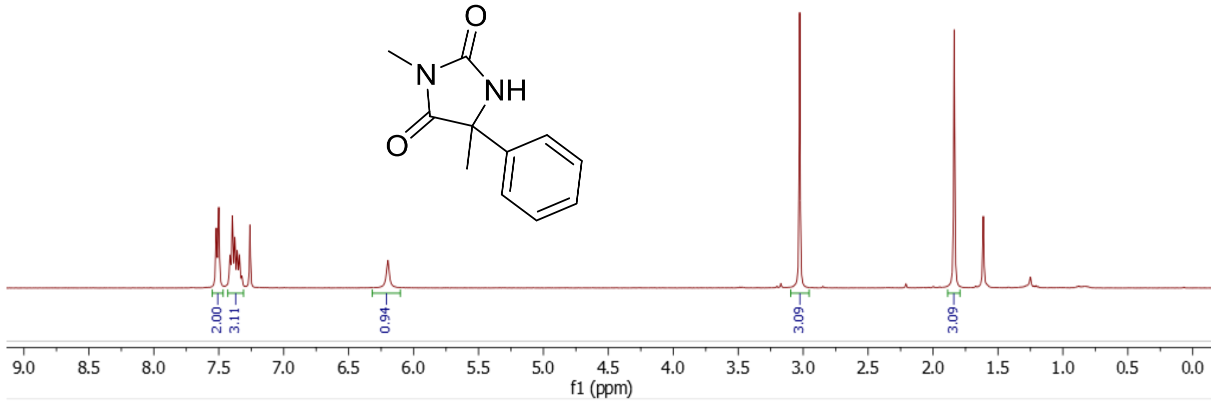

^{13}C NMR of 10b

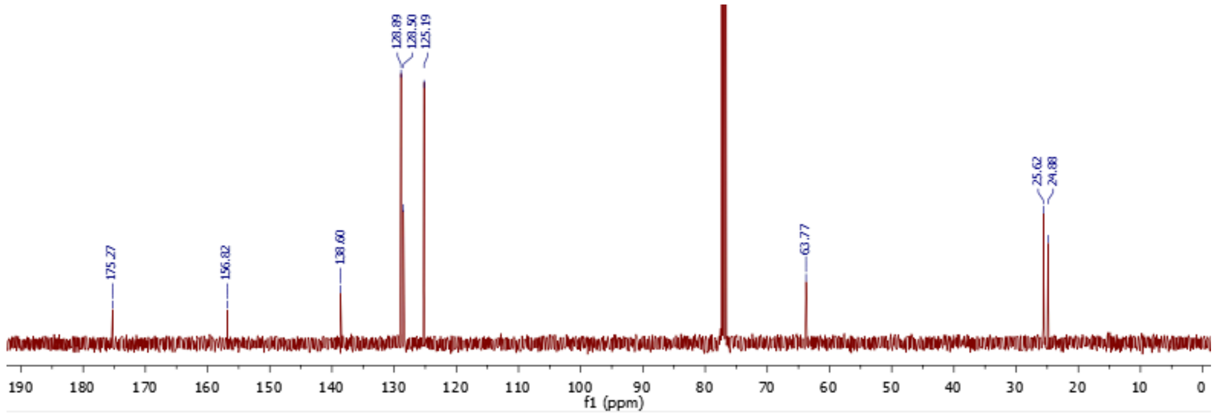

HSQC of 10b

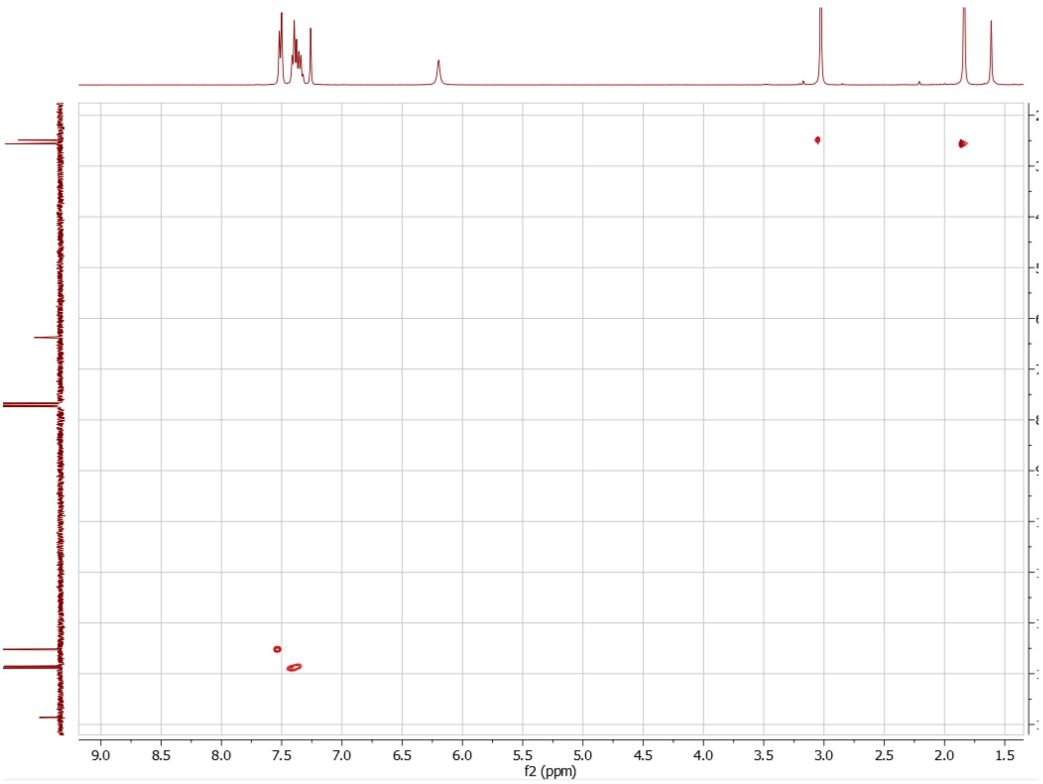

^{1}H NMR of 10c

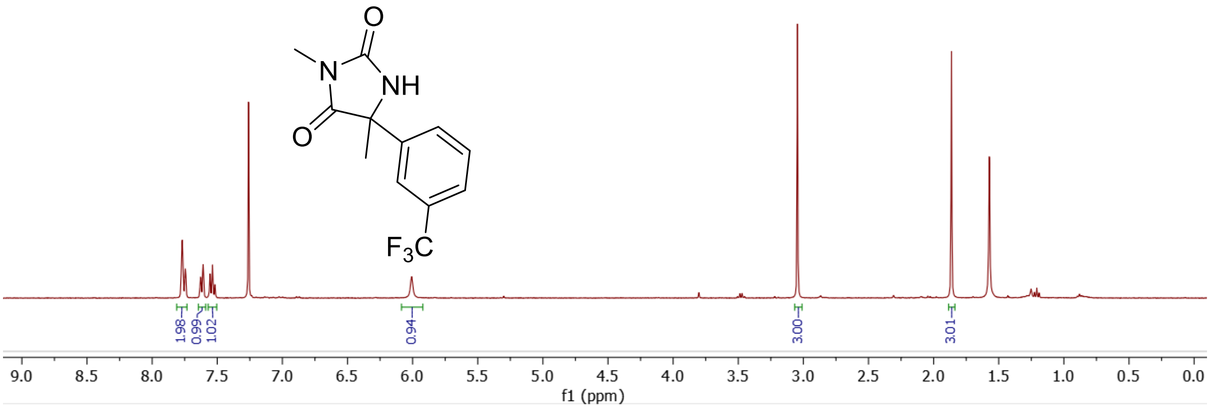

^{13}C NMR of 10c

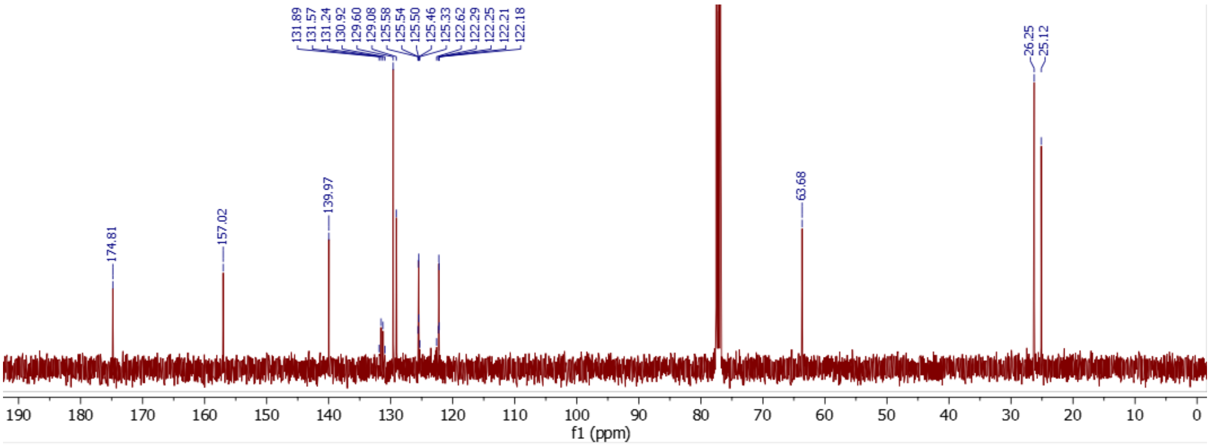

HSQC of 10c

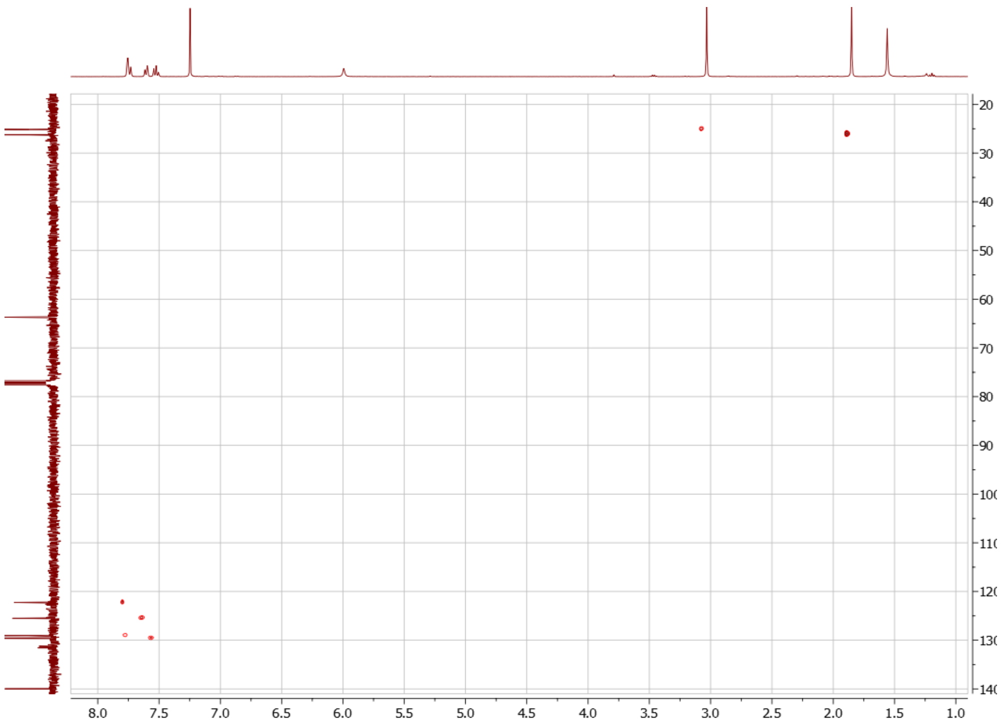

Supplement: Supplementary file 1 [file SC-010-C8SC05263H-s001.pdf]
